# Tankyrase inhibitor XAV-939 enhances osteoblastogenesis and mineralization of human skeletal (mesenchymal) stem cells

**DOI:** 10.1038/s41598-020-73439-9

**Published:** 2020-10-07

**Authors:** Nuha Almasoud, Sarah Binhamdan, Ghaydaa Younis, Hanouf Alaskar, Amal Alotaibi, Muthurangan Manikandan, Musaad Alfayez, Moustapha Kassem, Nihal AlMuraikhi

**Affiliations:** 1 Stem Cell Unit, Department of Anatomy, College of Medicine, King Saud University, Riyadh, 11461 Kingdom of Saudi Arabia; 2 College of Medicine, Alfaisal University, Riyadh, 11533 Kingdom of Saudi Arabia; 3 Science Department, College of Science, King Saud University, Riyadh, 11461 Kingdom of Saudi Arabia; 4 Molecular Endocrinology Unit (KMEB), Department of Endocrinology, University Hospital of Odense and University of Southern Denmark, Odense, Denmark; 5 Department of Cellular and Molecular Medicine, Danish Stem Cell Center (DanStem), University of Copenhagen, 2200 Copenhagen, Denmark

**Keywords:** Cell biology, Drug discovery, Molecular biology, Stem cells

## Abstract

Tankyrase is part of poly (ADP-ribose) polymerase superfamily required for numerous cellular and molecular processes. Tankyrase inhibition negatively regulates Wnt pathway. Thus, Tankyrase inhibitors have been extensively investigated for the treatment of clinical conditions associated with activated Wnt signaling such as cancer and fibrotic diseases. Moreover, Tankyrase inhibition has been recently reported to upregulate osteogenesis through the accumulation of SH3 domain-binding protein 2, an adaptor protein required for bone metabolism. In this study, we investigated the effect of Tankyrase inhibition in osteoblast differentiation of human skeletal (mesenchymal) stem cells (hMSCs). A Tankyrase inhibitor, XAV-939, identified during a functional library screening of small molecules. Alkaline phosphatase activity and Alizarin red staining were employed as markers for osteoblastic differentiation and in vitro mineralized matrix formation, respectively. Global gene expression profiling was performed using the Agilent microarray platform. XAV-939, a Tankyrase inhibitor, enhanced osteoblast differentiation of hBMSCs as evidenced by increased ALP activity, in vitro mineralized matrix formation, and upregulation of osteoblast-related gene expression. Global gene expression profiling of XAV-939-treated cells identified 847 upregulated and 614 downregulated mRNA transcripts, compared to vehicle-treated control cells. It also points towards possible changes in multiple signaling pathways, including TGFβ, insulin signaling, focal adhesion, estrogen metabolism, oxidative stress, RANK-RANKL (receptor activator of nuclear factor κB ligand) signaling, Vitamin D synthesis, IL6, and cytokines and inflammatory responses. Further bioinformatic analysis, employing Ingenuity Pathway Analysis identified significant enrichment in XAV-939-treated cells of functional categories and networks involved in TNF, NFκB, and STAT signaling. We identified a Tankyrase inhibitor (XAV-939) as a powerful enhancer of osteoblastic differentiation of hBMSC that may be useful as a therapeutic option for treating conditions associated with low bone formation.

## Introduction

Tankyrase is part of poly (ADP-ribose) polymerase (PARPs) superfamily of 17 physiological human PARPs required for numerous cellular and molecular processes^1–3^ including glucose metabolism, mitosis^4^, DNA damage repair^5^, genome stability^6^, cellular stress signaling^7^, signal transduction^6^, gene transcription^8^, telomere maintenance, and Wnt signaling^3^. Tankyrases are also promising drug targets that may be useful in several diseases affecting multiple organs^9,10^. The two tankyrase proteins, Tankyrase-1 and tankyrase-2, also called PARP5a and PARP5b, respectively, are members of the PARP family^3,4,11^. Structurally, both tankyrase proteins comprise ankyrin repeats, a sterile alpha module, and a carboxy-terminal PARP catalytic domain with an additional N-terminal HPS domain in Tankyrase-1^3,4,11^. Tankyrase inhibition negatively regulates Wnt pathway^12,13^. Thus, Tankyrase inhibitors have been extensively investigated for the treatment of clinical conditions associated with activated Wnt signaling and uncontrolled proliferation as a tumor suppression as in cancer^14^ including colon cancer^15^, lung cancer^16^ and breast cancer^12^, and fibrotic diseases like lung fibrosis^16–18^. In addition, Tankyrase inhibition has been recently reported to upregulate both osteoclastogenesis and osteoblastogenesis through the accumulation of SH3 domain-binding protein 2 (SH3BP2), an adaptor protein required for bone metabolism^19^, despite their Wnt Inhibitory effect^4^. SH3BP2 is important for the activation of the tyrosine kinase ABL, essential for osteoblast differentiation together with the transcriptional coactivator TAZ^4^. Tankyrase inhibitors were found to increase SH3BP2 and the nuclear expression of ABL, TAZ, and RUNX2 in murine primary calvaria cells, which consequently activated the ABL–TAZ complex, and therefore enhanced the osteoblast differentiation and maturation evidenced by the significant increase in the expression of osteoblast differentiation genes and mineral deposition^4,20–22^. However, the mechanism of Tankyrase signaling in bone metabolism remains to be elucidated.

Human skeletal (mesenchymal) stem cells (hMSCs) are multipotent stem cells that have the potential to proliferate and differentiate into various cell types including bone-forming osteoblasts^23,24^. The osteoblastic differentiation of hMSCs involves various signaling pathways including Tankyrase^4^, JAK-STAT signaling^25^, Wnt/β-catenin^26^, TGFβ^27^, Notch signaling^28^, and Hedgehog signaling^29^. However, the relative contribution of these signaling pathways on osteoblastic differentiation remained to be determined.

Small molecule inhibitors are currently employed as chemical tools to dissect the molecular mechanisms involved in stem cell differentiation to osteoblastic cells, which may help identifying new therapeutic targets^25,30^. We have previously reported the effects of a number of small molecules on differentiation potential of hMSCs into osteoblastic and adipocytic cells^31^. Here, we identified a small molecule XAV-939, through small molecules library screen, a potent Tankyrase inhibitor, as an enhancer of osteoblastic differentiation of hMSCs.

## Results

### XAV-939 enhances osteoblast differentiation of hMSCs

**Figure 1:**
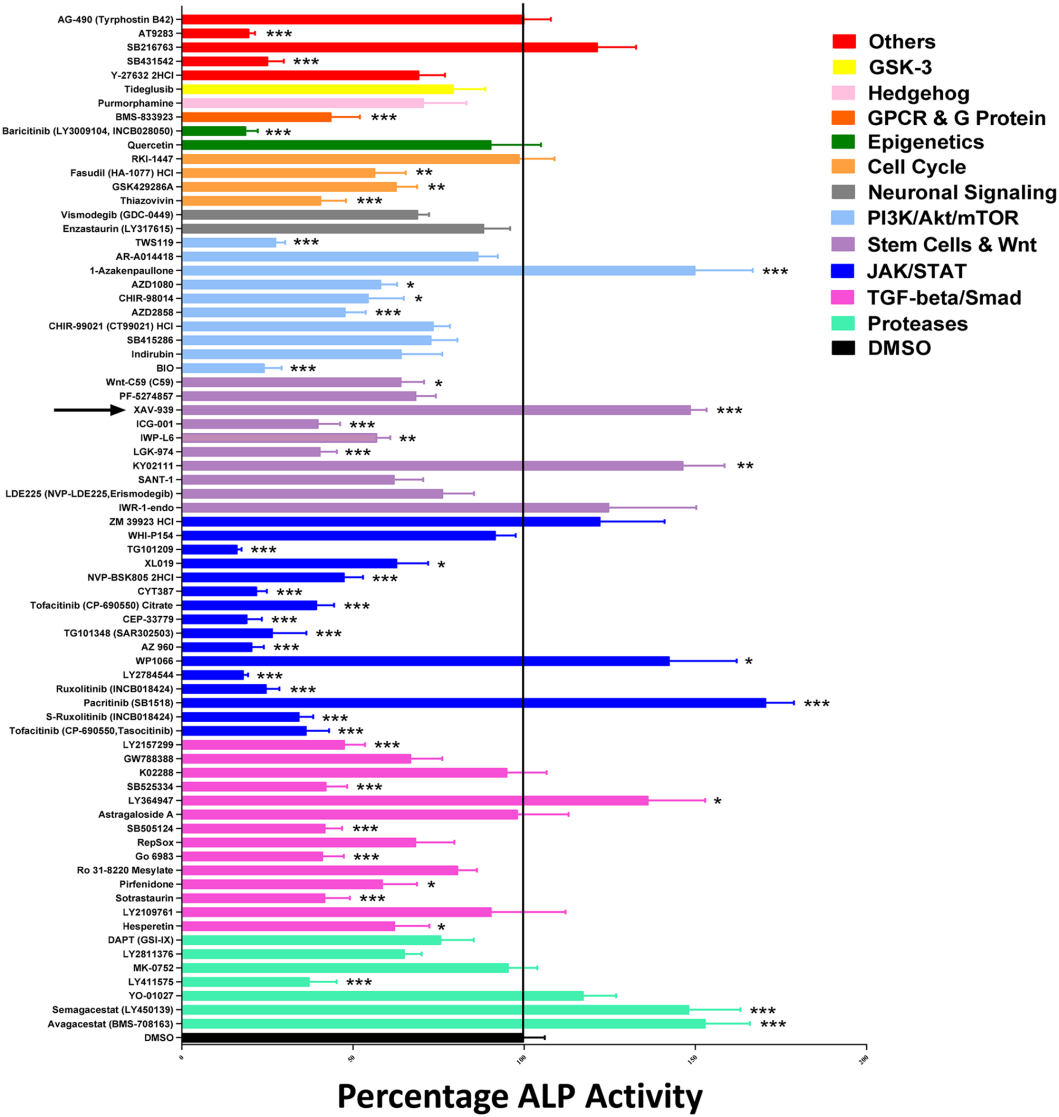
Functional screen of stem cell signaling small molecule library for their effects on osteoblast differentiation of hMSCs. hMSCs were induced into osteoblasts for 10 days in the presence of the indicated small molecule inhibitors (3.0 μM) or DMSO vehicle control. Data are presented as mean ALP activity ± SEM, n ≥ 6 from three independent experiments. Small molecules are grouped according to their targeted signaling pathway. *ALP* alkaline phosphatase, *DMSO* dimethyl sulfoxide. *p < 0.05; **p < 0.005; ***p < 0.0005.

**Figure 2:**
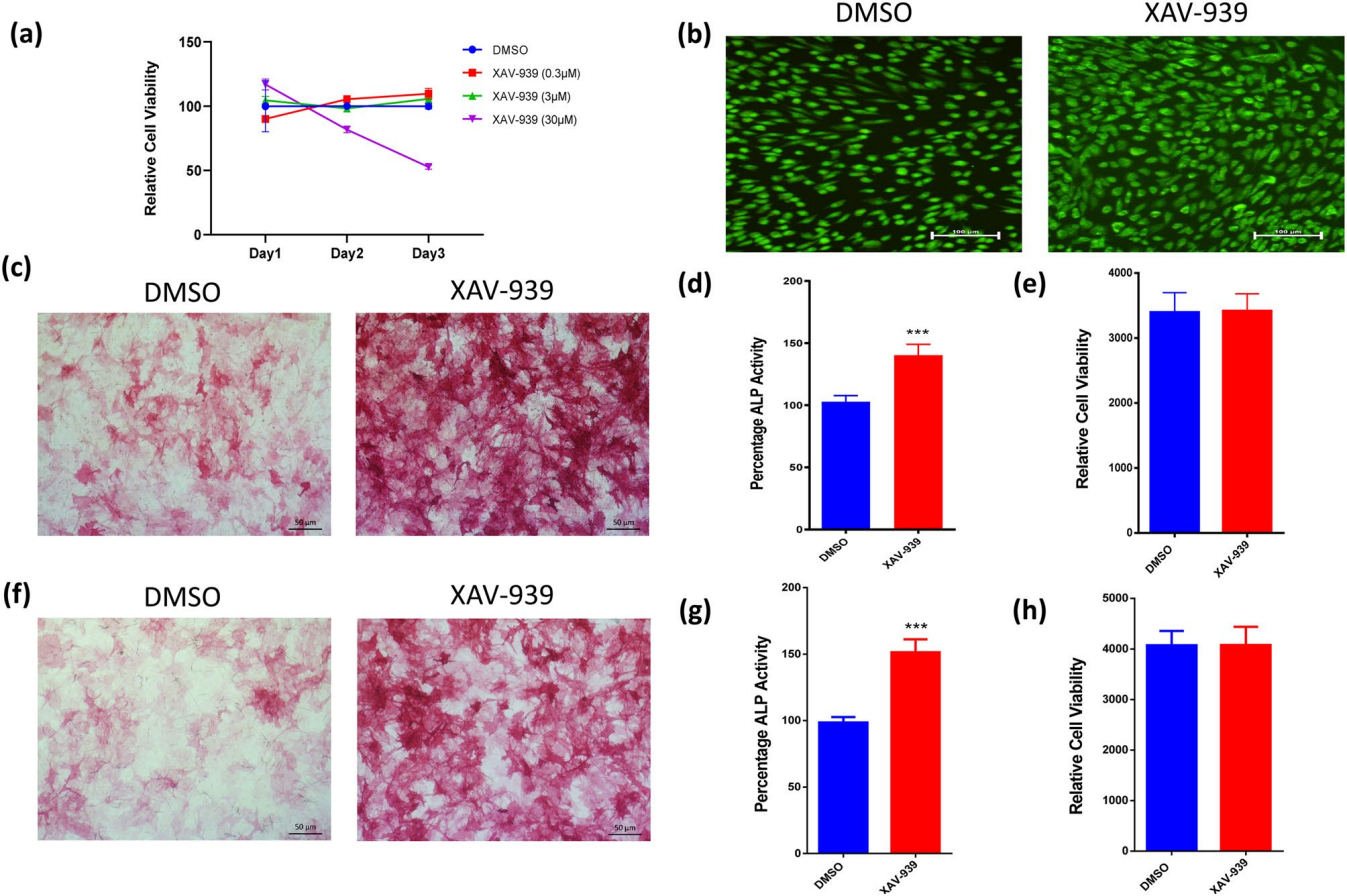
Effects of XAV-939 treatment on the osteoblast differentiation of hMSCs. (**a**) Dose–response proliferation curve of hMSCs to different doses of XAV-939 treatment, as indicated in the graph, versus DMSO-treated control cells as measured by cell viability over 3 days. (**b**) Representative fluorescence images of XAV-939-treated hBMSCs (3.0 µM) versus DMSO-treated control cells on day 3 after exposure. Photomicrographs magnification ×20. Cells were stained with AO/EtBr to detect apoptotic (cells with green condensed chromatin) and necrotic cells (red). (**c**) Representative alkaline phosphatase (ALP) staining of XAV-939-treated hBMSCs (3.0 µM) versus DMSO-treated control cells on day10 post-osteoblastic differentiation. Photomicrographs magnification ×10. (**d**) Quantification of ALP activity in XAV-939-treated hBMSCs (3.0 µM) versus DMSO-treated control cells on day10 post-osteoblastic differentiation. Data are presented as mean percentage ALP activity ± SEM (n = 20). (**e**) Assay for cell viability using Alamar Blue assay in XAV-939-treated hBMSCs (3.0 µM) versus DMSO-treated control cells on day10 post-osteoblastic differentiation. Data are presented as mean ± SEM (n = 20). (**f**) Validation of ALP staining in XAV-939-treated primary hBMSCs (3.0 µM) versus DMSO-treated primary hBMSCs control cells on day10 post-osteoblastic differentiation. Photomicrographs magnification ×10. (**g**) Validation of quantification of ALP activity in XAV-939-treated primary hBMSCs (3.0 µM) versus DMSO-treated primary hBMSCs control cells on day10 post-osteoblastic differentiation. Data are presented as mean percentage ALP activity ± SEM (n = 10). (**h**) Assay for cell viability using Alamar Blue assay in XAV-939-treated primary hBMSCs (3.0 µM) versus DMSO-treated primary hBMSCs control cells on day10 post-osteoblastic differentiation. Data are presented as mean ± SEM (n = 10). *ALP* alkaline phosphatase, *DMSO* dimethyl sulfoxide. *p < 0.05; **p < 0.005; ***p < 0.0005.

We have previously published the result of a small molecule library screen that identified several small molecule inhibitors with different effects on osteoblast differentiation of hMSCs using ALP activity quantification as a read-out^25^. Among these, XAV-939 exhibited potent enhancing effects, as shown in Fig. 1. Then, we performed a dose–response proliferation curve of hMSCs to XAV-939 treatment as measured by cell viability. Effect of different concentration of XAV-939 (0.3, 3, and 30 nM) on hMSCs proliferation was explored and the relative proliferation at day 1, 2, and 3 was plotted (Fig. 2a). There was no significant effect of XAV-939 on proliferation at day 1, 2, and 3 at dose of 0.3 and 3 μM. However, 30 μM XAV-939 inhibited hMSCs cell proliferation on day 3. Moreover, apoptosis assay was performed on day 3 after exposure of the cells to XAV-939 (3 µM), which showed a minute percentage of cell death (apoptosis and necrosis) in the XAV-939-treated hBMSC compared to DMSO-vehicle treated control cells (Fig. 2b).

**Figure 3:**
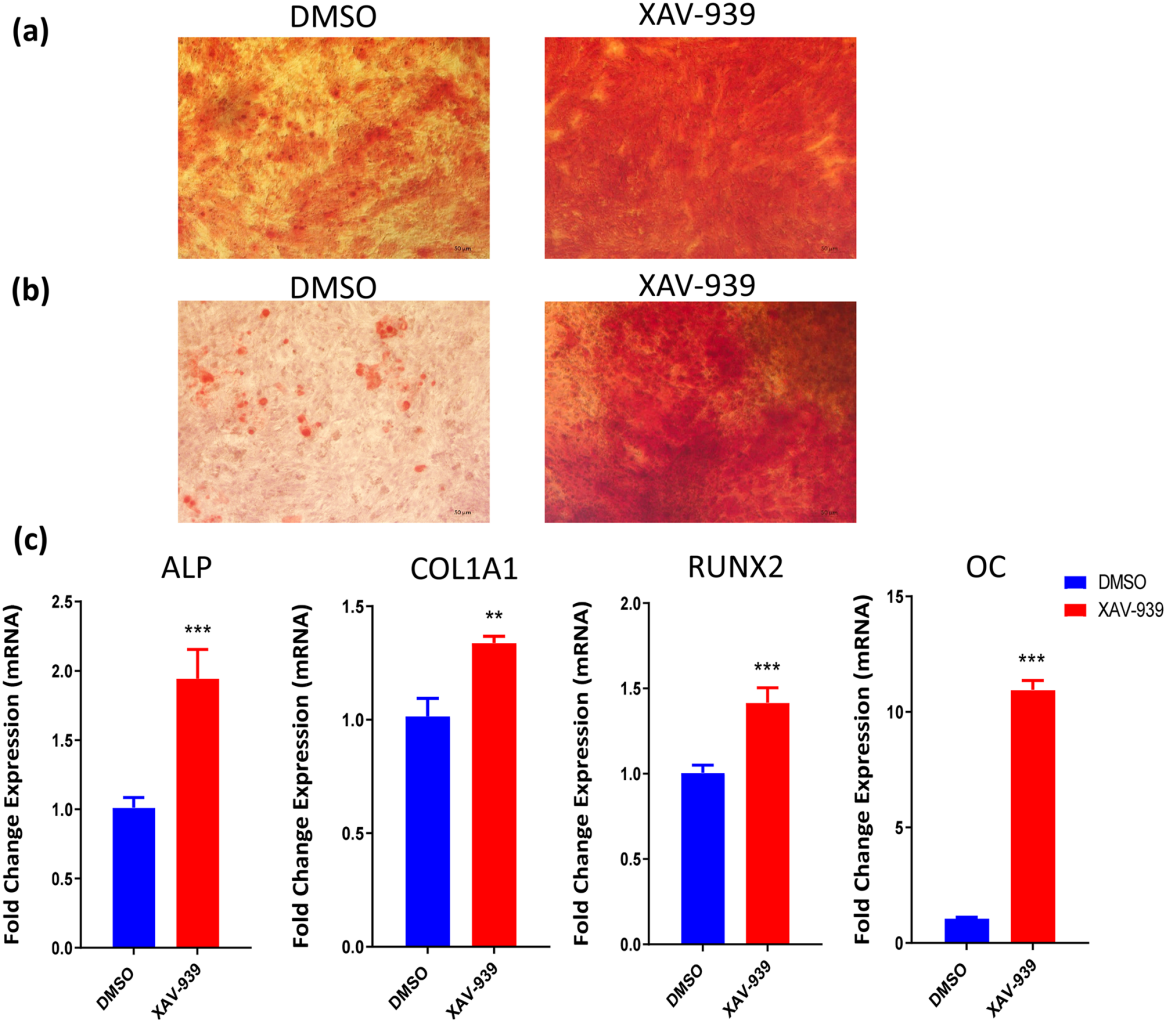
Effects of XAV-939 treatment on the mineralization and gene expression of hMSCs. (**a**) Cytochemical staining for mineralized matrix formation using Alizarin red stained on day 21 post-osteoblastic differentiation in the absence (left panel) or presence (right panel) of XAV-939 (3.0 µM). Photomicrographs magnification ×10. (**b**) Validation of Cytochemical staining for mineralized matrix formation using Alizarin red stained on day 21 post-osteoblastic differentiation in the absence (left panel) or presence (right panel) of XAV-939 (3.0 µM) in primary hBMSCs. Photomicrographs magnification ×10. (**c**) Quantitative RT-PCR analysis for gene expression of ALP, COL1A1, RUNX2 and OC in hBMSCs on day 10 post osteoblasts differentiation in the absence (blue) or presence (red) of XAV-939 (3.0 µM). Gene expression was normalized to β-actin. Data are presented as mean fold change ± SEM (n = 6) from two independent experiments; *p < 0.05; ***p ≤ 0.0005. *ALP* alkaline phosphatase, *COL1A1* Collagen Type I Alpha 1, *RUNX2* runt-related transcription factor 2, *OC* Osteocalcin, *DMSO* dimethyl sulfoxide.

hBMSCs exposed to XAV-939 (3 µM) showed a significant increase in ALP cytochemical staining intensity and ALP activity measurement compared to DMSO-vehicle treated control cells (Fig. 2c,d). In addition, XAV-939 did not exert significant effects on hBMSC viability on day 10 of osteoblastic differentiation (Fig. 2e). Furthermore, hBMSCs exposed to XAV-939 (3 µM) exhibited increased in mineralized matrix formation as evidenced by Alizarin red staining, compared to vehicle-treated control cells (Fig. 3a). To confirm our findings, we tested the effects of XAV-939 in primary normal hBMSCs. ALP cyochemical staining intensity (Fig. 2f), ALP activity measurement (Fig. 2g), cell viability using Alamar Blue assay (Fig. 2h), and cytochemical staining for mineralized matrix formation. Alizarin red (Fig. 3b) revealed enhanced osteoblast differentiation following treatment with XAV-939 (3 µM). Moreover, hBMSCs exposed to XAV-939 (3 µM) upregulated gene expression of osteoblast-associated gene markers including: ALP, COL1A1, RUNX2, and OC (Fig. 3c).

### XAV-939 promoted osteoblast differentiation of hMSCs via accumulation of SH3BP2

**Figure 4:**
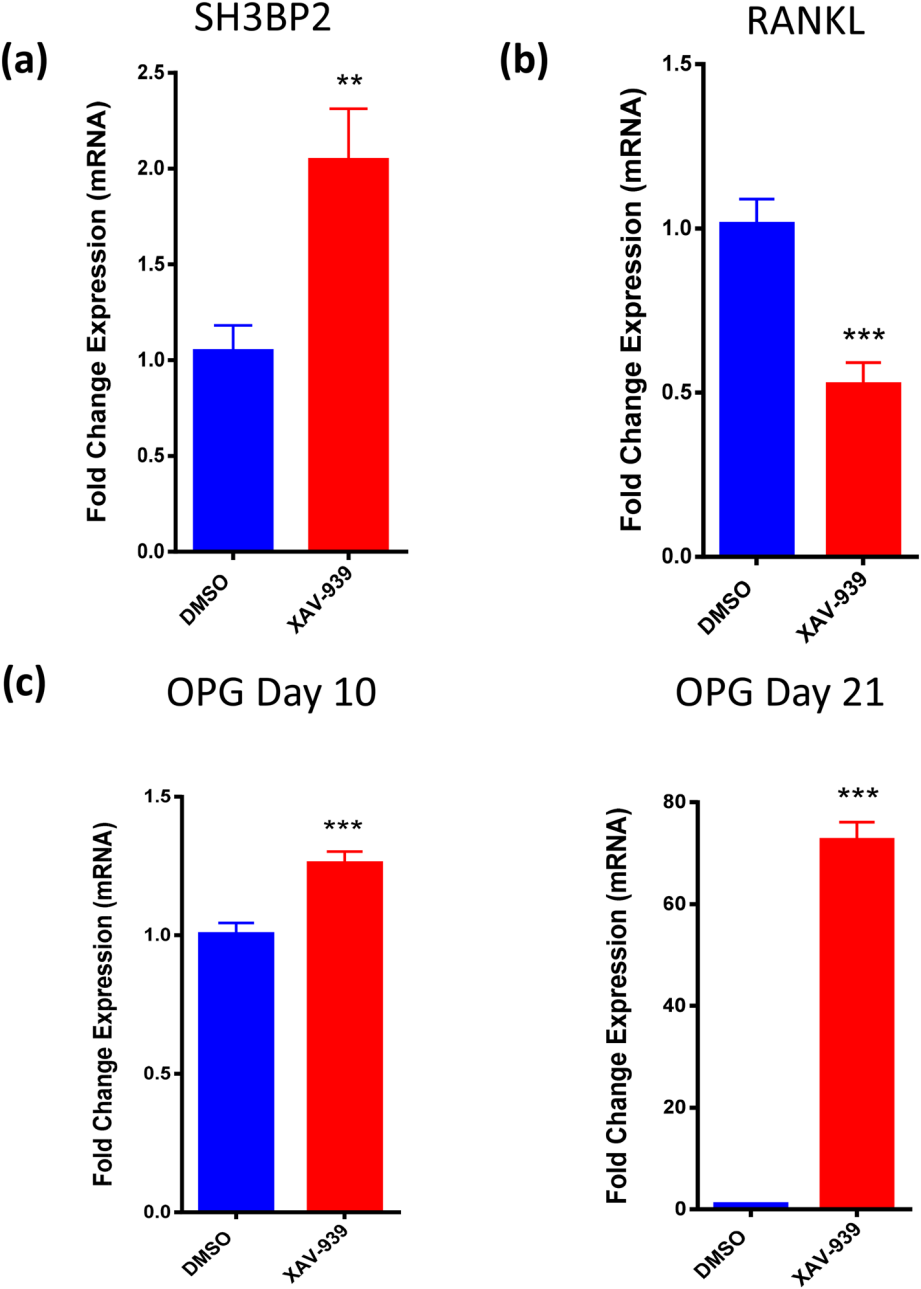
XAV-939 promotes osteoblast differentiation of hMSCs via accumulation of SH3BP2 and regulates the expression of osteoclastic regulatory molecules (RANKL and OPG) in hBMSCs during osteoblast differentiation. (**a**) Quantitative RT-PCR analysis for gene expression of SH3BP2 in hBMSCs on day 21 post osteoblasts differentiation in the absence (blue) or presence (red) of XAV-939 (3.0 µM). Quantitative RT-PCR analysis for gene expression of (**b**) RANKL on day 10 and (**c**) OPG on day 10 (left) and 21 (right) in hBMSCs post osteoblasts differentiation in the absence (blue) or presence (red) of XAV-939 (3.0 µM). Gene expression was normalized to β-actin. Data are presented as mean fold change ± SEM (n = 6) from two independent experiments; *p < 0.05; *** p ≤ 0.0005. *SH3BP2* SH3 domain-binding protein 2, *DMSO* dimethyl sulfoxide, *RANKL* receptor activator of nuclear factor κB ligand, *OPG* Osteoprotegerin, *DMSO* dimethyl sulfoxide.

Previous studies have reported that Tankyrase inhibition upregulate SH3BP2^1,4^, thus we examined gene expression of SH3BP2 in hBMSCs. Treatment with XAV-939 (3 µM) induced a significant upregulation in SH3BP2 gene expression compared to DMSO vehicle-treated control cells as determined at day 21 of osteoblastic differentiation (Fig. 4a).

### XAV-939 upregulated the expression of OPG while downregulated the expression of RANKL in hBMSCs during osteoblast differentiation

Osteoblasic cells mediate control of osteoclastic bone resorption through production of OPG and RANKL. To determine whether Tankyrase inhibition affects osteoblastic-osteoclastic interaction, we assessed gene expression of OPG and RANKL during osteoblast differentiation of hBMSCs and following treatment with XAV-939 (3 µM). As shown in Fig. 3b,c, XAV-939 treatment led to significant upregulation of OPG gene expression (Fig. 4c) and down-regulation of RANKL gene expression (Fig. 4b) as measured on day 10 of osteoblastic differentiation. Moreover, OPG remained significantly high later in the culture at day 21 (Fig. 4c).

### Global gene expression could point towards multiple differentially expressed signaling pathways in XAV-939-treated hBMSCs

**Figure 5:**
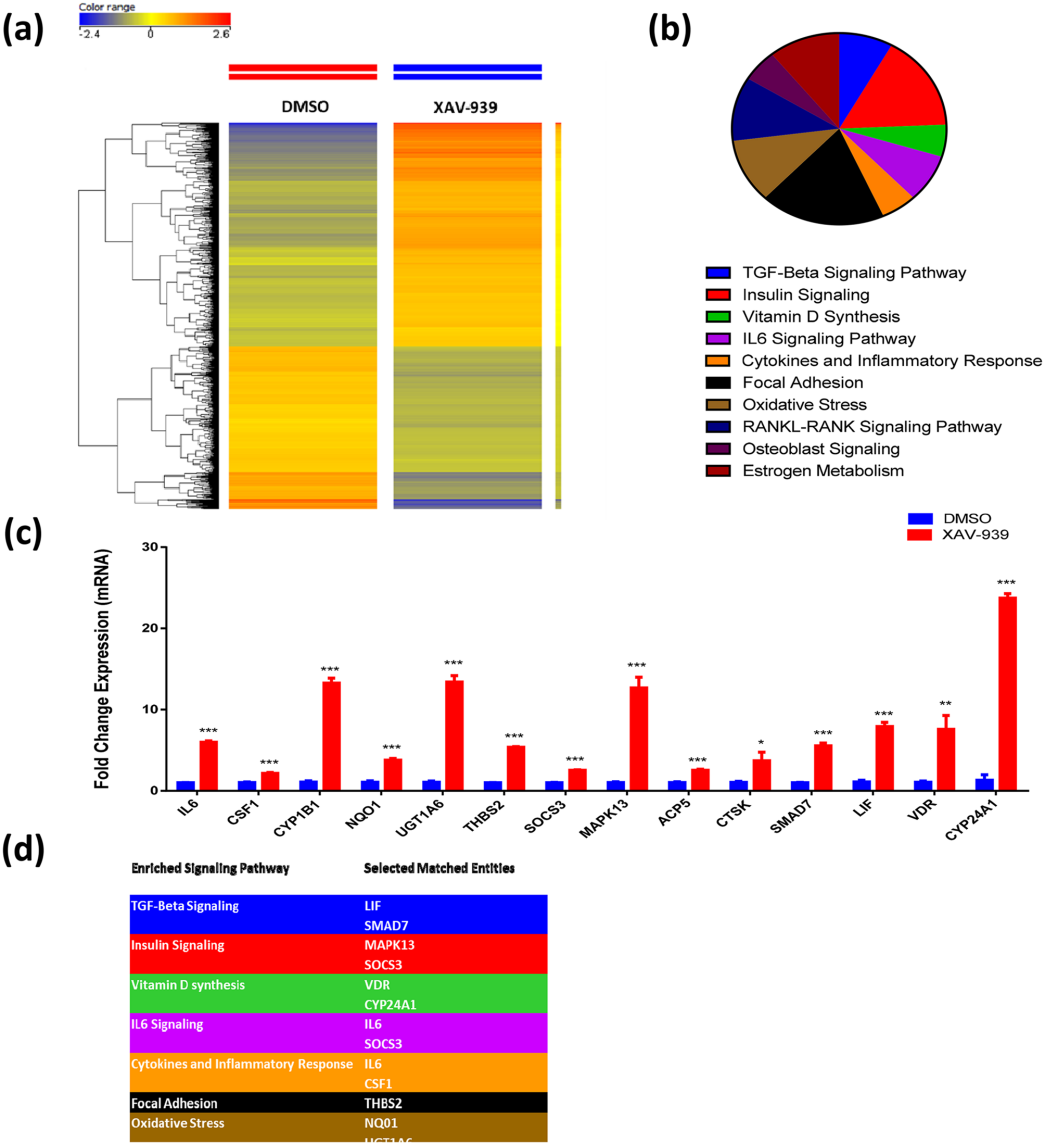
XAV-939 enhances expression of multiple signaling pathways in hBMSCs during osteoblast differentiation. (**a**) Heat-map and unsupervised hierarchical clustering performed on differentially expressed genes during osteoblastic differentiation of XAV-939-treated hBMSCs versus DMSO-treated control cells. (**b**) Pie chart demonstrating the distribution of selected signaling pathways enriched in the significantly up-regulated genes identified in XAV-939-treated hBMSCs versus DMSO-treated control cells. (**c**) Validation of a selected panel of upregulated genes in XAV-939-treated hBMSCs versus DMSO-treated control using qRT-PCR. Gene expression was normalized to β-actin. Data are presented as mean fold change ± SEM (n = 6) from two independent experiments; ***p < 0.0001. (**d**) Selected matched entities associated with the validated signaling pathways enriched in the significantly up-regulated genes identified in XAV-939-treated hBMSCs versus DMSO-treated control cells. Gene expression was normalized to β-actin. Data are presented as mean fold change ± SEM (n = 6) from two independent experiments; *p < 0.05; ***p ≤ 0.0005. *MAPK9* Mitogen-activated protein kinase 9, *SMAD4* mothers against decapentaplegic homolog 4, *DMSO* dimethyl sulfoxide.

**Figure 6:**
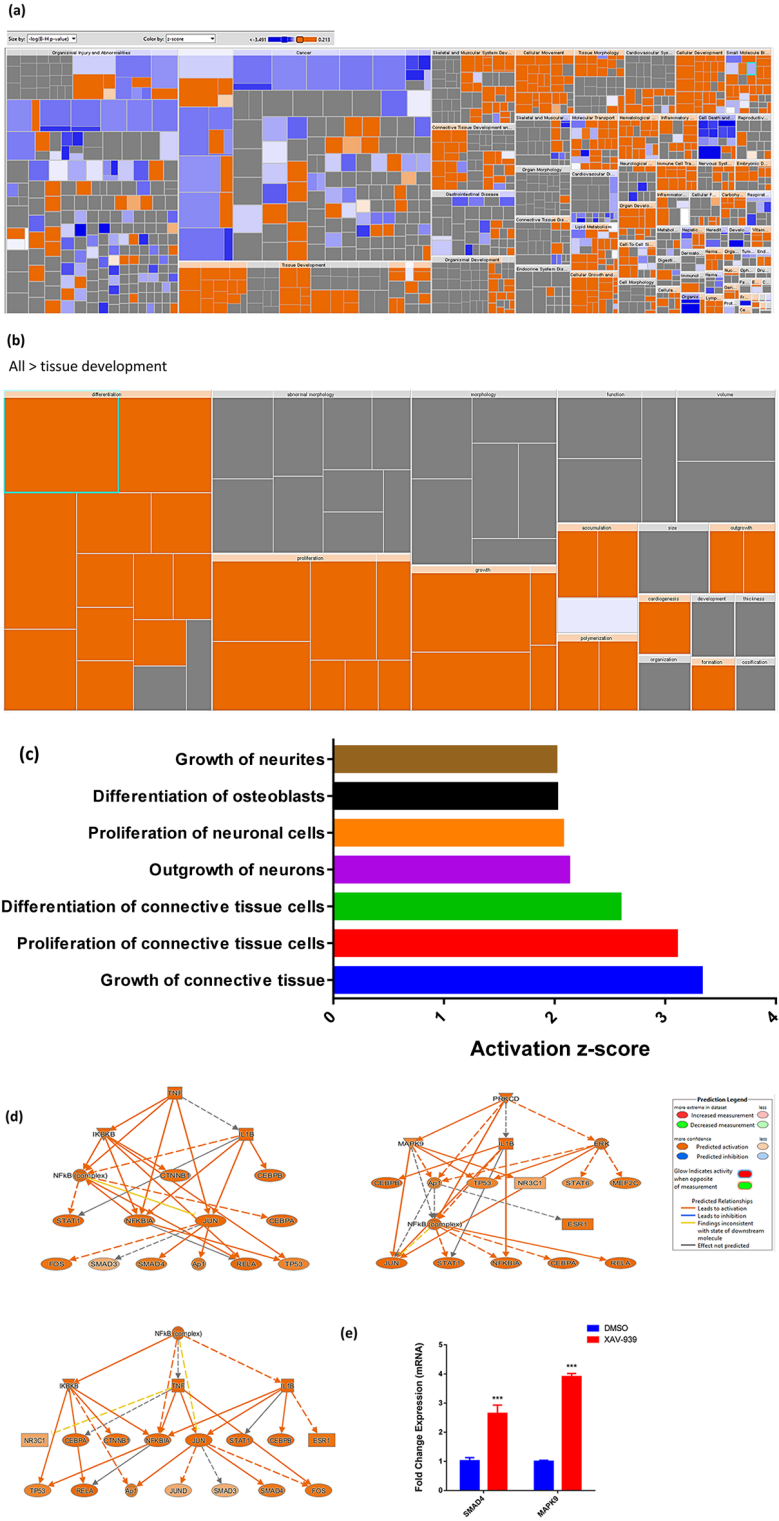
Bioinformatic analysis of signaling networks regulated in XAV-939-treated hBMSCs. (**a**) Disease and function heat map depicting activation (red) or inhibition (blue) of the indicated functional and disease categories identified in the downregulated transcripts in XAV-939-treated hBMSCs. (**b**,**c**) Heat map-illustrating affected tissue development functional category and associated functional annotations, respectively. (**d**) Illustration of the TNF, PRKCD, and NFκB (complex) genetic networks with predicted activated state based on transcriptome data with subsequent predicted effects on the STAT signaling. Figure legend illustrates the interaction between molecules within the network. (**e**) Validation of predicted activation effect on the downstream effector molecules SMAD4 and MAPK9 in XAV-939-treated hBMSCs versus DMSO-treated control using qRT-PCR on day 10 post osteoblasts differentiation in the absence (blue) or presence (red) of XAV-939 (3.0 µM). Gene expression was normalized to β-actin. Data are presented as mean fold change ± SEM (n = 6) from two independent experiments; *p < 0.05; ***p ≤ 0.0005. *MAPK9* Mitogen-activated protein kinase 9, *SMAD4* mothers against decapentaplegic homolog 4, *DMSO* dimethyl sulfoxide.

To understand the molecular mechanism by which XAV-939 enhances osteoblastic differentiation of hBMSCs, we performed global gene expression profiling followed by bioinformatics analysis of XAV-939-treated hBMSCs compared to vehicle-treated controls. Heat-map showed a large number of differentially expressed genes in XAV-939-treated compared to DMSO-treated control cells (Fig. 5a). We identified 847 upregulated and 614 downregulated genes (fold change ≥ 2.0; p (Corr) < 0.05) (Supplementary Table 1). Pathway analysis of the up-regulated genes identified several differentially regulated signaling pathways highly associated to osteoblastic differentiation including TGFβ, insulin signaling, focal adhesion, estrogen metabolism, oxidative stress, osteoblast signaling, RANK-RANKL signaling, Vitamin D synthesis, IL6, and cytokines and inflammatory responses (Fig. 5b,d). A number of genes from the enriched pathways (IL6, CSF1, CYP1B1, NQO1, UGT1A6, THBS2, SOCS3, MAPK13, ACP5, CTSK, SMAD7, LIF, VDR, and CYP24A1) were selected for a further validation using qRT-PCR, which was concordant with the microarray data (Fig. 5c,d). We subsequently determined the enriched functional categories and intracellular signaling networks regulated by XAV-939 during the osteogenic differentiation of hMSCs. The list of upregulated genes was subjected to core significance analysis using manually curated human functional category annotations and network databases (Ingenuity Pathway Analysis). Disease and functional analysis revealed a significant increase in the gene expression in different functional categories including those involved in tissue development (Fig. 6a–c). Follow-up upstream regulator analysis revealed a number of activated networks including TNF, PRKCD, and NFκB (complex), with a subsequent activation of STAT signaling (Fig. 6d). The predicted activated networks were further validated for both SMAD4 and MAPK9 activation using qRT-PCR, which was concordant with the transcriptome analysis (Fig. 6e). Our data suggest that XAV-939 regulates a number of signaling network beyond Tankyrase signaling to enhance osteoblastic differentiation of hBMSCs.

## Discussion

MSCs are multipotent stem cells in the bone marrow that can give rise to different mesodermal cell types including osteoblasts^23,24^. However, the molecular processes and signaling pathways involved in osteoblastic differentiation are being studied, in order to identify the novel molecular target for treatment of bone diseases^32,33^.

Small molecule inhibitors targeting intracellular signaling pathways have been employed as chemical tools to determine the molecular mechanisms controlling stem cell proliferation and differentiation^25,30^. Our group has employed this chemical biology approach to identify a number of molecular targets and pathways that are important for osteoblast and adipocyte differentiation of hMSCs^25,27–29,31,34,35^. In the current study, we identified XAV-939 small molecule inhibitor, during small molecule library functional screen, as an enhancer of osteoblast differentiation of hMSCs^25^.

We reported that XAV-939 treatment enhanced osteoblast differentiation and mineralization in vitro. Global gene expression profiling of hBMSC treated with XAV-939 identified significant enrichment in several osteoblast-associated signaling pathways including TGFβ^27^, insulin signaling^36^, focal adhesion^37^, estrogen metabolism^38^, oxidative stress^39^, RANK-RANKL signaling^40^, Vitamin D synthesis^41^, IL6 signaling^42^, and cytokines and inflammatory responses signaling^43^, corroborating the relevance of XAV-939 in enhancing bone formation.

We observed that XAV-939-treated hBMSCs exhibited significant upregulation in number of genes essential during normal bone repair and recruitment of progenitor cells for bone remodeling including MAPK9, which is involved in osteogenesis and found to enhance mineral deposition and late stages osteoblastic differentiation^44^, showed a significant upregulation in hBMSCs treated with XAV-939. Expression of SMAD4, which has a major role in regulating osteoblast viability and bone homeostasis^45^, was also significantly increased in hBMSCs treated with XAV-939. In addition, we identified TNF, NFκB, and STAT signaling among the top activated signaling pathways in XAV-939-treated hBMSCs; all are known to play a role in osteoblast differentiation and bone formation^29^.

PARPs, originally described as DNA repair enzymes, regulates various cellular functions including transcription, metabolism, and replication^46,47^. The effect of PARP in osteoblast differentiation is rather controversial, with some of the literature reporting an inhibitory effect of PARP inhibitors on osteoblasts. Kishi et al. reported that inhibition of PARP by the PARP inhibitor PJ34 suppresses osteogenic differentiation in mouse mesenchymal stem cells^48^. On the other hand, scattered studies, however, are in line with our study and describe that PARP inhibition may promote osteoblastic differentiation. Moreover, PARylation signaling controls osteogenic differentiation-associated cell death including inhibition of metabolic pathways and stimulation of cell death^47,49^. Dying cells at the terminal stage of differentiation release PARP that incorporate into the bone matrix and help calcification^47^. hMSCs stimulate osteoblast differentiation through activating the p38 MAPK pathway^50^. PARP inhibition impaired the activation of the downstream mediator, p38, leading to suppression of cell death and osteodifferentiation, mineralization, alkaline phosphatase activity, and marker gene expression^47,51^. In addition, PARP-1, a member of the PARP family, negatively regulates the expression of osteoclast-related upregulated genes in RANKL induction^52^. PARPs also have metabolic regulatory roles in adipocyte differentiation and were shown to modulate skeletal muscle myoblast differentiation^53^. PARPs are main factors in aging-related diseases such as neurodegenerative diseases and metabolic diseases^46^. Inhibition of PARP can restore endothelial function, neurovascular coupling responses and cognitive function in aged mice models that resemble features of brain dysfunctions observed in elderly patients^54^. In addition, it can inhibit neuroinflammation in animal models of Alzheimer’s disease^54^. In specific, Tankyrases are also involved in several biological processes including the positive regulation of Wnt/β-catenin pathway. Thus, inhibition of Tankyrase may potentially treat Wnt-dependent cancers^55^. Furthermore, Tankyrase inhibition in vitro enhance SH3BP2 expression^56^, which is required for normal bone homeostasis for both osteoblasts and osteoclasts via activation of the associated tyrosine kinases ABL in osteoblasts and Src in osteoclasts^57^.

Recently, Fujita et al.^1^ have reported that other tankyrase inhibitors promoted osteoblast differentiation and in vitro mineralization in spite of the observed inhibitory effects on Wnt/β-catenin signaling. The authors suggested that XAV-939 effects may be mediated by increased osteoblastic SH3BP2 which in turn stimulates a positive feedback loop of tyrosine kinase ABL, the transcriptional coactivator TAZ, and the RUNX2-TAZ complex necessary for osteoblastic cell differentiation^1,57–59^. Observation in the current study corroborate these findings as we reported that XAV-939 increased the expression of SH3BP2 and upregulated RUNX2 expression which may be caused by activation of ABL-TAZ complex^20–22^.

RANKL, which is expressed by osteoblast precursor^60^, is crucial for osteoclast differentiation and bone resorption. RANKL binds its cognate receptor RANK^60^, a cell surface receptor located on osteoclast lineages^61^. On the other hand, OPG (osteoprotegerin), a soluble decoy receptor for RANKL^62^ produced by osteoblastic cells^61^, interacts with RANKL, to inhibit RANKL/RANK binding, thus preventing subsequent osteoclast formation and bone resorption^62,63^. RANKL and OPG expressed differentially during osteoblastogenesis^60,64^. They both are expressed at the initiation of mineralization, RANKL then decreases in cells during the mineralization phase^64^, while OPG continue to increase during matrix formation, maturation, and mineralization phase^64^. In our study, we found a significant increase in the OPG expression along the differentiation together with a significant decrease in the expression of RANKL after the treatment with XAV-939. These findings suggest that XAV-939 enhanced osteoblast formation and maturation, and may also play a role in osteoblast-osteoclast interaction. These outcomes suggest that XAV-939 is an enhancer of osteoblast differentiation and inhibitor of osteoclast differentiation which is relevant for treating of bone diseases with impaired bone formation e.g. osteoporosis, and that is a novel addition to its possible use as a new therapeutic agent in clinical management of patients with breast cancer^65^.

## Materials and methods

### Cell culture

A hMSC-TERT cell line was used in all experiments of this study as a model for hBMSCs. The hMSC-TERT line was produced by overexpressing the human telomerase reverse transcriptase gene (hTERT). hMSC-TERT retains the typical features of primary hMSCs including unlimited self-renewal and multipotency, besides gene expression profile^66,67^. Normal human primary hBMSCs were purchased from Thermo Fisher Scientific Life Sciences.

The cells were maintained in Dulbecco modified eagle medium (DMEM), a basal medium supplemented with 4 mM l-glutamine, 4,500 mg/l d-glucose, and 110 mg/l 10% sodium pyruvate, in addition to 10% fetal bovine serum (FBS), 1% penicillin–streptomycin, and 1% non-essential amino acids as previously described^66^. All reagents were purchased from Thermo Fisher Scientific Life Sciences, Waltham, MA (https://www.thermofisher.com). Cells were incubated in 5% CO_2_ incubators at 37 °C and 95% humidity.

### Osteoblast differentiation

In accordance with our previously published protocol^25^, the cells were cultured until 80–90% confluency was reached then the medium was substituted with osteoblast induction medium (DMEM containing 10% FBS, 1% penicillin–streptomycin, 50 mg/ml l-ascorbic acid (Wako Chemicals GmbH, Neuss, Germany, https://www.wako-chemicals.de/), 10 mM b-glycerophosphate (Sigma-Aldrich), 10 nM calcitriol (1a,25-dihydroxyvitamin D3; Sigma-Aldrich), and 10 nM dexamethasone (Sigma-Aldrich). The stem cell signaling small molecule inhibitor library including XAV-939 were purchased from Selleckchem Inc. (Houston, TX, https://www.selleckchem.com). Small molecule inhibitors were added at a concentration of 3 µM to the osteoblast induction medium and cells were continuously exposed to the inhibitor over the differentiation period. Control cells were cultured with osteoblast induction medium containing dimethyl sulfoxide (DMSO) as vehicle.

### Cell viability assay

Cell viability assays was performed using alamarBlue assay, as previously described^25^ according to the manufacturer’s recommendations (Thermo Fisher Scientific). For dose–response growth curve, cells were cultured in 96-well plates in 300 μl of the medium in the presence of 0.3, 3, and 30 μM. XAV-939 compared to DMSO vehicle-treated control cells. On day 1, 2, and 3, 30 μl/well of alamarBlue substrate was added (10%) and plates were incubated for 1 h in the dark at 37 °C. Readings were obtained using BioTek Synergy II microplate reader (BioTek Inc., Winooski, VT, USA) at fluorescent mode (Ex 530 nm/Em 590 nm). For cell viability, cells were cultured in 96-well plates in 300 μl of the medium. On day10, 30 μl/well of alamarBlue substrate was added (10%) and plates were incubated for 1 h in the dark at 37 °C. Readings were obtained using BioTek Synergy II microplate reader (BioTek Inc., Winooski, VT, USA) at fluorescent mode (Ex 530 nm/Em 590 nm).

### Measurement of apoptosis

A fluorescence-based apoptosis assay using the acridine orange/ethidium bromide (AO/EtBr) staining method was performed as previously described^68^ after exposure of the cells to XAV-939 (3 µM) compared to DMSO-vehicle treated control cells. On day 3, cells were stained with dual fluorescent staining solution (1.0 µl) containing 100 µg/ml AO and 100 µg/ml EtBr (AO/EB, Sigma, St. Louis, MO, USA). Cells were mixed with AO/EtBr (1:100) dye solution for 1 min before they were imaged under a Nikon Eclipse Ti fluorescence microscope (Nikon, Tokyo, Japan).

### Quantification of alkaline phosphatase activity

Alkaline phosphatase (ALP) activity was quantified using the BioVision ALP activity colorimetric assay kit (BioVision, Inc., Milpitas, CA, https://www.biovision.com/) with some modifications as previously described^25^. The cells were cultured in 96-well plates. On day 10 of osteoblast differentiation, the cells were rinsed once with PBS and fixed with 3.7% formaldehyde in 90% ethanol for 30 s at room temperature. Fixative was removed and 50 µl/well of p-nitrophenyl phosphate solution was added and incubated for 30–60 min. Optical densities were then measured at 405 nm using a SpectraMax/M5 fluorescence spectrophotometer plate reader, and ALP enzymatic activity was then normalized to cell number.

### Alkaline phosphatase staining

Cells were cultured in a 6-well plate in osteoblast differentiation medium. In accordance with our previously published protocols^25^, on day 10, the cells were washed in PBS and fixed in 10 mM acetone/citrate buffer at pH 4.2 for 5 min at room temperature. The fixative was removed and Naphthol/Fast Red stain [0.2 mg/mL Naphthol AS-TR phosphate substrate (Sigma)] [0.417 mg/mL of Fast Red (Sigma)] was added for 1 h at room temperature. Then, cells were washed with water 3 times and images were taken under the microscope.

### Alizarin Red S Staining for mineralized matrix formation

In accordance with our previously published protocols^25^, on day 21 of osteoblast differentiation, cells were washed twice with PBS and fixed with 4% paraformaldehyde for 10 min at room temperature. The fixative was rinsed and the cells were then washed 3 times with distilled water and stained with the 2% Alizarin Red S Staining Kit (ScienceCell, Research Laboratories, Cat. No. 0223) for 10–20 min at room temperature. Later, the cells were washed with water and images were taken under the microscope.

### RNA extraction and cDNA synthesis

Total RNA was isolated from cell pellets on day 10 of osteoblast differentiation using the total RNA Purification Kit (Norgen Biotek Corp., Thorold, ON, Canada, https://norgenbiotek.com/) according to the manufacturer’s instructions as previously described^25^. The concentrations of total RNA extracted were measured using NanoDrop 2000 (ThermoFisher Scientific Life Sciences). cDNA was synthesized with 500 ng of total RNA using High Capacity cDNA Transcription Kit (ThermoFisher Scientific Life Sciences) according to manufacturer’s instructions.

### Quantitative real time-polymerase chain reaction

Quantitative Real Time-Polymerase Chain Reaction (RT-PCR) was performed using fast SYBR Green in Applied Biosystems ViiA 7 Real-Time PCR System (ThermoFisher Scientific Life Sciences). Primers used in this study are listed in Table 1. Relative expression was calculated using the 2∆CT value method, and analysis was made as previously described^69^.

**Table 1:** List of SYBR Green primers used in current study.

Gene name	Forward primer	Reverse primer
ACTB	5′AGCCATGTACGTTGCTA	5′AGTCCGCCTAGAAGCA
ALPL	5′ GGA ACT CCT GAC CCT TGA CC 3′	5′ TCC TGT TCA GCT CGT ACT GC 3′
COL1A1	5′GAGTGCTGTCCCGTCTGC 3′	5′TTTCTTGGTCGGTGGGTG 3′
OC	GGCAGCGAGGTAGTGAAGAG	CTCACACACCTCCCTCCTG
RUNX2	5′GTAGATGGACCTCGGGAACC3′	5′GAGGCGGTCAGAGAACAAAC3′
LIF	5′GCCACCCATGTCACAACAAC	5′CCCCCTGGGCTGTGTAATAG
VDR	CTCTGATAGCCTCATGCCAGG	ACCCAAAGGCTTCCCAAAGAG
CYP24A1	AGCGATAATACGCCTCAGATGG	GATGGTGCTGACACAGGTGA
CYP1B1	GCAAGGGCATGGGAATTGAC	TGGTGCCCATGCTGCG
IL6	CGAGCCCACCGGGAACGAAA	GGACCGAAGGCGTTGTGGAG
THBS2	5′TTGGCAAACCAGGAGCTCAG3′	5′GGTCTTGCGGTTGATGTTGC3′
SOCS3	5′TTCGGGACCAGCCCCC3′	5′AAACTTGCTGTGGGTGACCA3′
NQO1	TGAAAGGCTGGTTTGAGCGA	GCCTTCTTACTCCGGAAGGG
UGT1A6	CTCGCATCAGCTGTCCTCAA	AGGCTTCAAATTCCTGAGACAAGT
CSF1	GCCAGTGAGATTCCCGTACC	TGCCTCTCATGGCCAGTTAC
SMAD7	CCCATCACCTTAGCCGACTC	TGGACAGTCTGCAGTTGGTTT
MAPK13	GGACGTCAACAAGACAGCCT	TTGGCGAAGATCTCGGACTG
ACP5	CTACCCACTGCCTGGTCAAG	TCCATGAAATTCCCAGCCCC
CTSK	GGGGGACATGACCAGTGAAG	CAGAGTCTGGGGCTCTACCT
OPG	5′TGTCCAGATGGGTTCTTCTCA3′	5′CGTTGTCATGTGTTGCATTTCC3′
RANKL	5′TGAAGACACACTACCTGACTCCTG3′	5′CCACAATGTGTTGCAGTTCC3′
SH3BP2	GAACATGAAGATGAGGACGACTC	GGCTGGTGGATAGGTCAGG
MAPK9	TGGGCTACAAAGAGAACGTTGAT	AAGGTCGCGGGGAAGGATA
SMAD4	GGATACGTGGACCCTTCTGG	TGTGCAACCTTGCTCTCTCAA

### Gene expression profiling by microarray

One hundred fifty nanograms of total RNA from day 10 of osteoblast differentiation were labeled using a low input Quick Amp Labeling Kit (Agilent Technologies, Santa Clara, CA, https://www.agilent.com) and hybridized to the Agilent Human SurePrint G3 Human GE 8 × 60 k microarray chip. All microarray experiments were performed at the Microarray Core Facility (Stem Cell Unit, Department of Anatomy, King Saud University College of Medicine, Riyadh, Saudi Arabia). Data were normalized and evaluated using GeneSpring 13.0 software (Agilent Technologies). Pathway analyses was concluded using GeneSpring 13.0 as defined previously^70^. Two-fold cutoff and p(corr) < 0.05 (Benjamini–Hochberg multiple testing corrected) were used to define significantly changed transcripts. Pathway and functional annotation analyses were conducted using the Ingenuity Pathway Analysis (Ingenuity Systems, https://www.ingenuity.com/)^29,71^. upregulated genes ≤ 2 FC (fold change) and corrected p value < 0.05 were chosen for analysis. Enriched network categories were algorithmically generated based on their connectivity and ranked according to Z score.

### Statistical analysis

Statistical analysis and graphing were performed using Microsoft Excel 2010 and GraphPad Prism 6 software (GraphPad software, San Diego, CA, USA), respectively. Results obtained were shown as mean ± SEM from at least two independent experiments. Unpaired, two-tailed Student t-test was used to determine statistical significance and p-values < 0.05 was considered statistically significant.

## Supplementary information

Supplementary Information.

## Data Availability

All data generated or analysed during this study are included in this published article (and its Supplementary Information files).

## Abbreviations

hBMSCs: Human bone marrow skeletal (mesenchymal) stromal cell
hTERT: Human telomerase reverse transcriptase
qRT-PCR: Quantitative reverse transcriptase-polymerase chain reaction
PARP: Poly (ADP-ribose) polymerase
SH3BP2: SH3 domain-binding protein 2
DMSO: Dimethyl sulfoxide
DMEM: Dulbecco’s modified Eagle’s medium
PBS: Phosphate-buffered saline
ALP: Alkaline phosphatase
RUNX2: Runt-related transcription factor 2
COL1A1: Collagen Type I Alpha 1
OC: Osteocalcin
ALZR: Alizarin red
RANKL: Receptor activator of nuclear factor κB ligand
RANK: Receptor activator of nuclear factor κB
OPG: Osteoprotegerin
MAPK9: Mitogen-activated protein kinase 9
SMAD4: Mothers against decapentaplegic homolog 4
