# Author Correction: Tankyrase inhibitor XAV-939 enhances osteoblastogenesis and mineralization of human skeletal (mesenchymal) stem cells

**DOI:** 10.1038/s41598-021-83943-1

**Published:** 2021-02-19

**Authors:** Nuha Almasoud, Sarah Binhamdan, Ghaydaa Younis, Hanouf Alaskar, Amal Alotaibi, Muthurangan Manikandan, Musaad Alfayez, Moustapha Kassem, Nihal AlMuraikhi

**Affiliations:** 1 Stem Cell Unit, Department of Anatomy, College of Medicine, King Saud University, Riyadh, 11461 Kingdom of Saudi Arabia; 2 College of Medicine, Alfaisal University, Riyadh, 11533 Kingdom of Saudi Arabia; 3 Science Department, College of Science, King Saud University, Riyadh, 11461 Kingdom of Saudi Arabia; 4 Molecular Endocrinology Unit (KMEB), Department of Endocrinology, University Hospital of Odense and University of Southern Denmark, Odense, Denmark; 5 Department of Cellular and Molecular Medicine, Danish Stem Cell Center (DanStem), University of Copenhagen, 2200 Copenhagen, Denmark

Correction to: *Scientific Reports* 10.1038/s41598-020-73439-9, published online 07 October 2020

The original version of this Article omitted an affiliation for Moustapha Kassem. The correct affiliations for Moustapha Kassem are listed below:

Stem Cell Unit, Department of Anatomy, College of Medicine, King Saud University, Riyadh, 11461, Kingdom of Saudi Arabia

Molecular Endocrinology Unit (KMEB), Department of Endocrinology, University Hospital of Odense and University of Southern Denmark, Odense, Denmark

Department of Cellular and Molecular Medicine, Danish Stem Cell Center (DanStem), University of Copenhagen, 2200, Copenhagen, Denmark

Additionally, Nihal AlMuraikhi was incorrectly affiliated with “Department of Cellular and Molecular Medicine, Danish Stem Cell Center (DanStem), University of Copenhagen, 2200, Copenhagen, Denmark”. The correct affiliation is listed below.

Stem Cell Unit, Department of Anatomy, College of Medicine, King Saud University, Riyadh, 11461, Kingdom of Saudi Arabia

This error has now been corrected in the PDF and HTML versions of the Article.

