# Supplementary material for: Tankyrase inhibitor XAV-939 enhances osteoblastogenesis and mineralization of human skeletal (mesenchymal) stem cells
Source: Sci Rep. 2020 Oct 7;10:16746. doi: 10.1038/s41598-020-73439-9 (PMC7541626; doi:10.1038/s41598-020-73439-9)
Supplement: Supplementary file 1 — Supplementary Information. [file 41598_2020_73439_MOESM1_ESM.pdf]

Title: Tankyrase Inhibition Via XAV-939 Enhances Osteoblastogenesis and Mineralization of Human Skeletal (Mesenchymal) Stem Cells

Nuha Almasoud, Sarah Binhamdan, Ghaydaa Younis, Hanouf Alaskar, Amal Alotaibi, Muthurangan Manikandan, Musaad Alfayez, Moustapha Kassem, Nihal AlMuraikhi

Supplementary Table 1. List of differentiatlly expressed genes (2.0 FC, p corr <0.05) in human bone marrow mesenchymal stem cells (hBMSCs) differentiated into osteoblasts (day 10) in the presence of XAV-939 compared to DMSO

ProbeNam	FC ([XAV-9	Log FC ([XA	FC (abs) ([X	Regulation	US1049385	US1049385	US1049385	US1049385	US1049385	US1049385	GeneSymb	GeneName	ControlTyp	Descriptior	EntrezGene	Go	Chromosor	Chromosor	Chromosor	Chromosor	Cytoband	GenbankA	GenomicC	RefSeqAcc	UniGeneID
GE_BrightC	3.441975	1.783237	3.441975	up	-0.818802	-0.923452	-0.822461	0.927212	0.818803	1.038981			pos	Unknown											
A_21_P000	2.183423	1.126591	2.183423	up	-0.567207	-0.566665	-0.541161	0.569862	0.54116	0.59372	MTRNR2L2	MT-RNR2-l	FALSE	Homo sapi	1E+08	GO:000573	79945878	chr5	79945819	-	hs|5q14.1	NM_00119	chr5:79945	NM_00119	Hs.666077
A_22_P000	2.569926	1.361727	2.569926	up	-0.640225	-0.69492	-0.614405	0.844059	0.677166	0.614405	lnc-OST4-2	lnc-OST4-2	FALSE	LNCipedia lincRNA (lnc-OST4-2), lir			27237428	chr2	27237369	-	hs|2p23.3	AK125769	chr2:27237428-27237		Hs.666730
A_23_P156	3.629815	1.859896	3.629815	up	-0.980624	-1.076739	-0.826394	0.839187	1.03035	0.826394	TNXB	tenascin XE	FALSE	Homo sapi	7148	GO:003003	32009022	chr6	32008964	-	hs|6p21.33	NM_03247	chr6:32009	NM_03247	Hs.485104
A_33_P321	3.481015	1.799508	3.481015	up	-0.874826	-1.091875	-0.692217	0.937393	0.692218	1.109995	SCGB3A1	secretoglot	FALSE	Homo sapi	92304	GO:003030	1.8E+08	chr5	1.8E+08	-	hs|5q35.3	NM_05286	chr5:18001	NM_05286	Hs.62492
A_23_P126	2.072537	1.051398	2.072537	up	-0.363703	-0.357299	-0.382668	0.819484	0.357299	0.87374	PGD	phosphoglu	FALSE	Homo sapi	5226	GO:005066	10478970	chr1	10478911	+	hs|1p36.22	NM_00263	chr1:10478	NM_00263	Hs.464071
A_21_P001	2.878898	1.525517	2.878898	up	-0.804823	-0.886235	-0.694154	0.71152	0.785664	0.694154	XLOC_l2_004844		FALSE	BROAD Institute lincRNA (XLOC_l2			23186602	chr15	23186543	-	hs|15q11.2		chr15:23186602-23186543		
A_23_P427	2.068874	1.048846	2.068874	up	-0.57426	-0.508175	-0.510192	0.533648	0.508175	0.512089	MT1L	metallothic	FALSE	Homo sapi	4500		56652639	chr16	56652580	+	hs|16q12.2	NR_00144	chr16:5665	NR_00144	Hs.647358
A_24_P152	3.188497	1.672877	3.188497	up	-0.670757	-0.982821	-1.098899	0.882928	0.670757	0.712467			FALSE				1.32E+08	chr5	1.32E+08	+	hs|5q31.1		chr5:132279661-132279720		
A_23_P163	2.455948	1.29628	2.455948	up	-0.537689	-0.754233	-0.517342	0.633381	0.928853	0.517342	CGNL1	cingulin-lik	FALSE	Homo sapi	84952	GO:000377	57842906	chr15	57842847	+	hs|15q21.3	NM_03286	chr15:5784	NM_03286	Hs.148989
A_23_P412	3.085543	1.625524	3.085543	up	-0.586779	-1.114485	-1.169916	0.727591	0.586779	0.691022	ANKRD29	ankyrin rep	FALSE	Homo sapi	147463		21180440	chr18	21180381	-	hs|18q11.2	NM_17350	chr18:2118	NM_17350	Hs.374774
A_22_P000	3.89991	1.963441	3.89991	up	-0.670175	-1.387704	-0.818965	1.195755	0.670175	1.147549	LINC00271	long interg	FALSE	Homo sapi	1E+08		1.36E+08	chr6	1.36E+08	+	hs|6q23.3	NR_02680	chr6:13581	NR_02680	Hs.734007
A_23_P943	2.615885	1.387299	2.615885	up	-0.608797	-0.580119	-0.641018	0.835118	0.580119	0.916725	ENPP2	ectonucleo	FALSE	Homo sapi	5168	GO:000588	1.21E+08	chr8	1.21E+08	-	hs|8q24.12	NM_00620	chr8:12058	NM_00620	Hs.190977
A_23_P200	3.416705	1.772606	3.416705	up	-0.931493	-0.655386	-0.86572	1.155506	0.655386	1.054327	TGFBR3	transformi	FALSE	Homo sapi	7049	GO:000551	92148947	chr1	92148888	-	hs|1p22.1	NM_00324	chr1:92148	NM_00324	Hs.482390
A_23_P614	2.615478	1.387075	2.615478	up	-0.596031	-0.733852	-0.646562	0.840957	0.596031	0.747792	CD163L1	CD163 mol	FALSE	Homo sapi	283316	GO:000588	7521549	chr12	7520755	-	hs|12p13.3	NM_17494	chr12:7521	NM_17494	Hs.631727
A_32_P729	2.219792	1.150424	2.219792	up	-0.605254	-0.521029	-0.617544	0.54808	0.638337	0.521029	RPL35	ribosomal p	FALSE	Homo sapi	11224	GO:001046	1.28E+08	chr9	1.28E+08	-	hs|9q33.3	NM_00720	chr9:12762	NM_00720	Hs.182825
A_22_P000	6.067785	2.60117	6.067785	up	-1.199405	-1.36013	-1.142458	1.462291	1.142458	1.496767	HGC6.3	uncharacte	FALSE	Homo sapi	1E+08		1.68E+08	chr6	1.68E+08	-	hs|6q27	NM_00112	chr6:16837	NM_00112	Hs.448059
A_33_P331	2.31564	1.211411	2.31564	up	-0.634649	-0.520064	-0.544899	0.614306	0.520064	0.800251			FALSE				1.01E+08	chr7	1.01E+08	+	hs|7q22.1		chr7:101286790-101286849		
A_22_P000	9.784171	3.29045	9.784171	up	-1.190116	-1.864123	-1.737698	2.013718	1.190115	1.875579	lnc-TRA2A-	lnc-TRA2A-	FALSE	Q4G0Q7_HUMAN (Q4G0Q7) MGC			23719873	chr7	23719814	-	hs|7p15.3		chr7:23719873-23719814		
A_24_P502	2.01761	1.012648	2.01761	up	-0.630767	-0.316161	-0.434582	0.606415	0.316161	0.733856	HLA-DMA	major histc	FALSE	Homo sapi	3108	GO:004883	32917477	chr6	32917418	-	hs|6p21.32	NM_00612	chr6:32917	NM_00612	Hs.728759
A_19_P003	9.072103	3.181437	9.072103	up	-1.328174	-1.52688	-1.465448	1.920905	1.328175	1.974729	SNHG11	small nucle	FALSE	Homo sapi	128439		37075436	chr20	37075377	+	hs|20q11.2	NR_00323	chr20:3707	NR_00323	Hs.16936
A_22_P000	2.344406	1.229222	2.344406	up	-0.394407	-0.870373	-0.989433	0.474333	0.564714	0.394407	LOC10272	uncharacte	FALSE	Homo sapi	1.03E+08		4000337	chr16	4000278	-	hs|16p13.3	NR_12031	chr16:4000	NR_12031	Hs.364739
A_23_P374	2.063236	1.044909	2.063236	up	-0.59421	-0.645719	-0.426562	0.426562	0.494166	0.547508	SH3KBP1	SH3-domai	FALSE	Homo sapi	30011	GO:000551	19554143	chrX	19554084	-	hs|Xp22.12	NM_00102	chrX:19554	NM_00102	Hs.726365
A_23_P257	4.133847	2.047485	4.133847	up	-1.088094	-0.890399	-1.098434	0.890399	1.194842	0.980287	CLMN	calmin (cal	FALSE	Homo sapi	79789	GO:000573	95660925	chr14	95660250	-	hs|14q32.1	NM_02473	chr14:9566	NM_02473	Hs.301478
A_22_P000	2.714502	1.440688	2.714502	up	-0.71077	-0.676054	-0.596547	0.596547	0.807095	0.935049	lnc-GSTO2-	lnc-GSTO2-	FALSE	Q2U8N5_ASPOR (Q2U8N5) Predic			1.06E+08	chr10	1.06E+08	+	hs|10q25.1		chr10:106098140-106098199		
A_33_P323	2.545891	1.348171	2.545891	up	-0.660595	-0.606861	-0.488796	0.817416	0.488796	0.982046	C1S	compleme	FALSE	Homo sapi	716	GO:000551	7171652	chr12	7171593	+	hs|12p13.3	NM_20144	chr12:7171	NM_20144	Hs.458355
A_21_P001	2.771571	1.470704	2.771571	up	-0.7103	-0.792892	-0.6505	0.726715	0.6505	0.881205	LOC100507	two pore cl	FALSE	Homo sapi	1.01E+08		1.11E+08	chr2	1.11E+08	+	hs|2q13	NR_03762	chr2:11102	NR_03762	Hs.721040
A_33_P340	3.603436	1.849373	3.603436	up	-0.686127	-0.759379	-0.964634	1.487069	0.686127	0.964783			FALSE	Q39AC8_BURS3 (Q39AC8) Cytochr			20841137	chr17	20841078	-	hs|17p11.2		chr17:020841137-020841078		
A_23_P347	2.426645	1.278963	2.426645	up	-0.591151	-0.594561	-0.586298	0.594997	0.883583	0.586299	CTSK	cathepsin K	FALSE	Homo sapi	1513	GO:004545	1.51E+08	chr1	1.51E+08	-	hs|1q21.3	NM_00039	chr1:15076	NM_00039	Hs.632466
A_23_P715	2.447388	1.291243	2.447388	up	-0.634187	-0.585499	-0.604374	0.585499	0.773793	0.690377	TNFRSF11E	tumor necr	FALSE	Homo sapi	4982	GO:004668	1.2E+08	chr8	1.2E+08	-	hs|8q24.12	NM_00254	chr8:11993	NM_00254	Hs.81791
A_32_P477	4.49884	2.169553	4.49884	up	-0.945642	-0.968265	-1.011082	1.411082	0.945642	1.226947	EEF1A1	eukaryotic	FALSE	Homo sapi	1915	GO:000551	74228782	chr6	74228723	-	hs|6q13	NM_00140	chr6:74228	NM_00140	Hs.585192
A_24_P406	2.373632	1.247096	2.373632	up	-0.588283	-0.620241	-0.682509	0.588284	0.639918	0.622053	STEAP1	six transme	FALSE	Homo sapi	26872	GO:000521	89790590	chr7	89790531	+	hs|7q21.13	NM_01244	chr7:89790	NM_01244	Hs.61635
A_23_P264	4.787406	2.259244	4.787406	up	-0.933028	-1.129603	-1.00891	1.390976	0.933028	1.382186	RHBDL1	rhomboid,	FALSE	Homo sapi	9028	GO:000716	726988	chr16	726855	+	hs|16p13.3	NM_00127	chr16:7268	NM_00127	Hs.137572
A_22_P000	2.062509	1.044401	2.062509	up	-0.463118	-0.383645	-0.356196	0.819926	0.356196	0.75412	KRT81	keratin 81,	FALSE	Homo sapi	3887	GO:000551	52679756	chr12	52679697	-	hs|12q13.1	NM_00228	chr12:5267	NM_00228	Hs.658118
A_23_P134	3.931714	1.975159	3.931714	up	-0.799249	-1.146898	-0.962399	1.149989	0.799249	1.067691	EN2	engrailed h	FALSE	Homo sapi	2020	GO:004356	1.55E+08	chr7	1.55E+08	+	hs|7q36.3	NM_00142	chr7:15525	NM_00142	Hs.134989
A_23_P405	5.128848	2.358635	5.128848	up	-1.144917	-1.143894	-0.99093	1.385349	0.99093	1.419884	MGC45922	uncharacte	FALSE	Homo sapi	284365		51321733	chr19	51321674	+	hs|19q13.3	NR_03835	chr19:5132	NR_03835	Hs.161397
A_23_P139	3.335057	1.737711	3.335057	up	-0.767411	-0.985052	-0.966671	0.823037	0.903551	0.767411	IGF1	insulin-like	FALSE	Homo sapi	3479	GO:001046	1.03E+08	chr12	1.03E+08	-	hs|12q23.2	NM_00061	chr12:1027	NM_00061	Hs.160562
A_24_P392	2.22088	1.151131	2.22088	up	-0.537035	-0.594705	-0.600585	0.537035	0.638672	0.545363			FALSE	ribosomal protein S12 pseudogene			90750062	chr15	90750001	+	hs|15q26.1	AK124741	chr15:90750001-9075C		Hs.585046
A_24_P279	3.790272	1.922301	3.790272	up	-1.083847	-0.895864	-0.841876	1.000523	0.841876	1.102919	TRPM2	transient re	FALSE	Homo sapi	7226	GO:000526	45862270	chr21	45862211	+	hs|21q22.3	NM_00330	chr21:4586	NM_00330	Hs.369759
A_33_P335	2.363418	1.240875	2.363418	up	-0.672869	-0.496782	-0.548959	0.496782	0.986415	0.520819	IFITM10	interferon i	FALSE	Homo sapi	402778	GO:000960	1753699	chr11	1753640	-	hs|11p15.5	NM_00117	chr11:1753	NM_00117	Hs.121575
A_21_P000	2.262462	1.177893	2.262462	up	-0.547021	-0.539543	-0.565541	0.599628	0.742404	0.539543	SNHG16	small nucle	FALSE	Homo sapi	1.01E+08		74561418	chr17	74561359	+	hs|17q25.1	NR_03810	chr17:7456	NR_03810	Hs.288215
A_33_P341	6.314879	2.658755	6.314879	up	-1.361619	-1.22773	-1.268974	1.292102	1.598111	1.22773	FAM43A	family with	FALSE	Homo sapi	131583		1.94E+08	chr3	1.94E+08	+	hs|3q29	NM_15369	chr3:19440	NM_15369	Hs.435080
A_19_P008	2.413179	1.270935	2.413179	up	-0.627966	-0.423029	-0.571801	0.690691	0.423029	1.076289	lnc-DYM-1	lnc-DYM-1:	FALSE	LNCipedia lincRNA (lnc-DYM-1), lir			46521362	chr18	46521303	-	hs|18q21.1		chr18:46521362-46521303		
A_24_P186	2.276494	1.186813	2.276494	up	-0.489662	-0.483348	-0.400852	0.840401	0.400852	0.945325	ELN	elastin	FALSE	Homo sapi	2006	GO:000551	73474823	chr7	73474764	+	hs|7q11.23	NM_00050	chr7:73474	NM_00050	Hs.647061
A_24_P504	2.080314	1.056801	2.080314	up	-0.366138	-0.611559	-0.745323	0.611633	0.469613	0.366138			FALSE	ribosomal protein S3a pseudogene			57817089	chr18	57817028	+	hs|18q21.3	BC065737	chr18:57817028-5781		Hs.626174
A_24_P190	2.071558	1.050716	2.071558	up	-0.381441	-0.575293	-0.51692	0.527702	0.769352	0.381441	TMEM97	transmemk	FALSE	Homo sapi	27346	GO:004263	26654983	chr17	26654924	+	hs|17q11.2	NM_01457	chr17:2665	NM_01457	Hs.199695
A_33_P341	3.771444	1.915117	3.771444	up	-0.729483	-1.161096	-0.783372	0.992151	0.729483	1.349767			FALSE				2299301	chr7	2299242	+	hs|7p22.3		chr7:002299242-002299301		
A_33_P339	8.162135	3.028947	8.162135	up	-1.538486	-1.653633	-1.455398	1.467873	1.455398	1.516052	ADAMDEC:	ADAM-like,	FALSE	Homo sapi	27299	GO:000716	24259593	chr8	24259534	+	hs|8p21.2	NM_00114	chr8:24259	NM_00114	Hs.521459
A_33_P321	2.040501	1.028924	2.040501	up	-0.726239	-0.493473	-0.321929	0.616978	0.321929	0.606224	SPOCK2	sparc/oste	FALSE	Homo sapi	9806	GO:001980	73846187	chr10	73846128	-	hs|10q22.1	NM_00113	chr10:7384	NM_00113	Hs.523009
A_32_P193	2.142859	1.099537	2.142859	up	-0.474757	-0.618446	-0.57559	0.528049	0.474757	0.627012	RBMX	RNA bindin	FALSE	Homo sapi	27316	GO:000551	1.36E+08	chrX	1.36E+08	-	hs|Xq26.3	NM_00213	chrX:13595	NM_00213	Hs.380118
A_23_P829	2.248453	1.168933	2.248453	up	-0.555756	-0.453141	-0.588895	0.453141	0.936517	0.519348	NOV	nephroblas	FALSE	Homo sapi	4856	GO:001046	1.2E+08	chr8	1.2E+08	+	hs|8q24.12	NM_00251	chr8:12043	NM_00251	Hs.235935
A_23_P256	2.296206	1.199252	2.296206	up	-0.223983	-0.541369	-0.655807	0.861182	0.223983	1.091433	MSC	musculin	FALSE	Homo sapi	9242	GO:000370	72754906	chr8	72754847	-	hs|8q13.3	NM_00509	chr8:72754	NM_00509	Hs.442619
A_22_P000	3.032637	1.600573	3.032637	up	-1.202736	-0.596613	-0.523518	1.123527	0.523518	0.831808	lnc-MEGF1	lnc-MEGF1	FALSE	LNCipedia lincRNA (lnc-MEGF10-1)			1.27E+08	chr5	1.27E+08	+	hs|5q23.2		chr5:126565170-1265€		Hs.438709
A_23_P472	2.201386	1.138412	2.201386	up	-0.471856	-0.507826	-0.380336	0.78377	0.380336	0.891112	BANF1	barrier to a	FALSE	Homo sapi	8815	GO:000551	65771372	chr11	65771313	+	hs|11q13.1	NM_00386	chr11:6577	NM_00386	Hs.433759

A_33_P339	3.603352	1.84934	3.603352	up	-0.87698	-0.67949	-1.108352	1.080063	0.67949	1.123644			FALSE				1.11E+08	chrX	1.11E+08	-	hs|Xq23		chrX:110861854-110861795		
A_33_P331	2.214801	1.147177	2.214801	up	-0.573855	-0.497428	-0.642791	0.497428	0.730117	0.499913	FAM86B2	family with	FALSE	Homo sapi	653333	GO:000816	12283457	chr8	12283398	-	hs|8p23.1	NM_00113	chr8:12283	NM_00113	Hs.458413
A_33_P332	3.67801	1.878925	3.67801	up	-0.690507	-1.055164	-1.135865	1.198278	0.690507	0.866455	SUSD4	sushi doma	FALSE	sushi doma	55061	GO:003044	2.24E+08	chr1	2.24E+08	-	hs|1q41		chr1:223533605-223533546		
A_33_P330	2.358347	1.237776	2.358347	up	-0.686084	-0.699635	-0.474579	0.611495	0.47458	0.766955			FALSE				55208895	chr17	55208836	-	hs|17q22		chr17:055208895-055208836		
A_22_P000	2.282473	1.190598	2.282473	up	-0.325236	-0.550092	-1.006895	0.486727	0.877607	0.325236	lnc-FKBP2-	lnc-FKBP2-	FALSE	Q2AC61_9BURK (Q2AC61) HrpW,			64015686	chr11	64015627	+	hs|11q13.1		chr11:64015627-64015686		
A_22_P000	2.413081	1.270876	2.413081	up	-0.814628	-0.464065	-0.650892	0.792721	0.626259	0.464065	LOC100131	uncharacte	FALSE	Homo sapi	1E+08		74534031	chr18	74533972	-	hs|18q23	NR_04002	chr18:7453	NR_04002	Hs.385806
A_24_P166	2.101044	1.071107	2.101044	up	-0.397014	-0.524559	-0.421455	0.727194	0.397014	0.746084	REPIN1	replication	FALSE	Homo sapi	29803	GO:000626	1.5E+08	chr7	1.5E+08	+	hs|7q36.1	NM_01437	chr7:15007	NM_01437	Hs.647086
A_22_P000	3.341185	1.74036	3.341185	up	-0.694396	-1.057471	-0.698054	0.857436	0.694396	1.219327	lnc-CTTN-3	lnc-CTTN-3	FALSE	AGENCOURT_25900990 NIH_MGC			70493505	chr11	70493446	+	hs|11q13.4	CO247556	chr11:70493446-70493		Hs.672096
A_33_P326	2.053039	1.037761	2.053039	up	-0.345521	-0.743841	-0.452973	0.625411	0.345521	0.600015	LRRC26	leucine rich	FALSE	Homo sapi	389816	GO:000551	1.4E+08	chr9	1.4E+08	-	hs|9q34.3	NM_00101	chr9:14006	NM_00101	Hs.669977
A_21_P000	2.012019	1.008644	2.012019	up	-0.774995	-0.467453	-0.420483	0.458989	0.420483	0.483528	lnc-TAF12-	lnc-TAF12-	FALSE	LNCipedia lincRNA (lnc-TAF12-3), l			29198755	chr1	29197459	-	hs|1p35.3		chr1:29198755-29197		Hs.601502
A_33_P324	3.175732	1.667089	3.175732	up	-0.596937	-0.9932	-0.704587	1.117161	0.596936	0.992446	CCDC78	coiled-coil	FALSE	Homo sapi	124093	GO:004238	772645	chr16	772586	-	hs|16p13.3	NM_00103	chr16:7726	NM_00103	Hs.381943
A_23_P894	3.756775	1.909495	3.756775	up	-0.951735	-0.925693	-0.928469	0.925693	1.042386	0.954509	CCL2	chemokine	FALSE	Homo sapi	6347	GO:000940	32584109	chr17	32584050	+	hs|17q12	NM_00298	chr17:3258	NM_00298	Hs.303649
A_33_P328	2.532781	1.340722	2.532781	up	-0.95684	-0.775186	-0.286485	0.608891	0.286485	1.10828	PHF21B	PHD finger	FALSE	Homo sapi	112885	GO:000827	45279025	chr22	45278966	-	hs|22q13.3	NM_13841	chr22:4527	NM_13841	Hs.254097
A_22_P000	3.410058	1.769796	3.410058	up	-0.965843	-0.756887	-0.66434	1.159685	0.66434	1.098294	LOC101928	uncharacte	FALSE		1.02E+08		76336745	chr11	76336686	-	hs|11q13.5		chr11:76336745-76336686		
A_23_P121	2.24116	1.164245	2.24116	up	-0.418271	-0.837314	-0.523008	0.526949	0.768923	0.418271	SEPP1	selenoprot	FALSE	Homo sapi	6414	GO:001995	42799939	chr5	42799904	-	hs|5p12	NM_00541	chr5:42799	NM_00541	Hs.275775
A_22_P000	3.163972	1.661737	3.163972	up	-0.852549	-0.882593	-0.725621	0.886435	0.72562	0.912393	lnc-C17orf6	lnc-C17orf6	FALSE	293T_111 SVA 5' RACE from 293T			80416352	chr17	80416293	-	hs|17q25.3	GR564636	chr17:80416352-80416		Hs.723123
A_33_P371	3.422325	1.774977	3.422325	up	-0.872133	-0.843434	-0.763549	1.019041	0.763549	1.063223	CXCL12	chemokine	FALSE	Homo sapi	6387	GO:000834	44880453	chr10	44880394	-	hs|10q11.2	NM_00103	chr10:4488	NM_00103	Hs.522891
A_23_P414	2.30029	1.201816	2.30029	up	-0.591874	-0.500969	-0.621784	0.500969	0.771179	0.618671	SLC39A8	solute carri	FALSE	Homo sapi	64116	GO:000588	1.03E+08	chr4	1.03E+08	-	hs|4q24	NM_02215	chr4:10318	NM_02215	Hs.288034
A_22_P000	2.060211	1.042792	2.060211	up	-0.686432	-0.535718	-0.414435	0.521255	0.414435	0.556102			FALSE				74274697	chr8	74274638	+	hs|8q21.11	DB201663	chr8:74274638-74274		Hs.586405
A_24_P258	3.115445	1.639438	3.115445	up	-0.538973	-0.810529	-0.827642	1.228639	0.538973	0.973558	OR5L2	olfactory re	FALSE	Homo sapi	26338	GO:005091	55595252	chr11	55595193	+	hs|11q11	NM_00100	chr11:5559	NM_00100	Hs.528356
A_33_P341	2.453772	1.295001	2.453772	up	-0.668881	-0.611985	-0.569459	0.569458	0.845065	0.620155	ATP8	ATP syntha	FALSE	mitochond	4509		8508	chrM	8449	+		AK128101	chrM:8449-8508		
A_23_P143	2.119074	1.083434	2.119074	up	-0.530551	-0.491883	-0.473267	0.483138	0.798197	0.473267	TIPARP	TCDD-indu	FALSE	Homo sapi	25976	GO:003009	1.56E+08	chr3	1.56E+08	+	hs|3q25.31	NM_01550	chr3:15642	NM_01550	Hs.744050
A_24_P354	4.156102	2.055231	4.156102	up	-0.978254	-1.027575	-1.059984	0.984902	1.136725	0.978254	SPOCK1	sparc/oste	FALSE	Homo sapi	6695	GO:003159	1.36E+08	chr5	1.36E+08	-	hs|5q31.2	NM_00459	chr5:13631	NM_00459	Hs.596136
A_22_P000	5.439205	2.443396	5.439205	up	-1.35468	-1.562508	-0.906212	1.22907	0.906212	1.371506	lnc-HDDC3	lnc-HDDC3	FALSE	Q6AGI4_LEIXX (Q6AGI4) Compete			91478274	chr15	91478215	-	hs|15q26.1		chr15:91478274-91478215		
A_33_P326	6.093658	2.607308	6.093658	up	-0.974232	-1.007584	-1.116621	1.771294	0.974232	1.977962			FALSE				47053112	chr21	47053053	-	hs|21q22.3		chr21:047053112-047053053		
A_23_P554	2.057838	1.041129	2.057838	up	-0.162219	-0.374707	-1.209527	0.711895	0.162219	0.50282	CBX8	chromobox	FALSE	Homo sapi	57332	GO:000079	77768401	chr17	77768342	-	hs|17q25.3	NM_02064	chr17:7776	NM_02064	Hs.387258
A_23_P121	2.146458	1.101958	2.146458	up	-0.529699	-0.523667	-0.528217	0.542901	0.65772	0.523668	PTX3	pentraxin 3	FALSE	Homo sapi	5806	GO:005076	1.57E+08	chr3	1.57E+08	+	hs|3q25.32	NM_00285	chr3:15716	NM_00285	Hs.591286
A_33_P332	4.426268	2.146091	4.426268	up	-1.074061	-0.852686	-1.058448	1.280787	0.852686	1.319605	PMF1-BGL	PMF1-BGL	FALSE	Homo sapi	1.01E+08		1.56E+08	chr1	1.56E+08	+	hs|1q22	NM_00119	chr1:15621	NM_00119	Hs.530479
A_33_P334	3.569358	1.835665	3.569358	up	-0.787149	-1.021787	-0.726101	1.072528	0.726101	1.173327	CASKIN1	CASK intera	FALSE	Homo sapi	57524	GO:000551	2228170	chr16	2228111	-	hs|16p13.3	NM_02076	chr16:2228	NM_02076	Hs.643537
A_22_P000	5.315478	2.410199	5.315478	up	-0.839434	-1.759757	-1.341052	1.295137	0.839435	1.155783	lnc-MME-4	lnc-MME-4	FALSE	BX094640 Soares_NFL_T_GBC_S1			1.55E+08	chr3	1.55E+08	+	hs|3q25.31	BX094640	chr3:155204837-1552		Hs.128933
A_23_P151	5.38887	2.429983	5.38887	up	-1.188297	-1.146539	-1.167868	1.146539	1.463434	1.177271	MT1F	metallothi	FALSE	Homo sapi	4494	GO:007129	56693136	chr16	56693077	+	hs|16q12.2	NM_00594	chr16:5669	NM_00594	Hs.513626
A_33_P323	3.100502	1.632502	3.100502	up	-0.803197	-0.811576	-0.811155	0.835043	0.803197	0.833338	MT1X	metallothi	FALSE	Homo sapi	4501	GO:007129	56718021	chr16	56717962	+	hs|16q13	NM_00595	chr16:5671	NM_00595	Hs.374950
A_21_P001	2.383632	1.253162	2.383632	up	-0.612943	-0.634344	-0.586273	0.659163	0.586273	0.68049	FAM86C1	family with	FALSE	Homo sapi	55199	GO:000816	71502797	chr11	71500845	+	hs|11q13.4	NM_00109	chr11:7150	NM_00109	Hs.567527
A_21_P001	27.27218	4.769358	27.27218	up	-2.031363	-2.125929	-2.287266	2.855394	2.031363	2.976759	lnc-TPTE-3	lnc-TPTE-3	FALSE	LNCipedia lincRNA (lnc-TPTE-3), lir			9827566	chr21	9827507	-	hs|21p11.2	HQ013231	chr21:9827566-98275		Hs.426704
A_22_P000	4.724132	2.240049	4.724132	up	-1.255268	-1.134138	-0.731661	1.700747	0.731661	1.166673	MIR940	microRNA	FALSE	microRNA	1E+08		2318748	chr16	2318689	+	hs|16p13.3	BM766675	chr16:2318689-23187		Hs.729989
A_32_P194	2.530585	1.339471	2.530585	up	-0.84887	-0.525623	-0.641951	0.525623	0.923247	0.553099			FALSE	600944622T1 NIH_MGC_17 Homo			49993745	chr19	49993510	-	hs|19q13.3	BE300169	chr19:49993745-4999		Hs.744351
A_24_P941	2.334471	1.223096	2.334471	up	-0.870354	-0.487637	-0.542929	0.487637	0.763774	0.516957	FAM65B	family with	FALSE	Homo sapi	9750	GO:000573	24804801	chr6	24804742	-	hs|6p22.3	NM_01472	chr6:24804	NM_01472	Hs.559459
A_23_P207	2.189928	1.130884	2.189928	up	-0.692134	-0.490011	-0.387091	0.681057	0.387091	0.755267	PLCD3	phospholip	FALSE	Homo sapi	113026	GO:000588	43189341	chr17	43189282	-	hs|17q21.3	NM_13337	chr17:4318	NM_13337	Hs.380094
A_33_P333	3.209969	1.682559	3.209969	up	-0.748113	-0.650364	-0.555583	1.217059	0.555583	1.320976	CRTC1	CREB regul	FALSE	Homo sapi	23373	GO:000551	18888290	chr19	18888231	+	hs|19p13.1	NM_00109	chr19:1888	NM_00109	Hs.371096
A_23_P162	2.695805	1.430716	2.695805	up	-0.753596	-0.641078	-0.693029	0.641078	0.921267	0.6421	VDR	vitamin D (	FALSE	Homo sapi	7421	GO:006055	48235561	chr12	48235502	-	hs|12q13.1	NM_00101	chr12:4823	NM_00101	Hs.524368
A_22_P000	5.91773	2.565044	5.91773	up	-1.038182	-1.259899	-1.112226	1.557601	1.038182	1.689044			FALSE	AGENCOURT_13642683 NIH_MGC			4710182	chr17	4710123	-	hs|17p13.2	CB990811	chr17:4710182-471012		Hs.579211
A_21_P000	7.329586	2.873732	7.329586	up	-1.323559	-1.544167	-1.257213	1.728446	1.257213	1.510595	LINC01018	long interg	FALSE	Homo sapi	255167		6583914	chr5	6583858	+	hs|5p15.31	NR_02442	chr5:65838	NR_02442	Hs.435515
A_33_P332	3.064394	1.615602	3.064394	up	-0.74439	-0.736586	-0.949033	0.899393	0.736586	0.780818	HES6	hes family	FALSE	Homo sapi	55502	GO:000371	2.39E+08	chr2	2.39E+08	-	hs|2q37.3	NM_01864	chr2:23914	NM_01864	Hs.42949
A_33_P337	2.684328	1.424561	2.684328	up	-0.609212	-0.749018	-0.660496	0.633551	1.012193	0.609212	NAMPT	nicotinami	FALSE	Homo sapi	10135	GO:000551	1.06E+08	chr7	1.06E+08	-	hs|7q22.3	NM_00574	chr7:10588	NM_00574	Hs.489615
A_33_P330	4.890901	2.2901	4.890901	up	-0.948896	-1.422561	-1.006423	1.31726	0.948895	1.226266	MON1B	MON1 secr	FALSE	Homo sapi	22879	GO:000551	77229557	chr16	77229498	+	hs|16q23.1	NM_01494	chr16:7722	NM_01494	Hs.513743
A_33_P336	2.083115	1.058743	2.083115	up	-0.637102	-0.372706	-0.509018	0.58921	0.372706	0.695486	LOC100129	uncharacte	FALSE	Homo sapi	1E+08		1.46E+08	chr8	1.46E+08	-	hs|8q24.3	AK130913	chr8:146045756-14604		Hs.639990
A_24_P100	3.31426	1.728687	3.31426	up	-0.722801	-0.582898	-0.649549	1.280651	0.582898	1.367264	CYB5R3	cytochrom	FALSE	Homo sapi	1727	GO:000581	43026905	chr22	43024246	-	hs|22q13.2	NM_00732	chr22:4302	NM_00732	Hs.561064
A_23_P113	2.170539	1.118053	2.170539	up	-0.368888	-0.773574	-0.45173	0.456942	0.934136	0.368888	APLN	apelin	FALSE	Homo sapi	8862	GO:000716	1.29E+08	chrX	1.29E+08	-	hs|Xq26.1	NM_01741	chrX:12877	NM_01741	Hs.303084
A_24_P237	3.595734	1.846286	3.595734	up	-0.629182	-1.159436	-0.923816	1.206545	0.629182	0.990696	MAN1C1	mannosida	FALSE	Homo sapi	57134	GO:003017	26098263	chr1	26098204	+	hs|1p36.11	NM_02037	chr1:26098	NM_02037	Hs.197043
A_33_P334	2.89039	1.531264	2.89039	up	-0.60329	-0.580197	-0.614474	1.107524	0.580197	1.10811	C3	complemer	FALSE	Homo sapi	718	GO:000551	6686195	chr19	6686136	-	hs|19p13.3	NM_00006	chr19:6686	NM_00006	Hs.529053
A_22_P000	2.029465	1.021099	2.029465	up	-0.044791	-0.121776	-0.574253	1.075456	0.044791	1.202229	SGMS1-AS	SGMS1 ant	FALSE	Homo sapi	1.04E+08		52391111	chr10	52391052	+	hs|10q11.23		chr10:5239	NR_12641	Hs.450493
A_23_P509	2.145438	1.101272	2.145438	up	-0.532747	-0.524064	-0.566056	0.540046	0.524064	0.616839	SERPINE2	serpin pept	FALSE	Homo sapi	5270	GO:000551	2.25E+08	chr2	2.25E+08	-	hs|2q36.1	NM_00621	chr2:22484	NM_00621	Hs.38449
A_21_P000	5.856454	2.550027	5.856454	up	-1.107935	-1.209487	-1.467012	1.409368	1.107935	1.348346			FALSE				53626998	chr7	53626939	-	hs|7p12.1		chr7:53626998-53626939		
A_22_P000	2.299825	1.201524	2.299825	up	-0.840782	-0.395009	-0.901005	0.395009	0.558032	0.514735	LINC00092	long interg	FALSE	long interg	1E+08		98790053	chr9	98789994	-	hs|9q22.32	DA079394	chr9:98790053-98789		Hs.738584
A_23_P620	4.055614	2.01992	4.055614	up	-0.98763	-0.971092	-0.990897	0.971092	1.149374	0.989677	SCG5	secretogra	FALSE	Homo sapi	6447	GO:000551	32989165	chr15	32989106	+	hs|15q13.3	NM_00302	chr15:3298	NM_00302	Hs.156540
A_19_P008	2.57789	1.366191	2.57789	up	-0.511882	-0.675776	-0.407228	0.961153	0.407228	1.135306	TLE1	transducin-	FALSE	Homo sapi	7088	GO:000551	84267161	chr9	84249190	-	hs|9q21.32	NM_00130	chr9:84267	NM_00130	Hs.197320
A_23_P135	2.333136	1.22227	2.333136	up	-0.548686	-0.721899	-0.625913	0.548686	0.617834	0.603792	DPYD	dihydropyr	FALSE	Homo sapi	1806	GO:005066	97543959	chr1	97543900	-	hs|1p21.3	NM_00011	chr1:97543	NM_00011	Hs.335034
A_24_P278	2.098942	1.069663	2.098942	up	-0.496805	-0.464589	-0.453928	0.595716	0.744021	0.453928	ASB13	ankyrin rep	FALSE	Homo sapi	79754	GO:003555	5681088	chr10	5681029	-	hs|10p15.1	NM_02470	chr10:5681	NM_02470	Hs.445899

A_21_P000	3.403393	1.766974	3.403393	up	-0.686287	-1.042577	-0.695456	0.986083	0.686287	1.204231	lnc-BTRC-2	lnc-BTRC-2	FALSE	LNCipedia lincRNA (lnc-BTRC-2), lin			1.03E+08	chr10	1.03E+08	+	hs|10q24.32		chr10:103044967-103045026		
A_22_P000	2.677084	1.420662	2.677084	up	-0.496527	-0.631696	-1.278418	0.496527	0.795531	0.563289	ESRG	embryonic	FALSE	Homo sapi	790952	GO:000563	54666226	chr3	54666167	-	hs|3p14.3	NR_027122	chr3:54666	NR_027122	Hs.720658
A_22_P000	3.15989	1.659875	3.15989	up	-0.828374	-0.88079	-0.610126	0.972107	0.610126	1.078101	lnc-HSP90A	lnc-HSP90A	FALSE	LNCipedia lincRNA (lnc-HSP90AA1-			1.03E+08	chr14	1.03E+08	-	hs|14q32.31		chr14:102606311-102606252		
A_33_P330	3.570764	1.836233	3.570764	up	-0.801426	-1.057978	-0.916486	0.941341	0.801426	0.990042	VIT	vitrin	FALSE	Homo sapi	5212	GO:003019	37041576	chr2	37041517	+	hs|2p22.2	NM_05327	chr2:37041	NM_05327	Hs.137415
A_23_P551	3.211043	1.683042	3.211043	up	-0.745303	-0.587709	-0.623845	1.234946	0.587709	1.269614	G6PC3	glucose 6 p	FALSE	Homo sapi	92579	GO:000597	42152071	chr17	42151599	+	hs|17q21.3	NM_13838	chr17:4215	NM_13838	Hs.294005
A_23_P936	2.359627	1.238559	2.359627	up	-0.764676	-0.780888	-0.455459	0.455459	0.643506	0.615689	AKR1B10	aldo-keto r	FALSE	Homo sapi	57016	GO:000551	1.34E+08	chr7	1.34E+08	+	hs|7q33	NM_02029	chr7:13422	NM_02029	Hs.116724
A_23_P203	3.220784	1.687412	3.220784	up	-0.938264	-0.778234	-0.923202	0.83668	0.807621	0.778234	APOC3	apolipopro	FALSE	Homo sapi	345	GO:005099	1.17E+08	chr11	1.17E+08	+	hs|11q23.3	NM_00004	chr11:1167	NM_00004	Hs.73849
A_22_P000	7.070648	2.821842	7.070648	up	-0.795973	-1.558948	-1.367976	1.831981	0.795973	2.114677	lnc-RP11-4	lnc-RP11-4	FALSE	LNCipedia lincRNA (lnc-RP11-422N			1.11E+08	chr8	1.11E+08	+	hs|8q23.2		chr8:110986951-110987010		
A_23_P202	5.54213	2.47044	5.54213	up	-1.228666	-1.209038	-1.220192	1.244228	1.300159	1.209038	CXCL12	chemokine	FALSE	Homo sapi	6387	GO:000834	44872947	chr10	44872888	-	hs|10q11.2	NM_19916	chr10:4487	NM_19916	Hs.522891
A_32_P377	2.695282	1.430436	2.695282	up	-0.627045	-0.740163	-0.727998	0.824394	0.744664	0.627045	GDNF	glial cell de	FALSE	Homo sapi	2668	GO:000165	37812991	chr5	37812932	-	hs|5p13.2	NM_00119	chr5:37812	NM_00119	Hs.248114
A_21_P001	2.037844	1.027044	2.037844	up	-0.358063	-0.401145	-0.544835	0.558247	0.860778	0.358063			FALSE				1.03E+08	chr4	1.03E+08	-	hs|4q24		chr4:103339819-10333		Hs.731809
A_22_P000	6.17522	2.626491	6.17522	up	-1.080119	-1.415761	-1.162279	1.590684	1.080119	1.55051	LOC10050	uncharacte	FALSE	Homo sapi	1.01E+08		8302049	chr7	8301990	+	hs|7p21.3	NR_12574	chr7:83019	NR_12574	Hs.729535
A_24_P122	6.327756	2.661694	6.327756	up	-1.591039	-1.25804	-1.248751	1.248751	1.323617	1.314885	LIF	leukemia ir	FALSE	Homo sapi	3976	GO:006042	30636772	chr22	30636713	-	hs|22q12.2	NM_00230	chr22:3063	NM_00230	Hs.2250
A_33_P330	2.619727	1.389417	2.619727	up	-0.772401	-0.81077	-0.565005	0.590088	0.565005	0.864981	KLRG2	killer cell le	FALSE	Homo sapi	346689	GO:003024	1.39E+08	chr7	1.39E+08	-	hs|7q34	NM_19850	chr7:13913	NM_19850	Hs.17572
A_32_P219	2.469424	1.304174	2.469424	up	-0.558411	-0.658306	-0.793407	0.71644	0.627548	0.558411	WTAP	Wilms tum	FALSE	Homo sapi	9589	GO:000838	1.6E+08	chr6	1.6E+08	+	hs|6q25.3	NM_15285	chr6:16016	NM_15285	Hs.446091
A_23_P353	3.912018	1.967913	3.912018	up	-1.209727	-0.992348	-0.845061	0.845061	1.119671	0.891871	TAF5L	TAF5-like R	FALSE	Homo sapi	27097	GO:000635	2.3E+08	chr1	2.3E+08	-	hs|1q42.13	NM_01440	chr1:22972	NM_01440	Hs.270621
A_22_P000	5.488331	2.456368	5.488331	up	-1.031784	-1.090499	-1.101863	1.666635	1.031784	1.446538	lnc-FKBP3-	lnc-FKBP3-	FALSE	LNCipedia lincRNA (lnc-FKBP3-3), l			45431501	chr14	45431442	-	hs|14q21.2		chr14:45431501-45431442		
A_33_P331	10.16969	3.346203	10.16969	up	-1.782285	-1.533932	-1.572258	1.753539	1.862666	1.533932	RNF222	ring finger	FALSE	Homo sapi	643904	GO:000827	8296576	chr17	8296517	-	hs|17p13.1	NM_00114	chr17:8296	NM_00114	Hs.526550
A_33_P323	2.479279	1.30992	2.479279	up	-0.961225	-0.655174	-0.451165	0.531954	0.451165	0.879078	NMNAT1	nicotinamic	FALSE	Homo sapi	64802	GO:000551	10041371	chr1	10041227	+	hs|1p36.22	NM_00129	chr1:10041	NM_00129	Hs.666730
A_33_P328	8.083746	3.015024	8.083746	up	-1.244702	-1.794076	-1.36919	1.712891	1.244701	1.679512	lnc-C16orf4	lnc-C16orf4	FALSE	Homo sapiens cDNA FLJ45052 fis,			1358800	chr16	1358741	-	hs|16p13.3	AK126997	chr16:1358800-1358741		
A_21_P000	4.939458	2.304353	4.939458	up	-1.046649	-1.032419	-0.989798	1.285293	1.569101	0.989798	lnc-ZNF366	lnc-ZNF366	FALSE	LNCipedia lincRNA (lnc-ZNF366-2),			71870167	chr5	71817327	-	hs|5q13.2		chr5:71870167-71817327		
A_33_P327	2.047897	1.034143	2.047897	up	-0.453954	-0.327342	-0.852556	0.384853	0.756382	0.327342			FALSE				1139814	chr4	1139755	+	hs|4p16.3		chr4:001139755-001139814		
A_33_P354	3.049932	1.608777	3.049932	up	-0.69927	-0.837573	-0.660018	0.9298	0.660019	1.039651	TUSC8	tumor supp	FALSE	AGENCOUF	400128		44978922	chr13	44978863	-	hs|13q14.1	BQ950045	chr13:44978922-44978		Hs.438480
A_21_P000	3.202748	1.67931	3.202748	up	-0.815835	-0.879431	-0.637023	1.044919	0.637023	1.023702	lnc-AC0074	lnc-AC0074	FALSE	LNCipedia lincRNA (lnc-AC007405.			1.72E+08	chr2	1.72E+08	+	hs|2q31.1		chr2:171626804-171626863		
A_33_P326	3.090595	1.627884	3.090595	up	-0.709613	-1.041963	-0.816264	0.709613	0.738652	0.867549			FALSE	Homo sapiens cDNA FLJ26160 fis,			1.32E+08	chr5	1.32E+08	-	hs|5q31.1	AK129671	chr5:132279131-132279072		
A_33_P333	2.697994	1.431887	2.697994	up	-0.659029	-0.621378	-0.684176	0.681424	1.028276	0.621378	ACACB	acetyl-CoA	FALSE	Homo sapi	32	GO:000551	1.1E+08	chr12	1.1E+08	+	hs|12q24.1	NM_00109	chr12:1097	NM_00109	Hs.234898
A_23_P333	3.458358	1.790087	3.458358	up	-0.773605	-1.37292	-0.809483	0.811735	0.828915	0.773605	EEPD1	endonuclea	FALSE	Homo sapi	80820	GO:000628	36340917	chr7	36340858	+	hs|7p14.2	NM_03063	chr7:3634	NM_03063	Hs.487994
A_33_P325	2.088774	1.062656	2.088774	up	-0.548502	-0.487147	-0.504774	0.487147	0.626112	0.534287	RPS23	ribosomal	FALSE	Homo sapi	6228	GO:000551	81569349	chr5	81569290	-	hs|5q14.2	NM_00102	chr5:81569	NM_00102	Hs.527193
A_33_P331	3.403903	1.76719	3.403903	up	-1.087771	-0.833818	-0.665493	0.783947	1.265047	0.665493			FALSE				1.36E+08	chr7	1.36E+08	+	hs|7q33		chr7:135614637-135614696		
A_33_P326	2.141799	1.098823	2.141799	up	-0.365763	-0.534244	-0.348554	0.81869	0.348554	0.880664	LOC10013	uncharacte	FALSE	Homo sapi	1E+08		1.33E+08	chr12	1.33E+08	+	hs|12q24.3	NR_02456	chr12:1328	NR_02456	Hs.536338
A_23_P358	2.420519	1.275316	2.420519	up	-0.606906	-0.6757	-0.699581	0.629677	0.606906	0.607179	AHRR	aryl-hydroc	FALSE	Homo sapi	57491	GO:000119	438089	chr5	438030	+	hs|5p15.33	NM_02073	chr5:43803	NM_02073	Hs.50823
A_22_P000	4.916952	2.297764	4.916952	up	-1.098625	-1.146921	-0.992587	1.235433	0.992587	1.42714			FALSE	PREDICTED: Homo sapiens unchara			1210679	chr1	1210620	+	hs|1p36.33	XR_241053	chr1:12106	XR_241053	
A_32_P194	2.393556	1.259155	2.393556	up	-0.589524	-0.601418	-0.579308	0.579308	0.840795	0.587114	RPL21	ribosomal	FALSE	Homo sapi	6144	GO:001046	29188408	chr10	29188349	-	hs|10p12.1	NM_00098	chr10:0291	NM_00098	Hs.381123
A_19_P003	2.780129	1.475152	2.780129	up	-0.729866	-0.71159	-0.626972	0.871808	0.626972	0.858248	LINC00861	long interg	FALSE	long interg	1E+08		1.27E+08	chr8	1.27E+08	-	hs|8q24.13		chr8:126963492-126963433		
A_23_P353	3.572791	1.837052	3.572791	up	-0.940155	-0.872064	-0.914273	0.872064	0.996845	0.915753	SVIL	supervillin	FALSE	Homo sapi	6840	GO:000551	29747064	chr10	29747005	-	hs|10p11.2	NM_02173	chr10:2974	NM_02173	Hs.499209
A_33_P324	2.777017	1.473536	2.777017	up	-0.652535	-0.639953	-0.781469	0.639953	1.025045	0.681654	RGL1	ral guanine	FALSE	Homo sapi	23179	GO:000832	1.84E+08	chr1	1.84E+08	+	hs|1q25.3	NM_01514	chr1:18389	NM_01514	Hs.497148
A_33_P338	3.605938	1.850375	3.605938	up	-0.726772	-0.773865	-0.815599	1.362963	0.726772	1.145153	YJEFN3	YjeF N-tern	FALSE	Homo sapi	374887		19648393	chr19	19648334	+	hs|19p13.1	NM_19853	chr19:1964	NM_19853	Hs.729508
A_33_P332	2.86907	1.520583	2.86907	up	-0.769954	-0.850675	-0.682963	0.748605	0.682963	0.826589	TPP1	tripeptidyl	FALSE	Homo sapi	1200	GO:000662	6636189	chr11	6636130	-	hs|11p15.4	NM_00039	chr11:6636	NM_00039	Hs.523454
A_24_P315	6.456416	2.690733	6.456416	up	-1.173515	-1.347777	-1.027945	1.755278	1.027945	1.739741	NFYC-AS1	NFYC antis	FALSE	Homo sapi	1E+08		41157533	chr1	41157474	-	hs|1p34.2	NR_02456	chr1:41157	NR_02456	Hs.233458
A_22_P000	4.156423	2.055342	4.156423	up	-1.018479	-0.883632	-0.88652	1.209499	0.883632	1.284276	lnc-SPATC1	lnc-SPATC1	FALSE	PREDICTED: Homo sapiens sphingo			1.45E+08	chr8	1.45E+08	+	hs|8q24.3	XM_00171	chr8:1451	XM_00171	Hs.711115
A_33_P333	6.816604	2.769053	6.816604	up	-1.57104	-1.305568	-1.213847	1.580532	1.213847	1.422326			FALSE												
A_24_P406	2.093843	1.066153	2.093843	up	-0.561893	-0.372679	-0.395361	0.73098	0.372679	0.764868	MAPK13	mitogen-ac	FALSE	Homo sapi	5603	GO:003275	36107374	chr6	36107315	+	hs|6p21.31	NM_00275	chr6:36107	NM_00275	Hs.178695
A_33_P337	2.069807	1.049496	2.069807	up	-0.352507	-0.317719	-0.29633	0.944827	0.29633	0.940776	SPTAN1	spectrin, al	FALSE	Homo sapi	6709	GO:000551	1.31E+08	chr9	1.31E+08	+	hs|9q34.11	NM_00113	chr9:13138	NM_00113	Hs.372331
A_22_P000	4.064146	2.022952	4.064146	up	-1.350205	-1.23564	-0.796217	0.948467	0.796217	0.942112	lnc-PCM1-4	lnc-PCM1-4	FALSE	602688802F1 NIH_MGC_95 Homo			17659251	chr8	17659192	+	hs|8p22		chr8:17659192-176592		Hs.637621
A_33_P324	2.011552	1.008309	2.011552	up	-0.693755	-0.541995	-0.28528	0.491878	0.28528	0.726739	PRKAG3	protein kin	FALSE	Homo sapi	53632	GO:000828	2.2E+08	chr2	2.2E+08	-	hs|2q35	NM_01743	chr2:21968	NM_01743	Hs.591634
A_33_P338	4.038776	2.013918	4.038776	up	-0.929445	-0.889359	-0.899897	0.927599	1.506096	0.889359	RN7SK	RNA, 7SK s	FALSE	Homo sapi	125050		52860718	chr6	52860659	+	hs|6p12.2	NR_00144	chr6:52860	NR_00144	Hs.224866
A_33_P335	2.261798	1.17747	2.261798	up	-0.659108	-0.385991	-0.246035	0.983132	0.246035	1.012108	CSF1	colony stim	FALSE	Homo sapi	1435	GO:004847	1.1E+08	chr1	1.1E+08	+	hs|1p13.3	NM_17221	chr1:11046	NM_17221	Hs.173894
A_33_P328	2.347832	1.231329	2.347832	up	-0.747772	-0.546675	-0.524269	0.524269	0.5693	0.7817			FALSE				1.61E+08	chr1	1.61E+08	-	hs|1q23.3		chr1:161209136-161209077		
A_33_P341	2.749426	1.459131	2.749426	up	-0.568617	-0.737789	-0.623123	0.889318	0.568617	0.989927	CD72	CD72 mole	FALSE	CD72 mole	971	GO:000551	35616995	chr9	35616936	-	hs|9p13.3	AK309268	chr9:35616995-356169		Hs.116481
A_33_P303	2.563537	1.358136	2.563537	up	-0.565782	-0.63307	-0.484175	1.032278	0.484175	0.874927	LCE1C	late cornifi	FALSE	Homo sapi	353133	GO:003142	1.53E+08	chr1	1.53E+08	+	hs|1q21.3	NM_17835	chr1:15277	NM_17835	Hs.516429
A_22_P000	2.771947	1.4709	2.771947	up	-0.813095	-0.634414	-0.979318	0.634414	0.716368	0.635091	LOC10272	uncharacte	FALSE		1.03E+08		2.02E+08	chr2	2.02E+08	+	hs|2q33.1		chr2:201967678-201967737		
A_33_P341	3.131146	1.646691	3.131146	up	-0.952876	-1.052136	-0.562035	0.779083	0.562036	1.031906	POR	P450 (cyto	FALSE	P450 (cyto	5447	GO:005066	75544843	chr7	75544784	+	hs|7q11.23		chr7:75544784-75544843		
A_33_P326	2.025196	1.018062	2.025196	up	-0.782736	-0.327852	-0.719549	0.545166	0.327852	0.35103	LOC40086	uncharacte	FALSE	Homo sapi	400863		35335486	chr21	35335427	+	hs|21q22.11		chr21:3533	NM_00128	Hs.729814
A_23_P369	3.086371	1.625912	3.086371	up	-0.766498	-0.715187	-1.000284	0.715187	0.91529	0.765289			FALSE	Homo sapiens HSPC254 mRNA, pa			6917043	chr17	6916080	-	hs|17p13.1	AF161372	chr17:6917043-6916080		
A_33_P342	2.347216	1.230951	2.347216	up	-0.648439	-0.67631	-0.576496	0.576496	0.601073	0.614038	TMEM200C	transmeml	FALSE	transmeml	645369	GO:001602	5895116	chr18	5895057	-	hs|18p11.3	XM_00525	chr18:5895	XM_00525	Hs.650086
A_33_P382	2.280256	1.189196	2.280256	up	-0.274076	-0.859731	-0.49748	0.858287	0.274076	0.803936	RABL6	RAB, meml	FALSE	Homo sapi	55684	GO:000551	1.4E+08	chr9	1.4E+08	+	hs|9q34.3	NM_00117	chr9:13972	NM_00117	Hs.370555
A_23_P402	2.501061	1.322541	2.501061	up	-0.490334	-0.88176	-0.840729	0.697477	0.566988	0.490334	DOK5	docking pro	FALSE	Homo sapi	55816	GO:000016	53267262	chr20	53267203	+	hs|20q13.2	NM_01843	chr20:5326	NM_01843	Hs.656582
A_22_P000	4.946209	2.306323	4.946209	up	-0.964812	-1.135485	-0.894361	1.49223	0.894361	1.537722	CDC42SE1	CDC42 sma	FALSE	CDC42 sma	56882	GO:000690	1.51E+08	chr1	1.51E+08	-	hs|1q21.3	DA776403	chr1:151032154-15103		Hs.628492

A_33_P331	3.043433	1.6057	3.043433	up	-0.681154	-0.828731	-0.876595	1.039073	0.681154	0.710392	HAPLN2	hyaluronar	FALSE	Homo sapi	60484	GO:008502	1.57E+08	chr1	1.57E+08	+	hs|1q23.1	NM_02181	chr1:15659	NM_02181	Hs.410719
A_22_P000	5.238681	2.389204	5.238681	up	-1.406485	-1.079821	-1.065807	1.065807	1.438913	1.110777	lnc-CCDC90	lnc-CCDC90	FALSE	RST25001 Athersys RAGE Library H			82924391	chr11	82924332	-	hs|11q14.1	BG205572	chr11:82924391-82924		Hs.615971
A_23_P109	2.456165	1.296408	2.456165	up	-0.54705	-0.468818	-0.600873	0.865193	0.468818	0.938472	PLBD2	phospholip	FALSE	Homo sapi	196463	GO:001604	1.14E+08	chr12	1.14E+08	+	hs|12q24.1	NM_17354	chr12:1138	NM_17354	Hs.115896
A_33_P322	2.268492	1.181734	2.268492	up	-0.433951	-0.515351	-0.451459	0.80019	0.433951	0.910297	ARAFP2	ARAF pseu	FALSE	Homo sapi	644000		62865027	chr7	62864968	-	hs|7q11.21	BC033982	chr7:62865027-62864968		
A_33_P325	3.592948	1.845168	3.592948	up	-1.017964	-0.703429	-1.266048	0.71091	1.133724	0.703429	CLDN11	claudin 11	FALSE	Homo sapi	5010	GO:001633	1.7E+08	chr3	1.7E+08	+	hs|3q26.2	NM_00560	chr3:17015	NM_00560	Hs.31595
A_33_P338	4.560047	2.189049	4.560047	up	-1.005493	-1.039722	-1.046076	1.230345	1.005493	1.240017	EEF1A1	eukaryotic	FALSE	Homo sapi	1915	GO:000551	74229639	chr6	74229214	-	hs|6q13	NM_00140	chr6:74229	NM_00140	Hs.535192
A_23_P154	2.575258	1.364717	2.575258	up	-0.544121	-0.566378	-0.611198	0.897442	0.544121	0.930892	DUSP15	dual specifi	FALSE	Homo sapi	128853	GO:000551	30449397	chr20	30449338	-	hs|20q11.2	NM_08061	chr20:3044	NM_08061	Hs.585017
A_22_P000	2.718342	1.442727	2.718342	up	-0.592688	-0.809117	-0.612392	0.820596	0.900699	0.592688	lnc-FSIP1-1	lnc-FSIP1-1	FALSE	LNCipedia lincRNA (lnc-FSIP1-1), li			39886424	chr15	39886365	-	hs|15q14		chr15:39886424-39886365		
A_33_P332	4.063629	2.022769	4.063629	up	-0.998436	-0.878862	-0.854042	1.166504	0.854042	1.31642	TNRC18	trinucleotic	FALSE	Homo sapi	84629	GO:000573	5352703	chr7	5352644	-	hs|7p22.1	NM_00108	chr7:53527	NM_00108	Hs.520638
A_22_P000	5.963639	2.576193	5.963639	up	-1.015655	-1.370136	-1.140597	1.529751	1.015655	1.656784			FALSE	long intergenic non-protein coding			58655599	chr2	58655540	+	hs|2p16.1		chr2:58655540-58655599		
A_33_P344	4.053117	2.019032	4.053117	up	-0.815529	-1.023908	-0.972966	1.193011	0.815529	1.236154			FALSE	AGENCOURT_6387596 NIH_MGC_71 Homo sapiens cDNA clone IMAGE:5529651 5', mRNA se								BM453041			Hs.740939
A_33_P338	6.124631	2.614623	6.124631	up	-1.323012	-1.192501	-1.369804	1.192501	1.280931	1.485119			FALSE	tubulin tyrosine ligase-like family,			50453671	chr22	50453612	-	hs|22q13.33		chr22:50453671-50453612		
A_23_P105	2.343663	1.228765	2.343663	up	-0.542133	-0.599473	-0.622028	0.706856	0.542133	0.673672	APPL2	adaptor pr	FALSE	Homo sapi	55198	GO:000551	1.06E+08	chr12	1.06E+08	-	hs|12q23.3	NM_01817	chr12:1055	NM_01817	Hs.506603
A_33_P321	4.312247	2.10844	4.312247	up	-0.94064	-1.182788	-0.915586	0.994422	1.376297	0.915586	KCNK2	potassium	FALSE	Homo sapi	3776	GO:001527	2.15E+08	chr1	2.15E+08	+	hs|1q41	NM_00101	chr1:21541	NM_00101	Hs.497745
A_33_P325	3.189219	1.673203	3.189219	up	-1.034561	-0.767186	-0.756773	0.942846	0.756773	0.76147			FALSE	1203217A dehydrogenase,glyceral			39647338	chrX	39647279	+	hs|Xp11.4		chrX:039647279-039647338		
A_22_P000	2.341998	1.22774	2.341998	up	-0.42534	-0.618545	-0.545023	0.803561	0.865412	0.42534	lnc-SRGAP3	lnc-SRGAP3	FALSE	Homo sapiens hypothetical gene s			9438303	chr3	9438244	-	hs|3p25.3	BC132680	chr3:9438303-9438244		Hs.598958
A_33_P321	2.688553	1.42683	2.688553	up	-0.53437	-0.779364	-0.692256	0.785777	0.53437	0.954352			FALSE	UI-E-EJ0-ahi-b-22-0-UI.s1 UI-E-EJ0			30832743	chr16	30832684	-	hs|16p11.2	BM674043	chr16:30832743-30832		Hs.681104
A_32_P584	2.224036	1.15318	2.224036	up	-0.464449	-0.723803	-0.651128	0.58721	0.464449	0.568501	KCND3	potassium	FALSE	potassium	3752	GO:000525	1.12E+08	chr1	1.12E+08	-	hs|1p13.2	AF070632	chr1:112313618-11231		Hs.731722
A_23_P401	2.594072	1.375218	2.594072	up	-0.472081	-1.075194	-0.961534	0.516572	0.472081	0.628192	TTC39C	tetratricop	FALSE	Homo sapi	125488		21712496	chr18	21711873	+	hs|18q11.2	NM_15321	chr18:2171	NM_15321	Hs.128576
A_23_P689	2.429454	1.280632	2.429454	up	-0.656903	-0.982788	-0.50255	0.623252	0.50255	0.573852	SSTR3	somatostat	FALSE	Homo sapi	6753	GO:000551	37602917	chr22	37602858	-	hs|22q13.1	NM_00105	chr22:3760	NM_00105	Hs.225995
A_21_P000	2.592139	1.374143	2.592139	up	-0.774568	-0.52291	-0.650255	0.587246	1.064539	0.52291	SNORA80B	small nucle	FALSE	Homo sapi	1E+08		10586962	chr2	10586903	-	hs|2p25.1	NR_02837	chr2:10586	NR_02837	Hs.730525
A_22_P000	3.388495	1.760645	3.388495	up	-0.733737	-0.850066	-0.722941	1.122934	0.722941	1.129314			FALSE				2.28E+08	chr1	2.28E+08	-	hs|1q42.13		chr1:227698356-227698297		
A_33_P322	2.058693	1.041729	2.058693	up	-0.336657	-0.585914	-0.388505	0.519692	0.957761	0.336657			FALSE				1.79E+08	chr5	1.79E+08	-	hs|5q35.3		chr5:178507837-178507778		
A_22_P000	9.762378	3.287233	9.762378	up	-1.631929	-1.697242	-1.326589	1.924459	1.326589	1.95489	lnc-TMEM1	lnc-TMEM1	FALSE	DA554227 HCHON2 Homo sapiens			66080463	chr11	66080404	+	hs|11q13.2	DA554227	chr11:66080404-66080		Hs.577306
A_33_P327	4.304746	2.105928	4.304746	up	-0.735729	-1.109499	-1.014544	1.499154	0.735729	1.223131			FALSE				20786879	chr15	20786820	-	hs|15q11.2		chr15:020786879-020786820		
A_21_P001	2.545952	1.348205	2.545952	up	-0.59292	-0.761754	-0.667224	0.713705	0.716094	0.59292	lnc-C7orf23	lnc-C7orf23	FALSE	PREDICTED: Homo sapiens unchar			86780818	chr7	86780759	-	hs|7q21.12	XM_00671	chr7:86780	XM_00671	Hs.633960
A_23_P499	2.366746	1.242905	2.366746	up	-0.593076	-0.549026	-0.575164	0.586958	0.875464	0.549026	KRT10	keratin 10,	FALSE	Homo sapi	3858	GO:000588	38974495	chr17	38974436	-	hs|17q21.2	NM_00042	chr17:3897	NM_00042	Hs.99936
A_33_P342	2.785646	1.478012	2.785646	up	-0.754065	-0.633476	-0.803751	0.633476	0.78865	0.820617			FALSE	HSENOAL2 alpha-enola		GO:000609	8934975	chr1	8934916	+	hs|1p36.23		chr1:008934916-008934975		
A_32_P443	4.511059	2.173466	4.511059	up	-1.001916	-1.073486	-1.038561	1.185363	1.001917	1.219154	EEF1A1	eukaryotic	FALSE	Homo sapi	1915	GO:000551	74229189	chr6	74229130	-	hs|6q13	NM_00140	chr6:74229	NM_00140	Hs.535192
A_24_P100	2.830952	1.501287	2.830952	up	-0.934197	-1.20299	-0.434682	0.765139	0.434682	0.732172			FALSE	long intergenic non-protein coding			46723274	chr21	46723215	-	hs|21q22.3		chr21:46723274-46723		Hs.171428
A_33_P333	4.610338	2.204873	4.610338	up	-1.232021	-1.169786	-0.920247	0.920247	1.30297	1.069347			FALSE				17286861	chr17	17286802	+	hs|17p11.2		chr17:017286802-017286861		
A_22_P000	2.433299	1.282913	2.433299	up	-1.107892	-0.390263	-0.727982	0.390263	0.72185	0.510489	lnc-C1orf31	lnc-C1orf31	FALSE	LNCipedia lincRNA (lnc-C1orf31-1)			2.34E+08	chr1	2.34E+08	+	hs|1q42.2	U09759	chr1:234492776-23449		Hs.484371
A_22_P000	2.932007	1.551889	2.932007	up	-0.655368	-0.724069	-0.810316	0.655368	0.943373	0.867171	lnc-FAM76	lnc-FAM76	FALSE	Q5VVR3_HUMAN (Q5VVR3) Interf			27996089	chr1	27996030	+	hs|1p36.11		chr1:27996030-27996089		
A_24_P418	2.057207	1.040687	2.057207	up	-0.362476	-0.382978	-0.406316	0.802584	0.362476	0.805231	GNAS	GNAS comp	FALSE	Homo sapi	2778	GO:000551	57485394	chr20	57485083	+	hs|20q13.3	NM_00107	chr20:5748	NM_00107	Hs.125898
A_22_P000	2.915734	1.543859	2.915734	up	-0.535952	-1.004705	-1.004266	0.739966	0.535952	0.810737	lnc-ATG7-2	lnc-ATG7-2	FALSE	BP417745 Homo sapiens small inte			11761209	chr3	11761150	+	hs|3p25.3	BP417745	chr3:11761150-117612		Hs.581519
A_23_P253	2.436952	1.285078	2.436952	up	-0.512346	-0.500996	-0.757481	0.846427	0.500996	0.736988	PFN2	profilin 2	FALSE	Homo sapi	5217	GO:000551	1.5E+08	chr3	1.5E+08	-	hs|3q25.1	NM_05302	chr3:14968	NM_05302	Hs.91747
A_33_P338	4.394563	2.13572	4.394563	up	-1.013777	-0.737695	-1.262395	1.38608	0.737695	1.269518	LINC00923	long interg	FALSE	Homo sapi	91948		98285957	chr15	98285898	-	hs|15q26.2	NR_02417	chr15:9828	NR_02417	Hs.130423
A_33_P340	2.633703	1.397093	2.633703	up	-0.579363	-0.679969	-0.706365	0.801525	0.579364	0.844691			FALSE				28966479	chr10	28966420	-	hs|10p12.1		chr10:028966479-028966420		
A_23_P134	2.518788	1.33273	2.518788	up	-0.890053	-0.561775	-0.612252	0.634962	0.561775	0.737371	CLDN23	claudin 23	FALSE	Homo sapi	137075	GO:001633	8561472	chr8	8561413	+	hs|8p23.1	NM_19428	chr8:85614	NM_19428	Hs.183617
A_33_P336	2.213085	1.146058	2.213085	up	-0.762669	-0.748705	-0.408647	0.614633	0.408647	0.494874	NAMPT	nicotinami	FALSE	Homo sapi	10135	GO:000551	1.06E+08	chr7	1.06E+08	-	hs|7q22.3	AK023341	chr7:105900620-105900561		
A_22_P000	7.492942	2.905532	7.492942	up	-1.113214	-1.684096	-1.420409	1.672406	1.113214	1.713258	LOC100129	uncharacte	FALSE	Homo sapi	1E+08		775622	chr4	775563	-	hs|4p16.3	NR_03651	chr4:77562	NR_03651	Hs.731755
A_21_P000	2.310129	1.207973	2.310129	up	-0.543432	-0.506354	-0.525743	0.582497	0.959539	0.506354	WFDC21P	WAP four-c	FALSE	Homo sapi	645638		58160989	chr17	58160930	-	hs|17q23.1	NR_03073	chr17:5816	NR_03073	Hs.463652
A_21_P001	5.222399	2.384713	5.222399	up	-1.315282	-1.175116	-0.916111	0.916111	1.877215	0.954301			FALSE	Q14867_HUMAN (Q14867) K222 p			33256590	chr22	33256531	-	hs|22q12.3		chr22:033256590-033256531		
A_24_P411	2.165068	1.114412	2.165068	up	-0.531996	-0.45528	-0.775684	0.529356	0.45528	0.595641	GPR126	G protein-c	FALSE	Homo sapi	57211	GO:000718	1.43E+08	chr6	1.43E+08	+	hs|6q24.1	NM_02045	chr6:14276	NM_02045	Hs.743302
A_22_P000	2.353643	1.234896	2.353643	up	-0.526674	-0.620727	-0.561152	0.526674	0.835288	0.634173	lnc-MUSK-2	lnc-MUSK-2	FALSE	Homo sapiens cDNA FLJ37814 fis,			1.13E+08	chr9	1.13E+08	+	hs|9q31.3	AK095133	chr9:113343325-11334		Hs.726867
A_33_P341	4.968292	2.31275	4.968292	up	-0.826934	-1.770899	-1.49016	1.17772	0.826934	0.845603	LYNX1	Ly6/neurot	FALSE	Homo sapi	66004	GO:000588	1.44E+08	chr8	1.44E+08	-	hs|8q24.3	NM_17745	chr8:14384	NM_17745	Hs.158665
A_22_P000	2.522434	1.334816	2.522434	up	-0.726299	-0.702666	-0.57367	0.57367	0.731217	0.696928			FALSE	603075994F1 NIH_MGC_119 Hom			1.27E+08	chr3	1.27E+08	+	hs|3q21.3		chr3:127209460-12720		Hs.581602
A_21_P000	2.393115	1.258889	2.393115	up	-0.79845	-0.456837	-0.747039	0.555641	0.761863	0.456837	lnc-DEC1-3	lnc-DEC1-3	FALSE	LNCipedia lincRNA (lnc-DEC1-3), li			1.18E+08	chr9	1.18E+08	+	hs|9q33.1		chr9:118454345-118454404		
A_23_P103	2.823269	1.497367	2.823269	up	-0.711482	-0.67004	-0.713885	0.671936	1.054717	0.67004	MAN1C1	mannosida	FALSE	Homo sapi	57134	GO:003017	26110894	chr1	26110835	+	hs|1p36.11	NM_02037	chr1:26110	NM_02037	Hs.197043
A_24_P312	4.280951	2.097931	4.280951	up	-0.820247	-0.72408	-1.051659	1.447764	0.72408	1.525963	PLAGL1	pleiomorph	FALSE	Homo sapi	5325	GO:000097	1.44E+08	chr6	1.44E+08	-	hs|6q24.2	NM_00671	chr6:14426	NM_00671	Hs.743225
A_21_P000	2.312528	1.209471	2.312528	up	-0.312742	-0.724662	-0.368195	1.079475	0.312742	0.830598	lnc-CCDC90	lnc-CCDC90	FALSE	LNCipedia lincRNA (lnc-CCDC90A-5			14448958	chr6	14448899	-	hs|6p23		chr6:14448958-14448899		
A_23_P746	4.674097	2.224688	4.674097	up	-1.128706	-1.03566	-1.111964	1.03566	1.316622	1.045452	G0S2	G0/G1 swit	FALSE	Homo sapi	50486	GO:000581	2.1E+08	chr1	2.1E+08	+	hs|1q32.2	NM_01571	chr1:20984	NM_01571	Hs.432132
A_33_P332	2.102885	1.07237	2.102885	up	-0.273149	-0.379877	-0.374618	0.754768	0.273149	1.161548	PLAGL2	pleiomorph	FALSE	Homo sapi	5326	GO:004356	30784257	chr20	30784198	-	hs|20q11.2	NM_00265	chr20:3078	NM_00265	Hs.154104
A_24_P352	5.201048	2.378802	5.201048	up	-0.989198	-0.970018	-1.198386	1.449579	0.970018	1.559209	FAM20A	family with	FALSE	Homo sapi	54757	GO:000579	66537099	chr17	66537040	-	hs|17q24.2	NM_01756	chr17:6653	NM_01756	Hs.268874
A_22_P000	2.71231	1.439522	2.71231	up	-0.590926	-0.770916	-0.636947	0.801321	0.590926	0.92753			FALSE				1.81E+08	chr1	1.81E+08	-	hs|1q25.3		chr1:181159813-181159754		
A_22_P000	4.29055	2.101163	4.29055	up	-0.743002	-0.962379	-1.194912	1.384491	0.743002	1.275702	lnc-TMCO3	lnc-TMCO3	FALSE	LNCipedia lincRNA (lnc-TMCO3-1),			1.14E+08	chr13	1.14E+08	+	hs|13q34		chr13:114130232-114130291		
A_33_P336	11.1534	3.479412	11.1534	up	-1.561916	-1.92969	-2.137836	1.561916	1.634837	1.61204	AMY1C	amylase, al	FALSE	Homo sapi	278	GO:000551	1.04E+08	chr1	1.04E+08	+	hs|1p21.1	NM_00100	chr1:10420	NM_00100	Hs.655232
A_21_P000	3.442428	1.783426	3.442428	up	-0.588299	-1.394102	-0.887583	0.946373	0.588299	0.945622	TMPRSS4-A	TMPRSS4 a	FALSE	Homo sapi	1.01E+08		1.18E+08	chr11	1.18E+08	-	hs|11q23.3	NR_03831	chr11:1179	NR_03831	Hs.627426

A_21_P000	2.073349	1.051963	2.073349	up	-0.348134	-0.40499	-0.207806	0.85901	0.207806	1.128143	lnc-GUSB-2	lnc-GUSB-2	FALSE	LNCipedia lincRNA (lnc-GUSB-2), li			65564698	chr7	65564639	-	hs|7q11.21		chr7:65564698-65564639		
A_24_P178	2.014686	1.010555	2.014686	up	-0.429086	-0.529782	-0.532591	0.609116	0.429086	0.502004			FALSE	ribosomal protein L18a pseudoger			1.11E+08	chrX	1.11E+08	-	hs|Xq23		chrX:110867823-110867764		
A_33_P349	6.007953	2.586874	6.007953	up	-1.113032	-1.493611	-1.051167	1.498148	1.051167	1.553496	SNORA71A	small nucle	FALSE	AGENCOUF	26777		37054976	chr20	37054917	-	hs|20q11.2	BM918074	chr20:37054976-37054		Hs.681779
A_33_P334	3.032716	1.60061	3.032716	up	-0.629902	-1.042915	-0.697641	0.897927	0.629903	0.903543			FALSE				29887696	chr16	29887637	-	hs|16p11.2		chr16:029887696-029887637		
A_22_P000	2.117287	1.082217	2.117287	up	-0.359452	-0.390872	-0.441135	0.926411	0.359452	0.769327	GAS6-AS1	GAS6 antis	FALSE	Homo sapi	650669		1.15E+08	chr13	1.15E+08	+	hs|13q34	NR_04499	chr13:1145	NR_04499	Hs.369201
A_22_P000	2.613295	1.38587	2.613295	up	-0.765254	-0.778486	-0.597334	0.700922	0.597334	0.718281	lnc-AL3535	lnc-AL3535	FALSE	LNCipedia lincRNA (lnc-AL353597.			37515052	chr6	37514993	+	hs|6p21.2		chr6:37514993-37515052		
A_22_P000	2.00949	1.006829	2.00949	up	-0.857514	-0.977485	0.14723	0.052654	-0.052654	1.332719	lnc-IDH3G-	lnc-IDH3G-	FALSE	BX364425 Homo sapiens NEUROBI			1.53E+08	chrX	1.53E+08	-	hs|Xq28	BX364425	chrX:153031905-15303		Hs.738472
A_24_P187	2.581605	1.368268	2.581605	up	-0.642475	-0.787858	-0.686566	0.644232	0.642475	0.7012	SVEP1	sushi, von \	FALSE	sushi, von \	79987	GO:000573	1.13E+08	chr9	1.13E+08	-	hs|9q31.3	BC030816	chr9:113238473-11323		Hs.522334
A_33_P330	2.089748	1.063329	2.089748	up	-0.348538	-0.528776	-0.336678	0.767967	0.336679	0.871349	LOC643549	uncharacte	FALSE	Homo sapi	643549		44563229	chr20	44563170	-	hs|20q13.1	BC133006	chr20:44563229-44563		Hs.472856
A_33_P327	3.259513	1.704657	3.259513	up	-0.607987	-1.038727	-0.614416	1.028104	0.607987	1.216749	HMX1	H6 family h	FALSE	Homo sapi	3166	GO:004356	8869424	chr4	8869365	-	hs|4p16.1	NM_01894	chr4:88694	NM_01894	Hs.104134
A_33_P368	3.080243	1.623044	3.080243	up	-0.715579	-0.743272	-0.878298	0.906101	0.715579	0.910306	PREPL	prolyl endc	FALSE	Homo sapi	9581	GO:000579	44549028	chr2	44548969	-	hs|2p21	NM_00603	chr2:44549	NM_00603	Hs.444349
A_33_P340	4.664967	2.221867	4.664967	up	-0.871241	-1.030229	-0.827613	1.555558	0.827613	1.553347			FALSE				50036	chrUn_gl00	49977	+			chrUn_gl000217:000049977-000050036		
A_33_P322	2.137716	1.09607	2.137716	up	-0.419404	-0.678743	-0.538935	0.419404	0.734547	0.497178	ARHGAP26	Rho GTPas	FALSE	Homo sapi	23092	GO:000551	1.43E+08	chr5	1.43E+08	+	hs|5q31.3	NM_01507	chr5:14260	NM_01507	Hs.654668
A_33_P331	7.097212	2.827252	7.097212	up	-1.320676	-1.20779	-1.022455	1.813514	1.022455	2.094867			FALSE				19193130	chr20	19193071	-	hs|20p11.23		chr20:019193130-019193071		
A_21_P001	9.029201	3.174598	9.029201	up	-1.42097	-1.613925	-1.416522	1.416522	2.200447	1.45541			FALSE				1.06E+08	chr7	1.06E+08	+	hs|7q22.3		chr7:105891330-105891389		
A_24_P203	4.558246	2.188479	4.558246	up	-0.8072	-1.237117	-0.875694	1.40486	0.8072	1.433366	LOC439951	uncharacte	FALSE	PREDICTED	439951	GO:000561	11653678	chr10	11653619	+	hs|10p14	XR_171055	chr10:1165	XR_171055	Hs.590986
A_22_P000	6.866602	2.779596	6.866602	up	-1.04868	-1.245648	-1.261294	1.824284	1.04868	1.910203	SLC38A3	solute carri	FALSE	Homo sapi	10991	GO:000529	50242781	chr3	50242722	+	hs|3p21.31	NM_00684	chr3:50242	NM_00684	Hs.76460
A_33_P335	2.775754	1.47288	2.775754	up	-0.762281	-0.688714	-0.676331	0.676331	0.844522	0.770459	CSF1	colony stim	FALSE	Homo sapi	1435	GO:004847	1.1E+08	chr1	1.1E+08	+	hs|1p13.3	NM_17221	chr1:11046	NM_17221	Hs.173894
A_22_P000	2.031025	1.022208	2.031025	up	-0.497113	-0.448591	-0.387578	0.691113	0.387578	0.654652	LSP1	lymphocyte	FALSE	Homo sapi	4046	GO:000716	1887897	chr11	1887838	+	hs|11p15.5	NM_00124	chr11:1887	NM_00124	Hs.56729
A_21_P000	3.732674	1.90021	3.732674	up	-0.894391	-0.904042	-1.108554	0.974413	0.924838	0.894391	SNORD15B	small nucle	FALSE	Homo sapi	114599		75115610	chr11	75115551	+	hs|11q13.4	NR_00002	chr11:7511	NR_00002	Hs.689080
A_33_P335	3.563653	1.833357	3.563653	up	-0.899815	-0.9776	-0.820169	1.023254	0.820169	0.959064	MIF-AS1	MIF antiser	FALSE	Homo sapi	284889		24236690	chr22	24236631	-	hs|22q11.2	NR_03891	chr22:2423	NR_03891	Hs.632772
A_22_P000	2.846227	1.509051	2.846227	up	-0.618265	-0.843185	-0.625205	0.877522	0.618265	0.94471	LRRC26	leucine rich	FALSE	Homo sapi	389816	GO:000551	1.4E+08	chr9	1.4E+08	-	hs|9q34.3	NM_00101	chr9:14006	NM_00101	Hs.669977
A_22_P000	2.200058	1.137541	2.200058	up	-0.673185	-0.459142	-0.56553	0.487937	0.767689	0.459142	LOC101929	uncharacte	FALSE	PREDICTED	1.02E+08		31596982	chr10	31596923	-	hs|10p11.2	XR_424109	chr10:3159	XR_424109	
A_23_P488	2.075917	1.053749	2.075917	up	-0.487204	-0.244273	-0.273148	0.925829	0.244273	0.986518	AP2A1	adaptor-rel	FALSE	Homo sapi	160	GO:007258	50309390	chr19	50309154	+	hs|19q13.3	NM_01420	chr19:5030	NM_01420	Hs.467125
A_33_P331	3.307916	1.725923	3.307916	up	-0.891535	-0.846624	-0.813247	0.813247	0.941107	0.872007	MT1HL1	metallothic	FALSE	Homo sapi	645745	GO:004687	2.37E+08	chr1	2.37E+08	-	hs|1q43	NM_00127	chr1:23716	NM_00127	Hs.632513
A_23_P262	3.308589	1.726216	3.308589	up	-0.649696	-1.168004	-0.944737	0.955293	0.649695	0.811223	TPSG1	tryptase ga	FALSE	Homo sapi	25823	GO:000425	1271739	chr16	1271680	-	hs|16p13.3	NM_01246	chr16:1271	NM_01246	Hs.592076
A_23_P170	2.319169	1.213608	2.319169	up	-0.498122	-0.499526	-0.401639	0.872297	0.401639	0.967602	CST5	cystatin D	FALSE	Homo sapi	1473	GO:001095	23858186	chr20	23856894	-	hs|20p11.2	NM_00190	chr20:2385	NM_00190	Hs.121489
A_32_P160	2.218554	1.14962	2.218554	up	-0.921814	-0.37157	-0.431853	0.579626	0.37157	0.772427	DOK6	docking prc	FALSE	Homo sapi	220164	GO:000551	67406329	chr18	67406270	+	hs|18q22.2	NM_15272	chr18:6740	NM_15272	Hs.278285
A_33_P336	2.49562	1.319398	2.49562	up	-0.577246	-0.535176	-0.693716	0.817796	0.535176	0.799084	THBS2	thrombosp	FALSE	Homo sapi	7058	GO:005196	1.7E+08	chr6	1.7E+08	-	hs|6q27	NM_00324	chr6:16962	NM_00324	Hs.371147
A_33_P324	11.01215	3.461025	11.01215	up	-1.66175	-1.674268	-1.656467	1.681286	2.052837	1.656467	KCTD12	potassium	FALSE	Homo sapi	115207	GO:004323	77456107	chr13	77456048	-	hs|13q22.3	NM_13844	chr13:7745	NM_13844	Hs.644125
A_33_P332	3.975811	1.991249	3.975811	up	-0.848816	-1.126514	-0.849039	1.111905	0.848816	1.188658			FALSE				2.37E+08	chr2	2.37E+08	-	hs|2q37.2		chr2:236673342-236673283		
A_33_P321	2.548106	1.349425	2.548106	up	-0.526197	-0.713481	-0.705983	0.751532	0.526197	0.824887	IQGAP2	IQ motif co	FALSE	Homo sapi	10788	GO:003431	76003854	chr5	76003795	+	hs|5q13.3	NM_00663	chr5:76003	NM_00663	Hs.291030
A_24_P691	2.137734	1.096083	2.137734	up	-0.465597	-0.551298	-0.538665	0.465597	0.76207	0.505021	WFDC21P	WAP four-c	FALSE	WAP four-c	645638		58162479	chr17	58161084	-	hs|17q23.1		chr17:58162479-58161084		
A_23_P502	2.32993	1.220287	2.32993	up	-0.780508	-0.556734	-0.476884	0.476884	0.809776	0.560074	RFX2	regulatory	FALSE	Homo sapi	5990	GO:000370	5993250	chr19	5993191	-	hs|19p13.3	NM_00063	chr19:5993	NM_00063	Hs.465709
A_33_P338	3.472155	1.795831	3.472155	up	-0.726433	-0.991092	-0.935588	1.009008	0.726434	0.998939	CCM2L	cerebral ca	FALSE	Homo sapi	140706	GO:003209	30607204	chr20	30607145	+	hs|20q11.2	NM_08062	chr20:3060	NM_08062	Hs.382151
A_32_P108	4.207597	2.072997	4.207597	up	-1.058052	-1.069782	-1.01964	1.031273	1.01964	1.020603	FAM20A	family with	FALSE	Homo sapi	54757	GO:000579	66533427	chr17	66533368	-	hs|17q24.2	NM_01756	chr17:6653	NM_01756	Hs.268874
A_33_P326	2.189877	1.13085	2.189877	up	-0.523151	-0.470719	-0.445377	0.449269	1.058656	0.445377	MAP7D3	MAP7 dom	FALSE	Homo sapi	79649	GO:000801	1.35E+08	chrX	1.35E+08	-	hs|Xq26.3	NM_00117	chrX:13529	NM_00117	Hs.446275
A_33_P330	4.037128	2.013329	4.037128	up	-0.80929	-1.467667	-0.918199	1.005403	0.80929	1.030139			FALSE				67276199	chr11	67276140	+	hs|11q13.2		chr11:067276140-067276199		
A_24_P256	3.929271	1.974262	3.929271	up	-0.839704	-0.965853	-0.773312	1.333655	0.773312	1.23695	NKX1-2	NK1 homec	FALSE	Homo sapi	390010	GO:004356	1.26E+08	chr10	1.26E+08	-	hs|10q26.1	NM_00114	chr10:1261	NM_00114	Hs.712041
A_23_P141	2.168961	1.117004	2.168961	up	-0.490609	-0.572257	-0.599231	0.538149	0.660157	0.490609	RCBTB2	regulator o	FALSE	Homo sapi	1102	GO:000508	49063410	chr13	49063351	-	hs|13q14.2	NM_00126	chr13:4906	NM_00126	Hs.652712
A_19_P003	2.352417	1.234144	2.352417	up	-0.670788	-0.510514	-0.754365	0.510514	0.739325	0.516925	PCAT19	prostate ca	FALSE	Homo sapi	1.01E+08		41985124	chr19	41985065	-	hs|19q13.2	NR_04010	chr19:4198	NR_04010	Hs.648878
A_24_P482	2.914162	1.543081	2.914162	up	-0.634723	-0.712704	-0.818659	0.763691	1.064742	0.634724	SECTM1	secreted ar	FALSE	Homo sapi	6398	GO:000716	80279241	chr17	80279182	-	hs|17q25.3	NM_00300	chr17:8027	NM_00300	Hs.558009
A_21_P000	2.689118	1.427133	2.689118	up	-0.418839	-0.835747	-0.624816	0.996257	0.418839	0.9869	LINC00173	long interg	FALSE	Homo sapi	1E+08		1.17E+08	chr12	1.17E+08	+	hs|12q24.2	NR_02734	chr12:1169	NR_02734	Hs.441601
A_22_P000	3.018407	1.593788	3.018407	up	-0.734262	-0.84924	-0.812786	0.842764	0.734263	0.808046	lnc-BEGAIN	lnc-BEGAIN	FALSE	LNCipedia lincRNA (lnc-BEGAIN-1),			1.01E+08	chr14	1.01E+08	-	hs|14q32.2		chr14:101055651-101055592		
A_33_P338	5.970794	2.577923	5.970794	up	-1.044576	-1.265276	-1.267209	1.563698	1.044576	1.548434	NFKBIL1	nuclear fac	FALSE	Homo sapi	4795	GO:000551	31516011	chr6	31515952	+	hs|6p21.33	NM_00114	chr6:31515	NM_00114	Hs.2764
A_33_P331	4.183531	2.064721	4.183531	up	-0.901487	-1.02282	-0.83925	1.243047	0.83925	1.348311			FALSE				12523165	chr8	12523106	-	hs|8p23.1		chr8:012523165-012523106		
A_33_P335	3.107148	1.635591	3.107148	up	-0.651523	-0.829011	-0.910214	0.864257	0.651523	1.000243			FALSE				1.78E+08	chr5	1.78E+08	+	hs|5q35.3		chr5:178157612-178157671		
A_33_P331	3.190797	1.673917	3.190797	up	-0.699993	-1.095055	-0.592342	0.941548	0.592341	1.100472	ARHGAP33	Rho GTPas	FALSE	Homo sapi	115703	GO:000551	36278984	chr19	36278925	+	hs|19q13.1	NM_00117	chr19:3627	NM_00117	Hs.515364
A_22_P000	2.10442	1.073422	2.10442	up	-0.596273	-0.482585	-0.43174	0.586122	0.691807	0.43174	lnc-C11orf4	lnc-C11orf4	FALSE	LNCipedia lincRNA (lnc-C11orf41-1			33731566	chr11	33731507	+	hs|11p13		chr11:33731507-33731566		
A_33_P342	4.442508	2.151374	4.442508	up	-0.965568	-1.086796	-1.117705	1.059611	1.258876	0.965568	CADM1	cell adhesi	FALSE	Homo sapi	23705	GO:000551	1.15E+08	chr11	1.15E+08	-	hs|11q23.3	NM_00130	chr11:1150	NM_00130	Hs.370510
A_33_P332	2.016795	1.012064	2.016795	up	-0.294221	-0.327671	-0.419133	0.845839	0.294221	0.855109	JUND	jun D protc	FALSE	Homo sapi	3727	GO:000551	18391430	chr19	18391371	-	hs|19p13.1	NM_00128	chr19:1839	NM_00128	Hs.2780
A_22_P000	7.164524	2.840871	7.164524	up	-1.313837	-1.646575	-1.418746	1.478669	1.313837	1.350949	lnc-RTL1-2	lnc-RTL1-2:	FALSE	LNCipedia lincRNA (lnc-RTL1-2), lir			1.02E+08	chr14	1.02E+08	-	hs|14q32.31		chr14:101537464-101537405		
A_22_P000	3.591531	1.844599	3.591531	up	-0.983901	-0.897451	-0.843773	0.988	0.843773	0.976898	lnc-ANKRD	lnc-ANKRD	FALSE	LNCipedia lincRNA (lnc-ANKRD6-1)			90383267	chr6	90383208	+	hs|6q15		chr6:90383208-90383267		
A_22_P000	3.416929	1.7727	3.416929	up	-0.718619	-0.685856	-0.703273	1.320519	0.685856	1.203978	lnc-ZKSCAN	lnc-ZKSCAN	FALSE	LNCipedia lincRNA (lnc-ZKSCAN1-1			99605803	chr7	99605744	+	hs|7q22.1		chr7:99605744-996058		Hs.742792
A_21_P000	5.724229	2.517082	5.724229	up	-1.674345	-1.087816	-1.290365	1.087816	1.32177	1.089132	LOC102477	uncharacte	FALSE	Homo sapi	1.02E+08		71870072	chr5	71870013	-	hs|5q13.2	NR_10500	chr5:71870	NR_10500	Hs.741936
A_24_P759	2.028586	1.020474	2.028586	up	-0.459046	-0.568214	-0.498451	0.493376	0.583291	0.459046	ITGB8	integrin, be	FALSE	Homo sapi	3696	GO:000830	20455196	chr7	20455137	+	hs|7p21.1	NM_00221	chr7:20455	NM_00221	Hs.592171
A_22_P000	2.19643	1.135161	2.19643	up	-0.588237	-0.425926	-0.580157	0.666318	0.425926	0.718918	LINC00900	long interg	FALSE	Homo sapi	283143		1.16E+08	chr11	1.16E+08	-	hs|11q23.3	NR_03414	chr11:1156	NR_03414	Hs.130499
A_23_P901	2.164488	1.114026	2.164488	up	-0.420784	-0.37935	-0.43723	0.786327	0.37935	0.939036	PPP1R15A	protein phc	FALSE	Homo sapi	23645	GO:000551	49378901	chr19	49378127	+	hs|19q13.3	NM_01433	chr19:4937	NM_01433	Hs.631593

A_19_P003	2.532468	1.340544	2.532468	up		-0.850219	-0.566253	-0.653614	0.58317	0.566253	0.802122	lnc-LTBP3-	lnc-LTBP3-	FALSE	Homo sapiens clone FLC0165 mRN			65267547	chr11	65267488	-		hs|11q13.1	AF130094	chr11:65267547-65267488		
A_33_P328	2.504045	1.324261	2.504045	up		-0.517583	-0.795565	-0.799739	0.805706	0.536605	0.517583	HOXA10	homeobox	FALSE	Homo sapi	3206	GO:000551	27213023	chr7	27211789	-		hs|7p15.2	NM_01895	chr7:27213	NM_01895	Hs.110637
A_23_P209	5.990641	2.58271	5.990641	up		-1.187501	-1.320458	-1.284759	1.229138	1.538774	1.187501	CYP1B1	cytochrom	FALSE	Homo sapi	1545	GO:003362	38295503	chr2	38295444	-		hs|2p22.2	NM_00010	chr2:38295	NM_00010	Hs.154654
A_23_P996	2.114551	1.080351	2.114551	up		-0.558127	-0.478675	-0.523978	0.566205	0.635393	0.478675	SLC7A7	solute carri	FALSE	Homo sapi	9056	GO:000652	23242663	chr14	23242604	-		hs|14q11.2	NM_00112	chr14:2324	NM_00112	Hs.513147
A_24_P316	2.339887	1.226439	2.339887	up		-0.389449	-0.60459	-0.675702	0.857623	0.389449	0.762503	NDUFA10	NADH dehy	FALSE	Homo sapi	4705	GO:002290	2.41E+08	chr2	2.41E+08	-		hs|2q37.3	NM_00454	chr2:24096	NM_00454	Hs.277677
A_22_P000	4.539969	2.182683	4.539969	up		-0.514958	-1.419452	-1.270293	1.397942	0.514958	1.430445	lnc-ANKRD	lnc-ANKRD	FALSE	LNCipedia lincRNA (lnc-ANKRD11-			89323646	chr16	89323587	-		hs|16q24.3		chr16:89323646-89323587		
A_23_P206	20.48564	4.356541	20.48564	up		-2.45899	-2.012495	-2.091517	2.012495	2.463418	2.03071	HP	haptoglobi	FALSE	Homo sapi	3240	GO:000551	72094936	chr16	72094877	+		hs|16q22.2	NM_00514	chr16:7209	NM_00514	Hs.513711
A_33_P340	2.011846	1.00852	2.011846	up		-0.372892	-0.374941	-0.072697	0.845333	0.072697	1.287			FALSE				53875228	chr18	53875169	-		hs|18q21.31		chr18:053875228-053875169		
A_33_P324	2.020045	1.014388	2.020045	up		-0.551684	-0.529373	-0.375197	0.375197	0.796171	0.415541	HRH1	histamine r	FALSE	Homo sapi	3269	GO:000588	11304938	chr3	11304879	+		hs|3p25.3	NM_00109	chr3:11304	NM_00109	Hs.1570
A_33_P334	2.175382	1.121269	2.175382	up		-0.448577	-0.595976	-0.447578	0.75004	0.447578	0.674056			FALSE				58245362	chr6	58245303	+		hs|6p11.2		chr6:058245303-058245362		
A_22_P000	4.317806	2.110298	4.317806	up		-0.94165	-0.962201	-0.920148	1.395448	0.920148	1.191301			FALSE				33025830	chr5	33025771	+		hs|5p13.3		chr5:33025771-33025830		
A_33_P339	6.605078	2.723576	6.605078	up		-1.382632	-1.608192	-0.955617	1.414147	0.955617	1.854523			FALSE				4643191	chr17	4643132	+		hs|17p13.2		chr17:004643132-004643191		
A_21_P000	3.588989	1.843578	3.588989	up		-0.41861	-2.222197	-0.766648	0.878732	0.41861	0.825935	lnc-TSC22D	lnc-TSC22D	FALSE	LNCipedia lincRNA (lnc-TSC22D1-1			44974260	chr13	44974201	-		hs|13q14.11		chr13:44974260-44974201		
A_21_P001	4.250998	2.087802	4.250998	up		-0.953036	-1.156714	-0.837342	1.299904	0.837342	1.179066			FALSE	Homo sapiens programmed cell de			1632759	chr5	1630626	-		hs|5p15.33		chr5:1632759-163062		Hs.720393
A_23_P206	2.26186	1.17751	2.26186	up		-0.5824	-0.550262	-0.545787	0.545787	0.760521	0.547772	MT1E	metallothic	FALSE	Homo sapi	4493	GO:007129	56660912	chr16	56660853	+		hs|16q12.2	NM_17561	chr16:5666	NM_17561	Hs.744893
A_22_P000	2.870534	1.521319	2.870534	up		-0.778673	-1.156706	-0.460064	0.710676	0.460064	0.997775	lnc-PCYOX1	lnc-PCYOX1	FALSE	AGENCOURT_8866852 NIH_MGC_			70529148	chr2	70529089	+		hs|2p13.3	BU501378	chr2:70529089-705291		Hs.737626
A_33_P336	2.308216	1.206778	2.308216	up		-0.41657	-0.754904	-0.72185	0.41657	0.807857	0.502583			FALSE	Q8IXM3_HUMAN (Q8IXM3) Mitoc			1.4E+08	chr9	1.4E+08	-		hs|9q34.3		chr9:140446712-140446653		
A_19_P003	2.235242	1.160431	2.235242	up		-0.425635	-0.572227	-0.541858	0.425635	0.827791	0.688148			FALSE	PREDICTED: Homo sapiens unchar			1.4E+08	chr5	1.4E+08	+		hs|5q31.3	XR_425926	chr5:13954	XR_425926	
A_22_P000	6.076237	2.603178	6.076237	up		-1.705444	-1.106747	-1.202312	1.106747	1.552453	1.135831	lnc-SLA2-2	lnc-SLA2-2	FALSE	LNCipedia lincRNA (lnc-SLA2-2), lir			35177589	chr20	35177530	-		hs|20q11.23		chr20:35177589-35177530		
A_22_P000	2.165839	1.114926	2.165839	up		-0.835197	-0.376966	-0.519994	0.509362	0.376966	0.726294			FALSE	BX118385 Soares_NFL_T_GBC_S1			15950993	chr2	15950934	-		hs|2p24.3		chr2:15950993-159509		Hs.738316
A_21_P000	2.867628	1.519858	2.867628	up		-0.689771	-0.767285	-0.607185	0.974877	0.607185	0.913269	lnc-STK35-	lnc-STK35-	FALSE	LNCipedia lincRNA (lnc-STK35-1), l			2181237	chr20	2181178	+		hs|20p13		chr20:2181178-2181237		
A_22_P000	2.847508	1.5097	2.847508	up		-0.578737	-0.70077	-0.722678	1.029076	0.578737	0.919102			FALSE	long intergenic non-protein coding			67946597	chr13	67946538	+		hs|13q21.32		chr13:67946538-67946597		
A_23_P383	2.737442	1.452828	2.737442	up		-0.726262	-0.666532	-0.690181	0.876505	0.666532	0.732474	GDA	guanine de	FALSE	Homo sapi	9615	GO:000614	74862346	chr9	74860160	+		hs|9q21.13	NM_00429	chr9:74860	NM_00429	Hs.494163
A_22_P000	4.804204	2.264297	4.804204	up		-1.217363	-1.283005	-0.983459	1.162282	1.163325	0.983459	EWSAT1	Ewing sarc	FALSE	long interg	283673		69367002	chr15	69366943	+		hs|15q23		chr15:69366943-69367		Hs.177927
A_21_P001	14.19988	3.827807	14.19988	up		-2.213518	-1.923317	-1.681871	2.030794	1.681871	1.95205	CES1	carboxylest	FALSE	Homo sapi	1066	GO:005268	55840252	chr16	55837463	-		hs|16q12.2	NM_00102	chr16:5584	NM_00102	Hs.558865
A_23_P592	2.162377	1.112618	2.162377	up		-0.605652	-0.470195	-0.567512	0.587685	0.470195	0.636616	TPBG	trophoblas	FALSE	Homo sapi	7162	GO:000998	83076120	chr6	83076061	+		hs|6q14.1	NM_00667	chr6:83076	NM_00667	Hs.82128
A_33_P329	9.435835	3.23815	9.435835	up		-1.555314	-1.543715	-1.631223	1.739266	1.543715	1.701217	CYP1B1	cytochrom	FALSE	Homo sapi	1545	GO:003362	38297765	chr2	38297706	-		hs|2p22.2	NM_00010	chr2:38297	NM_00010	Hs.154654
A_33_P331	2.750032	1.459448	2.750032	up		-0.863033	-0.698684	-0.580973	0.740694	0.580973	0.913988			FALSE				1.59E+08	chr4	1.59E+08	+		hs|4q32.1		chr4:158558989-158559048		
A_22_P000	2.138944	1.096899	2.138944	up		-0.39275	-0.527482	-0.526557	0.850551	0.392751	0.600605	PCBP1-AS1	PCBP1 anti	FALSE	Homo sapi	400960		70310729	chr2	70310670	-		hs|2p13.3	NR_03387	chr2:70310	NR_03387	Hs.716915
A_33_P337	2.10163	1.071509	2.10163	up		-0.424018	-0.586946	-0.416613	0.416613	0.927342	0.442995	LURAP1L	leucine rich	FALSE	Homo sapi	286343		12822892	chr9	12822833	+		hs|9p23	NM_20340	chr9:12822	NM_20340	Hs.445356
A_33_P336	3.01224	1.590837	3.01224	up		-0.858389	-0.581493	-0.963629	0.771458	0.581493	1.016047	C19orf26	chromoson	FALSE	chromoson	255057	GO:001602	1229297	chr19	1229238	-		hs|19p13.3	XM_00672	chr19:1229	XM_006722714	
A_21_P000	7.558897	2.918176	7.558897	up		-1.514201	-1.468845	-1.397593	1.429208	1.547087	1.397593	SNORD46	small nucle	FALSE	Homo sapi	94161		45242261	chr1	45242202	+		hs|1p34.1	NR_00002	chr1:45242	NR_000024	
A_23_P627	2.026684	1.019121	2.026684	up		-0.450857	-0.700205	-0.46702	0.460262	0.528163	0.450857	ELTD1	EGF, latrop	FALSE	Homo sapi	64123	GO:000718	79383345	chr1	79358842	-		hs|1p31.1	NM_02215	chr1:79383	NM_02215	Hs.132314
A_33_P337	2.406825	1.267131	2.406825	up		-0.865893	-0.640635	-0.506963	0.524283	0.506963	0.756657	LTK	leukocyte r	FALSE	Homo sapi	4058	GO:004306	41795900	chr15	41795841	-		hs|15q15.1	NM_00234	chr15:4179	NM_00234	Hs.434481
A_33_P340	7.345133	2.876789	7.345133	up		-1.348286	-1.319389	-1.451102	1.451164	1.741036	1.319389	SAA2	serum amy	FALSE	Homo sapi	6289	GO:003436	18266850	chr11	18266791	-		hs|11p15.1	NM_03075	chr11:1826	NM_03075	Hs.731376
A_22_P000	3.836119	1.939647	3.836119	up		-0.929932	-1.025558	-0.684499	1.265857	0.684499	1.228599	lnc-CR3920	lnc-CR3920	FALSE	LNCipedia lincRNA (lnc-CR392000.			1.39E+08	chr9	1.39E+08	+		hs|9q34.3		chr9:139010455-139010514		
A_21_P000	2.508777	1.326984	2.508777	up		-0.6978	-0.794646	-0.461391	0.615315	0.95041	0.461391	VEPH1	ventricular	FALSE	Homo sapi	79674	GO:000588	1.57E+08	chr3	1.57E+08	-		hs|3q25.32	NM_00116	chr3:15717	NM_00116	Hs.658046
A_24_P368	2.703156	1.434645	2.703156	up		-0.622506	-0.863698	-0.521814	0.802275	0.521814	0.971829	EVX1	even-skipp	FALSE	Homo sapi	2128	GO:004356	27286018	chr7	27285959	+		hs|7p15.2	NM_00198	chr7:27285	NM_00198	Hs.369879
A_22_P000	4.435765	2.149183	4.435765	up		-0.948028	-1.341847	-1.174027	1.016628	0.948028	1.018992			FALSE	Homo sapiens cDNA clone IMAGE:			78428310	chr17	78428251	-		hs|17q25.3	BC033347	chr17:78428310-78428		Hs.650532
A_22_P000	3.310113	1.726881	3.310113	up		-1.067457	-1.244114	-0.308619	0.744794	0.308619	1.507038	LOC102724	uncharacte	FALSE	PREDICTED	1.03E+08		41136436	chr15	41136377	-		hs|15q15.1	XR_424592	chr15:4113	XR_424592	Hs.569458
A_23_P132	3.110322	1.637064	3.110322	up		-0.887654	-0.547003	-0.538517	1.037363	0.538517	1.362138	C21orf58	chromoson	FALSE	Homo sapi	54058		47731417	chr21	47722479	-		hs|21q22.3	NM_00128	chr21:4773	NM_00128	Hs.236572
A_33_P334	3.501702	1.808056	3.501702	up		-0.795481	-0.706889	-0.768022	1.234559	0.706889	1.212328	DPP9-AS1	DPP9 antis	FALSE	Homo sapi	1E+08		4685929	chr19	4685870	+		hs|19p13.3	NM_00124	chr19:4685	NM_00124	Hs.678135
A_23_P146	3.759983	1.910726	3.759983	up		-0.717612	-1.118164	-0.973315	1.11572	0.717611	1.089757	UTS2R	urotensin 2	FALSE	Homo sapi	2837	GO:000588	80333296	chr17	80333237	+		hs|17q25.3	NM_01894	chr17:8033	NM_01894	Hs.192720
A_23_P236	9.262527	3.211406	9.262527	up		-1.645914	-1.47196	-1.711762	1.472133	1.860487	1.47196	AMY1C	amylase, al	FALSE	Homo sapi	278	GO:000551	1.04E+08	chr1	1.04E+08	+		hs|1p21.1	NM_00100	chr1:10420	NM_00100	Hs.655232
A_22_P000	2.323786	1.216477	2.323786	up		-0.871509	-0.410462	-0.765816	0.410462	0.735996	0.455187	lnc-DBX1-1	lnc-DBX1-1	FALSE	LNCipedia lincRNA (lnc-DBX1-1), li			20141299	chr11	20141240	-		hs|11p15.1		chr11:20141299-20141240		
A_22_P000	3.138504	1.650077	3.138504	up		-0.938932	-0.924651	-0.727296	0.787178	0.727296	0.844878	lnc-RAD1-2	lnc-RAD1-2	FALSE	PREDICTED: Homo sapiens unchar			34656616	chr5	34656557	-		hs|5p13.2	XM_37932	chr5:34656616-346565		Hs.534795
A_33_P321	4.10778	2.038359	4.10778	up		-0.94943	-1.163217	-0.739231	1.155958	0.73923	1.368011	PPDPF	pancreatic	FALSE	pancreatic	79144	GO:000727	62152993	chr20	62152934	+		hs|20q13.33		chr20:62152934-62152993		
A_22_P000	3.048018	1.607871	3.048018	up		-0.75515	-0.730016	-0.743943	0.961637	0.730016	0.902851	lnc-C10orf	lnc-C10orf	FALSE	17000600021474 GRN_PRENEU H			50627552	chr10	50627493	+		hs|10q11.2	CN291113	chr10:50627493-50627		Hs.568853
A_22_P000	2.923633	1.547762	2.923633	up		-1.010424	-0.670957	-0.691582	0.741488	0.857878	0.670957	ERVMER34	endogenou	FALSE	Homo sapi	1E+08	GO:001903	53617162	chr4	53617103	-		hs|4q12	NM_00124	chr4:53617	NM_00124	Hs.363087
A_19_P003	2.159477	1.110682	2.159477	up		-0.700392	-0.724207	-0.123471	0.477167	0.123471	1.183338	LINC01122	long interg	FALSE	Homo sapi	400955		59252064	chr2	59252005	+		hs|2p16.1	NR_03387	chr2:59252	NR_03387	Hs.503210
A_33_P340	2.390742	1.257458	2.390742	up		-0.544853	-0.676077	-0.482032	0.696221	0.482032	0.891161			FALSE				37308350	chr17	37308291	+		hs|17q12		chr17:037308291-037308350		
A_22_P000	4.520943	2.176624	4.520943	up		-1.146385	-0.796274	-0.665328	1.552251	0.665328	1.704305	lnc-GPR15-	lnc-GPR15-	FALSE	AY359121 C3ORF4 {Homo sapiens}			98241677	chr3	98241618	+		hs|3q11.2		chr3:98241618-98241677		
A_23_P502	2.039885	1.028488	2.039885	up		-0.459612	-0.425465	-0.124089	0.813583	0.124089	1.138627	CLPTM1	cleft lip and	FALSE	Homo sapi	1209	GO:000551	45495641	chr19	45495582	+		hs|19q13.3	NM_00129	chr19:4549	NM_00129	Hs.444441
A_21_P001	3.735633	1.901353	3.735633	up		-0.906442	-0.831397	-0.793268	1.194181	0.793268	1.185501			FALSE	supervillin pseudogene 1 [Source:H			30980007	chr10	30978376	+		hs|10p11.23		chr10:30978376-30980007		
A_33_P333	2.00206	1.001485	2.00206	up		-0.382417	-0.383396	-0.425165	0.382417	0.841272	0.58979	SCARNA13	small Cajal	FALSE	Homo sapi	677768		95999751	chr14	95999692	-		hs|14q32.1	NR_00300	chr14:9599	NR_00300	Hs.448753
A_22_P000	4.124473	2.04421	4.124473	up		-0.763985	-1.115451	-1.039339	1.283514	0.763985	1.166356			FALSE				74281507	chr8	74281448	-		hs|8q21.11		chr8:74281507-74281448		
A_23_P575	2.366396	1.242692	2.366396	up		-0.626903	-0.490843	-0.652229	0.907668	0.490843	0.559589	DDX17	DEAD (Asp-	FALSE	Homo sapi	10521	GO:000551	38882199	chr22	38882140	-		hs|22q13.1	NM_00638	chr22:3888	NM_00638	Hs.528305
A_24_P258	2.536621	1.342908	2.536621	up		-0.609127	-0.628537	-0.661229	0.894587	0.626117	0.609127	NFATC1	nuclear fac	FALSE	Homo sapi	4772	GO:000551	77227513	chr18	77227454	+		hs|18q23	NM_17239	chr18:7722	NM_17239	Hs.534074

A_24_P353	2.330047	1.220359	2.330047	up	-0.514626	-0.300725	-0.352829	1.032145	0.300725	1.160026	ALPL	alkaline ph	FALSE	Homo sapi	249	GO:000551	21903884	chr1	21903084	+	hs|1p36.12	NM_00047	chr1:21903	NM_00047	Hs.75431
A_33_P362	2.085794	1.060597	2.085794	up	-0.4729	-0.454559	-0.507684	0.585401	0.454559	0.706687	PKI55	DKFZp434H	FALSE	Homo sapi	150967		2.17E+08	chr2	2.17E+08	+	hs|2q35	NR_037701	chr2:21708	NR_037701	Hs.56876
A_22_P000	4.233183	2.081743	4.233183	up	-0.779645	-1.416037	-0.858419	1.167874	0.779645	1.243609			FALSE				70796305	chr15	70796246	-	hs|15q23		chr15:70796305-70796246		
A_23_P567	6.162247	2.623457	6.162247	up	-1.171181	-1.639503	-1.115588	1.311599	1.115588	1.516912	TUBA3D	tubulin, alp	FALSE	Homo sapi	113457	GO:000551	1.32E+08	chr2	1.32E+08	+	hs|2q21.1	NM_08038	chr2:13224	NM_08038	Hs.503749
A_22_P000	5.257387	2.394346	5.257387	up	-1.474888	-1.02337	-1.122928	1.385173	1.153308	1.02337	HLX-AS1	HLX antiser	FALSE	Homo sapi	1.01E+08		2.21E+08	chr1	2.21E+08	-	hs|1q41	NR_046901	chr1:22105	NR_046901	
A_22_P000	2.686989	1.425991	2.686989	up	-0.990701	-0.629236	-0.5506	0.865238	0.5506	0.691598	DNAJC27-A	DNAJC27 a	FALSE	Homo sapi	729723		25195101	chr2	25195042	+	hs|2p23.3	NR_03411	chr2:25195	NR_03411	Hs.436366
A_32_P468	2.840593	1.506192	2.840593	up	-0.676323	-0.619694	-1.119545	0.702208	0.619694	0.781113	LOC72968	uncharacte	FALSE	Homo sapi	729680		21276863	chr13	21276804	-	hs|13q12.1	AK090664	chr13:21276863-2127		Hs.655142
A_22_P000	2.908443	1.540247	2.908443	up	-0.450473	-0.646463	-0.856556	1.310737	0.450473	0.906037			FALSE				17216107	chr1	17216048	-	hs|1p36.13	BC070363	chr1:17216107-17216		Hs.465405
A_23_P142	2.296994	1.199747	2.296994	up	-0.594446	-0.542031	-0.532831	0.532832	0.803352	0.593747	RPL37A	ribosomal	FALSE	Homo sapi	6168	GO:001046	2.17E+08	chr2	2.17E+08	+	hs|2q35	NM_00099	chr2:21736	NM_00099	Hs.433701
A_23_P320	2.300652	1.202043	2.300652	up	-0.717815	-0.428174	-0.731304	0.428174	0.699098	0.601564	RGS16	regulator o	FALSE	Homo sapi	6004	GO:004354	1.83E+08	chr1	1.83E+08	-	hs|1q25.3	NM_00292	chr1:18256	NM_00292	Hs.413297
A_22_P000	2.826282	1.498906	2.826282	up	-0.740137	-0.781346	-0.740166	0.740136	0.754777	0.740154	MT1B	metallothic	FALSE	Homo sapi	4490	GO:007129	56686548	chr16	56686489	+	hs|16q12.2	NM_00594	chr16:5668	NM_00594	Hs.656629
A_22_P000	2.710167	1.438382	2.710167	up	-0.737766	-0.549633	-0.550161	0.956459	0.549633	0.971493	lnc-TRIM41	lnc-TRIM41	FALSE	LNCipedia lincRNA (lnc-TRIM41-3),			1.81E+08	chr5	1.81E+08	+	hs|5q35.3		chr5:180618957-180619016		
A_24_P335	2.950441	1.560931	2.950441	up	-0.761313	-0.77076	-0.768086	0.771269	0.850053	0.761313	SAA1	serum amy	FALSE	Homo sapi	6288	GO:000166	18291310	chr11	18290868	+	hs|11p15.1	NM_00033	chr11:1829	NM_00033	Hs.632144
A_33_P333	2.147206	1.10246	2.147206	up	-0.813933	-0.951465	-0.33436	0.395769	0.477495	0.33436			FALSE				42894280	chr20	42894221	+	hs|20q13.12		chr20:042894221-042894280		
A_24_P156	3.442046	1.783267	3.442046	up	-0.781425	-0.865997	-0.767601	1.053769	0.767601	1.113407			FALSE	Homo sapiens chromosome 19 op			4909287	chr19	4909228	-	hs|19p13.3	BC137009	chr19:4909287-490922		Hs.108106
A_33_P322	4.117366	2.041722	4.117366	up	-0.816482	-1.131077	-1.107005	1.123856	0.816482	1.130262	LHX3	LIM homec	FALSE	Homo sapi	8022	GO:000566	1.39E+08	chr9	1.39E+08	-	hs|9q34.3	NM_01456	chr9:13908	NM_01456	Hs.148427
A_23_P401	2.626031	1.392884	2.626031	up	-0.667605	-0.555794	-0.663071	0.904941	0.555794	0.831447	CDH22	cadherin 2	FALSE	Homo sapi	64405	GO:001633	44802794	chr20	44802735	-	hs|20q13.1	NM_02124	chr20:4480	NM_02124	Hs.472861
A_22_P000	11.7692	3.556944	11.7692	up	-1.391381	-2.258331	-2.049236	1.992605	1.391381	1.5879	ERICH1-AS	ERICH1 ant	FALSE	Homo sapi	619343		687825	chr8	687766	+	hs|8p23.3	NR_07339	chr8:68776	NR_07339	Hs.722154
A_22_P000	2.032557	1.023296	2.032557	up	-0.448837	-0.332373	-0.396288	0.820092	0.332373	0.739924			FALSE	Homo sapiens mRNA; cDNA DKFZ			22221998	chr6	22221939	+	hs|6p22.3	AL117559	chr6:22221939-22221		Hs.233120
A_24_P127	3.347551	1.743106	3.347551	up	-0.999541	-0.738884	-0.630246	0.872594	0.630246	1.357807	MAFA	v-maf aviar	FALSE	Homo sapi	389692	GO:004356	1.45E+08	chr8	1.45E+08	-	hs|8q24.3	NM_20158	chr8:14451	NM_20158	Hs.521914
A_24_P566	5.477641	2.453555	5.477641	up	-0.965232	-1.110133	-0.90623	1.779139	0.90623	1.693701	ZNF205	zinc finger	FALSE	Homo sapi	7755	GO:000551	3169894	chr16	3169835	+	hs|16p13.3	NM_00345	chr16:3169	NM_00345	Hs.592088
A_32_P207	2.086847	1.061325	2.086847	up	-0.590246	-0.402678	-0.680107	0.599228	0.402678	0.509038	CT47A11	cancer/test	FALSE	Homo sapi	255313		1.2E+08	chrX	1.2E+08	-	hs|Xq24	NM_17357	chrX:12007	NM_17357	Hs.522775
A_23_P516	2.245436	1.166996	2.245436	up	-0.761896	-0.60758	-0.464964	0.464965	0.562585	0.638997	MEF2D	myocyte er	FALSE	Homo sapi	4209	GO:000551	1.56E+08	chr1	1.56E+08	-	hs|1q22	NM_00592	chr1:15643	NM_00592	Hs.314327
A_24_P810	2.071565	1.050721	2.071565	up	-0.416242	-0.5437	-0.211094	0.794027	0.211094	0.976006	MXRA7	matrix-rem	FALSE	Homo sapi	439921	GO:001602	74676626	chr17	74676567	-	hs|17q25.1	NM_00100	chr17:7467	NM_00100	Hs.250723
A_33_P336	2.002466	1.001777	2.002466	up	-0.358889	-0.464814	-0.372538	0.761937	0.358889	0.688264			FALSE				68409003	chr9	68408944	-	hs|9q13		chr9:068409003-068408944		
A_33_P321	2.581641	1.368288	2.581641	up	-0.754262	-0.559611	-0.722575	0.785873	0.559611	0.722931	TLE1	transducin-	FALSE	Homo sapi	7088	GO:000551	84225208	chr9	84225149	-	hs|9q21.32	NM_00130	chr9:84225	NM_00130	Hs.197320
A_33_P328	6.506463	2.701874	6.506463	up	-0.965095	-1.601017	-1.607907	1.647749	0.965096	1.318757	FOXP4	forkhead b	FALSE	Homo sapi	116113	GO:000750	41567697	chr6	41567638	+	hs|6p21.1	NM_00101	chr6:41567	NM_00101	Hs.131436
A_23_P337	2.690448	1.427846	2.690448	up	-0.728539	-0.682129	-0.705065	0.682129	0.738467	0.747209	DHRS3	dehydroge	FALSE	Homo sapi	9249	GO:000760	12628169	chr1	12628110	-	hs|1p36.22	NM_00475	chr1:12628	NM_00475	Hs.289347
A_19_P008	2.060103	1.042717	2.060103	up	-0.466177	-0.498757	-0.498004	0.565914	0.466177	0.633122	ARPC2	actin relate	FALSE	Homo sapi	10109	GO:000551	2.19E+08	chr2	2.19E+08	+	hs|2q35	NM_15286	chr2:21911	NM_15286	Hs.529303
A_33_P334	2.335793	1.223912	2.335793	up	-0.577139	-0.659291	-0.488543	0.698833	0.488542	0.759389	TMEM88B	transmemb	FALSE	Homo sapi	643965	GO:001602	1363107	chr1	1363048	+	hs|1p36.33	NM_00114	chr1:13630	NM_00114	Hs.729765
A_22_P000	3.941682	1.978812	3.941682	up	-0.859578	-1.033551	-1.029956	1.065114	0.859578	1.088657			FALSE	603052692F1 NIH_MGC_122 Hom			1.34E+08	chr11	1.34E+08	-	hs|11q25	BI766433	chr11:133906851-1339		Hs.573898
A_24_P406	5.360445	2.422353	5.360445	up	-1.237303	-1.062791	-1.221017	1.062791	1.566456	1.116702	GREM2	gremlin 2,	FALSE	Homo sapi	64388	GO:006030	2.41E+08	chr1	2.41E+08	-	hs|1q43	NM_02246	chr1:24065	NM_02246	Hs.98206
A_23_P206	2.079176	1.056012	2.079176	up	-0.865549	-0.590806	-0.40341	0.40341	0.473675	0.431186	NQO1	NAD(P)H d	FALSE	Homo sapi	1728	GO:000551	69744333	chr16	69744274	-	hs|16q22.1	NM_00090	chr16:6974	NM_00090	Hs.406515
A_24_P393	3.166737	1.662997	3.166737	up	-0.818386	-0.746667	-0.796474	0.746667	1.04662	0.834178	GDA	guanine de	FALSE	Homo sapi	9615	GO:000614	74867001	chr9	74866942	+	hs|9q21.13	NM_00429	chr9:74866	NM_00429	Hs.494163
A_24_P447	2.482782	1.311958	2.482782	up	-0.540667	-0.549335	-0.521672	0.521671	1.211742	0.590786	CSRP2	cysteine ar	FALSE	Homo sapi	1466	GO:000367	77252613	chr12	77252554	-	hs|12q21.2	NM_00132	chr12:7725	NM_00132	Hs.530904
A_33_P323	2.70846	1.437473	2.70846	up	-0.787671	-0.665735	-0.55943	0.818525	0.55943	0.921626	PSD	pleckstrin a	FALSE	Homo sapi	5662	GO:000551	1.04E+08	chr10	1.04E+08	-	hs|10q24.3	NM_00277	chr10:1041	NM_00277	Hs.154658
A_22_P000	2.159455	1.110668	2.159455	up	-0.502582	-0.557873	-0.590347	0.652484	0.526134	0.502582	LOC10192	uncharacte	FALSE	PREDICTED	1.02E+08		57112577	chr4	57112518	-	hs|4q12	XR_245260	chr4:57112	XR_245260	Hs.637117
A_33_P328	3.900868	1.963795	3.900868	up	-0.848178	-0.999999	-0.552527	1.583042	0.552527	1.355113	C11orf95	chromoson	FALSE	Homo sapi	65998		63533339	chr11	63533280	-	hs|11q13.1	NM_00114	chr11:6353	NM_00114	Hs.191073
A_23_P138	2.058904	1.041877	2.058904	up	-0.401194	-0.29688	-0.393145	0.858543	0.29688	0.878989	ATN1	atrophin 1	FALSE	Homo sapi	1822	GO:000551	7051314	chr12	7051255	+	hs|12p13.3	NM_00100	chr12:7051	NM_00100	Hs.143766
A_32_P930	2.357242	1.2371	2.357242	up	-0.45035	-0.795093	-0.43706	0.43706	0.721723	0.870013			FALSE	Synthetic construct Homo sapiens			45411956	chr19	45411897	-	hs|19q13.3	CU678501	chr19:045411956-045411897		
A_33_P321	3.428374	1.777525	3.428374	up	-0.716784	-0.936636	-0.756979	1.159467	0.716784	1.045925	ELFN1	extracellula	FALSE	Homo sapi	392617	GO:005080	1787459	chr7	1787400	+	hs|7p22.3	NM_00112	chr7:17874	NM_00112	Hs.42896
A_33_P339	2.213556	1.146366	2.213556	up	-0.585	-0.639918	-0.535579	0.591292	0.53558	0.551728	LOC10013	uncharacte	FALSE	PREDICTED	1E+08		90235494	chr16	90235435	+	hs|16q24.3	XR_429801	chr16:9023	XR_429801	Hs.661255
A_33_P322	2.73163	1.449762	2.73163	up	-0.727003	-0.618067	-0.685387	0.618067	1.061601	0.639161	SNORD3B-	small nucle	FALSE	Homo sapi	26851		18965441	chr17	18965382	+	hs|17p11.2	NR_00327	chr17:1896	NR_00327	Hs.658815
A_21_P001	3.47859	1.798502	3.47859	up	-0.724282	-0.757838	-0.678174	1.31217	0.678174	1.24487	BRD9	bromodom	FALSE	bromodom	65980	GO:000815	880158	chr5	880099	-	hs|5p15.33		chr5:880158-880099		
A_23_P743	2.275165	1.185971	2.275165	up	-0.666887	-0.801657	-0.443665	0.618572	0.443665	0.583467	LINC00626	long interg	FALSE	Homo sapi	79100		1.69E+08	chr1	1.69E+08	+	hs|1q24.2	NR_02416	chr1:16876	NR_02416	Hs.250624
A_23_P256	3.591645	1.844645	3.591645	up	-0.696952	-1.130336	-0.72623	1.121203	0.696952	1.16226	GALR3	galanin rec	FALSE	Homo sapi	8484	GO:000763	38221385	chr22	38221326	+	hs|22q13.1	NM_00361	chr22:3822	NM_00361	Hs.158353
A_33_P334	2.319028	1.21352	2.319028	up	-0.444323	-0.649165	-0.842739	0.6798	0.444323	0.580212	AATK	apoptosis-a	FALSE	apoptosis-a	9625	GO:000551	79139542	chr17	79139483	-	hs|17q25.3		chr17:79139542-79139483		
A_21_P000	2.016023	1.011512	2.016023	up	-0.641468	-0.412094	-0.445264	0.532054	0.591562	0.412094	LINC01111	long interg	FALSE	Homo sapi	1.02E+08		77436562	chr8	77436503	+	hs|8q21.11	NR_10500	chr8:77436	NR_10500	Hs.615028
A_33_P329	2.699232	1.432549	2.699232	up	-0.807717	-0.683662	-0.514959	0.874406	0.514959	0.901944	SFXN5	sideroflexir	FALSE	Homo sapi	94097	GO:001574	73215461	chr2	73215402	-	hs|2p13.2	NM_14457	chr2:73215	NM_14457	Hs.368171
A_33_P115	2.021079	1.015126	2.021079	up	-0.466961	-0.402437	-0.658979	0.492669	0.621892	0.402437	PHYH	phytanoyl-	FALSE	Homo sapi	5264	GO:000551	13320342	chr10	13320283	-	hs|10p13	NM_00103	chr10:1332	NM_00103	Hs.498732
A_33_P328	2.068963	1.048908	2.068963	up	-0.539037	-0.47011	-0.545602	0.479188	0.642677	0.47011	TMEM37	transmemb	FALSE	Homo sapi	140738	GO:000526	1.2E+08	chr2	1.2E+08	+	hs|2q14.2	NM_18324	chr2:12019	NM_18324	Hs.26216
A_23_P218	2.045548	1.032488	2.045548	up	-0.440298	-0.452225	-0.390604	0.634851	0.390604	0.788882			FALSE	immunoglobulin heavy constant g			1.06E+08	chr14	1.06E+08	-	hs|14q32.3	BC024289	chr14:106208310-1062		Hs.510635
A_22_P000	5.003243	2.322864	5.003243	up	-0.892719	-1.783999	-1.129241	1.205175	0.892718	1.064738	lnc-DOLPP	lnc-DOLPP	FALSE	LNCipedia lincRNA (lnc-DOLPP1-1)			1.32E+08	chr9	1.32E+08	+	hs|9q34.11		chr9:131860266-131860325		
A_21_P000	2.071189	1.050459	2.071189	up	-0.563677	-0.476354	-0.553884	0.542964	0.476354	0.538145	LOC10028	uncharacte	FALSE	Homo sapi	1E+08		46781579	chr12	46777935	+	hs|12q13.1	NR_125381	chr12:4677	NR_125381	
A_23_P153	2.017197	1.012352	2.017197	up	-0.299189	-0.44345	-0.311571	0.961067	0.29919	0.72259	KRTAP2-4	keratin ass	FALSE	Homo sapi	85294	GO:004509	39221369	chr17	39221320	-	hs|17q21.2	NM_03318	chr17:3922	NM_03318	Hs.560502
A_33_P339	2.417279	1.273384	2.417279	up	-0.60559	-0.720248	-0.646447	0.63402	0.605591	0.608255	TMEM119	transmemb	FALSE	Homo sapi	338773	GO:000815	1.09E+08	chr12	1.09E+08	-	hs|12q23.3	NM_18172	chr12:1089	NM_18172	Hs.449718
A_33_P327	2.270381	1.182934	2.270381	up	-0.680398	-0.683043	-0.295328	0.900411	0.295328	0.694295			FALSE	Homo sapiens mRNA, endogenous			68598273	chr6	68598214	-	hs|6q12	AB128832	chr6:68598273-685982		Hs.289232
A_23_P109	4.917687	2.29798	4.917687	up	-0.906911	-0.831062	-1.140753	1.393834	0.831062	1.790318	AVP	arginine va	FALSE	Homo sapi	551	GO:003030	3063624	chr20	3063391	-	hs|20p13	NM_00049	chr20:3063	NM_00049	Hs.89648

A_23_P150	2.390412	1.257259	2.390412	up		-0.588194	-0.466364	-0.511505	0.820028	0.466364	0.919323	LRP5	low density	FALSE	Homo sapi	4041	GO:000551	68216418	chr11	68216359	+		hs|11q13.2	NM_00233	chr11:6821	NM_00233	Hs.6347
A_22_P000	2.283143	1.191021	2.283143	up		-0.46743	-0.494843	-0.715967	0.650039	0.46743	0.777355			FALSE				1.38E+08	chr9	1.38E+08	+		hs|9q34.3		chr9:138245095-138245154		
A_24_P831	6.19023	2.629993	6.19023	up		-1.379854	-1.761857	-0.86127	1.280583	0.86127	1.745144	C1orf229	chromoson	FALSE	Homo sapi	388759		2.47E+08	chr1	2.47E+08	-		hs|1q44	NM_20740	chr1:24727	NM_20740	Hs.456511
A_33_P329	4.490832	2.166983	4.490832	up		-0.820938	-1.522005	-1.068687	1.141629	0.820937	1.126752	NKX2-5	NK2 homeo	FALSE	Homo sapi	1482	GO:000551	1.73E+08	chr5	1.73E+08	-		hs|5q35.1	NM_00116	chr5:17265	NM_00116	Hs.54473
A_22_P000	3.356051	1.746765	3.356051	up		-0.948473	-1.489921	-0.329299	0.635133	0.329299	1.508167			FALSE				1.34E+08	chr10	1.34E+08	+		hs|10q26.3		chr10:134232627-134232686		
A_33_P336	2.055819	1.039713	2.055819	up		-0.522487	-0.457411	-0.414222	0.414222	0.757317	0.55348	NAMPT	nicotinami	FALSE	Homo sapi	10135	GO:000551	1.06E+08	chr7	1.06E+08	-		hs|7q22.3	NM_00574	chr7:10591	NM_00574	Hs.489615
A_33_P337	2.030683	1.021965	2.030683	up		-0.549365	-0.532404	-0.459756	0.506132	0.459756	0.558482			FALSE				41406	chr17	41347	+		hs|17p13.3		chr17:000041347-000041406		
A_33_P340	2.708619	1.437558	2.708619	up		-0.689957	-0.613694	-0.55859	1.033748	0.858093	0.55859			FALSE				1.84E+08	chr1	1.84E+08	+		hs|1q25.3		chr1:183604736-183604795		
A_33_P337	2.108325	1.076098	2.108325	up		-0.86468	-0.329516	-0.647114	0.720254	0.329516	0.337212	PPP1R13B	protein pho	FALSE	Homo sapi	23368	GO:000551	1.04E+08	chr14	1.04E+08	-		hs|14q32.3	NM_01531	chr14:1042	NM_01531	Hs.709297
A_21_P001	2.029348	1.021016	2.029348	up		-0.555494	-0.654907	-0.413187	0.413187	0.485114	0.54116	lnc-TSC1-1	lnc-TSC1-1:	FALSE	Homo sapiens mRNA expressed or			1.36E+08	chr9	1.36E+08	-		hs|9q34.13	AB019568	chr9:135896274-13589		Hs.535192
A_23_P129	2.097832	1.0689	2.097832	up		-0.424758	-0.384254	-0.481138	0.790657	0.384254	0.741636	IDH2	isocitrate d	FALSE	Homo sapi	3418	GO:000028	90630706	chr15	90630472	-		hs|15q26.1	NM_00216	chr15:9063	NM_00216	Hs.596461
A_24_P145	2.223691	1.152956	2.223691	up		-0.339931	-0.374424	-0.327419	0.993615	0.327419	1.096061	SERINC2	serine inco	FALSE	Homo sapi	347735	GO:000666	31902364	chr1	31902305	+		hs|1p35.2	NM_17886	chr1:31902	NM_17886	Hs.270655
A_21_P001	2.300739	1.202097	2.300739	up		-0.5034	-0.644318	-0.470193	0.769898	0.470193	0.748289			FALSE	Gene marker which use for analys			1E+08	chr15	1E+08	+		hs|15q26.3		chr15:100379122-100379181		
A_23_P101	2.708067	1.437264	2.708067	up		-0.62914	-0.57102	-0.610026	0.930534	0.57102	1.00005	C3	complemer	FALSE	Homo sapi	718	GO:000551	6679434	chr19	6679177	-		hs|19p13.3	NM_00006	chr19:6679	NM_00006	Hs.529053
A_33_P321	2.618437	1.388706	2.618437	up		-0.500213	-0.567517	-0.475871	1.048689	0.475871	1.097956			FALSE				19625495	chr14	19625436	-		hs|14q11.2		chr14:019625495-019625436		
A_22_P000	2.054454	1.038755	2.054454	up		-0.715513	-0.696547	-0.25277	0.573597	0.25277	0.625069			FALSE				96322144	chr14	96322085	-		hs|14q32.2		chr14:96322144-96322085		
A_33_P336	5.095995	2.349364	5.095995	up		-1.062839	-1.350636	-1.111231	1.19062	1.06284	1.269927	CISD3	CDGSH iror	FALSE	Homo sapi	284106	GO:000573	36891393	chr17	36891334	+		hs|17q12	NM_00113	chr17:3689	NM_00113	Hs.713595
A_23_P950	2.340517	1.226827	2.340517	up		-0.530786	-0.606981	-0.598504	0.712293	0.530786	0.701131	SNTB1	syntrophin,	FALSE	Homo sapi	6641	GO:004238	1.22E+08	chr8	1.22E+08	-		hs|8q24.12	NM_02102	chr8:12155	NM_02102	Hs.46701
A_22_P000	3.134383	1.648182	3.134383	up		-0.80363	-0.82853	-0.618605	1.030255	0.618605	1.04492	ADARB2-AS	ADARB2 ar	FALSE	Homo sapi	642394		1569022	chr10	1568963	+		hs|10p15.3	NR_03338	chr10:1568	NR_03338	Hs.568831
A_33_P341	3.348128	1.743355	3.348128	up		-0.852171	-0.797422	-1.03343	0.926658	0.822961	0.797422	KLF1	Kruppel-lik	FALSE	Homo sapi	10661	GO:000098	12995295	chr19	12995236	-		hs|19p13.2	NM_00656	chr19:1299	NM_00656	Hs.37860
A_24_P830	2.140283	1.097801	2.140283	up		-0.45679	-0.451631	-0.683656	0.582828	0.666868	0.451631	RPL21	ribosomal p	FALSE	Homo sapi	6144	GO:001046	27829379	chr13	27828361	+		hs|13q12.2	NM_00098	chr13:2782	NM_00098	Hs.381123
A_23_P948	18.31924	4.195288	18.31924	up		-2.064623	-2.169691	-2.039694	2.110188	2.039694	2.161972	ORM2	orosomucc	FALSE	Homo sapi	5005	GO:000681	1.17E+08	chr9	1.17E+08	+		hs|9q32	NM_00060	chr9:11709	NM_00060	Hs.719954
A_23_P137	3.144063	1.65263	3.144063	up		-0.726213	-0.880752	-0.996042	0.865933	0.762736	0.726213	NFE2	nuclear fac	FALSE	Homo sapi	4778	GO:000370	54686038	chr12	54685979	-		hs|12q13.1	NM_00616	chr12:5468	NM_00616	Hs.75643
A_21_P000	2.117269	1.082205	2.117269	up		-0.319406	-0.380956	-1.008386	0.319406	0.704295	0.514165	VTRNA2-1	vault RNA 2	FALSE	Homo sapi	1E+08		1.35E+08	chr5	1.35E+08	-		hs|5q31.1	NR_03058	chr5:13541	NR_030583	
A_23_P106	3.376595	1.755569	3.376595	up		-0.932139	-0.820223	-0.883922	0.820223	0.973052	0.837148	EMP2	epithelial n	FALSE	Homo sapi	2013	GO:000551	10626759	chr16	10626700	-		hs|16p13.1	NM_00142	chr16:1062	NM_00142	Hs.531561
A_32_P311	2.745036	1.456825	2.745036	up		-0.619473	-0.605693	-0.787306	0.846399	0.905911	0.605693	RPL7	ribosomal p	FALSE	Homo sapi	6129	GO:000551	74202885	chr8	74202845	-		hs|8q21.11	NM_00097	chr8:74202	NM_00097	Hs.571841
A_22_P000	4.908327	2.295231	4.908327	up		-0.982533	-1.166803	-0.978014	1.399768	0.978013	1.380564	LOC102031	uncharacte	FALSE	Homo sapi	1.02E+08		32636403	chr10	32636344	+		hs|10p11.2	NR_10416	chr10:3263	NR_10416	Hs.128132
A_33_P328	2.549192	1.35004	2.549192	up		-0.680594	-0.567785	-0.64381	0.730896	0.859252	0.567785	DPP4	dipeptidyl-	FALSE	Homo sapi	1803	GO:000551	1.63E+08	chr2	1.63E+08	-		hs|2q24.2	NM_00193	chr2:16284	NM_00193	Hs.368912
A_22_P000	3.586946	1.842756	3.586946	up		-0.820234	-0.975144	-1.078395	0.953332	0.88093	0.820234	lnc-ANKRD	lnc-ANKRD	FALSE	BX110621 Soares_testis_NHT Hom			71229756	chr2	71229697	+		hs|2p13.3	BX110621	chr2:71229697-71229		Hs.560906
A_23_P140	4.124239	2.044128	4.124239	up		-0.802781	-1.414511	-0.867781	1.054644	0.802781	1.189885	FOXB1	forkhead b	FALSE	Homo sapi	27023	GO:003350	60297967	chr15	60297908	+		hs|15q22.2	NM_01218	chr15:6029	NM_01218	Hs.734475
A_33_P329	2.027861	1.019959	2.027861	up		-0.483193	-0.536078	-0.470697	0.470697	0.61319	0.486021	WEE1	WEE1 G2 c	FALSE	Homo sapi	7465	GO:000551	9611132	chr11	9611073	+		hs|11p15.4	NM_00339	chr11:9611	NM_00339	Hs.249441
A_33_P322	2.696307	1.430985	2.696307	up		-0.855839	-0.479506	-0.586535	0.941566	0.479506	0.950004	EEF2KMT	eukaryotic	FALSE	Homo sapi	196483	GO:000551	5134584	chr16	5134526	-		hs|16p13.3	NM_20140	chr16:5134	NM_20140	Hs.406461
A_23_P150	2.424473	1.277671	2.424473	up		-0.608803	-0.69915	-0.628415	0.643415	0.608803	0.644429	C11orf1	chromoson	FALSE	Homo sapi	64776	GO:000815	1.12E+08	chr11	1.12E+08	+		hs|11q23.1	NM_02276	chr11:1117	NM_02276	Hs.17546
A_22_P000	3.81286	1.930874	3.81286	up		-0.75734	-1.04161	-0.880667	1.181657	0.75734	1.174007	PRRT3-AS1	PRRT3 anti	FALSE	Homo sapi	1.01E+08		9989288	chr3	9989229	+		hs|3p25.3	NR_04673	chr3:99892	NR_04673	Hs.616388
A_24_P200	2.899382	1.535745	2.899382	up		-0.439247	-0.982681	-0.801802	0.922845	0.439247	1.021415	STEAP3	STEAP fami	FALSE	Homo sapi	55240	GO:000551	1.2E+08	chr2	1.2E+08	+		hs|2q14.2	NM_18291	chr2:12001	NM_18291	Hs.647822
A_23_P314	2.302394	1.203135	2.302394	up		-0.49138	-0.49527	-0.556839	0.58283	0.991706	0.491381	STEAP1	six transme	FALSE	Homo sapi	26872	GO:000521	89794063	chr7	89794004	+		hs|7q21.13	NM_01244	chr7:89794	NM_01244	Hs.61635
A_21_P001	2.141774	1.098806	2.141774	up		-0.505767	-0.613691	-0.536424	0.595863	0.538905	0.505767			FALSE	family with sequence similarity 86			3952876	chr4	3948618	-		hs|4p16.3		chr4:3952876-3948618		Hs.591652
A_23_P118	2.288868	1.194634	2.288868	up		-0.507679	-0.720638	-0.508345	0.507679	0.755958	0.583602	HSD17B2	hydroxyste	FALSE	Homo sapi	3294	GO:000669	82131956	chr16	82131897	+		hs|16q23.3	NM_00215	chr16:8213	NM_00215	Hs.162795
A_22_P000	2.42196	1.276175	2.42196	up		-0.793602	-0.666091	-0.371666	0.798495	0.371666	0.827004	lnc-UNC93I	lnc-UNC93I	FALSE	Homo sapiens mRNA; cDNA DKFZp			67777412	chr11	67777353	-		hs|11q13.2	AL137634	chr11:67777412-67777		Hs.610985
A_21_P000	2.249702	1.169734	2.249702	up		-0.722541	-0.339072	-0.497474	0.721664	0.889378	0.339072	lnc-FOXO6-	lnc-FOXO6-	FALSE	LNCipedia lincRNA (lnc-FOXO6-1),			41930229	chr1	41898933	+		hs|1p34.2		chr1:41898933-41930229		
A_33_P330	2.330656	1.220736	2.330656	up		-0.792729	-0.474075	-0.694894	0.696929	0.474075	0.529507	MRGPRG	MAS-relate	FALSE	Homo sapi	386746	GO:000718	3239233	chr11	3239174	-		hs|11p15.4	NM_00116	chr11:3239	NM_00116	Hs.730306
A_23_P322	2.480891	1.310859	2.480891	up		-0.648138	-0.577427	-0.545475	0.545474	1.012678	0.603384	SQRDL	sulfide quir	FALSE	Homo sapi	58472	GO:003464	45983423	chr15	45983364	+		hs|15q21.1	NM_02119	chr15:4598	NM_02119	Hs.511251
A_23_P555	2.424282	1.277557	2.424282	up		-0.615365	-0.580066	-0.569385	0.714919	0.569385	0.783551	SMAD7	SMAD fami	FALSE	Homo sapi	4092	GO:000551	46446841	chr18	46446782	-		hs|18q21.1	NM_00590	chr18:4644	NM_00590	Hs.465087
A_33_P335	3.16488	1.662151	3.16488	up		-0.694065	-1.107781	-0.769738	0.882498	0.694065	0.838306	LINC01001	long interg	FALSE	Homo sapi	1E+08		128915	chr11	128856	-		hs|11p15.5	NR_02832	chr11:1289	NR_02832	Hs.645398
A_23_P345	3.042416	1.605217	3.042416	up		-0.739046	-0.520841	-0.657391	1.141217	0.520841	1.236317	EPHX1	epoxide hy	FALSE	Homo sapi	2052	GO:001407	2.26E+08	chr1	2.26E+08	+		hs|1q42.12	NM_00012	chr1:22603	NM_00012	Hs.89649
A_23_P558	2.402356	1.26445	2.402356	up		-0.530059	-0.46506	-0.509707	0.870145	0.46506	0.953319	AP1M1	adaptor-rel	FALSE	Homo sapi	8907	GO:000551	16345701	chr19	16345642	+		hs|19p13.1	NM_03249	chr19:1634	NM_03249	Hs.71040
A_23_P413	3.104741	1.634473	3.104741	up		-0.874824	-0.681023	-0.861477	0.681023	1.06472	0.740351	PREX1	phosphatid	FALSE	Homo sapi	57580	GO:000551	47241677	chr20	47241619	-		hs|20q13.1	NM_02082	chr20:4724	NM_02082	Hs.153310
A_22_P000	4.813716	2.267151	4.813716	up		-1.298996	-1.097103	-0.952492	1.358261	0.952492	1.142108	lnc-OLFML	lnc-OLFML	FALSE	Q8NE53_HUMAN (Q8NE53) Zinedi			1.28E+08	chr9	1.28E+08	+		hs|9q33.3		chr9:127533846-127533905		
A_23_P205	4.831312	2.272415	4.831312	up		-1.093053	-1.077596	-1.212429	1.108752	1.247818	1.077596	ALDH1A3	aldehyde d	FALSE	Homo sapi	220	GO:004306	1.01E+08	chr15	1.01E+08	+		hs|15q26.3	NM_00069	chr15:1014	NM_00069	Hs.459538
A_33_P331	2.714133	1.440491	2.714133	up		-0.781376	-0.684995	-0.678206	0.742097	0.756593	0.678206	GREM1	gremlin 1,	FALSE	Homo sapi	26585	GO:000551	33025901	chr15	33025842	+		hs|15q13.3	NM_00119	chr15:3302	NM_00119	Hs.40098
A_23_P792	2.128902	1.09011	2.128902	up		-0.399221	-0.461056	-0.45721	0.771824	0.399221	0.781798	SH3BP4	SH3-domai	FALSE	Homo sapi	23677	GO:004309	2.36E+08	chr2	2.36E+08	+		hs|2q37.2	NM_01452	chr2:23596	NM_01452	Hs.516777
A_33_P321	3.920056	1.970874	3.920056	up		-0.925908	-0.997486	-0.921362	1.151381	0.921362	0.995124	ZC3HAV1L	zinc finger	FALSE	Homo sapi	92092		1.39E+08	chr7	1.39E+08	-		hs|7q34	NM_08066	chr7:13872	NM_08066	Hs.659143
A_23_P128	2.284246	1.191718	2.284246	up		-0.50358	-0.516619	-0.753946	0.59643	0.700999	0.50358	ENOX1	ecto-NOX c	FALSE	Homo sapi	55068	GO:000588	43787918	chr13	43787859	-		hs|13q14.1	NM_01799	chr13:4378	NM_01799	Hs.128258
A_23_P395	2.782387	1.476323	2.782387	up		-0.650133	-0.593875	-0.540544	1.161273	0.540544	0.9426	C1QTNF1	C1q and tu	FALSE	Homo sapi	114897	GO:000551	77045229	chr17	77045170	+		hs|17q25.3	NM_19859	chr17:7704	NM_19859	Hs.201398
A_23_P103	6.290295	2.653128	6.290295	up		-1.311406	-1.278657	-1.242255	1.431499	1.242254	1.453313	HSPB7	heat shock	FALSE	Homo sapi	27129	GO:000698	16341448	chr1	16341389	-		hs|1p36.13	NM_01442	chr1:16341	NM_01442	Hs.502612
A_22_P000	5.055621	2.337888	5.055621	up		-0.836842	-1.37048	-1.312855	1.330362	0.836843	1.326284			FALSE				23584064	chr8	23584005	-		hs|8p21.2	XR_247144	chr8:23584	XR_247144	
A_33_P332	3.971931	1.98984	3.971931	up		-0.753301	-0.842938	-0.797751	1.442598	0.753301	1.379632			FALSE				38830315	chr2	38830256	+		hs|2p22.1		chr2:038830256-038830315		
A_33_P337	5.590805	2.483056	5.590805	up		-0.921036	-1.483933	-1.092668	1.553562	0.921036	1.476934			FALSE				79454868	chr17	79454809	-		hs|17q25.3		chr17:079454868-079454809		

A_22_P000	9.237214	3.207458	9.237214	up	-1.403975	-1.545552	-1.362352	1.990085	1.362352	1.958059	MMP25-AS	MMP25 an	FALSE	Homo sapi	1.01E+08		3108600	chr16	3108541	-	hs|16p13.3	NR_12372	chr16:3108	NR_123723	
A_23_P350	2.089774	1.063347	2.089774	up	-0.540393	-0.386806	-0.511898	0.618776	0.745364	0.386806	FCRLB	Fc receptor	FALSE	Homo sapi	127943	GO:000573	1.62E+08	chr1	1.62E+08	+	hs|1q23.3	NM_00100	chr1:1616	NM_00100	Hs.517422
A_33_P325	2.549178	1.350032	2.549178	up	-0.587387	-0.711732	-0.799439	0.734092	0.630061	0.587386	TYMP	thymidine	FALSE	Homo sapi	1890	GO:000903	50964244	chr22	50964185	-	hs|22q13.3	NM_00111	chr22:5096	NM_00111	Hs.180903
A_23_P105	2.333185	1.222301	2.333185	up	-0.579769	-0.475402	-0.633825	0.475402	0.979788	0.522717	ROM1	retinal oute	FALSE	Homo sapi	6094	GO:001046	62382563	chr11	62382504	+	hs|11q12.3	NM_00032	chr11:6238	NM_00032	Hs.281564
A_33_P334	3.615124	1.854045	3.615124	up	-0.727171	-1.360402	-0.897735	0.961671	0.727172	0.887984	CDC42EP5	CDC42 effe	FALSE	Homo sapi	148170	GO:000588	54976270	chr19	54976211	-	hs|19q13.4	NM_14505	chr19:5497	NM_14505	Hs.415791
A_21_P001	2.020475	1.014695	2.020475	up	-0.458482	-0.374176	-0.388803	0.641708	0.374176	0.806738	LOC10192	uncharacte	FALSE	PREDICTED	1.02E+08		44077063	chr20	44077004	+	hs|20q13.1	XR_244183	chr20:4407	XR_244183	Hs.729907
A_24_P183	2.169468	1.117341	2.169468	up	-0.568775	-0.610872	-0.533659	0.533659	0.541358	0.563701	BTF3	basic trans	FALSE	Homo sapi	689	GO:000551	72795018	chr5	72794959	+	hs|5q13.2	NM_00103	chr5:72794	NM_00103	Hs.591768
A_33_P331	2.336443	1.224314	2.336443	up	-0.911212	-0.402766	-0.698736	0.640426	0.402766	0.617036			FALSE				1.27E+08	chr10	1.27E+08	+	hs|10q26.13		chr10:127445429-127445488		
A_33_P329	3.036945	1.602621	3.036945	up	-1.083534	-0.923186	-0.623745	0.623745	0.694169	0.859485	LIPE	lipase, hor	FALSE	Homo sapi	3991	GO:004634	42905725	chr19	42905666	-	hs|19q13.2	NM_00535	chr19:4290	NM_00535	Hs.656980
A_23_P304	2.336438	1.224311	2.336438	up	-0.444371	-0.731336	-0.743452	0.444371	0.678866	0.630538	GATA6	GATA bindi	FALSE	Homo sapi	2627	GO:000551	19781852	chr18	19781793	+	hs|18q11.2	NM_00525	chr18:1978	NM_00525	Hs.514746
A_21_P000	2.917471	1.544719	2.917471	up	-0.866213	-0.863682	-0.630383	0.84496	0.798535	0.630383	lnc-MBIP-2	lnc-MBIP-2	FALSE	LNCipedia lincRNA (lnc-MBIP-2), li			36604106	chr14	36604047	-	hs|14q13.3		chr14:36604106-36604047		
A_33_P328	3.547175	1.82667	3.547175	up	-0.624685	-1.102125	-1.089056	1.105894	0.624685	0.933566	GLI4	GLI family	FALSE	GLI family	2738	GO:000815	1.44E+08	chr8	1.44E+08	+	hs|8q24.3	AB209654	chr8:144357115-144357174		
A_21_P000	5.371393	2.425296	5.371393	up	-1.238147	-1.115644	-1.381103	1.264252	1.161098	1.115644	SNORD12C	small nucle	FALSE	Homo sapi	26765		47895560	chr20	47895501	+	hs|20q13.1	NR_00243	chr20:4789	NR_00243	Hs.722986
A_21_P001	2.544815	1.347561	2.544815	up	-0.601186	-0.486488	-0.788346	0.486488	0.898325	0.781848	XLOC_l2_008270		FALSE	BROAD Institute lincRNA (XLOC_l2			1.32E+08	chr2	1.32E+08	-	hs|2q21.1		chr2:132384604-132384486		
A_21_P000	2.14102	1.098299	2.14102	up	-0.536505	-0.452147	-0.344072	0.707812	0.344072	0.910287	lnc-MCCC1	lnc-MCCC1	FALSE	AF292100 RP42 protein {Homo sap			1.83E+08	chr3	1.83E+08	-	hs|3q26.33		chr3:182703645-182698701		
A_22_P000	3.034979	1.601687	3.034979	up	-0.990631	-0.822966	-0.631711	0.831455	0.631711	0.896585	lnc-PHTF2-	lnc-PHTF2-	FALSE	LNCipedia lincRNA (lnc-PHTF2-1),			77620484	chr7	77620425	+	hs|7q21.11		chr7:77620425-77620		Hs.636411
A_33_P336	5.766184	2.527617	5.766184	up	-1.080714	-1.336644	-1.25658	1.48924	1.080714	1.338959			FALSE				41363168	chr4	41363109	-	hs|4p13		chr4:041363168-041363109		
A_33_P329	2.092127	1.064971	2.092127	up	-0.559969	-0.457624	-0.603718	0.457624	0.618912	0.497065	POLR2E	polymerase	FALSE	Homo sapi	5434	GO:000838	1088231	chr19	1088172	-	hs|19p13.3	NM_00269	chr19:1088	NM_00269	Hs.24301
A_33_P336	2.04259	1.0304	2.04259	up	-0.304312	-0.318181	-0.415247	0.847217	0.304312	0.901929	GPX3	glutathione	FALSE	Homo sapi	2878	GO:000460	1.5E+08	chr5	1.5E+08	+	hs|5q33.1	NM_00208	chr5:1504	NM_00208	Hs.386793
A_23_P250	3.916809	1.969679	3.916809	up	-0.961897	-0.935435	-0.941541	1.019992	0.935435	1.114736	FAM20C	family with	FALSE	Homo sapi	56975	GO:000579	300440	chr7	300381	+	hs|7p22.3	NM_02022	chr7:30038	NM_02022	Hs.134742
A_22_P000	3.198273	1.677293	3.198273	up	-0.612467	-0.743992	-0.976619	1.10379	0.612467	0.982544	lnc-ERLEC1	lnc-ERLEC1	FALSE	LNCipedia lincRNA (lnc-ERLEC1-1),			54087068	chr2	54087009	+	hs|2p16.2		chr2:54087009-54087068		
A_33_P324	3.010585	1.590044	3.010585	up	-0.575682	-0.99494	-0.742956	0.992515	0.575683	0.888357	ADAMTSL5	ADAMTS-lil	FALSE	Homo sapi	339366	GO:000650	1506798	chr19	1506739	-	hs|19p13.3	NM_21360	chr19:1506	NM_21360	Hs.371674
A_33_P322	2.828884	1.500233	2.828884	up	-0.495734	-0.734094	-1.210931	0.840295	0.495734	0.723913	FLJ44477	FLJ44477 p	FALSE	Homo sapi	401157		1.44E+08	chr4	1.44E+08	+	hs|4q31.21	AK126441	chr4:143768284-14376		Hs.531403
A_33_P332	3.061274	1.614132	3.061274	up	-0.714164	-0.99313	-0.670128	0.861235	0.670129	0.93361	DNM1	dynamin 1	FALSE	Homo sapi	1759	GO:007258	1.31E+08	chr9	1.31E+08	+	hs|9q34.11	NM_00128	chr9:13101	NM_00128	Hs.522413
A_33_P324	4.441054	2.150902	4.441054	up	-1.262012	-1.053165	-0.774772	1.155958	0.774772	1.432027	ZNF329	zinc finger	FALSE	zinc finger	79673	GO:000635	58666162	chr19	58666103	-	hs|19q13.4	AK090893	chr19:58666162-5866		Hs.458377
A_32_P164	4.471973	2.160912	4.471973	up	-1.013032	-1.008453	-1.086718	1.015687	1.35039	1.008453	FOXQ1	forkhead b	FALSE	Homo sapi	94234	GO:004356	1314688	chr6	1314629	+	hs|6p25.3	NM_03326	chr6:1314	NM_03326	Hs.591352
A_33_P322	5.86987	2.553329	5.86987	up	-1.261016	-1.239572	-1.022919	1.386972	1.022919	1.726588	SBNO2	strawberry	FALSE	Homo sapi	22904	GO:007267	1108327	chr19	1108268	-	hs|19p13.3	NM_01496	chr19:1108	NM_01496	Hs.408708
A_22_P000	2.519414	1.333089	2.519414	up	-0.657961	-0.764388	-0.542362	0.693156	0.542363	0.799035	lnc-TPSAB1	lnc-TPSAB1	FALSE	LNCipedia lincRNA (lnc-TPSAB1-1),			1308075	chr16	1308016	+	hs|16p13.3		chr16:1308016-1308075		
A_33_P325	3.451422	1.787191	3.451422	up	-0.887326	-0.865349	-0.852207	0.960646	0.852207	0.943838	PDIA5	protein dis	FALSE	Homo sapi	10954	GO:003497	1.23E+08	chr3	1.23E+08	+	hs|3q21.1	NM_00681	chr3:12282	NM_00681	Hs.477352
A_22_P000	2.544635	1.347459	2.544635	up	-0.839072	-0.65173	-0.593684	0.616648	0.593683	0.74756	lnc-C9orf1(	lnc-C9orf1(	FALSE	Q5T6J5_HUMAN (Q5T6J5) Ubiquili			86322625	chr9	86322566	+	hs|9q21.32		chr9:86322566-86322625		
A_22_P000	2.291243	1.196131	2.291243	up	-0.798629	-0.481291	-0.354383	0.859207	0.354383	0.740498			FALSE	K-EST0130195 S13KMS5s1 Homo s			50864715	chr14	50864656	+	hs|14q21.3	BQ082534	chr14:50864656-50864		Hs.619827
A_19_P008	2.439652	1.286675	2.439652	up	-0.535338	-0.754241	-0.699157	0.774393	0.535338	0.561559	SEPT7-AS1	SEPT7 antis	FALSE	SEPT7 antis	1.02E+08		35792332	chr7	35792273	-	hs|7p14.2		chr7:35792332-35792273		
A_23_P575	2.006172	1.004445	2.006172	up	-0.47136	-0.415313	-0.438282	0.599082	0.415313	0.673985	EIF3L	eukaryotic	FALSE	Homo sapi	51386	GO:000551	38282789	chr22	38274137	+	hs|22q13.1	NM_01609	chr22:3827	NM_01609	Hs.446852
A_21_P000	2.772139	1.471	2.772139	up	-0.88424	-0.644883	-0.619949	0.619949	0.757905	0.886072	SNORD35A	small nucle	FALSE	Homo sapi	26816		49994510	chr19	49994451	+	hs|19q13.3	NR_00001	chr19:4999	NR_00001	Hs.744801
A_24_P664	2.256309	1.173964	2.256309	up	-0.566471	-0.569928	-0.629894	0.60208	0.587049	0.566471	CBX5	chromobox	FALSE	Homo sapi	23468	GO:000551	54624854	chr12	54624795	-	hs|12q13.1	NM_00112	chr12:5462	NM_00112	Hs.349283
A_21_P001	3.488868	1.802759	3.488868	up	-0.747099	-0.879022	-1.033749	0.747099	1.187763	0.813545	LOC10272	uncharacte	FALSE		1.03E+08		2.23E+08	chr2	2.23E+08	+	hs|2q36.1	XR_425533	chr2:2231	XR_425533	Hs.732732
A_23_P203	2.347541	1.231151	2.347541	up	-0.57841	-0.497978	-0.745214	0.587717	0.786156	0.497978	PSD3	pleckstrin	FALSE	Homo sapi	23362	GO:004354	18385112	chr8	18385053	-	hs|8p22	NM_01531	chr8:18385	NM_01531	Hs.434255
A_21_P000	2.353061	1.234539	2.353061	up	-0.68156	-0.716636	-0.511062	0.511062	0.61154	0.671757	lnc-APLN-1	lnc-APLN-1	FALSE	LNCipedia lincRNA (lnc-APLN-1), li			1.29E+08	chrX	1.29E+08	-	hs|Xq26.1		chrX:128812135-128809966		
A_33_P341	2.200988	1.138151	2.200988	up	-0.76183	-0.493782	-0.326305	0.66596	0.326305	0.840272	ZSCAN2	zinc finger	FALSE	Homo sapi	54993	GO:000635	85166387	chr15	85166329	+	hs|15q25.2	NM_18187	chr15:8516	NM_18187	Hs.594023
A_24_P217	4.230978	2.080991	4.230978	up	-1.229556	-0.956934	-0.943271	1.094644	0.943271	1.075297	EDNRA	endothelin	FALSE	Homo sapi	1909	GO:000551	1.48E+08	chr4	1.48E+08	+	hs|4q31.22	NM_00195	chr4:14846	NM_00195	Hs.183713
A_22_P000	3.083988	1.624797	3.083988	up	-0.454323	-0.801098	-0.624884	1.421783	0.454323	1.117982	lnc-RP11-1	lnc-RP11-1	FALSE	LNCipedia lincRNA (lnc-RP11-166B			12070767	chr16	12070708	-	hs|16p13.13		chr16:12070767-12070708		
A_24_P902	2.572414	1.363123	2.572414	up	-0.723942	-0.621965	-0.716216	0.663424	0.621965	0.741857	DANCR	differentia	FALSE	Homo sapi	57291		53579768	chr4	53579709	+	hs|4q12	NR_02403	chr4:53579	NR_02403	Hs.744077
A_22_P000	3.19184	1.674388	3.19184	up	-0.754882	-0.741098	-0.670514	1.036565	0.670513	1.149592			FALSE				51301739	chr20	51301680	+	hs|20q13.2		chr20:51301680-51301		Hs.738179
A_33_P323	3.929243	1.974252	3.929243	up	-0.99176	-0.874827	-1.072556	0.95751	0.874827	1.151275	LOC10013	uncharacte	FALSE	Homo sapi	1E+08		37447245	chr21	37447186	-	hs|21q22.1	NR_04008	chr21:3744	NR_04008	Hs.728809
A_22_P000	8.165615	3.029562	8.165615	up	-1.49505	-1.536432	-1.548768	1.496229	1.49505	1.517157	lnc-PLEKHG	lnc-PLEKHG	FALSE	nm91g12.s1 NCI_CGAP_Co9 Homo			6438101	chr12	6438042	+	hs|12p13.3	AA577485	chr12:6438042-643810		Hs.732532
A_24_P384	4.470854	2.160551	4.470854	up	-0.898373	-1.312501	-0.801327	1.232572	0.801328	1.435552	RAVER1	ribonucleo	FALSE	Homo sapi	125950	GO:000573	10431850	chr19	10431791	-	hs|19p13.2	NM_13345	chr19:1043	NM_13345	Hs.744952
A_33_P339	2.650218	1.406111	2.650218	up	-0.982948	-0.633572	-0.498911	0.682319	0.498911	0.921671	MGA	MGA, MAX	FALSE	MGA, MAX	23269	GO:000566	42063153	chr15	42063094	+	hs|15q15.1	AF176922	chr15:42063094-42063		Hs.187569
A_33_P682	2.166947	1.115664	2.166947	up	-0.399225	-0.716852	-0.534571	0.591435	0.399225	0.705683	PDCD4-AS1	PDCD4 anti	FALSE	Homo sapi	282997		1.13E+08	chr10	1.13E+08	+	hs|10q25.2	NR_02693	chr10:1126	NR_02693	Hs.599931
A_22_P000	4.432296	2.148054	4.432296	up	-0.95329	-1.300551	-0.70879	1.177112	0.708791	1.59563			FALSE				74952117	chr11	74952058	-	hs|11q13.4		chr11:74952117-74952058		
A_22_P000	11.56853	3.532134	11.56853	up	-1.563568	-2.080443	-1.915669	1.73991	1.563568	1.733244			FALSE				1.31E+08	chr12	1.31E+08	+	hs|12q24.33		chr12:131143292-131143351		
A_33_P327	2.195851	1.13478	2.195851	up	-0.506038	-0.50395	-0.590517	0.50395	0.626019	0.673867	LTK	leukocyte r	FALSE	leukocyte r	4058	GO:004306	41799749	chr15	41799690	-	hs|15q15.1	BC045607	chr15:41799749-41799690		
A_22_P000	2.226359	1.154686	2.226359	up	-0.515915	-0.557393	-0.439719	0.741819	0.439719	0.769492	lnc-SYT13-2	lnc-SYT13-2	FALSE	LNCipedia lincRNA (lnc-SYT13-2), li			45168253	chr11	45168194	-	hs|11p11.2		chr11:45168253-45168194		
A_24_P108	5.13003	2.358967	5.13003	up	-1.062875	-1.185452	-0.933996	1.447026	0.933996	1.513558	SCARF2	scavenger	FALSE	Homo sapi	91179	GO:000551	20780247	chr22	20780188	-	hs|22q11.2	NM_15333	chr22:2078	NM_15333	Hs.474251
A_23_P160	2.019848	1.014247	2.019848	up	-0.536153	-0.447806	-0.576966	0.447806	0.557352	0.476656	CDC42EP2	CDC42 effe	FALSE	Homo sapi	10435	GO:000551	65089805	chr11	65089746	+	hs|11q13.1	NM_00677	chr11:6508	NM_00677	Hs.343380
A_21_P001	2.436609	1.284874	2.436609	up	-0.824783	-0.773095	-0.166174	0.166174	1.153507	0.770889			FALSE	HUMFNC fibronectin {Homo sapie			2.16E+08	chr2	2.16E+08	+	hs|2q35		chr2:216226694-216226753		
A_22_P000	7.776848	2.959186	7.776848	up	-1.229689	-1.68096	-1.378602	1.744997	1.229689	1.61362	SLC12A5	solute carri	FALSE	Homo sapi	57468	GO:000588	44650525	chr20	44650466	+	hs|20q13.1	NM_00113	chr20:4465	NM_00113	Hs.21413
A_33_P336	3.426723	1.77683	3.426723	up	-0.722103	-1.095582	-0.724517	1.01397	0.722102	1.052215	TMEM238	transmemb	FALSE	Homo sapi	388564	GO:001602	55890672	chr19	55890613	-	hs|19q13.4	NM_00119	chr19:5589	NM_00119	Hs.534672
A_21_P000	2.005575	1.004016	2.005575	up	-0.46027	-0.421947	-0.466391	0.46023	0.781262	0.421947	MT1E	metallothio	FALSE	Homo sapi	4493	GO:007129	56660942	chr16	56660883	+	hs|16q12.2	NM_17561	chr16:5666	NM_17561	Hs.744893

A_23_P213	3.952256	1.982677	3.952256	up	-0.989065	-1.016666	-0.926891	0.926891	1.107129	0.981388	TWIST2	twist family	FALSE	Homo sapi	117581	GO:000551	2.4E+08	chr2	2.4E+08	+	hs|2q37.3	NM_00127	chr2:23983	NM_00127	Hs.422585
A_33_P339	14.31193	3.839147	14.31193	up	-1.604004	-2.225722	-1.866883	2.32225	1.604004	1.894577	KCNQ2	potassium	FALSE	Homo sapi	3785	GO:000525	62065157	chr20	62065098	-	hs|20q13.3	NM_17210	chr20:6206	NM_17210	Hs.161851
A_23_P396	2.053604	1.038158	2.053604	up	-0.262466	-0.476462	-0.533504	0.779803	0.262466	0.799774	NCOA1	nuclear rec	FALSE	Homo sapi	8648	GO:000551	24980964	chr2	24980905	+	hs|2p23.3	NM_14723	chr2:2498C	NM_14723	Hs.596314
A_23_P216	2.234062	1.159669	2.234062	up	-0.607887	-0.54064	-0.551731	0.568659	0.54064	0.669452	SVEP1	sushi, von \	FALSE	Homo sapi	79987	GO:000573	1.13E+08	chr9	1.13E+08	-	hs|9q31.3	NM_15336	chr9:11313	NM_15336	Hs.522334
A_33_P325	5.826862	2.542719	5.826862	up	-1.259896	-0.996456	-1.296448	1.503698	0.996456	1.575205	SP8	Sp8 transcr	FALSE	Homo sapi	221833	GO:000635	20823968	chr7	20823909	-	hs|7p21.1	NM_19895	chr7:20823	NM_19895	Hs.195922
A_21_P000	2.821715	1.496572	2.821715	up	-0.825674	-0.612006	-0.872364	0.762132	0.612006	0.805535	SNORD110	small nucle	FALSE	Homo sapi	692213		2634928	chr20	2634869	+	hs|20p13	NR_00307	chr20:2634	NR_003078	
A_23_P256	2.012569	1.009039	2.012569	up	-0.423893	-0.592174	-0.627142	0.447096	0.423893	0.512917	ARSE	arylsulfatas	FALSE	Homo sapi	415	GO:000666	2854805	chrX	2853195	-	hs|Xp22.33	NM_00004	chrX:28548	NM_00004	Hs.386975
A_33_P337	2.323461	1.216276	2.323461	up	-0.568921	-0.615918	-0.585783	0.610007	0.699277	0.568921			FALSE				4608351	chr17	4608292	-	hs|17p13.2		chr17:4608351-4608292		
A_33_P341	2.617284	1.388071	2.617284	up	-0.539299	-1.08725	-0.654063	0.539299	0.555576	0.788724	NPB	neuropepti	FALSE	Homo sapi	256933	GO:000551	79860776	chr17	79860717	+	hs|17q25.3	NM_14889	chr17:7986	NM_14889	Hs.708916
A_24_P203	2.034667	1.024793	2.034667	up	-0.601861	-0.336669	-0.49457	0.706713	0.336669	0.597896	TPRXL	tetra-pepti	FALSE	Homo sapi	348825		14107369	chr3	14107310	+	hs|3p25.1	NR_00222	chr3:14107	NR_00222	Hs.529180
A_23_P374	3.022825	1.595897	3.022825	up	-0.860124	-0.729484	-0.75501	0.843596	0.729484	0.869994	LACC1	laccase (mu	FALSE	Homo sapi	144811		44464390	chr13	44464331	+	hs|13q14.1	NM_15321	chr13:4446	NM_15321	Hs.210586
A_23_P864	2.963441	1.567273	2.963441	up	-0.654248	-0.969283	-0.756887	0.849219	0.654247	0.817935	LBX1	ladybird hc	FALSE	Homo sapi	10660	GO:000566	1.03E+08	chr10	1.03E+08	-	hs|10q24.3	NM_00656	chr10:1029	NM_00656	Hs.37128
A_33_P339	8.47238	3.082767	8.47238	up	-1.273468	-1.405176	-1.42239	1.875482	1.273468	1.998319			FALSE				208989	chr7	208930	+	hs|7p22.3		chr7:000208930-000208989		
A_23_P129	2.067802	1.048098	2.067802	up	-0.388074	-0.554339	-0.424393	0.665962	0.388074	0.723453	ZNF689	zinc finger	FALSE	Homo sapi	115509	GO:000635	30615108	chr16	30615049	-	hs|16p11.2	NM_13844	chr16:3061	NM_13844	Hs.454685
A_24_P152	2.251308	1.170764	2.251308	up	-0.348098	-0.583265	-0.700384	0.781039	0.348098	0.751406	VAT1	vesicle ami	FALSE	Homo sapi	10493	GO:001063	41170689	chr17	41170630	-	hs|17q21.3	NM_00637	chr17:4117	NM_00637	Hs.514199
A_23_P214	2.04634	1.033046	2.04634	up	-0.507449	-0.492282	-0.441711	0.465764	0.750221	0.44171	AKAP12	A kinase (P	FALSE	Homo sapi	9590	GO:000551	1.52E+08	chr6	1.52E+08	+	hs|6q25.1	NM_14449	chr6:15167	NM_14449	Hs.371240
A_23_P241	2.486596	1.314172	2.486596	up	-0.427686	-0.537994	-0.799331	0.692452	0.427686	1.057367	TACR2	tachykinin	FALSE	Homo sapi	6865	GO:000551	71164785	chr10	71164726	-	hs|10q22.1	NM_00105	chr10:7116	NM_00105	Hs.88372
A_33_P340	8.210474	3.037466	8.210474	up	-1.315413	-1.230189	-1.568429	2.010618	1.230189	1.75756	LINC00176	long interg	FALSE	Homo sapi	284739		62671131	chr20	62671072	+	hs|20q13.3	NR_02768	chr20:6267	NR_02768	Hs.97840
A_23_P142	2.877106	1.524619	2.877106	up	-0.770682	-0.652792	-0.765162	0.652792	1.032612	0.699816	ACP5	acid phospl	FALSE	Homo sapi	54	GO:005083	11685584	chr19	11685525	-	hs|19p13.2	NM_00161	chr19:1168	NM_00161	Hs.1211
A_23_P865	2.143097	1.099697	2.143097	up	-0.355338	-0.357327	-0.868495	0.799346	0.355338	0.563247	BICC1	BicC family	FALSE	BicC family	80114	GO:000573	60573839	chr10	60573780	+	hs|10q21.1	AK026129	chr10:60573780-60573		Hs.158745
A_32_P693	3.918432	1.970277	3.918432	up	-0.885362	-0.979682	-0.999152	0.958767	1.202505	0.885362	ID2	inhibitor of	FALSE	Homo sapi	3398	GO:000165	8824341	chr2	8824282	+	hs|2p25.1	NM_00216	chr2:88242	NM_00216	Hs.180919
A_33_P326	5.216896	2.383192	5.216896	up	-1.120515	-1.189795	-0.984596	1.315373	0.984596	1.554701	MEX3D	mex-3 RNA	FALSE	Homo sapi	399664	GO:006115	1555636	chr19	1555577	-	hs|19p13.3	NM_00117	chr19:1555	NM_00117	Hs.436495
A_22_P000	3.015988	1.592631	3.015988	up	-0.681231	-0.741292	-0.753835	0.965421	0.681231	0.954883	LOC100507	uncharacte	FALSE	Homo sapi	1.01E+08		1.44E+08	chr8	1.44E+08	-	hs|8q24.3	NR_12068	chr8:14436	NR_12068	Hs.446671
A_33_P338	2.586968	1.371262	2.586968	up	-0.920117	-0.723156	-0.473589	0.65931	0.473589	0.864026	MATN1-AS	MATN1 ant	FALSE	Homo sapi	1E+08		31194140	chr1	31192359	+	hs|1p35.2	NR_03418	chr1:31192	NR_03418	Hs.659751
A_33_P338	6.004247	2.585983	6.004247	up	-1.250567	-1.42608	-1.017329	1.510078	1.017329	1.536567	LKAAEAR1	LKAAEAR m	FALSE	Homo sapi	198437		62714996	chr20	62714937	-	hs|20q13.3	NM_00100	chr20:6271	NM_00100	Hs.570316
A_23_P167	3.457706	1.789815	3.457706	up	-0.846365	-0.925881	-0.912176	0.846365	0.978579	0.86008	PDIA5	protein dis	FALSE	Homo sapi	10954	GO:003497	1.23E+08	chr3	1.23E+08	+	hs|3q21.1	NM_00681	chr3:12288	NM_00681	Hs.477352
A_33_P328	2.591777	1.373941	2.591777	up	-0.583154	-0.82233	-0.460314	0.8362	0.460314	0.959513			FALSE	Homo sapiens mRNA; cDNA DKFZp			48087083	chr18	48087024	+	hs|18q21.1	BX648603	chr18:48087024-48087		Hs.433728
A_33_P340	2.319404	1.213754	2.319404	up	-0.578516	-0.51093	-0.588241	0.648919	0.51093	0.803729	LETM1	leucine zip	FALSE	Homo sapi	3954	GO:000573	45600828	chr14	45600769	-	hs|14q21.2	NM_01231	chr14:0456	NM_01231	Hs.120165
A_23_P752	7.101588	2.828142	7.101588	up	-1.320288	-1.671747	-1.469042	1.354147	1.348913	1.320288	RBP4	retinol binc	FALSE	Homo sapi	5950	GO:000551	95351728	chr10	95351669	-	hs|10q23.3	NM_00674	chr10:9535	NM_00674	Hs.50223
A_23_P207	2.247707	1.168454	2.247707	up	-0.582897	-0.538461	-0.625435	0.569911	0.650197	0.538461	SOCS3	suppressor	FALSE	Homo sapi	9021	GO:000551	76353186	chr17	76353127	-	hs|17q25.3	NM_00395	chr17:7635	NM_00395	Hs.527973
A_23_P133	2.040654	1.029032	2.040654	up	-0.32774	-0.348635	-0.365195	0.823408	0.327741	0.894376	GPX3	glutathione	FALSE	Homo sapi	2878	GO:000460	1.5E+08	chr5	1.5E+08	+	hs|5q33.1	NM_00208	chr5:1504C	NM_00208	Hs.386793
A_33_P336	3.022981	1.595972	3.022981	up	-0.651673	-0.794562	-0.72081	0.96415	0.651673	1.005049	CYP24A1	cytochrom	FALSE	Homo sapi	1591	GO:000680	52773988	chr20	52773929	-	hs|20q13.2	NM_00078	chr20:5277	NM_00078	Hs.89663
A_22_P000	3.260969	1.705301	3.260969	up	-0.757556	-0.917648	-0.898485	0.885854	0.757556	0.898803	YTHDF3	YTH N(6)-m	FALSE	Homo sapi	253943	GO:000551	64081460	chr8	64081401	+	hs|8q12.3	NM_00127	chr8:64081	NM_00127	Hs.491861
A_33_P338	2.741097	1.454753	2.741097	up	-0.848972	-0.703974	-0.621769	0.822651	0.621769	0.745125	HLA-DRB3	major histc	FALSE	Homo sapi	3125	GO:0005515|GO:0002437|GO:0050852|GO:0005886|GO:0019221|G						NM_022555		NM_02255	Hs.696211
A_21_P000	2.076939	1.054459	2.076939	up	-0.438221	-0.656034	-0.629941	0.438221	0.443817	0.557142	SNORD96A	small nucle	FALSE	Homo sapi	619571		1.81E+08	chr5	1.81E+08	-	hs|5q35.3	NR_00259	chr5:18066	NR_002592	
A_33_P329	3.311662	1.727555	3.311662	up	-0.914529	-0.973206	-0.80458	0.80458	0.866321	0.819449	CCDC172	coiled-coil	FALSE	Homo sapi	374355		1.18E+08	chr10	1.18E+08	+	hs|10q25.3	NM_19851	chr10:1181	NM_19851	Hs.233407
A_33_P326	3.023462	1.596201	3.023462	up	-0.864677	-0.775366	-0.379766	1.309923	0.379766	1.079108	AIF1L	allograft in	FALSE	Homo sapi	83543	GO:000573	1.34E+08	chr9	1.34E+08	+	hs|9q34.12	NM_00118	chr9:13399	NM_00118	Hs.4944
A_22_P000	3.732403	1.900105	3.732403	up	-0.976944	-0.946263	-0.905957	0.905956	0.958589	1.006605	lnc-GGCT-1	lnc-GGCT-1	FALSE	Homo sapiens mRNA; cDNA DKFZp			30625181	chr7	30625122	-	hs|7p14.3	AL137445	chr7:30625181-306251		Hs.561708
A_21_P000	3.440794	1.782742	3.440794	up	-0.778354	-0.86907	-0.786715	1.091243	0.778354	1.04449	lnc-FBLN2-	lnc-FBLN2-	FALSE	LNCipedia lincRNA (lnc-FBLN2-1), l			13759231	chr3	13759172	+	hs|3p25.1		chr3:13759172-13759231		
A_23_P156	2.74054	1.45446	2.74054	up	-0.7323	-0.707186	-0.712256	0.7121	0.792352	0.707187	TGFBI	transformi	FALSE	Homo sapi	7045	GO:000551	1.35E+08	chr5	1.35E+08	+	hs|5q31.1	NM_00035	chr5:13539	NM_00035	Hs.369397
A_22_P000	3.914185	1.968712	3.914185	up	-0.834704	-0.9559	-0.596253	1.381037	0.596253	1.54199	lnc-TRAPPC	lnc-TRAPPC	FALSE	LNCipedia lincRNA (lnc-TRAPPC12-			2876363	chr2	2876304	+	hs|2p25.3		chr2:2876304-2876363		
A_22_P000	2.658763	1.410755	2.658763	up	-0.744237	-0.917405	-0.50733	0.693564	0.862397	0.50733			FALSE	Homo sapiens cDNA FLJ38525 fis,			30001807	chr11	30001748	-	hs|11p14.1	AK095844	chr11:30001807-30001		Hs.128812
A_23_P848	2.231031	1.157711	2.231031	up	-0.587682	-0.547465	-0.585636	0.547465	0.614953	0.589932	FAM107A	family with	FALSE	Homo sapi	11170	GO:004300	58550152	chr3	58550093	-	hs|3p14.3	NM_00717	chr3:5855C	NM_00717	Hs.506357
A_21_P000	2.224555	1.153517	2.224555	up	-0.786772	-0.520127	-0.435827	0.449081	0.832918	0.435827	SNORD33	small nucle	FALSE	Homo sapi	26818		49993956	chr19	49993897	+	hs|19q13.3	NR_00002	chr19:4999	NR_000020	
A_22_P000	3.954938	1.983655	3.954938	up	-0.801144	-0.920629	-0.796981	1.280342	0.796982	1.354887	LOC72873C	uncharacte	FALSE	Homo sapi	728730		39664676	chr2	39664617	+	hs|2p22.1	NR_03787	chr2:39664	NR_03787	Hs.655344
A_19_P003	2.257395	1.174659	2.257395	up	-0.29772	-0.570857	-0.674116	0.775615	0.29772	0.907949	LOC10192	uncharacte	FALSE		1.02E+08		77899201	chr17	77899142	+	hs|17q25.3		chr17:77899142-77899		Hs.629500
A_33_P332	2.125199	1.087598	2.125199	up	-0.439979	-0.634914	-0.615762	0.613702	0.43998	0.518456	IP6K1	inositol hex	FALSE	Homo sapi	9807	GO:004685	49761787	chr3	49761728	-	hs|3p21.31	NM_00124	chr3:49761	NM_00124	Hs.386168
A_23_P379	2.351216	1.233407	2.351216	up	-0.556025	-0.551018	-0.619946	0.637097	0.785118	0.551017	MT1B	metallothic	FALSE	Homo sapi	4490	GO:007129	56687007	chr16	56686948	+	hs|16q12.2	NM_00594	chr16:5668	NM_00594	Hs.656629
A_24_P178	4.853763	2.279104	4.853763	up	-1.174707	-1.068123	-1.273356	1.129293	1.068123	1.12371	GGT1	gamma-glu	FALSE	Homo sapi	2678	GO:001936	25023930	chr22	25023871	+	hs|22q11.2	NM_00128	chr22:2502	NM_00128	Hs.645535
A_33_P342	2.062431	1.044346	2.062431	up	-0.810422	-0.504728	-0.31778	0.515275	0.31778	0.667053	RAPH1	Ras associa	FALSE	Homo sapi	65059	GO:000551	2.04E+08	chr2	2.04E+08	-	hs|2q33.2	NM_21358	chr2:2043C	NM_21358	Hs.471162
A_33_P336	2.286133	1.192909	2.286133	up	-0.794273	-0.754316	-0.294592	0.569861	0.294592	0.871094			FALSE	ICP0_HHV11 (P08393) Trans-acting			37899792	chr2	37899733	+	hs|2p22.2		chr2:037899733-037899792		
A_33_P333	2.319547	1.213843	2.319547	up	-0.871077	-0.334728	-0.655718	0.716263	0.334728	0.729015	CDC42EP3	CDC42 effe	FALSE	Homo sapi	10602	GO:000551	37870802	chr2	37870743	-	hs|2p22.2	NM_00644	chr2:3787C	NM_00644	Hs.369574
A_23_P285	5.291755	2.403746	5.291755	up	-1.156564	-1.36384	-1.099596	1.159009	1.332633	1.099596	DLX2	distal-less l	FALSE	Homo sapi	1746	GO:004574	1.73E+08	chr2	1.73E+08	-	hs|2q31.1	NM_00440	chr2:17296	NM_00440	Hs.419
A_24_P724	2.47323	1.306396	2.47323	up	-0.826742	-0.413763	-0.707242	0.615898	0.413763	0.94178			FALSE	Synthetic construct Homo sapiens			1.52E+08	chr4	1.52E+08	-	hs|4q31.3	CU688199	chr4:152025742-15202		Hs.606561
A_33_P339	2.04508	1.032158	2.04508	up	-0.446006	-0.366952	-0.447815	0.507301	0.366952	0.961447			FALSE	DA197111 BRASW1 Homo sapiens			1.32E+08	chr2	1.32E+08	+	hs|2q21.1	DA197111	chr2:132121559-13212		Hs.712140
A_23_P548	3.049544	1.608593	3.049544	up	-0.831808	-0.75933	-0.756313	0.756314	0.898881	0.823133	MT1A	metallothic	FALSE	Homo sapi	4489	GO:007129	56673808	chr16	56673205	+	hs|16q12.2	NM_00594	chr16:5667	NM_00594	Hs.655199
A_33_P339	2.717226	1.442135	2.717226	up	-0.435385	-0.796448	-0.813463	1.083803	0.435385	0.76192			FALSE	GB			22707640	chr22	22707581	+	hs|22q11.22		chr22:022707581-022707640		
A_33_P321	3.316725	1.729759	3.316725	up	-0.889066	-0.92658	-0.59363	0.896795	0.59363	1.289577	PNPLA2	patatin-like	FALSE	Homo sapi	57104	GO:000581	824823	chr11	824764	+	hs|11p15.5	NM_02037	chr11:8247	NM_02037	Hs.654697

A_23_P333	3.811935	1.930523	3.811935	up	-0.823545	-1.064934	-0.960879	1.050358	0.823545	1.06831			FALSE	double homeobox 4 like 11 [Sourc			73992	chrUn_gl00	73925	+		XM_00671	chrUn_gl00	XM_00671	Hs.725918
A_22_P000	5.68228	2.50647	5.68228	up	-1.1392	-1.313915	-1.083771	1.412051	1.083771	1.486702	lnc-CADPS-	lnc-CADPS-	FALSE	LNCipedia lincRNA (lnc-CADPS-1),			63265024	chr3	63264965	-	hs|3p14.2		chr3:63265024-63264965		
A_33_P340	2.195162	1.134327	2.195162	up	-0.493899	-0.697733	-0.413229	0.584134	0.413229	0.800756	VAMP2	vesicle-ass	FALSE	Homo sapi	6844	GO:000551	8063853	chr17	8063794	-	hs|17p13.1	NM_01423	chr17:8063	NM_01423	Hs.25348
A_21_P001	2.143952	1.100273	2.143952	up	-0.478491	-0.412089	-0.521316	0.412089	0.952253	0.525481			FALSE	AV738989 CB Homo sapiens cDNA			22418370	chr1	22418311	-	hs|1p36.12	AV738989	chr1:22418370-224183		Hs.612535
A_22_P000	2.92101	1.546467	2.92101	up	-0.61126	-0.898915	-0.753739	0.862523	0.61126	0.901704			FALSE				30449613	chr12	30449554	-	hs|12p11.2	DB066917	chr12:30449613-30449		Hs.569114
A_23_P144	2.07725	1.054675	2.07725	up	-0.274497	-0.889478	-0.846768	0.391531	0.487254	0.274497	GFPT2	glutamine-	FALSE	Homo sapi	9945	GO:001605	1.8E+08	chr5	1.8E+08	-	hs|5q35.3	NM_00511	chr5:17972	NM_00511	Hs.696497
A_24_P150	3.17182	1.665311	3.17182	up	-0.98671	-0.683552	-0.666824	0.670039	1.321983	0.666824	KCNA4	potassium	FALSE	Homo sapi	3739	GO:000525	30031851	chr11	30031792	-	hs|11p14.1	NM_00223	chr11:3003	NM_00223	Hs.592002
A_33_P323	3.027391	1.598075	3.027391	up	-0.63932	-0.654411	-0.832248	1.032012	0.63932	0.996915	HDGF	hepatoma-	FALSE	Homo sapi	3068	GO:000828	1.57E+08	chr1	1.57E+08	-	hs|1q23.1	NM_00449	chr1:15671	NM_00449	Hs.743948
A_33_P323	2.435315	1.284109	2.435315	up	-0.408816	-0.504126	-0.542095	0.918268	0.408816	1.070205	EEF2KMT	eukaryotic	FALSE	Homo sapi	196483	GO:000551	5134996	chr16	5134937	-	hs|16p13.3	NM_20140	chr16:5134	NM_20140	Hs.406461
A_21_P001	8.824483	3.141512	8.824483	up	-1.684571	-1.432518	-1.33887	1.33887	2.042531	1.587175			FALSE	HNC45-1-G12.R HNC (Human Norr			1.32E+08	chr6	1.32E+08	+	hs|6q23.2	BG927974	chr6:132269645-1322		Hs.720521
A_22_P000	3.257182	1.703625	3.257182	up	-0.879351	-0.899663	-0.732179	0.930152	0.732179	0.93735	LOC646762	uncharacte	FALSE	zinc and rir	646762		29638504	chr7	29638445	-	hs|7p14.3		chr7:29638504-29638445		
A_22_P000	2.819849	1.495618	2.819849	up	-0.73536	-0.76692	-0.696767	0.696767	0.717065	0.873975	SNHG17	small nucle	FALSE	Homo sapi	388796		37063911	chr20	37063852	-	hs|20q11.2	NR_01536	chr20:3706	NR_01536	Hs.400876
A_23_P156	2.513942	1.329951	2.513942	up	-0.517545	-0.913686	-0.625332	0.754088	0.661656	0.517545	SLC22A4	solute carri	FALSE	Homo sapi	6583	GO:000551	1.32E+08	chr5	1.32E+08	+	hs|5q31.1	NM_00305	chr5:13167	NM_00305	Hs.310591
A_21_P000	2.632813	1.396605	2.632813	up	-0.687131	-0.663976	-0.714553	0.70518	0.755	0.663976	MTRNR2L2	MT-RNR2-l	FALSE	Homo sapi	1E+08	GO:000573	79945938	chr5	79945879	-	hs|5q14.1	NM_00119	chr5:79945	NM_00119	Hs.666077
A_33_P322	5.915626	2.564531	5.915626	up	-1.108295	-1.513956	-1.207962	1.457034	1.108295	1.29805	PRR25	proline rich	FALSE	Homo sapi	388199		863861	chr16	863802	+	hs|16p13.3	NM_00101	chr16:8638	NM_00101	Hs.528461
A_33_P339	2.743788	1.456169	2.743788	up	-0.632112	-0.722596	-0.535631	0.936339	0.53563	1.006199	TMEM8C	transmemb	FALSE	Homo sapi	389827	GO:000751	1.36E+08	chr9	1.36E+08	-	hs|9q34.2	NM_00108	chr9:13637	NM_00108	Hs.512467
A_33_P328	5.311262	2.409055	5.311262	up	-1.225704	-1.261955	-1.11131	1.184428	1.11131	1.332458			FALSE	Homo sapiens cDNA FLJ46084 fis,			64414947	chr13	64414888	-	hs|13q21.31		chr13:64414947-64414888		
A_22_P000	3.153206	1.656819	3.153206	up	-0.681746	-0.975687	-0.68169	0.94482	0.68169	1.004825			FALSE	Q8S994_PHAAN (Q8S994) Glucosy			1.39E+08	chr5	1.39E+08	-	hs|5q31.2		chr5:139154925-139154866		
A_21_P000	3.459524	1.790574	3.459524	up	-1.107864	-0.794739	-0.974801	0.837045	0.794739	0.862533	LOC10192	uncharacte	FALSE	PREDICTED	1.02E+08		4576914	chr4	4576855	-	hs|4p16.2	XR_241657	chr4:45769	XR_241657	
A_33_P328	2.542515	1.346256	2.542515	up	-0.641146	-0.6092	-0.513559	0.513559	1.092862	0.668442	LOC10192	uncharacte	FALSE	PREDICTED	1.02E+08		34665248	chr6	34665191	+	hs|6p21.31	XR_249973	chr6:34665	XR_249973	Hs.645315
A_22_P000	6.117172	2.612865	6.117172	up	-1.120143	-1.527558	-1.467301	1.322419	1.120144	1.281029	FOXD3-AS1	FOXD3 anti	FALSE	Homo sapi	1.01E+08		63788046	chr1	63787987	-	hs|1p31.3	NR_12163	chr1:63788	NR_12163	Hs.585161
A_32_P207	2.144866	1.100887	2.144866	up	-0.706018	-0.491804	-0.376614	0.376614	0.917239	0.434372	SERTAD4-A	SERTAD4 a	FALSE	Homo sapi	574036		2.1E+08	chr1	2.1E+08	-	hs|1q32.2	NR_02433	chr1:2104	NR_02433	Hs.446946
A_21_P000	2.216812	1.148486	2.216812	up	-0.44471	-0.401309	-0.633971	0.5769	0.987259	0.401309	LOC10028	uncharacte	FALSE	PREDICTED	1E+08		46781584	chr12	46781388	+	hs|12q13.1	XR_171951	chr12:4678	XR_171951	Hs.730135
A_33_P327	2.149746	1.104167	2.149746	up	-0.503118	-0.561275	-0.658851	0.51316	0.503118	0.572978	C20orf196	chromoson	FALSE	chromoson	149840		5844557	chr20	5844498	+	hs|20p12.3	XM_00672	chr20:5844	XM_00672	Hs.529340
A_23_P605	6.639255	2.731021	6.639255	up	-1.487925	-1.312411	-1.313237	1.312411	1.447143	1.319937	UGT1A6	UDP glucur	FALSE	Homo sapi	54578	GO:005269	2.35E+08	chr2	2.35E+08	+	hs|2q37.1	NM_00107	chr2:23468	NM_00107	Hs.554822
A_23_P324	2.423073	1.276838	2.423073	up	-0.605117	-0.659149	-0.687824	0.666593	0.606714	0.605117	CEMIP	cell migrati	FALSE	Homo sapi	57214	GO:000551	81243693	chr15	81243634	+	hs|15q25.1	NM_01868	chr15:8124	NM_01868	Hs.459088
A_24_P501	2.200207	1.137639	2.200207	up	-0.495554	-0.617609	-0.560937	0.574054	0.66921	0.495554			FALSE				1.52E+08	chr6	1.52E+08	-	hs|6q25.1		chr6:151546724-151546685		
A_23_P381	2.256127	1.173848	2.256127	up	-0.696854	-0.625699	-0.436555	0.473264	0.436555	0.852617	ORMDL3	ORMDL sph	FALSE	Homo sapi	94103	GO:000551	38079397	chr17	38078912	-	hs|17q12	NM_13928	chr17:3807	NM_13928	Hs.514151
A_33_P341	3.519548	1.81539	3.519548	up	-0.897862	-0.90779	-0.734817	0.977669	0.734818	1.193215	UNCX	UNC home	FALSE	Homo sapi	340260	GO:004356	1276493	chr7	1276434	+	hs|7p22.3	NM_00108	chr7:12764	NM_00108	Hs.232272
A_24_P303	10.69335	3.418642	10.69335	up	-1.467748	-1.898528	-1.532617	1.964596	1.467748	1.924687	C9orf62	chromoson	FALSE	Homo sapi	157927		1.38E+08	chr9	1.38E+08	+	hs|9q34.3	NM_17352	chr9:13823	NM_17352	Hs.559511
A_23_P428	2.835929	1.503822	2.835929	up	-0.597106	-0.820654	-0.968401	0.692542	0.597106	0.835655	EZH1	enhancer c	FALSE	Homo sapi	2145	GO:003509	40852733	chr17	40852674	-	hs|17q21.2	NM_00199	chr17:4085	NM_00199	Hs.194669
A_21_P001	3.192751	1.6748	3.192751	up	-0.838723	-0.771781	-0.793621	0.948814	0.771781	0.899681	XLOC_l2_002910		FALSE	BROAD Institute lincRNA (XLOC_l2			17144925	chr12	17144740	+	hs|12p12.3		chr12:17144740-17144925		
A_21_P000	2.005353	1.003856	2.005353	up	-0.473121	-0.466208	-0.486743	0.466208	0.611806	0.507484	STEAP1B	STEAP fami	FALSE	Homo sapi	256227	GO:001602	22532239	chr7	22478371	-	hs|7p15.3	NM_00116	chr7:22532	NM_00116	Hs.729825
A_22_P000	6.607307	2.724062	6.607307	up	-1.504674	-1.280935	-1.196324	1.420711	1.196324	1.573221	lnc-CCT5-1	lnc-CCT5-1	FALSE	LNCipedia lincRNA (lnc-CCT5-1), lir			10249779	chr5	10249720	+	hs|5p15.2		chr5:10249720-10249779		
A_33_P338	2.384283	1.253556	2.384283	up	-0.595477	-0.582453	-0.596006	0.607871	0.796406	0.582453	RPS18	ribosomal	FALSE	Homo sapi	6222	GO:000551	33240492	chr6	33240433	+	hs|6p21.32	NM_02255	chr6:3324	NM_02255	Hs.627414
A_33_P323	2.201893	1.138744	2.201893	up	-0.52998	-0.439052	-0.465776	0.599951	0.439052	0.942422	ALDH3B1	aldehyde d	FALSE	Homo sapi	221	GO:000662	67786720	chr11	67786661	+	hs|11q13.2	NM_00129	chr11:6778	NM_00129	Hs.523841
A_22_P000	6.370231	2.671346	6.370231	up	-1.207486	-1.393625	-1.409626	1.211318	1.584497	1.207486	lnc-GLIPR1	lnc-GLIPR1	FALSE	DA381791 BRTHA2 Homo sapiens			76425060	chr12	76425001	+	hs|12q21.2	DA381791	chr12:76425001-76425		Hs.628359
A_23_P336	2.03261	1.023333	2.03261	up	-0.117219	-0.496538	-1.096156	0.553994	0.117219	0.688873	FURIN	furin (paire	FALSE	Homo sapi	5045	GO:000580	91426056	chr15	91425997	+	hs|15q26.1	NM_00256	chr15:9142	NM_00256	Hs.513153
A_19_P003	2.458962	1.298049	2.458962	up	-0.880668	-0.483149	-0.669688	0.530716	0.846778	0.483149	LINC01291	long interg	FALSE	Homo sapi	1.03E+08		75159858	chr2	75159799	+	hs|2p12	NR_12579	chr2:75159	NR_12579	Hs.708649
A_33_P328	3.712941	1.892562	3.712941	up	-0.942147	-0.997698	-0.915954	0.977979	0.915954	0.927956	CYGB	cytoglobin	FALSE	Homo sapi	114757	GO:004300	74523520	chr17	74523461	-	hs|17q25.1	NM_13426	chr17:7452	NM_13426	Hs.95120
A_32_P115	2.948189	1.559829	2.948189	up	-1.158128	-0.642245	-0.807862	0.656882	0.772125	0.642245			FALSE	BOVPOUBA polyubiqui		GO:000551	16285923	chr17	16285864	-	hs|17p11.2		chr17:016285923-016285864		
A_24_P931	16.14986	4.01345	16.14986	up	-1.927726	-1.962851	-1.982653	2.005964	2.233432	1.927726	GPR68	G protein-c	FALSE	Homo sapi	8111	GO:000695	91698988	chr14	91698929	-	hs|14q32.1	NM_00348	chr14:9169	NM_00348	Hs.8882
A_22_P000	3.868306	1.951702	3.868306	up	-0.817465	-1.100622	-0.836091	1.126829	0.817466	1.156632	TMEM210	transmemb	FALSE	transmemb	1.01E+08	GO:001602	1.4E+08	chr9	1.4E+08	-	hs|9q34.3		chr9:140066398-14006		Hs.512463
A_23_P392	2.10165	1.071523	2.10165	up	-0.541822	-0.513975	-0.506738	0.506739	0.602713	0.542581	ZFP36	ZFP36 ring	FALSE	Homo sapi	7538	GO:000551	39899817	chr19	39899758	+	hs|19q13.2	NM_00340	chr19:3989	NM_00340	Hs.534052
A_33_P327	4.052087	2.018665	4.052087	up	-0.792016	-1.207791	-1.144826	1.105284	0.792015	1.014063	HCN2	hyperpolar	FALSE	Homo sapi	610	GO:000588	617157	chr19	617098	+	hs|19p13.3	NM_00119	chr19:6170	NM_00119	Hs.124161
A_33_P324	4.691787	2.230138	4.691787	up	-1.005642	-1.007632	-0.886153	1.452852	0.886153	1.451981			FALSE	Pvt1 oncogene (non-protein codin			1.29E+08	chr8	1.29E+08	+	hs|8q24.21	M34432	chr8:128806902-1288		Hs.133107
A_32_P544	2.609162	1.383587	2.609162	up	-0.546861	-0.586472	-0.957562	0.546861	0.945408	0.567596			FALSE	Synthetic construct Homo sapiens			30210519	chr8	30210460	-	hs|8p12	CU676483	chr8:030210519-03021		Hs.706246
A_33_P341	2.219009	1.149916	2.219009	up	-0.60131	-0.441396	-0.438177	0.693135	0.438177	0.837552			FALSE	aa64c04.s1 NCI_CGAP_GCB1 Hom			11143339	chr19	11143280	-	hs|19p13.2	AA504818	chr19:11143339-11143		Hs.741659
A_33_P320	3.58506	1.841997	3.58506	up	-0.962664	-0.715687	-0.839348	1.096318	0.715687	1.196288	IQSEC3	IQ motif ar	FALSE	Homo sapi	440073	GO:004354	208312	chr12	176547	+	hs|12p13.3	NM_00117	chr12:1765	NM_00117	Hs.536319
A_22_P000	2.184911	1.127574	2.184911	up	-0.641696	-0.447345	-0.421435	0.421435	0.848398	0.602414	OSER1-AS1	OSER1 anti	FALSE	Homo sapi	1.01E+08		42839996	chr20	42839937	+	hs|20q13.1	NR_03833	chr20:4283	NR_03833	Hs.159049
A_33_P322	2.815944	1.493619	2.815944	up	-0.68674	-0.846345	-0.569742	0.953049	0.569742	0.855237	DOK3	docking pro	FALSE	Homo sapi	79930	GO:000551	1.77E+08	chr5	1.77E+08	-	hs|5q35.3	NM_00114	chr5:17693	NM_00114	Hs.720849
A_33_P322	2.455378	1.295945	2.455378	up	-0.559748	-0.689019	-0.638283	0.701593	0.559747	0.739445	MAML1	mastermin	FALSE	mastermin	9794	GO:000551	1.79E+08	chr5	1.79E+08	+	hs|5q35.3	AB209135	chr5:179223213-17922		Hs.631951
A_24_P219	6.104188	2.609799	6.104188	up	-1.305368	-1.137569	-1.370329	1.137569	1.721814	1.15675	CASKIN1	CASK intera	FALSE	Homo sapi	57524	GO:000551	2227503	chr16	2227444	-	hs|16p13.3	NM_02076	chr16:2227	NM_02076	Hs.643537
A_23_P288	2.324407	1.216863	2.324407	up	-0.551481	-0.581636	-0.639061	0.676458	0.55148	0.650472	CYP24A1	cytochrom	FALSE	Homo sapi	1591	GO:000680	52771005	chr20	52770946	-	hs|20q13.2	NM_00078	chr20:5277	NM_00078	Hs.89663
A_33_P338	2.236457	1.161215	2.236457	up	-0.443986	-0.563391	-0.442562	0.749044	0.442561	0.8421	LOC40055	uncharacte	FALSE	Homo sapi	400558		89235411	chr16	89235352	+	hs|16q24.3	NR_04620	chr16:8923	NR_04620	Hs.513818
A_22_P000	2.500097	1.321984	2.500097	up	-0.482008	-0.741296	-0.778571	0.697897	0.482008	0.784171	lnc-MAT2B	lnc-MAT2B	FALSE	Homo sapiens, clone IMAGE:4479			1.64E+08	chr5	1.64E+08	+	hs|5q34	BC011998	chr5:164028425-16402		Hs.437730
A_23_P294	2.145504	1.101317	2.145504	up	-0.587744	-0.465162	-0.473151	0.759939	0.465162	0.552792	GYG1	glycogenin	FALSE	Homo sapi	2992	GO:000551	1.49E+08	chr3	1.49E+08	+	hs|3q24	NM_00413	chr3:14871	NM_00413	Hs.477892
A_33_P362	3.636511	1.862555	3.636511	up	-0.720796	-1.100496	-0.770177	1.091598	0.720796	1.183801	LOC28493	uncharacte	FALSE	Homo sapi	284933		48939193	chr22	48939134	-	hs|22q13.3	BC048428	chr22:48939193-4893		Hs.159057

A_32_P123	2.089589	1.063219	2.089589	up	-0.479651	-0.477122	-0.409564	0.409564	0.928021	0.485737	TTC39C	tetratricop	FALSE	Homo sapi	125488		21715511	chr18	21715452	+	hs|18q11.2	NM_00129	chr18:2171	NM_00129	Hs.128576
A_33_P341	2.987596	1.578985	2.987596	up	-0.703272	-0.781139	-0.700758	0.911411	0.700759	0.939615	VPS9D1	VPS9 doma	FALSE	Homo sapi	9605	GO:004354	89777241	chr16	89777182	-	hs|16q24.3	NM_00491	chr16:8977	NM_00491	Hs.164410
A_24_P299	3.14443	1.652799	3.14443	up	-0.780333	-0.946867	-0.761569	0.804097	0.761569	0.903961	PDPN	podoplanir	FALSE	Homo sapi	10630	GO:000669	13940881	chr1	13940822	+	hs|1p36.21	NM_19838	chr1:1394C	NM_19838	Hs.468675
A_33_P342	2.049036	1.034945	2.049036	up	-0.707706	-0.62943	-0.327511	0.438066	0.327511	0.674612	FAM118A	family with	FALSE	Homo sapi	55007	GO:001602	45736854	chr22	45736795	+	hs|22q13.3	NM_00110	chr22:4573	NM_00110	Hs.265018
A_33_P323	3.019071	1.594105	3.019071	up	-0.767956	-0.678543	-0.919351	0.810592	0.92733	0.678543			FALSE	metallothionein 1C, pseudogene [S			56682802	chr16	56682743	+	hs|16q12.2		chr16:56682743-56682802		
A_21_P001	3.201405	1.678705	3.201405	up	-0.53509	-1.228635	-0.974356	0.53509	1.143634	0.619309			FALSE												
A_33_P327	5.307905	2.408143	5.307905	up	-1.04298	-1.118246	-1.049878	1.474438	1.04298	1.495907			FALSE				45737511	chr19	45737452	-	hs|19q13.32		chr19:045737511-045737452		
A_22_P00C	6.588466	2.719943	6.588466	up	-1.103865	-1.49844	-1.164822	1.717535	1.103865	1.571301	lnc-CDK5R1	lnc-CDK5R1	FALSE	BX098590 Soares_NFL_T_GBC_S1			30846114	chr17	30846055	+	hs|17q11.2	BX098590	chr17:30846055-3084		Hs.671440
A_19_P008	3.365308	1.750739	3.365308	up	-0.798028	-0.598603	-1.561653	0.927887	0.598603	0.767441	LOC101928	uncharacte	FALSE	Homo sapi	1.02E+08		1.73E+08	chr1	1.73E+08	-	hs|1q25.1	NR_12596	chr1:17338	NR_125960	
A_24_P641	2.35037	1.232888	2.35037	up	-0.695778	-0.508806	-0.327767	0.780052	0.327767	1.058491	PRRC2A	proline-rich	FALSE	Homo sapi	7916	GO:000551	31604126	chr6	31604067	+	hs|6p21.33	NM_08068	chr6:31604	NM_08068	Hs.436093
A_22_P00C	2.091519	1.064551	2.091519	up	-0.632743	-0.944321	-0.257829	0.480679	0.257829	0.620254	lnc-STOM-3	lnc-STOM-3	FALSE	Q3PGX6_PARDE (Q3PGX6) Malate			1.24E+08	chr9	1.24E+08	-	hs|9q33.2		chr9:124337236-124337177		
A_23_P811	2.783131	1.476709	2.783131	up	-0.574205	-0.696976	-0.60806	0.974312	0.574205	1.002368	SFRP2	secreted fr	FALSE	Homo sapi	6423	GO:001714	1.55E+08	chr4	1.55E+08	-	hs|4q31.3	NM_00301	chr4:1547C	NM_00301	Hs.481022
A_33_P341	2.736046	1.452092	2.736046	up	-0.838763	-0.70713	-0.465417	0.809944	0.465417	1.069607			FALSE				1.02E+08	chr10	1.02E+08	+	hs|10q24.31		chr10:101941285-101941344		
A_22_P00C	3.508106	1.810692	3.508106	up	-0.872999	-0.883168	-0.941731	0.882497	0.872999	0.978684	lnc-MARVE	lnc-MARVE	FALSE	LNCipedia lincRNA (lnc-MARVELD3			71757344	chr16	71757285	+	hs|16q22.2		chr16:71757285-71757344		
A_24_P336	6.557523	2.713151	6.557523	up	-1.324679	-1.306414	-1.459285	1.306414	1.339304	1.403357	BGLAP	bone gamn	FALSE	Homo sapi	632	GO:004362	1.56E+08	chr1	1.56E+08	+	hs|1q22	NM_19917	chr1:15621	NM_19917	Hs.654541
A_23_P705	2.653128	1.407694	2.653128	up	-0.397511	-0.463927	-0.441697	1.236298	0.397511	1.286138	VPS52	vacuolar pr	FALSE	Homo sapi	6293	GO:001990	33219398	chr6	33219339	-	hs|6p21.32	NM_02255	chr6:33219	NM_02255	Hs.480356
A_23_P259	5.788396	2.533164	5.788396	up	-1.098883	-1.171317	-0.931481	1.716734	0.931481	1.749595	GDF5	growth diff	FALSE	Homo sapi	8200	GO:000551	34022206	chr20	34022147	-	hs|20q11.2	NM_00055	chr20:3402	NM_00055	Hs.1573
A_33_P323	2.505947	1.325356	2.505947	up	-0.772337	-0.893536	-0.376207	0.789638	0.376207	0.768142			FALSE				66489339	chr9	66489280	-	hs|9q13		chr9:066489339-066489280		
A_33_P326	2.265732	1.179977	2.265732	up	-0.60296	-0.528494	-0.583811	0.528494	0.757898	0.538275	ITGA10	integrin, alp	FALSE	Homo sapi	8515	GO:000830	1.46E+08	chr1	1.46E+08	+	hs|1q21.1	NM_00363	chr1:14554	NM_00363	Hs.158237
A_24_P227	2.66452	1.413876	2.66452	up	-1.069755	-0.651494	-0.55087	0.717494	0.701145	0.55087	IL21R	interleukin	FALSE	Homo sapi	50615	GO:003010	27461383	chr16	27461324	+	hs|16p12.1	NM_18107	chr16:2746	NM_18107	Hs.210546
A_33_P670	2.544596	1.347436	2.544596	up	-0.64159	-0.880555	-0.526172	0.756136	0.526172	0.711685	SLC12A5	solute carri	FALSE	solute carri	57468	GO:000588	44652090	chr20	44652031	+	hs|20q13.1	AW963483	chr20:44652031-44652		Hs.732905
A_22_P00C	2.286532	1.193161	2.286532	up	-0.524139	-0.636917	-0.636173	0.615884	0.52414	0.642229	lnc-C20orf9	lnc-C20orf9	FALSE	LNCipedia lincRNA (lnc-C20orf96-1			278273	chr20	278214	-	hs|20p13		chr20:278273-278214		
A_33_P339	6.736528	2.752005	6.736528	up	-1.350452	-1.271349	-1.286058	1.271349	1.797797	1.27901	RNA5-8S5	RNA, 5.8S r	FALSE	Homo sapi	1E+08		156109	chrUn_gl00	156050	+		NR_00328	chrUn_gl0	NR_00328	Hs.426704
A_33_P324	3.919364	1.970619	3.919364	up	-1.296369	-0.830942	-0.906551	0.870492	0.830942	1.176563			FALSE	Homo sapiens cDNA clone IMAGE:			1.35E+08	chr10	1.35E+08	-	hs|10q26.3	BC027847	chr10:135137970-1351		Hs.551860
A_33_P327	2.128414	1.089779	2.128414	up	-0.485531	-0.537303	-0.613304	0.532653	0.615015	0.485531	ZNF217	zinc finger	FALSE	Homo sapi	7764	GO:000551	52185714	chr20	52185655	-	hs|20q13.2	NM_00652	chr20:5218	NM_00652	Hs.155040
A_22_P00C	2.639663	1.400353	2.639663	up	-0.640219	-0.698993	-0.586735	0.63416	0.586735	1.05422	KTN1-AS1	KTN1 antis	FALSE	Homo sapi	1E+08		56046713	chr14	56046654	-	hs|14q22.3	NR_02712	chr14:5604	NR_02712	Hs.633724
A_23_P153	2.544634	1.347458	2.544634	up	-0.680546	-0.670443	-0.51205	0.51205	1.125715	0.541572	INHBB	inhibin, bet	FALSE	Homo sapi	3625	GO:000551	1.21E+08	chr2	1.21E+08	+	hs|2q14.2	NM_00219	chr2:1211C	NM_00219	Hs.1735
A_24_P877	2.02138	1.01534	2.02138	up	-0.370822	-0.382887	-0.348103	0.79959	0.348103	0.796516	EEF2	eukaryotic	FALSE	Homo sapi	1938	GO:000551	3977256	chr19	3976730	-	hs|19p13.3	NM_00196	chr19:3977	NM_00196	Hs.515070
A_33_P341	2.753031	1.461021	2.753031	up	-0.688887	-0.712599	-0.668805	0.928866	0.715101	0.668805			FALSE				34875886	chr15	34875827	-	hs|15q14		chr15:034875886-034875827		
A_21_P001	3.134686	1.648321	3.134686	up	-0.657931	-0.842097	-0.733279	0.801466	1.25226	0.657931			FALSE				24114297	chr1	24114238	-	hs|1p36.11		chr1:024114297-024114238		
A_22_P00C	2.043335	1.030926	2.043335	up	-0.665949	-0.424824	-0.574061	0.449382	0.424824	0.553737	IPO9-AS1	IPO9 antise	FALSE	Homo sapi	1.01E+08		2.02E+08	chr1	2.02E+08	-	hs|1q32.1	NR_04669	chr1:20179	NR_04669	Hs.604842
A_23_P330	2.379692	1.250775	2.379692	up	-0.410078	-0.359785	-0.615093	1.012181	0.359785	0.995404	PIP5K1C	phosphatid	FALSE	Homo sapi	23396	GO:000690	3630729	chr19	3630670	-	hs|19p13.3	NM_01239	chr19:3630	NM_01239	Hs.282177
A_22_P00C	2.216883	1.148533	2.216883	up	-0.563791	-0.738172	-0.473636	0.586253	0.610111	0.473636	lnc-ENPP1-	lnc-ENPP1-	FALSE	LNCipedia lincRNA (lnc-ENPP1-2),			1.32E+08	chr6	1.32E+08	+	hs|6q23.2		chr6:132272275-132272334		
A_33_P334	5.512505	2.462708	5.512505	up	-1.351363	-1.425919	-0.830733	1.317648	0.830733	1.631728	VSX1	visual syste	FALSE	Homo sapi	30813	GO:004356	25062369	chr20	25062310	-	hs|20p11.2	NM_19942	chr20:2506	NM_19942	Hs.274264
A_33_P324	2.240053	1.163533	2.240053	up	-0.833073	-0.393914	-0.812784	0.567543	0.393914	0.489371			FALSE				1.02E+08	chr14	1.02E+08	+	hs|14q32.31		chr14:102231782-102231841		
A_22_P00C	5.345951	2.418447	5.345951	up	-1.056431	-1.482488	-1.012918	1.32291	1.012918	1.367675	lnc-UTS2-1	lnc-UTS2-1	FALSE	603205994F1 NIH_MGC_97 Homo			7887223	chr1	7887164	-	hs|1p36.23	BI464920	chr1:7887223-7887164		Hs.680218
A_33_P370	4.580123	2.195386	4.580123	up	-0.993764	-1.256975	-0.845793	1.251737	0.845793	1.392098			FALSE												
A_33_P323	3.272393	1.710346	3.272393	up	-0.911628	-0.782336	-0.874122	0.916278	0.782336	0.864338	RBAK-RBAK	RBAK-RBAK	FALSE	Homo sapi	1.01E+08		5112853	chr7	5112794	+	hs|7p22.1	NM_00120	chr7:51127	NM_00120	Hs.396178
A_24_P307	3.602418	1.848965	3.602418	up	-1.065397	-0.886352	-0.759272	1.13675	0.759272	0.939853	TMEM95	transmemt	FALSE	Homo sapi	339168	GO:001602	7259776	chr17	7259717	+	hs|17p13.1	NM_19815	chr17:7259	NM_19815	Hs.259432
A_33_P308	2.049801	1.035484	2.049801	up	-0.503886	-0.410773	-0.586424	0.410773	0.632458	0.562139	TTC39C	tetratricop	FALSE	Homo sapi	125488		21600037	chr18	21599978	+	hs|18q11.2	NM_00124	chr18:2159	NM_00124	Hs.128576
A_24_P917	2.049301	1.035132	2.049301	up	-0.785188	-0.725425	-0.348879	0.445367	0.348879	0.451658	NF1	neurofibro	FALSE	Homo sapi	4763	GO:000165	29704619	chr17	29704560	+	hs|17q11.2	NM_00104	chr17:2970	NM_00104	Hs.113577
A_33_P328	2.211612	1.145098	2.211612	up	-0.434556	-0.73024	-0.589769	0.727422	0.434556	0.518751	ATP1A3	ATPase, Na	FALSE	Homo sapi	478	GO:000834	42470805	chr19	42470746	-	hs|19q13.2	NM_00125	chr19:4247	NM_00125	Hs.515427
A_23_P200	4.239002	2.083725	4.239002	up	-0.950162	-1.037112	-1.117731	0.950162	1.153562	1.042444	DPT	dermatopc	FALSE	Homo sapi	1805	GO:003101	1.69E+08	chr1	1.69E+08	-	hs|1q24.2	NM_00193	chr1:16866	NM_00193	Hs.80552
A_23_P906	2.271228	1.183473	2.271228	up	-0.607366	-0.536097	-0.535245	0.648927	0.535245	0.687538	STEAP3	STEAP fami	FALSE	Homo sapi	55240	GO:000551	1.2E+08	chr2	1.2E+08	+	hs|2q14.2	NM_18291	chr2:12002	NM_18291	Hs.647822
A_33_P335	2.246621	1.167757	2.246621	up	-0.566879	-0.653684	-0.544215	0.544215	0.630015	0.564263			FALSE	ribosomal protein L15 pseudogene			16025979	chr16	16025920	-	hs|16p13.11		chr16:16025979-16025920		
A_19_P003	2.053561	1.038127	2.053561	up	-0.320305	-0.811121	-0.39393	0.581282	0.320305	0.687439	FLJ32255	uncharacte	FALSE	Homo sapi	643977		42991256	chr5	42991197	-	hs|5p12		chr5:42991	NR_10464	Hs.535791
A_23_P143	3.028456	1.598583	3.028456	up	-0.607786	-0.673736	-0.629631	1.061818	0.607786	1.214991	LBP	lipopolysac	FALSE	Homo sapi	3929	GO:000551	36995434	chr20	36993369	+	hs|20q11.2	NM_00413	chr20:3699	NM_00413	Hs.154078
A_33_P323	2.207265	1.14226	2.207265	up	-0.576003	-0.491897	-0.474298	0.689428	0.474298	0.720858	GOLM1	golgi meml	FALSE	Homo sapi	51280	GO:000551	88642401	chr9	88642342	-	hs|9q21.33	NM_01654	chr9:88642	NM_01654	Hs.494337
A_33_P327	3.434406	1.78006	3.434406	up	-0.718868	-0.972954	-0.715753	1.117529	0.715754	1.099324	TOR2A	torsin fami	FALSE	Homo sapi	27433	GO:000517	1.3E+08	chr9	1.3E+08	-	hs|9q34.11	NM_00113	chr9:13049	NM_00113	Hs.444106
A_21_P00C	3.662656	1.87289	3.662656	up	-1.022874	-0.823588	-0.907411	0.823588	1.003027	1.038183	lnc-EPHA6-	lnc-EPHA6-	FALSE	LNCipedia lincRNA (lnc-EPHA6-1),			96336779	chr3	96336720	+	hs|3q11.2		chr3:96336720-96336779		
A_33_P327	2.624482	1.392033	2.624482	up	-0.658781	-0.723954	-0.620205	0.737049	0.620204	0.815906			FALSE				627479	chr11	627420	-	hs|11p15.5		chr11:000627479-000627420		
A_32_P10C	2.469269	1.304084	2.469269	up	-0.795312	-0.550403	-0.64029	0.550403	0.797111	0.578733	LOC101927	uncharacte	FALSE	PREDICTED	1.02E+08		1.07E+08	chr7	1.07E+08	-	hs|7q22.3	XR_242328	chr7:10738	XR_242328	
A_24_P496	2.10519	1.07395	2.10519	up	-0.518632	-0.509279	-0.5007	0.5007	0.653276	0.539265			FALSE				62641433	chr4	62641374	-	hs|4q13.1		chr4:62641433-62641374		
A_33_P324	8.553537	3.096521	8.553537	up	-1.557534	-1.471077	-1.50577	1.481414	1.802692	1.471077	CES1	carboxylest	FALSE	Homo sapi	1066	GO:005268	55836823	chr16	55836764	-	hs|16q12.2	NM_00102	chr16:5583	NM_00102	Hs.558865
A_33_P387	3.190959	1.67399	3.190959	up	-0.833409	-0.8156	-0.800789	0.800789	0.913268	0.858114	SNED1	sushi, nidog	FALSE	Homo sapi	25992	GO:000716	2.42E+08	chr2	2.42E+08	+	hs|2q37.3	NM_00108	chr2:24203	NM_00108	Hs.471834
A_24_P808	4.131529	2.046676	4.131529	up	-0.967895	-0.942219	-1.12621	1.096726	0.942219	1.064758	RPS14	ribosomal p	FALSE	Homo sapi	6208	GO:004802	1.5E+08	chr5	1.5E+08	-	hs|5q33.1	NM_00561	chr5:14982	NM_00561	Hs.744846
A_21_P001	6.917691	2.790291	6.917691	up	-1.268118	-1.535347	-1.327276	1.268118	1.677595	1.294418			FALSE	acylphosphatase 2, muscle type [S			54307431	chr2	54307372	+	hs|2p16.2	XR_245004	chr2:54307	XR_245004	
A_22_P00C	2.368876	1.244202	2.368876	up	-0.585109	-0.675145	-0.381452	0.725194	0.381452	0.984255	lnc-RP11-5	lnc-RP11-5	FALSE	DB125444 THYMU2 Homo sapiens			2320816	chr4	2320757	+	hs|4p16.3	DB125444	chr4:2320757-232081		Hs.590197

A_33_P359	2.199321	1.137058	2.199321	up	-0.617655	-0.489942	-0.699825	0.566497	0.547313	0.489942	OR7E12P	olfactory re	FALSE	Homo sapi	10821		3412514	chr11	3412455	-	hs|11p15.4	NR_04499	chr11:3412	NR_04499	Hs.741779
A_33_P331	2.104097	1.073201	2.104097	up	-0.253326	-0.926314	-0.640556	0.423175	0.722905	0.253326	lnc-CDH4-1	lnc-CDH4-1	FALSE	LNCipedia lincRNA (lnc-CDH4-1), li			60523586	chr20	60523527	+	hs|20q13.33		chr20:60523527-60523586		
A_23_P972	2.666111	1.414737	2.666111	up	-0.589584	-0.533952	-1.006418	0.777815	0.533952	0.80249	ZNF691	zinc finger	FALSE	Homo sapi	51058	GO:000563	43317858	chr1	43317799	+	hs|1p34.2	NM_01591	chr1:43317	NM_01591	Hs.20879
A_33_P331	3.402	1.766383	3.402	up	-0.758468	-0.933643	-0.98635	0.967982	0.758468	0.894238	CALY	calcyon ne	FALSE	Homo sapi	50632	GO:004580	1.35E+08	chr10	1.35E+08	-	hs|10q26.3	NM_01572	chr10:1351	NM_01572	Hs.148680
A_22_P000	2.802667	1.4868	2.802667	up	-0.651276	-0.79151	-0.706083	0.819352	0.651277	0.840903	lnc-MEPCE	lnc-MEPCE	FALSE	LNCipedia lincRNA (lnc-MEPCE-1),			1E+08	chr7	1E+08	+	hs|7q22.1		chr7:100034074-100034133		
A_23_P872	3.808014	1.929039	3.808014	up	-1.015973	-0.76052	-1.616875	0.807878	0.82535	0.76052	SAA4	serum amy	FALSE	Homo sapi	6291	GO:003436	18254043	chr11	18253984	-	hs|11p15.1	NM_00651	chr11:1825	NM_00651	Hs.1955
A_24_P791	2.149248	1.103832	2.149248	up	-0.253246	-0.883131	-0.264021	0.941331	0.253246	0.716521	SCAMP4	secretory c	FALSE	Homo sapi	113178	GO:001602	1925606	chr19	1925547	+	hs|19p13.3	NM_07983	chr19:1925	NM_07983	Hs.144980
A_33_P321	3.167201	1.663208	3.167201	up	-0.832346	-0.806407	-0.88369	0.806407	0.83014	0.830637			FALSE				26363183	chr2	26363124	-	hs|2p23.3		chr2:026363183-026363124		
A_22_P000	2.004275	1.00308	2.004275	up	-0.36417	-0.37577	-0.644209	0.455497	0.805424	0.36417	LOC100288	uncharacte	FALSE	Homo sapi	1E+08		46965148	chr12	46965089	+	hs|12q13.11		chr12:4696	NR_12537	Hs.729166
A_24_P218	5.427316	2.440239	5.427316	up	-0.792821	-1.751459	-1.642241	1.165596	0.792822	1.175777	ZNF467	zinc finger	FALSE	Homo sapi	168544	GO:000635	1.49E+08	chr7	1.49E+08	-	hs|7q36.1	NM_20733	chr7:14946	NM_20733	Hs.726477
A_33_P332	6.143082	2.618963	6.143082	up	-1.315944	-1.275937	-1.276475	1.306177	1.275937	1.406417			FALSE	eukaryotic translation elongation f			97644732	chrX	97644673	+	hs|Xq21.33		chrX:97644673-97644732		
A_32_P167	3.279324	1.713398	3.279324	up	-0.809343	-0.80702	-0.808811	0.80702	1.089081	0.81892	CLMN	calmin (cal	FALSE	Homo sapi	79789	GO:000573	95648676	chr14	95648617	-	hs|14q32.1	NM_02473	chr14:9564	NM_02473	Hs.301478
A_23_P423	2.435984	1.284505	2.435984	up	-0.70476	-0.756457	-0.499089	0.663737	0.499089	0.730382	HLA-DMA	major histc	FALSE	Homo sapi	3108	GO:004883	32916625	chr6	32916566	-	hs|6p21.32	NM_00612	chr6:32916	NM_00612	Hs.728759
A_23_P397	2.126645	1.088579	2.126645	up	-0.43275	-0.414706	-0.341417	0.903142	0.341417	0.832306	GSTM2	glutathione	FALSE	Homo sapi	2946	GO:000680	1.1E+08	chr1	1.1E+08	+	hs|1p13.3	NM_00084	chr1:11021	NM_00084	Hs.279837
A_33_P328	3.344658	1.741859	3.344658	up	-0.689508	-0.821355	-0.769873	1.104072	0.689508	1.15126			FALSE				67343662	chr9	67343603	-	hs|9q13		chr9:067343662-067343603		
A_22_P000	2.581417	1.368163	2.581417	up	-0.696186	-0.643022	-0.740783	0.696322	0.643022	0.685154	DANCR	differentia	FALSE	Homo sapi	57291		53579126	chr4	53579067	+	hs|4q12	NR_02403	chr4:53579	NR_02403	Hs.744077
A_24_P147	6.652098	2.73381	6.652098	up	-0.950109	-1.455211	-1.138328	1.847186	0.95011	1.860485	PCSK1N	proprotein	FALSE	Homo sapi	27344	GO:001095	48690521	chrX	48690462	-	hs|Xp11.23	NM_01327	chrX:4869C	NM_01327	Hs.522640
A_23_P143	3.198978	1.677611	3.198978	up	-0.716157	-0.817006	-0.80103	0.815968	1.166515	0.716157	ID2	inhibitor of	FALSE	Homo sapi	3398	GO:000165	8823030	chr2	8822642	+	hs|2p25.1	NM_00216	chr2:88226	NM_00216	Hs.180919
A_33_P334	7.119142	2.831703	7.119142	up	-1.273139	-1.593389	-1.580488	1.348636	1.273139	1.42632			FALSE	Homo sapiens mRNA; cDNA DKFZp			21005279	chr15	21005220	+	hs|15q11.2	AL512723	chr15:21005220-21005		Hs.567600
A_33_P321	2.139474	1.097256	2.139474	up	-0.4217	-0.347276	-0.54696	0.85931	0.347276	0.769247	COL16A1	collagen, ty	FALSE	Homo sapi	1307	GO:000551	32148595	chr1	32148536	-	hs|1p35.2	NM_00185	chr1:32148	NM_00185	Hs.368921
A_24_P290	2.318828	1.213396	2.318828	up	-0.523689	-0.584757	-0.793569	0.653688	0.523689	0.560796	FAM86B3P	family with	FALSE	Homo sapi	286042		8094687	chr8	8093452	+	hs|8p23.1		chr8:80934	NR_02436	Hs.656318
A_23_P864	2.518776	1.332723	2.518776	up	-0.52452	-0.556525	-0.571519	0.52452	1.21199	0.609095	CH25H	cholesterol	FALSE	Homo sapi	9023	GO:000662	90965860	chr10	90965801	-	hs|10q23.3	NM_00395	chr10:9096	NM_00395	Hs.47357
A_23_P358	2.003907	1.002815	2.003907	up	-0.57679	-0.52692	-0.434721	0.513029	0.522266	0.434721	POPDC3	popeye dor	FALSE	Homo sapi	64208	GO:000815	1.06E+08	chr6	1.06E+08	-	hs|6q21	NM_02236	chr6:10560	NM_02236	Hs.458336
A_23_P104	3.024267	1.596585	3.024267	up	-0.72867	-0.848524	-0.674884	0.857297	0.674884	1.005497	DUX4	double hor	FALSE	Homo sapi	1E+08	GO:003196	107030	chrUn_gl00	106972	+		NM_00129	chrUn_gl0C	NM_00129	Hs.728749
A_23_P153	4.346498	2.119854	4.346498	up	-0.949158	-1.007454	-1.024279	1.222148	0.949158	1.207364	EEF1A1	eukaryotic	FALSE	Homo sapi	1915	GO:000551	74228739	chr6	74228680	-	hs|6q13	NM_00140	chr6:74228	NM_00140	Hs.535192
A_33_P320	2.013287	1.009553	2.013287	up	-0.384988	-0.408485	-0.495291	0.668404	0.384988	0.686503	KANK1	KN motif a	FALSE	Homo sapi	23189	GO:000551	745519	chr9	745460	+	hs|9p24.3	NM_00125	chr9:74546	NM_00125	Hs.306764
A_23_P106	2.034245	1.024493	2.034245	up	-0.727876	-0.655513	-0.395239	0.417518	0.395239	0.482096	CRISPLD2	cysteine-ric	FALSE	Homo sapi	83716	GO:003101	84942197	chr16	84942138	+	hs|16q24.1	NM_03147	chr16:8494	NM_03147	Hs.513779
A_33_P341	2.179644	1.124093	2.179644	up	-0.723065	-0.731751	-0.428856	0.428856	0.49222	0.567531			FALSE				1.49E+08	chr1	1.49E+08	+	hs|1q21.2	AV727532	chr1:149400094-1494C		Hs.576457
A_24_P218	2.504531	1.324541	2.504531	up	-0.199917	-0.740685	-0.425069	1.233516	0.199917	1.174518	EIF4B	eukaryotic	FALSE	Homo sapi	1975	GO:000551	53435355	chr12	53435296	+	hs|12q13.1	NM_00141	chr12:5343	NM_00141	Hs.648394
A_33_P335	2.044303	1.031609	2.044303	up	-0.333376	-0.791911	-0.261419	0.622318	0.261419	0.824386	C9orf173	chromoson	FALSE	Homo sapi	441476		1.4E+08	chr9	1.4E+08	+	hs|9q34.3	NM_00125	chr9:14014	NM_00125	Hs.372640
A_22_P000	3.005924	1.587809	3.005924	up	-0.772779	-0.709639	-0.815956	0.851574	0.709639	0.903838	lnc-OPN4-2	lnc-OPN4-2	FALSE	Homo sapiens cDNA FLJ33309 fis,			87407807	chr10	87407748	+	hs|10q23.1	AK090628	chr10:87407748-87407		Hs.568871
A_33_P329	2.608493	1.383217	2.608493	up	-1.234662	-0.372512	-0.529478	0.436709	1.203777	0.372512	FAM166A	family with	FALSE	Homo sapi	401565	GO:000563	1.4E+08	chr9	1.4E+08	-	hs|9q34.3	NM_00100	chr9:14013	NM_00100	Hs.522530
A_33_P341	3.302942	1.723752	3.302942	up	-0.682288	-0.867503	-0.700609	1.08937	0.682288	1.149198	NLRC3	NLR family,	FALSE	Homo sapi	197358	GO:000367	3612970	chr16	3612911	-	hs|16p13.3	AK090476	chr16:3612970-3612911		
A_33_P325	3.338496	1.739198	3.338496	up	-0.693586	-0.867578	-0.727039	1.112952	0.693585	1.122855	CEBPD	CCAAT/enh	FALSE	Homo sapi	1052	GO:000107	48649932	chr8	48649873	-	hs|8q11.21	NM_00519	chr8:48649	NM_00519	Hs.440829
A_22_P000	2.590699	1.373341	2.590699	up	-0.41941	-0.592724	-0.586292	0.966584	0.41941	1.135604	lnc-DHX15-	lnc-DHX15-	FALSE	PREDICTED: Homo sapiens unchara			24473774	chr4	24473715	-	hs|4p15.2		chr4:24473774-24473		Hs.627177
A_21_P000	2.168341	1.116592	2.168341	up	-0.275358	-0.806778	-0.357438	0.739522	0.275358	0.89532	LINC01016	long interg	FALSE	Homo sapi	1.01E+08		33857474	chr6	33857415	-	hs|6p21.31	NR_03898	chr6:33857	NR_03898	Hs.547104
A_23_P428	2.00732	1.005271	2.00732	up	-0.484869	-0.639378	-0.441664	0.441664	0.554978	0.45326	STEAP2	STEAP fami	FALSE	Homo sapi	261729	GO:005285	89866596	chr7	89866537	+	hs|7q21.13	NM_15299	chr7:89866	NM_15299	Hs.489051
A_23_P161	2.65855	1.41064	2.65855	up	-0.617785	-0.701724	-0.500938	1.005341	0.500938	0.905193	FOSL1	FOS-like an	FALSE	Homo sapi	8061	GO:000551	65660157	chr11	65660098	-	hs|11q13.1	NM_00543	chr11:6566	NM_00543	Hs.283565
A_33_P326	3.884416	1.957698	3.884416	up	-1.014996	-0.830694	-0.861763	1.127393	0.830694	1.207553	GDNF	glial cell de	FALSE	Homo sapi	2668	GO:000165	37814012	chr5	37813953	-	hs|5p13.2	NM_00119	chr5:37814	NM_00119	Hs.248114
A_21_P001	6.293427	2.653846	6.293427	up	-1.420557	-1.197045	-1.254802	1.46905	1.197045	1.423038			FALSE												
A_33_P322	2.049309	1.035138	2.049309	up	-0.361964	-0.363995	-0.358483	0.814173	0.358483	0.848315	CST3	cystatin C	FALSE	Homo sapi	1471	GO:000551	23614617	chr20	23614558	-	hs|20p11.2	NM_00009	chr20:2361	NM_00009	Hs.304682
A_24_P578	5.625622	2.492013	5.625622	up	-0.865645	-1.388419	-1.08762	1.639004	0.865644	1.629705	BHLHE23	basic helix-	FALSE	Homo sapi	128408	GO:000635	61637561	chr20	61637502	-	hs|20q13.3	NM_08060	chr20:6163	NM_08060	Hs.551230
A_33_P370	2.360861	1.239313	2.360861	up	-0.429872	-0.624224	-0.838582	0.429872	0.86397	0.531421	EPHA5-AS1	EPHA5 anti	FALSE	Homo sapi	1E+08		66559103	chr4	66559044	+	hs|4q13.1	NR_03413	chr4:66559	NR_03413	Hs.201854
A_24_P212	7.258136	2.859599	7.258136	up	-1.261567	-1.346404	-1.34405	1.779781	1.261566	1.58543			FALSE	Homo sapiens cDNA FLJ25155 fis,			46039332	chr13	46039273	-	hs|13q14.1	AK057884	chr13:46039332-46039		Hs.507948
A_33_P335	2.67301	1.418465	2.67301	up	-0.620852	-0.703797	-0.658468	0.815766	0.620853	0.835659	MAP4	microtubul	FALSE	Homo sapi	4134	GO:000551	48130383	chr3	48130324	-	hs|3p21.31	NM_03088	chr3:4813C	NM_03088	Hs.517949
A_24_P801	2.929001	1.550409	2.929001	up	-0.768696	-0.682852	-0.698757	0.866347	0.682852	0.951721			FALSE	Homo sapiens proliferation-inducii			1.36E+08	chr9	1.36E+08	-	hs|9q34.2	AY239294	chr9:136216408-136215841		
A_24_P399	2.19659	1.135266	2.19659	up	-0.582316	-0.483131	-0.472751	0.747094	0.472751	0.647755	HEPH	hephaestin	FALSE	Homo sapi	9843	GO:004847	65486387	chrX	65486328	+	hs|Xq12	NM_01479	chrX:65486	NM_01479	Hs.31720
A_21_P000	6.226461	2.638412	6.226461	up	-1.459477	-1.280725	-1.028063	1.588381	1.028063	1.530529	LINC01150	long interg	FALSE	Homo sapi	1.02E+08		1929145	chr11	1929086	+	hs|11p15.5	NR_12053	chr11:1929	NR_12053	Hs.523404
A_22_P000	3.932324	1.975382	3.932324	up	-0.832611	-1.205971	-0.98912	1.071635	0.832611	0.994199	lnc-VWF-2	lnc-VWF-2:	FALSE	LNCipedia lincRNA (lnc-VWF-2), lir			6277373	chr12	6277314	-	hs|12p13.31		chr12:6277373-6277314		
A_22_P000	2.089289	1.063012	2.089289	up	-0.97312	-0.372064	-0.544581	0.430737	0.372064	0.496469			FALSE				1.21E+08	chr8	1.21E+08	+	hs|8q24.12		chr8:121068361-121068420		
A_33_P338	2.696189	1.430922	2.696189	up	-0.691211	-0.769798	-0.416938	0.894347	0.416938	1.103532	OSCAR	osteoclast a	FALSE	Homo sapi	126014	GO:000588	54599098	chr19	54599039	-	hs|19q13.4	NM_20681	chr19:5459	NM_20681	Hs.347655
A_24_P233	2.19926	1.137018	2.19926	up	-0.452185	-0.675352	-0.435888	0.777505	0.435888	0.634236	SDHC	succinate d	FALSE	Homo sapi	6391	GO:000609	1.61E+08	chr1	1.61E+08	+	hs|1q23.3	NM_00300	chr1:16132	NM_00300	Hs.444472
A_23_P710	2.071798	1.050883	2.071798	up	-0.504468	-0.53672	-0.482875	0.482876	0.634675	0.511036	IL6	interleukin	FALSE	Homo sapi	3569	GO:000551	22771266	chr7	22771207	+	hs|7p15.3	NM_00060	chr7:22771	NM_00060	Hs.654458
A_22_P000	2.064801	1.046003	2.064801	up	-0.390392	-0.516814	-0.554394	0.390392	0.747931	0.538086	lnc-CCDC5	lnc-CCDC5:	FALSE	LNCipedia lincRNA (lnc-CCDC51-1)			48481995	chr3	48481936	-	hs|3p21.31		chr3:48481995-48481936		
A_23_P304	4.64619	2.216048	4.64619	up	-1.197471	-1.067161	-1.153736	1.067161	1.089083	1.073534	EMP2	epithelial n	FALSE	Homo sapi	2013	GO:000551	10622683	chr16	10622624	-	hs|16p13.1	NM_00142	chr16:1062	NM_00142	Hs.531561
A_33_P334	4.582342	2.196085	4.582342	up	-0.986878	-1.322374	-0.876323	1.171861	0.876323	1.354498	MOB1B	MOB kinas	FALSE	Homo sapi	92597	GO:000551	71768266	chr4	71768207	+	hs|4q13.3	NM_00124	chr4:71768	NM_00124	Hs.691454
A_22_P000	2.090161	1.063614	2.090161	up	-0.561285	-0.545653	-0.454043	0.555146	0.454043	0.620673	SSPO	SCO-spond	FALSE	Homo sapi	23145	GO:000573	1.5E+08	chr7	1.5E+08	+	hs|7q36.1	NM_19845	chr7:14950	NM_19845	Hs.632022
A_33_P324	2.021663	1.015542	2.021663	up	-0.58087	-0.580393	-0.415036	0.623823	0.415036	0.431468	MTURN	maturin, ne	FALSE	Homo sapi	222166	GO:000727	30197566	chr7	30197507	+	hs|7p14.3	NM_15279	chr7:30197	NM_15279	Hs.200100

A_21_P001	2.383137	1.252862	2.383137	up	-0.835871	-0.904127	-0.360524	0.665079	0.632461	0.360524			FALSE				65193472	chr11	65193413	-	hs|11q13.1		chr11:065193472-065193413		
A_22_P000	2.256654	1.174185	2.256654	up	-0.714247	-0.594605	-0.548251	0.565982	0.548251	0.551219	lnc-C1orf22	lnc-C1orf22	FALSE	LNCipedia lincRNA (lnc-C1orf222-2			1980827	chr1	1980768	-	hs|1p36.33		chr1:1980827-1980768		
A_33_P347	3.502672	1.808456	3.502672	up	-0.753886	-0.908118	-0.86784	1.06644	0.753885	1.075198	SFTA1P	surfactant	FALSE	AGENCOUF	207107		10835938	chr10	10835879	-	hs|10p14	BU601128	chr10:10835938-10835		Hs.31562
A_23_P158	2.058352	1.04149	2.058352	up	-0.478619	-0.472034	-0.492708	0.609851	0.472034	0.599223	ALPK2	alpha-kinas	FALSE	Homo sapi	115701	GO:000467	56148827	chr18	56148768	-	hs|18q21.3	NM_05294	chr18:5614	NM_05294	Hs.628152
A_22_P000	8.357558	3.063082	8.357558	up	-1.049901	-1.743586	-1.555296	1.90441	1.049901	1.886151	lnc-KIF25-2	lnc-KIF25-2	FALSE	LNCipedia lincRNA (lnc-KIF25-2), li			1.69E+08	chr6	1.69E+08	+	hs|6q27		chr6:168631835-168631894		
A_32_P216	4.733219	2.242822	4.733219	up	-1.312182	-0.919547	-1.140271	1.28707	0.919547	1.149847	lnc-NAV1-3	lnc-NAV1-3	FALSE	DB229023 TRACH3 Homo sapiens			2.01E+08	chr1	2.01E+08	+	hs|1q32.1	DB229023	chr1:201476464-20147		Hs.716063
A_22_P000	2.64898	1.405437	2.64898	up	-0.604089	-0.942868	-0.722416	0.604089	0.652723	0.690125	lnc-TRIM41	lnc-TRIM41	FALSE	LNCipedia lincRNA (lnc-TRIM41-3),			1.81E+08	chr5	1.81E+08	+	hs|5q35.3		chr5:180619080-180619139		
A_21_P000	2.09768	1.068795	2.09768	up	-0.528378	-0.603087	-0.496471	0.496471	0.513984	0.567993	SNORD83B	small nucle	FALSE	Homo sapi	116938		39709883	chr22	39709824	-	hs|22q13.1	NR_000028	chr22:3970	NR_000028	Hs.119598
A_22_P000	3.043635	1.605795	3.043635	up	-1.070825	-0.981055	-0.590895	0.925588	0.590895	0.658127	HIF1A-AS1	HIF1A antis	FALSE	Homo sapi	1.01E+08		62162484	chr14	62162425	-	hs|14q23.2	NR_047116	chr14:6216	NR_047116	
A_22_P000	3.183024	1.670398	3.183024	up	-0.730536	-1.056401	-0.753132	0.875943	0.730536	0.864646	PSMG3-AS1	PSMG3 ant	FALSE	Homo sapi	114796		1609915	chr7	1609856	+	hs|7p22.3	NR_021487	chr7:16098	NR_021487	Hs.436146
A_33_P338	3.850161	1.944919	3.850161	up	-0.696227	-0.898817	-1.107626	1.331945	0.696227	1.103913	TNXB	tenascin XE	FALSE	Homo sapi	7148	GO:003003	32009196	chr6	32009137	-	hs|6p21.33	NM_01910	chr6:32009	NM_01910	Hs.485104
A_33_P341	2.279501	1.188718	2.279501	up	-0.626458	-0.551992	-0.407793	0.616504	0.407793	0.955614	PDE9A	phosphodie	FALSE	Homo sapi	5152	GO:000551	44190178	chr21	44190119	+	hs|21q22.3	AK074662	chr21:44190119-44190178		
A_23_P606	2.287939	1.194048	2.287939	up	-0.712072	-0.681397	-0.525131	0.612358	0.525131	0.526056	RPRD2	regulation	FALSE	Homo sapi	23248	GO:001659	1.5E+08	chr1	1.5E+08	+	hs|1q21.3	NM_01520	chr1:15044	NM_01520	Hs.213666
A_21_P000	4.019708	2.007091	4.019708	up	-0.824585	-1.428118	-1.144711	0.824585	0.920221	0.879053	SNORA63	small nucle	FALSE	Homo sapi	6043		1.87E+08	chr3	1.87E+08	+	hs|3q27.3	NR_002586	chr3:18650	NR_002586	Hs.518475
A_32_P640	2.446115	1.290492	2.446115	up	-0.682893	-0.566726	-0.577194	0.566726	0.8135	0.664438	LOC400590	uncharacte	FALSE	Homo sapi	400590		29361620	chr17	29361561	+	hs|17q11.2	BC062632	chr17:29361561-29361		Hs.558901
A_33_P342	2.279422	1.188668	2.279422	up	-0.757602	-0.60671	-0.449334	0.580509	0.449334	0.722516	HYAL4	hyaluronog	FALSE	Homo sapi	23553	GO:003020	1.23E+08	chr7	1.23E+08	+	hs|7q31.32	NM_01226	chr7:12348	NM_01226	Hs.28673
A_23_P565	3.269327	1.708994	3.269327	up	-0.768752	-0.890836	-0.893136	0.768751	1.000478	0.805029	VIT	vitrin	FALSE	Homo sapi	5212	GO:003019	37041825	chr2	37041766	+	hs|2p22.2	NM_05327	chr2:37041	NM_05327	Hs.137415
A_22_P000	2.046307	1.033022	2.046307	up	-0.599421	-0.525624	-0.349733	0.551111	0.349733	0.723444	lnc-SERHL2	lnc-SERHL2	FALSE	LNCipedia lincRNA (lnc-SERHL2-3),			43116813	chr22	43116754	+	hs|22q13.2		chr22:43116754-43116813		
A_23_P121	2.811911	1.491551	2.811911	up	-0.643446	-0.643492	-0.409539	1.22582	0.409539	1.142815	SNCB	synuclein, l	FALSE	Homo sapi	6620	GO:000485	1.76E+08	chr5	1.76E+08	-	hs|5q35.2	NM_00100	chr5:17604	NM_00100	Hs.90297
A_24_P347	2.038596	1.027576	2.038596	up	-0.68143	-0.678189	-0.347258	0.434097	0.347258	0.594496	CTCF	CCCTC-binc	FALSE	Homo sapi	10664	GO:000551	67672173	chr16	67672114	+	hs|16q22.1	NM_00656	chr16:6767	NM_00656	Hs.368367
A_33_P340	2.335996	1.224038	2.335996	up	-0.639324	-0.606496	-0.486448	0.486448	0.925803	0.527593	RMRP	RNA compo	FALSE	Homo sapi	6023		35657998	chr9	35657939	-	hs|9p13.3	NR_003051	chr9:35657	NR_003051	Hs.587502
A_33_P333	2.284824	1.192083	2.284824	up	-0.400701	-0.544328	-0.342762	0.890681	0.342762	1.055015	GPSM1	G-protein s	FALSE	Homo sapi	26086	GO:005079	1.39E+08	chr9	1.39E+08	+	hs|9q34.3	NM_00114	chr9:13925	NM_00114	Hs.239370
A_22_P000	2.091573	1.064588	2.091573	up	-0.424821	-0.641588	-0.617454	0.474683	0.424821	0.610399	LOC102725	uncharacte	FALSE	PREDICTED	1.03E+08		31508240	chr15	31508181	+	hs|15q13.3		chr15:3150	XR_430889	
A_33_P327	2.85946	1.515743	2.85946	up	-0.620669	-0.594216	-0.490402	1.171389	0.490402	1.180151	FKBP8	FK506 bind	FALSE	Homo sapi	23770	GO:000041	18642801	chr19	18642742	-	hs|19p13.1	NM_01218	chr19:1864	NM_01218	Hs.173464
A_23_P354	2.48615	1.313913	2.48615	up	-0.70706	-0.569536	-0.606871	0.723988	0.569536	0.76475	PCDHGA8	protocadhe	FALSE	Homo sapi	9708	GO:000588	1.41E+08	chr5	1.41E+08	+	hs|5q31.3	NM_03208	chr5:14089	NM_03208	Hs.368160
A_23_P136	4.464853	2.158613	4.464853	up	-0.863403	-0.945353	-0.930394	1.435774	0.863402	1.437512	APOD	apolipopro	FALSE	Homo sapi	347	GO:000662	1.95E+08	chr3	1.95E+08	-	hs|3q29	NM_00164	chr3:19530	NM_00164	Hs.522555
A_33_P339	4.169591	2.059906	4.169591	up	-0.942294	-1.076949	-0.91219	1.103125	0.91219	1.232968			FALSE	Homo sapiens cDNA FLJ27292 fis,			2.01E+08	chr1	2.01E+08	-	hs|1q32.1	AK130802	chr1:201474852-201474793		
A_33_P330	6.143344	2.619024	6.143344	up	-1.165943	-1.229386	-1.149008	1.545909	1.149008	1.617821	RNF32	ring finger	FALSE	Homo sapi	140545	GO:000551	1.56E+08	chr7	1.56E+08	+	hs|7q36.3	NM_00118	chr7:15643	NM_00118	Hs.446194
A_23_P208	2.106432	1.074801	2.106432	up	-0.390689	-0.308743	-0.508683	0.811137	0.308743	0.896409	SPTAN1	spectrin, al	FALSE	Homo sapi	6709	GO:000551	1.31E+08	chr9	1.31E+08	+	hs|9q34.11	NM_00312	chr9:13139	NM_00312	Hs.372331
A_22_P000	4.045002	2.016141	4.045002	up	-0.752644	-0.892083	-0.737398	1.428103	0.737398	1.500795	LOC100287	uncharacte	FALSE	Homo sapi	1E+08		1.46E+08	chr8	1.46E+08	+	hs|8q24.3	NR_109770	chr8:14566	NR_109770	Hs.459376
A_22_P000	5.989906	2.582533	5.989906	up	-1.291651	-1.622014	-1.006428	1.308605	1.006428	1.512476	lnc-SNX17-	lnc-SNX17-	FALSE	ALU1_HUMAN (P39188) Alu subfa			27580159	chr2	27580100	+	hs|2p23.3		chr2:27580100-27580159		
A_22_P000	5.069405	2.341816	5.069405	up	-1.254126	-1.16616	-0.920472	1.346407	0.920472	1.417812			FALSE	PREDICTED: Homo sapiens unchara			52242299	chr12	52242240	+	hs|12q13.1	XM_00112	chr12:52242240-52242		Hs.505582
A_22_P000	3.308986	1.726389	3.308986	up	-0.788914	-0.884792	-0.688609	1.023697	0.688609	1.104547	VASH1	vasohibin 1	FALSE	Homo sapi	22846	GO:001059	77248152	chr14	77248093	+	hs|14q24.3	NM_01490	chr14:7724	NM_01490	Hs.525479
A_24_P304	2.152604	1.106083	2.152604	up	-0.482718	-0.618934	-0.567788	0.487822	0.67827	0.482718	AMPD3	adenosine	FALSE	Homo sapi	272	GO:009700	10528622	chr11	10528563	+	hs|11p15.4	NM_00102	chr11:1052	NM_00102	Hs.501890
A_24_P353	2.052259	1.037213	2.052259	up	-0.530275	-0.437742	-0.58257	0.581344	0.437742	0.541968	GALNT2	polypeptid	FALSE	Homo sapi	2590	GO:004847	2.3E+08	chr1	2.3E+08	+	hs|1q42.13	NM_00448	chr1:23041	NM_00448	Hs.743964
A_24_P763	2.916853	1.544413	2.916853	up	-0.744253	-0.740021	-0.727844	0.826687	0.727845	0.866589	EEF1A1	eukaryotic	FALSE	Homo sapi	1915	GO:000551	74227853	chr6	74227794	-	hs|6q13	NM_00140	chr6:74227	NM_00140	Hs.535192
A_23_P273	4.485562	2.165289	4.485562	up	-0.944986	-1.146774	-0.98732	1.104528	1.367272	0.944986	COLEC12	collectin su	FALSE	Homo sapi	81035	GO:000558	319757	chr18	319698	-	hs|18p11.3	NM_13038	chr18:3197	NM_13038	Hs.464422
A_33_P326	2.266331	1.180359	2.266331	up	-0.442032	-0.646184	-0.741801	0.627317	0.442032	0.64171	LIMS3L	LIM and se	FALSE	Homo sapi	1E+08	GO:000827	1.11E+08	chr2	1.11E+08	+	hs|2q13	NM_00120	chr2:11065	NM_00120	Hs.735995
A_33_P332	4.674129	2.224697	4.674129	up	-0.930397	-1.114726	-1.016489	1.327661	0.930397	1.354424	CELSR1	cadherin, E	FALSE	Homo sapi	9620	GO:000173	46760146	chr22	46760087	-	hs|22q13.3	NM_01424	chr22:4676	NM_01424	Hs.252387
A_33_P335	2.576544	1.365437	2.576544	up	-0.594236	-0.707488	-0.886746	0.707212	0.594236	0.606394	DUSP8	dual specifi	FALSE	Homo sapi	1850	GO:000018	1575341	chr11	1575282	-	hs|11p15.5	NM_00442	chr11:1575	NM_00442	Hs.41688
A_22_P000	2.619622	1.389359	2.619622	up	-1.345436	-0.448982	-0.696474	0.448982	0.633641	0.594561	lnc-CENPV-	lnc-CENPV-	FALSE	Q7ZU62_BRARE (Q7ZU62) Zgc:661			16285964	chr17	16285905	-	hs|17p11.2		chr17:16285964-16285905		
A_22_P000	4.249193	2.087189	4.249193	up	-1.067085	-0.948292	-0.898126	1.201805	0.898126	1.248134	LOC101927	uncharacte	FALSE	PREDICTED	1.02E+08		1.14E+08	chr5	1.14E+08	-	hs|5q22.3	XR_251845	chr5:11383	XR_251845	Hs.160027
A_33_P337	2.740208	1.454285	2.740208	up	-0.783903	-0.747335	-0.671924	0.684064	0.803706	0.671924			FALSE				1.03E+08	chr7	1.03E+08	+	hs|7q22.1		chr7:102989031-102989090		
A_19_P003	4.792755	2.260855	4.792755	up	-1.46103	-1.373752	-0.529637	0.892578	0.529637	1.99593	LOC100294	uncharacte	FALSE	Homo sapi	1E+08		32862391	chr6	32862332	+	hs|6p21.32	NR_037178	chr6:32862	NR_037178	Hs.559456
A_23_P217	2.117359	1.082266	2.117359	up	-0.418491	-0.973685	-0.547006	0.418491	0.453247	0.435879	ALAD	aminolevul	FALSE	Homo sapi	210	GO:003279	1.16E+08	chr9	1.16E+08	-	hs|9q32	NM_00003	chr9:11614	NM_00003	Hs.1227
A_21_P001	2.472829	1.306162	2.472829	up	-0.796379	-0.76597	-0.163445	0.845906	0.163445	1.183341	MSH6	mutS homc	FALSE	mutS homc	2956	GO:000551	47935442	chr2	47935383	+	hs|2p16.3		chr2:47935383-47935442		
A_21_P000	3.679402	1.879471	3.679402	up	-0.880118	-0.925007	-0.878853	0.878853	1.092828	0.982754	SNORD87	small nucle	FALSE	Homo sapi	641648		67834768	chr8	67834709	-	hs|8q13.1	NR_002598	chr8:67834	NR_002598	
A_23_P169	12.06832	3.593154	12.06832	up	-1.881113	-1.687516	-2.019929	1.723881	1.687516	1.779506	ORM1	orosomucc	FALSE	Homo sapi	5004	GO:000551	1.17E+08	chr9	1.17E+08	+	hs|9q32	NM_00060	chr9:11708	NM_00060	Hs.522356
A_33_P327	5.975966	2.579172	5.975966	up	-1.465977	-1.243344	-1.192679	1.216949	1.192679	1.425888	TMEM151E	transmemb	FALSE	Homo sapi	441151	GO:001602	44244264	chr6	44244205	+	hs|6p21.1	NM_00113	chr6:44244	NM_00113	Hs.632851
A_21_P000	3.414409	1.771636	3.414409	up	-1.11842	-0.814314	-0.850264	0.872515	0.814314	0.84508	SNORD10	small nucle	FALSE	Homo sapi	652966		7480276	chr17	7480217	+	hs|17p13.1	NR_002604	chr17:7480	NR_002604	Hs.742526
A_33_P340	2.139889	1.097536	2.139889	up	-0.437686	-0.610116	-0.632434	0.546005	0.437686	0.628683	MAT2A	methionine	FALSE	Homo sapi	4144	GO:000551	85771237	chr2	85771178	+	hs|2p11.2	NM_00591	chr2:85771	NM_00591	Hs.516157
A_22_P000	3.333721	1.737133	3.333721	up	-0.764759	-0.760059	-0.69626	1.208624	0.69626	1.085438	lnc-SNX20-	lnc-SNX20-	FALSE	BX109892 Soares_testis_NHT Hom			50817831	chr16	50817772	-	hs|16q12.1	BX109892	chr16:50817831-50817		Hs.599419
A_23_P141	2.501933	1.323043	2.501933	up	-0.918289	-0.657689	-0.495186	0.495186	0.893162	0.509618	CHST4	carbohydra	FALSE	Homo sapi	10164	GO:000580	71572337	chr16	71572278	+	hs|16q22.2	NM_00576	chr16:7157	NM_00576	Hs.251383
A_22_P000	2.816218	1.493759	2.816218	up	-0.760849	-0.748977	-0.604266	0.82649	0.604265	0.93643	lnc-TMEM1	lnc-TMEM1	FALSE	Homo sapiens cDNA FLJ26083 fis,			48290568	chr12	48290509	+	hs|12q13.1	AK129594	chr12:48290509-48290568		
A_33_P328	3.140818	1.651141	3.140818	up	-0.672723	-1.08794	-0.91006	0.672723	0.830313	0.779661	SLC39A8	solute carri	FALSE	Homo sapi	64116	GO:000588	1.03E+08	chr4	1.03E+08	-	hs|4q24	NM_00113	chr4:10317	NM_00113	Hs.288034
A_33_P327	2.102117	1.071843	2.102117	up	-0.419655	-0.379035	-0.348145	0.825195	0.348145	0.895352	KMT2E-AS1	KMT2E ant	FALSE	Homo sapi	1E+08		1.05E+08	chr7	1.05E+08	-	hs|7q22.3	NR_024586	chr7:10465	NR_024586	Hs.631150
A_33_P321	2.607708	1.382782	2.607708	up	-0.639938	-0.741456	-0.546788	0.546788	0.956561	0.716816	ACSL5	acyl-CoA sy	FALSE	Homo sapi	51703	GO:200123	1.14E+08	chr10	1.14E+08	+	hs|10q25.2	NM_20338	chr10:1141	NM_20338	Hs.11638
A_24_P348	3.362337	1.749464	3.362337	up	-0.712552	-1.038663	-0.873024	0.941572	0.712551	0.970031			FALSE	Homo sapiens chromosome 18 op			43915002	chr18	43914943	+	hs|18q21.1	BC093850	chr18:43914943-43915		Hs.501114

A_33_P339	2.325124	1.217307	2.325124	up	-0.723073	-0.605622	-0.48445	0.641388	0.48445	0.71294	LINC00963	long interg	FALSE	Homo sapi	1.01E+08		1.32E+08	chr9	1.32E+08	+	hs|9q34.11	NR_03895	chr9:1322	NR_03895	Hs.529860
A_33_P345	5.351288	2.419886	5.351288	up	-1.403081	-1.044483	-1.225753	1.237767	1.044483	1.304092			FALSE	PREDICTED: Homo sapiens glutenin, high molecular weight subunit DX5-like (LOC100996433)								XM_006710179		XM_00671	Hs.701992
A_33_P329	3.161194	1.66047	3.161194	up	-0.739105	-0.639476	-0.899759	0.937749	0.639476	1.125845			FALSE				47093168	chr7	47093109	+	hs|7p12.3		chr7:47093109-47093168		
A_33_P321	8.020324	3.00366	8.020324	up	-1.453301	-1.693292	-1.267147	1.561528	1.267146	1.768567	PPA2	pyrophospl	FALSE	pyrophospl	27068	GO:001046	1.06E+08	chr4	1.06E+08	-	hs|4q24	AK299501	chr4:106394684-10639		Hs.654957
A_33_P342	2.256037	1.173791	2.256037	up	-0.345831	-0.576608	-0.500892	0.813457	0.345831	0.938754	KIRREL2	kin of IRRE	FALSE	Homo sapi	84063	GO:001633	36357394	chr19	36357335	+	hs|19q13.1	NM_19918	chr19:3635	NM_19918	Hs.145729
A_23_P662	2.367553	1.243397	2.367553	up	-0.546699	-0.57354	-0.594321	0.617503	0.851428	0.546699	MT1M	metallothi	FALSE	Homo sapi	4499	GO:007129	56667869	chr16	56667810	+	hs|16q12.2	NM_17687	chr16:5666	NM_17687	Hs.647370
A_22_P000	2.519606	1.333198	2.519606	up	-1.144382	-0.469606	-0.712361	0.469606	0.578992	0.624648			FALSE	BX100836 Soares_NFL_T_GBC_S1			1.74E+08	chr5	1.74E+08	+	hs|5q35.2		chr5:174253130-17425		Hs.738217
A_23_P164	3.000458	1.585183	3.000458	up	-0.825256	-0.928968	-0.587558	0.81948	0.587558	1.006729	LRP3	low density	FALSE	Homo sapi	4037	GO:000590	33698395	chr19	33698336	+	hs|19q13.1	NM_00233	chr19:3369	NM_00233	Hs.515340
A_23_P787	2.398316	1.262022	2.398316	up	-0.437712	-0.632554	-0.593091	0.875688	0.437712	0.809307	MYH14	myosin, he	FALSE	Homo sapi	79784	GO:000551	50813376	chr19	50813317	+	hs|19q13.3	NM_00107	chr19:5081	NM_00107	Hs.467142
A_22_P000	2.415545	1.272348	2.415545	up	-0.739885	-0.618942	-0.587734	0.587734	0.665118	0.617632	lnc-IDH2-1	lnc-IDH2-1:	FALSE	Homo sapiens cDNA FLJ31419 fis,			90618141	chr15	90618082	-	hs|15q26.1	AK055981	chr15:90618141-90618		Hs.655249
A_22_P000	2.146402	1.10192	2.146402	up	-0.798151	-0.596335	-0.301618	0.66657	0.301618	0.641468	MFI2-AS1	MFI2 antise	FALSE	Homo sapi	1.01E+08		1.97E+08	chr3	1.97E+08	+	hs|3q29	NR_03828	chr3:19673	NR_03828	Hs.666451
A_24_P460	2.558567	1.355336	2.558567	up	-0.605338	-0.684973	-0.61122	0.631408	0.927729	0.605338	SLC6A6	solute carri	FALSE	Homo sapi	6533	GO:000652	14530505	chr3	14530446	+	hs|3p25.1	NM_00304	chr3:1453C	NM_00304	Hs.529488
A_33_P324	11.03699	3.464275	11.03699	up	-1.66486	-1.657941	-1.703043	1.70942	1.99962	1.657941	KCTD12	potassium	FALSE	Homo sapi	115207	GO:004323	77454410	chr13	77454351	-	hs|13q22.3	NM_13844	chr13:7745	NM_13844	Hs.644125
A_22_P000	2.233899	1.159564	2.233899	up	-0.832989	-0.888189	-0.067711	0.440198	0.067711	1.181894	lnc-CCDC1:	lnc-CCDC1:	FALSE	LNCipedia lincRNA (lnc-CCDC113-1			58231092	chr16	58231033	+	hs|16q21		chr16:58231033-58231092		
A_33_P326	3.265366	1.707245	3.265366	up	-0.724014	-0.940485	-0.728856	0.741746	1.262621	0.724014	SBSN	suprabasin	FALSE	Homo sapi	374897	GO:000815	36014331	chr19	36014272	-	hs|19q13.1	NM_00116	chr19:3601	NM_00116	Hs.433484
A_23_P215	3.944193	1.97973	3.944193	up	-0.813461	-0.775651	-0.988156	1.216897	0.775651	1.369376	CLU	clusterin	FALSE	Homo sapi	1191	GO:190022	27457462	chr8	27457403	-	hs|8p21.1	NM_00183	chr8:27457	NM_00183	Hs.436657
A_24_P378	4.190848	2.067242	4.190848	up	-0.950659	-1.066217	-0.85447	1.185666	0.85447	1.290244	GPR150	G protein-c	FALSE	Homo sapi	285601	GO:000718	94957261	chr5	94957202	+	hs|5q15	NM_19924	chr5:94957	NM_19924	Hs.143315
A_33_P333	2.369159	1.244375	2.369159	up	-0.5451	-0.590947	-0.570232	0.724113	0.5451	0.757633	DIABLO	diablo, IAP-	FALSE	Homo sapi	56616	GO:000551	1.23E+08	chr12	1.23E+08	-	hs|12q24.3	NM_00127	chr12:1227	NM_00127	Hs.169611
A_23_P963	3.338642	1.739261	3.338642	up	-0.830444	-0.915338	-0.83424	0.830443	0.943724	0.863595	SRPX	sushi-repea	FALSE	Homo sapi	8406	GO:003497	38008772	chrX	38008713	-	hs|Xp11.4	NM_00630	chrX:38008	NM_00630	Hs.15154
A_33_P337	2.12978	1.090704	2.12978	up	-0.634454	-0.320844	-0.695895	0.689679	0.320844	0.610398	HNRNPLL	heterogene	FALSE	Homo sapi	92906	GO:000639	38809115	chr2	38809056	-	hs|2p22.1	NM_13839	chr2:38809	NM_13839	Hs.445497
A_22_P000	6.028637	2.591832	6.028637	up	-0.907938	-1.348572	-1.542879	1.544794	0.907938	1.523376	FAM229A	family with	FALSE	Homo sapi	1E+08		32827390	chr1	32827331	-	hs|1p35.1	NM_00116	chr1:32827	NM_00116	Hs.660539
A_33_P321	3.550687	1.828098	3.550687	up	-0.763278	-1.076971	-0.86223	0.913453	0.763278	1.105084	HMGA2	high mobili	FALSE	Homo sapi	8091	GO:000551	66219160	chr12	66219101	+	hs|12q14.3	NM_00130	chr12:6621	NM_00130	Hs.505924
A_23_P137	2.377444	1.249412	2.377444	up	-0.31895	-0.5458	-0.596328	0.94236	0.31895	1.025847	NYX	nyctalopin	FALSE	Homo sapi	60506	GO:000562	41334629	chrX	41334570	+	hs|Xp11.4	NM_02256	chrX:41334	NM_02256	Hs.302019
A_24_P147	6.522079	2.705332	6.522079	up	-1.051848	-1.577999	-1.293671	1.604248	1.051848	1.536382	PRR36	proline rich	FALSE	Homo sapi	80164		7934686	chr19	7934627	-	hs|19p13.2	NM_00119	chr19:7934	NM_00119	Hs.288540
A_24_P539	3.913522	1.968468	3.913522	up	-1.088201	-1.153305	-0.907413	0.907413	0.92948	0.919591	GLUL	glutamate-	FALSE	Homo sapi	2752	GO:000828	1.82E+08	chr1	1.82E+08	-	hs|1q25.3	NM_00206	chr1:18235	NM_00206	Hs.518525
A_24_P196	2.253027	1.171865	2.253027	up	-0.276232	-0.277406	-0.301898	1.202592	0.276232	1.181235	TLN1	talin 1	FALSE	Homo sapi	7094	GO:000551	35697825	chr9	35697766	-	hs|9p13.3	NM_00628	chr9:35697	NM_00628	Hs.471014
A_22_P000	3.148833	1.654817	3.148833	up	-0.752338	-0.821084	-0.915252	0.832201	0.89124	0.752338	lnc-SLC7A5	lnc-SLC7A5	FALSE	LNCipedia lincRNA (lnc-SLC7A5-1),			87926605	chr16	87926546	-	hs|16q24.2		chr16:87926605-87926546		
A_33_P333	3.007793	1.588706	3.007793	up	-1.115146	-0.907895	-0.509647	0.814135	0.509647	0.909646	LOC10012S	uncharacte	FALSE	Homo sapi	1E+08		1.13E+08	chr1	1.13E+08	+	hs|1p13.2	AK092791	chr1:112939189-11293		Hs.744100
A_21_P000	4.631744	2.211556	4.631744	up	-0.902157	-0.804162	-0.879768	1.489778	0.804162	1.75464	lnc-EPHA6-	lnc-EPHA6-	FALSE	LNCipedia lincRNA (lnc-EPHA6-1),			96336969	chr3	96336910	+	hs|3q11.2		chr3:96336910-96336969		
A_23_P106	10.26382	3.359496	10.26382	up	-1.811148	-1.531784	-1.74912	1.531784	1.772824	1.681829	AQP9	aquaporin	FALSE	Homo sapi	366	GO:000588	58477943	chr15	58477884	+	hs|15q21.3	NM_02098	chr15:5847	NM_02098	Hs.104624
A_23_P601	2.934128	1.552932	2.934128	up	-0.958952	-0.727044	-0.703119	0.766035	0.800527	0.703119	DEPTOR	DEP domai	FALSE	Homo sapi	64798	GO:200123	1.21E+08	chr8	1.21E+08	+	hs|8q24.12	NM_02278	chr8:12106	NM_02278	Hs.112981
A_23_P941	3.222228	1.688059	3.222228	up	-0.783174	-0.774615	-0.976154	0.822591	0.933027	0.774615	ACO1	aconitase 1	FALSE	Homo sapi	48	GO:000551	32450604	chr9	32450545	+	hs|9p21.1	NM_00219	chr9:3245C	NM_00219	Hs.567229
A_24_P357	2.124926	1.087413	2.124926	up	-0.455524	-0.455301	-0.574647	0.650129	0.671337	0.455301	RPL21	ribosomal	FALSE	Homo sapi	6144	GO:001046	27829389	chr13	27828370	+	hs|13q12.2	NM_00098	chr13:2782	NM_00098	Hs.381123
A_23_P340	2.79776	1.484272	2.79776	up	-0.610418	-0.648382	-0.415122	1.172889	0.415122	1.190884	G6PD	glucose-6-p	FALSE	Homo sapi	2539	GO:005066	1.54E+08	chrX	1.54E+08	-	hs|Xq28	NM_00040	chrX:15376	NM_00040	Hs.461047
A_33_P327	4.384447	2.132395	4.384447	up	-1.134883	-1.067473	-0.755777	1.414147	0.755777	1.269126	TUSC1	tumor supp	FALSE	Homo sapi	286319		25677740	chr9	25677681	-	hs|9p21.2	NM_00100	chr9:25677	NM_00100	Hs.26268
A_23_P309	2.636342	1.398538	2.636342	up	-0.896067	-0.60633	-0.756198	0.60633	0.71214	0.618548	HIST2H2AA	histone clu	FALSE	Homo sapi	723790	GO:000815	1.5E+08	chr1	1.5E+08	+	hs|1q21.2	NM_00104	chr1:14982	NM_00104	Hs.745563
A_32_P133	2.683191	1.42395	2.683191	up	-0.635156	-0.721241	-0.76067	0.698433	0.821193	0.635156	TUSC1	tumor supp	FALSE	Homo sapi	286319		25677283	chr9	25677224	-	hs|9p21.2	NM_00100	chr9:25677	NM_00100	Hs.26268
A_21_P000	2.622518	1.390953	2.622518	up	-0.633521	-0.637014	-0.368953	1.008234	0.368953	1.156184	lnc-ERICH1	lnc-ERICH1	FALSE	LNCipedia lincRNA (lnc-ERICH1-5),			1376610	chr8	1376551	-	hs|8p23.3		chr8:1376610-1376551		
A_24_P675	2.04291	1.030625	2.04291	up	-0.518576	-0.458604	-0.373394	0.646781	0.373394	0.721128	VGLL3	vestigial-lik	FALSE	Homo sapi	389136	GO:000635	87017874	chr3	87017815	-	hs|3p12.1	NM_01620	chr3:87017	NM_01620	Hs.744116
A_21_P001	-4.39454	-2.135712	4.39454	down	0.868083	0.970074	0.916782	-1.356376	-0.868083	-1.42774	XLOC_l2_009328		FALSE	BROAD Institute lincRNA (XLOC_l2			24663060	chr22	24663001	+	hs|22q11.23		chr22:24663001-24663060		
A_23_P132	-2.233822	-1.159514	2.233822	down	0.349404	0.417137	0.400708	-0.899587	-0.349404	-1.062304	GPR155	G protein-c	FALSE	Homo sapi	151556	GO:003555	1.75E+08	chr2	1.75E+08	-	hs|2q31.1	NM_00103	chr2:17529	NM_00103	Hs.516604
A_32_P429	-2.443131	-1.288731	2.443131	down	0.671666	0.419626	0.598248	-0.990047	-0.766982	-0.419626	NKD1	naked cutic	FALSE	Homo sapi	85407	GO:000175	50667944	chr16	50667885	+	hs|16q12.1	NM_03311	chr16:5066	NM_03311	Hs.187578
A_23_P983	-2.027239	-1.019516	2.027239	down	0.427534	0.488261	0.496918	-0.65363	-0.427534	-0.564672	BIRC3	baculoviral	FALSE	Homo sapi	330	GO:000551	1.02E+08	chr11	1.02E+08	+	hs|11q22.2	NM_00116	chr11:1022	NM_00116	Hs.127799
A_23_P125	-3.906282	-1.965796	3.906282	down	0.823878	0.943618	0.848021	-1.198993	-0.823878	-1.259	CNN1	calponin 1,	FALSE	Homo sapi	1264	GO:000551	11661064	chr19	11661005	+	hs|19p13.2	NM_00129	chr19:1166	NM_00129	Hs.465929
A_32_P245	-2.04298	-1.030675	2.04298	down	0.407155	0.448796	0.437059	-0.761042	-0.407155	-0.630819	TMTC1	transmemb	FALSE	Homo sapi	83857	GO:000573	29653922	chr12	29653863	-	hs|12p11.2	NM_17586	chr12:2965	NM_17586	Hs.401954
A_21_P000	-3.53324	-1.820992	3.53324	down	0.914153	0.973734	0.835536	-0.850212	-0.835536	-1.053805	SNORD114	small nucle	FALSE	Homo sapi	767603		1.01E+08	chr14	1.01E+08	+	hs|14q32.3	NR_00321	chr14:1014	NR_003216	
A_33_P329	-2.697474	-1.431609	2.697474	down	0.672566	0.603605	0.800505	-0.709581	-0.603605	-0.904965	ARSJ	arylsulfatas	FALSE	Homo sapi	79642	GO:000666	1.15E+08	chr4	1.15E+08	-	hs|4q26	NM_02459	chr4:11482	NM_02459	Hs.22895
A_23_P122	-2.147049	-1.102355	2.147049	down	0.413953	0.45137	0.444623	-0.826268	-0.413953	-0.756898	INHBA	inhibin, bet	FALSE	Homo sapi	3624	GO:000551	41729305	chr7	41729246	-	hs|7p14.1	NM_00219	chr7:41729	NM_00219	Hs.583348
A_33_P339	-2.964463	-1.567771	2.964463	down	0.835189	0.74559	0.85493	-0.767271	-0.74559	-0.754744	CCDC85A	coiled-coil	FALSE	Homo sapi	114800		56612701	chr2	56612642	+	hs|2p16.1	NM_00108	chr2:56612	NM_00108	Hs.117136
A_23_P202	-2.154806	-1.107558	2.154806	down	0.639425	0.533522	0.488292	-0.598742	-0.488292	-0.574402	KIAA1598	KIAA1598	FALSE	Homo sapi	57698	GO:000741	1.19E+08	chr10	1.19E+08	-	hs|10q25.3	NM_01833	chr10:1186	NM_01833	Hs.501140
A_23_P291	-2.201389	-1.138414	2.201389	down	0.521943	0.551366	0.582164	-0.580857	-0.65697	-0.521943	GP1BB	glycoprotei	FALSE	Homo sapi	2812	GO:000551	19712242	chr22	19712183	+	hs|22q11.2	NM_00040	chr22:1971	NM_00040	Hs.283743
A_23_P828	-2.095029	-1.066971	2.095029	down	0.359316	0.385135	0.347858	-0.872046	-0.347858	-0.888698	PLAT	plasminoge	FALSE	Homo sapi	5327	GO:000551	42032871	chr8	42032812	-	hs|8p11.21	NM_00093	chr8:42032	NM_00093	Hs.491582
A_23_P774	-2.03864	-1.027607	2.03864	down	0.527876	0.464862	0.445263	-0.60694	-0.445263	-0.592618	CPPED1	calcineurin	FALSE	Homo sapi	55313	GO:000573	12757607	chr16	12757548	-	hs|16p13.1	NM_01834	chr16:1275	NM_01834	Hs.460002
A_23_P209	-2.211119	-1.144777	2.211119	down	0.386059	0.50647	0.422828	-0.872182	-0.38606	-0.860732	KLHL29	kelch-like f	FALSE	Homo sapi	114818		23931368	chr2	23931309	+	hs|2p24.1	NM_05292	chr2:23931	NM_05292	Hs.130593
A_33_P327	-2.162321	-1.112581	2.162321	down	0.55768	0.457539	0.527488	-0.62914	-0.708357	-0.457539	CASP3	caspase 3,	FALSE	Homo sapi	836	GO:000551	1.86E+08	chr4	1.86E+08	-	hs|4q35.1	NM_00434	chr4:18555	NM_00434	Hs.141125
A_23_P242	-2.108517	-1.076229	2.108517	down	0.736089	0.55627	0.3165	-0.447962	-0.3165	-0.855367	TBC1D12	TBC1 doma	FALSE	Homo sapi	23232	GO:003285	96295374	chr10	96295315	+	hs|10q23.3	NM_01518	chr10:9629	NM_01518	Hs.500598
A_24_P127	-2.051515	-1.036689	2.051515	down	0.586088	0.48945	0.415156	-0.415156	-0.723494	-0.480725	BCR	breakpoint	FALSE	Homo sapi	613	GO:000551	23653900	chr22	23652578	+	hs|22q11.2	NM_00432	chr22:2365	NM_00432	Hs.517461
A_32_P744	-4.5108	-2.173383	4.5108	down	0.903215	0.922447	0.908915	-1.455689	-0.903214	-1.426668	C11orf96	chromoson	FALSE	Homo sapi	387763		43965397	chr11	43965338	+	hs|11p11.2	NM_00114	chr11:4396	NM_00114	Hs.530443

A_23_P133	-2.499471	-1.321623	2.499471	down	0.542808	0.547944	0.621946	-0.542808	-1.038331	-0.671033	SLC29A1	solute carri	FALSE	Homo sapi	2030	GO:001586	44201834	chr6	44201775	+	hs|6p21.1	NM_00107	chr6:44201	NM_00107	Hs.25450
A_22_P000	-2.015942	-1.011454	2.015942	down	0.410513	0.489972	0.437157	-0.560652	-0.410513	-0.725555	LOC10192€	uncharacte	FALSE	PREDICTED	1.02E+08		42301075	chr17	42301019	+	hs|17q21.3	XR_243706	chr17:4230	XR_243706	
A_23_P140	-2.034315	-1.024543	2.034315	down	0.439916	0.448675	0.445392	-0.59982	-0.439916	-0.699911	AMIGO2	adhesion m	FALSE	Homo sapi	347902	GO:000551	47470990	chr12	47470931	-	hs|12q13.1	NM_18184	chr12:4747	NM_18184	Hs.121520
A_23_P324	-2.328429	-1.219357	2.328429	down	0.587371	0.484201	0.537522	-0.679409	-0.484201	-0.885365	GPRC5B	G protein-c	FALSE	Homo sapi	51704	GO:000166	19870688	chr16	19870629	-	hs|16p12.3	NM_01623	chr16:1987	NM_01623	Hs.148685
A_23_P120	-2.004361	-1.003142	2.004361	down	0.482537	0.513717	0.521341	-0.521087	-0.482537	-0.488209	ACSS1	acyl-CoA sy	FALSE	Homo sapi	84532	GO:000551	24987088	chr20	24987029	-	hs|20p11.2	NM_03250	chr20:2498	NM_03250	Hs.529353
A_23_P677	-3.072704	-1.619509	3.072704	down	0.714083	0.792402	0.80467	-0.954643	-0.714083	-0.878645	LMCD1	LIM and cy	FALSE	Homo sapi	29995	GO:003101	8609747	chr3	8609688	+	hs|3p26.1	NM_01458	chr3:86096	NM_01458	Hs.475353
A_23_P988	-2.220399	-1.150819	2.220399	down	0.491862	0.509779	0.48606	-0.821931	-0.48606	-0.656764	CDK2	cyclin-depe	FALSE	Homo sapi	1017	GO:000551	56365848	chr12	56365789	+	hs|12q13.2	NM_00179	chr12:5636	NM_00179	Hs.19192
A_24_P188	-2.214232	-1.146807	2.214232	down	0.663123	0.659424	0.455075	-0.631119	-0.455075	-0.576605	VPS18	vacuolar pr	FALSE	Homo sapi	57617	GO:000551	41195945	chr15	41195886	+	hs|15q15.1	NM_02085	chr15:4119	NM_02085	Hs.23876
A_23_P648	-3.562246	-1.832787	3.562246	down	0.990776	0.906654	0.768853	-0.768853	-1.204654	-0.858571	OAS1	2'-5'-oligoa	FALSE	Homo sapi	4938	GO:000372	1.13E+08	chr12	1.13E+08	+	hs|12q24.1	NM_00253	chr12:1133	NM_00253	Hs.524760
A_23_P333	-2.119906	-1.084001	2.119906	down	0.437085	0.429392	0.417028	-0.7881	-0.417027	-0.76337	ADRA1B	adrenocept	FALSE	Homo sapi	147	GO:000588	1.59E+08	chr5	1.59E+08	+	hs|5q33.3	NM_00067	chr5:15939	NM_00067	Hs.368632
A_23_P153	-2.159399	-1.11063	2.159399	down	0.525812	0.566566	0.559744	-0.6058	-0.548156	-0.525812	ICAM1	intercellula	FALSE	Homo sapi	3383	GO:000551	10396358	chr19	10396298	+	hs|19p13.2	NM_00020	chr19:1039	NM_00020	Hs.643447
A_23_P421	-2.017251	-1.01239	2.017251	down	0.831862	0.59069	0.331219	-0.331219	-0.591813	-0.360368	IGF2	insulin-like	FALSE	Homo sapi	3481	GO:000551	2154754	chr11	2154402	-	hs|11p15.5	NM_00061	chr11:2154	NM_00061	Hs.272259
A_23_P345	-2.077026	-1.054519	2.077026	down	0.430393	0.602019	0.455891	-0.430393	-0.81362	-0.431243	ADPRHL2	ADP-ribosy	FALSE	Homo sapi	54936	GO:007145	36558869	chr1	36558810	+	hs|1p34.3	NM_01782	chr1:36558	NM_01782	Hs.18021
A_33_P329	-2.270488	-1.183002	2.270488	down	0.432076	0.617423	0.531679	-0.746105	-0.432076	-0.789648			FALSE				1.39E+08	chr7	1.39E+08	-	hs|7q34		chr7:138734075-138734016		
A_23_P216	-2.828022	-1.499793	2.828022	down	0.83112	0.746094	0.634459	-0.634459	-0.753256	-0.899993	TRIM14	tripartite m	FALSE	Homo sapi	9830	GO:000562	1.01E+08	chr9	1.01E+08	-	hs|9q22.33	NM_01478	chr9:10084	NM_01478	Hs.575631
A_23_P319	-2.071785	-1.050874	2.071785	down	0.449605	0.688607	0.513096	-0.459116	-0.592592	-0.449605	CHST7	carbohydra	FALSE	Homo sapi	56548	GO:000679	46457467	chrX	46457408	+	hs|Xp11.23	NM_01988	chrX:46457	NM_01988	Hs.129955
A_22_P000	-2.005403	-1.003892	2.005403	down	0.351321	0.345528	0.536887	-0.486378	-0.946033	-0.345528	lnc-C16orf4	lnc-C16orf4	FALSE	LNCipedia lincRNA (lnc-C16orf45-1			15778167	chr16	15778108	+	hs|16p13.11		chr16:15778108-15778167		
A_23_P215	-2.18788	-1.129534	2.18788	down	0.480277	0.469847	0.514941	-0.702411	-0.469847	-0.751277	AHR	aryl hydroc	FALSE	Homo sapi	196	GO:000551	17385058	chr7	17384999	+	hs|7p21.1	NM_00162	chr7:17384	NM_00162	Hs.171189
A_33_P337	-2.248071	-1.168687	2.248071	down	0.505378	0.611756	0.602954	-0.694371	-0.505378	-0.586226	DDX58	DEAD (Asp-	FALSE	Homo sapi	23586	GO:000551	32455457	chr9	32455398	-	hs|9p21.1	NM_01431	chr9:32455	NM_01431	Hs.190622
A_21_P000	-3.969361	-1.988907	3.969361	down	0.940253	0.892039	0.920399	-1.031181	-0.892039	-1.29081	SNORD114	small nucle	FALSE	Homo sapi	767599		1.01E+08	chr14	1.01E+08	+	hs|14q32.3	NR_003214	chr14:1014	NR_003214	
A_23_P377	-2.045564	-1.032499	2.045564	down	0.444354	0.408538	0.443038	-0.652267	-0.740761	-0.408538	CIRBP	cold induci	FALSE	cold induci	1153	GO:000551	1274066	chr19	1274007	+	hs|19p13.3	AK128423	chr19:1274007-127406		Hs.618145
A_24_P810	-2.777682	-1.473882	2.777682	down	0.618225	0.886009	0.864798	-0.618225	-0.760589	-0.673799	PPAPDC1A	phosphatid	FALSE	Homo sapi	196051	GO:000588	1.22E+08	chr10	1.22E+08	+	hs|10q26.1	NM_00103	chr10:1223	NM_00103	Hs.40479
A_33_P337	-2.019978	-1.01434	2.019978	down	0.256423	0.291171	0.332773	-1.060964	-0.256423	-0.845264	NAA35	N(alpha)-ac	FALSE	N(alpha)-ac	60560	GO:000551	88601904	chr9	88601845	+	hs|9q21.33	AK056059	chr9:88601845-886019		Hs.436098
A_23_P134	-4.112311	-2.039949	4.112311	down	0.85819	0.855855	0.925633	-1.314478	-0.855855	-1.309837	RNF122	ring finger	FALSE	Homo sapi	79845	GO:000579	33405363	chr8	33405304	-	hs|8p12	NM_02478	chr8:33405	NM_02478	Hs.151237
A_24_P557	-3.132472	-1.647302	3.132472	down	0.818799	0.848617	0.806474	-0.806474	-0.83565	-0.825892	XAF1	XIAP associ	FALSE	Homo sapi	54739	GO:000573	6678878	chr17	6678819	+	hs|17p13.1	NM_01752	chr17:6678	NM_01752	Hs.441975
A_33_P351	-2.582239	-1.368623	2.582239	down	0.693318	0.682818	0.649969	-0.714754	-0.649969	-0.71504	POSTN	periostin, c	FALSE	Homo sapi	10631	GO:003101	38137425	chr13	38137366	-	hs|13q13.3	NM_00647	chr13:3813	NM_00647	Hs.136348
A_23_P152	-2.628813	-1.394411	2.628813	down	0.667257	0.647988	0.597107	-0.747965	-0.925809	-0.597108	IFI35	interferon-	FALSE	Homo sapi	3430	GO:000551	41165181	chr17	41165122	+	hs|17q21.3	NM_00553	chr17:4116	NM_00553	Hs.632258
A_23_P318	-4.440068	-2.150582	4.440068	down	1.074486	1.134773	0.888252	-1.143204	-0.888252	-1.322781	RAB11FIP1	RAB11 fam	FALSE	Homo sapi	80223	GO:004505	37718295	chr8	37718236	-	hs|8p11.23	NM_00100	chr8:37718	NM_00100	Hs.191179
A_23_P240	-2.973574	-1.572198	2.973574	down	0.695287	0.728539	0.731995	-0.921497	-0.695288	-0.943988	IFIT2	interferon-	FALSE	Homo sapi	3433	GO:003209	91068456	chr10	91068397	+	hs|10q23.3	NM_00154	chr10:9106	NM_00154	Hs.437609
A_24_P236	-2.236621	-1.161321	2.236621	down	0.671432	0.479481	0.569099	-0.513686	-0.770783	-0.479481	ENO2	enolase 2 (	FALSE	Homo sapi	2026	GO:000028	7032541	chr12	7032482	+	hs|12p13.3	NM_00197	chr12:7032	NM_00197	Hs.511915
A_23_P410	-2.016999	-1.01221	2.016999	down	0.488729	0.432379	0.550602	-0.608964	-0.432379	-0.523579	ZSWIM6	zinc finger,	FALSE	Homo sapi	57688	GO:004881	60841267	chr5	60841208	+	hs|5q12.1	NM_02092	chr5:60841	NM_02092	Hs.744939
A_32_P616	-3.323651	-1.732769	3.323651	down	0.890017	1.060283	0.728899	-0.825351	-0.964859	-0.728899	PAG1	phosphopr	FALSE	Homo sapi	55824	GO:000551	81880292	chr8	81880233	-	hs|8q21.13	NM_01844	chr8:81880	NM_01844	Hs.266175
A_23_P149	-2.465311	-1.30177	2.465311	down	0.630517	0.660298	0.636802	-0.663499	-0.630517	-0.683676	PLEKHG5	pleckstrin h	FALSE	Homo sapi	57449	GO:005105	6527281	chr1	6527222	-	hs|1p36.31	NM_19868	chr1:65272	NM_19868	Hs.284232
A_24_P353	-2.220424	-1.150836	2.220424	down	0.092833	0.195005	0.094033	-2.860865	-0.116937	-0.092834	GUCD1	guanylyl cy	FALSE	Homo sapi	83606		24936686	chr22	24936627	-	hs|22q11.2	NM_03144	chr22:2493	NM_03144	Hs.9850
A_33_P335	-2.258337	-1.175261	2.258337	down	0.411446	0.436037	0.598355	-0.816802	-0.411446	-0.851695	SUV420H2	suppressor	FALSE	Homo sapi	84787	GO:000551	55859488	chr19	55859429	+	hs|19q13.4	NM_03270	chr19:5585	NM_03270	Hs.590982
A_23_P397	-2.079983	-1.056572	2.079983	down	0.59232	0.534638	0.362834	-0.578588	-0.738502	-0.362834	PAQR4	progestin a	FALSE	Homo sapi	124222	GO:000487	3022845	chr16	3022786	+	hs|16p13.3	NM_15234	chr16:3022	NM_15234	Hs.351474
A_23_P538	-2.450038	-1.292804	2.450038	down	0.64338	0.620268	0.562598	-0.736521	-0.562598	-0.753046	KLF5	Kruppel-lik	FALSE	Homo sapi	688	GO:006057	73651038	chr13	73650979	+	hs|13q22.1	NM_00173	chr13:7365	NM_00173	Hs.508234
A_23_P660	-2.061625	-1.043782	2.061625	down	0.444762	0.387573	0.424041	-0.773506	-0.387573	-0.713889	PRRT2	proline-rich	FALSE	Homo sapi	112476	GO:000960	29826998	chr16	29826939	+	hs|16p11.2	NM_14523	chr16:2982	NM_14523	Hs.655071
A_23_P162	-2.124357	-1.087026	2.124357	down	0.537919	0.521237	0.525094	-0.529663	-0.625929	-0.521237	MCAM	melanoma	FALSE	Homo sapi	4162	GO:006104	1.19E+08	chr11	1.19E+08	-	hs|11q23.3	NM_00650	chr11:1191	NM_00650	Hs.599039
A_33_P333	-2.180371	-1.124574	2.180371	down	0.696426	0.674205	0.423384	-0.423384	-0.465663	-0.69066	TRPC6	transient re	FALSE	Homo sapi	7225	GO:000551	1.01E+08	chr11	1.01E+08	-	hs|11q22.1	NM_00462	chr11:1013	NM_00462	Hs.159003
A_33_P340	-2.249097	-1.169346	2.249097	down	0.467558	0.562104	0.452559	-0.811229	-0.452559	-0.76203	TAB2	TGF-beta a	FALSE	Homo sapi	23118	GO:000551	1.5E+08	chr6	1.5E+08	+	hs|6q25.1	NM_01509	chr6:14970	NM_01509	Hs.269775
A_33_P325	-2.345132	-1.229669	2.345132	down	0.641927	0.616916	0.566993	-0.566993	-0.673379	-0.622802	XAF1	XIAP associ	FALSE	Homo sapi	54739	GO:000573	6674202	chr17	6674143	+	hs|17p13.1	NM_01752	chr17:6674	NM_01752	Hs.441975
A_32_P196	-2.824767	-1.498132	2.824767	down	1.008688	0.900666	0.555157	-0.812483	-0.555157	-0.662246	FGF7	fibroblast g	FALSE	Homo sapi	2252	GO:000828	49778611	chr15	49778552	+	hs|15q21.2		chr15:4977	NM_00200	Hs.567268
A_21_P000	-2.53362	-1.3412	2.53362	down	0.454722	0.642514	0.699923	-0.73054	-0.454722	-1.041179	SNORD113	small nucle	FALSE	Homo sapi	767563		1.01E+08	chr14	1.01E+08	+	hs|14q32.2	NR_003231	chr14:1013	NR_003231	
A_33_P323	-2.08809	-1.062184	2.08809	down	0.596821	0.506933	0.476088	-0.476088	-0.620906	-0.509715	GOLGA6L9	golgin A6 fa	FALSE	Homo sapi	440295		83102942	chr15	83102883	+	hs|15q25.2	NM_19818	chr15:8310	NM_19818	Hs.498322
A_24_P127	-2.028201	-1.020201	2.028201	down	0.370099	0.503921	0.450799	-0.478062	-0.887622	-0.370099	SPACA6P	sperm acro	FALSE	Homo sapi	147650		52206548	chr19	52206489	+	hs|19q13.4	NR_024330	chr19:5220	NR_024330	Hs.467174
A_33_P340	-2.045547	-1.032487	2.045547	down	0.602432	0.504965	0.390561	-0.434868	-0.390561	-0.774074	FZD6	frizzled clas	FALSE	Homo sapi	8323	GO:004281	1.04E+08	chr8	1.04E+08	+	hs|8q22.3	NM_00350	chr8:10434	NM_00350	Hs.591863
A_24_P161	-2.676141	-1.420154	2.676141	down	0.711257	0.682577	0.646239	-0.759348	-0.646238	-0.814803	PARP14	poly (ADP-r	FALSE	Homo sapi	54625	GO:000635	1.22E+08	chr3	1.22E+08	+	hs|3q21.1	NM_01755	chr3:12243	NM_01755	Hs.518203
A_23_P110	-2.569907	-1.361716	2.569907	down	0.666328	0.52518	0.726503	-0.919521	-0.722436	-0.52518	CSF1R	colony stim	FALSE	Homo sapi	1436	GO:000828	1.49E+08	chr5	1.49E+08	-	hs|5q32	NM_00521	chr5:14943	NM_00521	Hs.586219
A_21_P000	-3.443036	-1.783681	3.443036	down	0.958883	0.98643	0.814399	-0.908265	-0.868667	-0.814399	SNORD114	small nucle	FALSE	Homo sapi	767585		1.01E+08	chr14	1.01E+08	+	hs|14q32.3	NR_003201	chr14:1014	NR_003201	
A_33_P323	-2.044701	-1.03189	2.044701	down	0.53657	0.397757	0.389638	-0.596707	-0.785359	-0.389638	SRSF6	serine/argi	FALSE	Homo sapi	6431	GO:000838	42087944	chr20	42087885	+	hs|20q13.1	NR_034009	chr20:4208	NR_034009	Hs.725336
A_23_P100	-2.009754	-1.007019	2.009754	down	0.458143	0.516019	0.491936	-0.502787	-0.458143	-0.59403	ORC6	origin reco	FALSE	Homo sapi	23594	GO:000626	46731578	chr16	46731519	+	hs|16q11.2	NM_01432	chr16:4673	NM_01432	Hs.49760
A_21_P001	-3.199535	-1.677862	3.199535	down	0.709021	0.742281	0.776919	-1.242244	-0.854101	-0.709021	XLOC_l2_010724		FALSE	BROAD Institute lincRNA (XLOC_l2			80131901	chr4	80131842	+	hs|4q21.21		chr4:80131842-80131901		
A_24_P941	-2.163697	-1.113498	2.163697	down	0.548013	0.458616	0.449592	-0.449592	-0.916269	-0.518412	APOL6	apolipopro	FALSE	Homo sapi	80830	GO:000573	36057177	chr22	36057118	+	hs|22q12.3	NM_03064	chr22:3605	NM_03064	Hs.257352
A_33_P322	-6.708342	-2.745956	6.708342	down	1.273021	1.297688	1.269583	-1.552083	-1.269584	-1.57591	BST2	bone marro	FALSE	Homo sapi	684	GO:000551	17513807	chr19	17513748	-	hs|19p13.1	NM_00433	chr19:1751	NM_00433	Hs.118110
A_24_P380	-2.36173	-1.239844	2.36173	down	0.648818	0.619714	0.505021	-0.668483	-0.505021	-0.772475	FKBP5	FK506 bind	FALSE	Homo sapi	2289	GO:000551	35541987	chr6	35541928	-	hs|6p21.31	NM_00411	chr6:35541	NM_00411	Hs.407190
A_23_P104	-2.443842	-1.289151	2.443842	down	0.622207	0.608725	0.613673	-0.642456	-0.771668	-0.608725	DDIT4	DNA-dama	FALSE	Homo sapi	54541	GO:000166	74035422	chr10	74035363	+	hs|10q22.1	NM_01905	chr10:7403	NM_01905	Hs.744875
A_33_P337	-2.151674	-1.10546	2.151674	down	0.468816	0.510506	0.451801	-0.770433	-0.451801	-0.663022	CLIC4	chloride int	FALSE	Homo sapi	25932	GO:000551	25167365	chr1	25167306	+	hs|1p36.11	NM_01394	chr1:25167	NM_01394	Hs.440544

A_24_P307	-3.142255	-1.6518	3.142255	down	0.851655	0.744532	0.77139	-0.744532	-0.963521	-0.879771			FALSE	activating transcription factor 4 ps			74222655	chr17	74222596	-	hs|17q25.1		chr17:74222655-74222596		
A_23_P353	-2.018548	-1.013318	2.018548	down	0.376014	0.413891	0.369156	-0.793583	-0.369156	-0.718154	C2CD2L	C2CD2-like	FALSE	Homo sapi	9854	GO:000815	1.19E+08	chr11	1.19E+08	+	hs|11q23.3	NM_01480	chr11:1189	NM_01480	Hs.26899
A_33_P337	-2.188481	-1.12993	2.188481	down	0.513385	0.504873	0.538498	-0.504873	-0.781026	-0.547134	HLA-F	major histc	FALSE	Homo sapi	3134	GO:000247	29692173	chr6	29692114	+	hs|6p22.1	NM_00109	chr6:29692	NM_00109	Hs.519972
A_23_P422	-2.300836	-1.202158	2.300836	down	0.33034	0.383733	0.581736	-1.00634	-0.33034	-0.973985	B3GALT4	UDP-Gal:be	FALSE	Homo sapi	8705	GO:000013	33246603	chr6	33246543	+	hs|6p21.32	NM_00378	chr6:33246	NM_00378	Hs.534375
A_24_P349	-2.166753	-1.115534	2.166753	down	0.461831	0.453536	0.518528	-0.529876	-0.929297	-0.453536	LOC441081	POM121 m	FALSE	Homo sapi	441081	GO:001602	68931876	chr5	68931817	+	hs|5q13.2	NR_07340	chr5:68931	NR_07340	Hs.721548
A_23_P214	-2.45212	-1.29403	2.45212	down	0.512198	0.535043	0.540856	-0.924793	-0.512198	-0.857001	EDN1	endothelin	FALSE	Homo sapi	1906	GO:000551	12296731	chr6	12296672	+	hs|6p24.1	NM_00195	chr6:12296	NM_00195	Hs.511899
A_24_P101	-2.557035	-1.354472	2.557035	down	0.686181	0.761086	0.609692	-0.633333	-0.609692	-0.763431	RGCC	regulator o	FALSE	Homo sapi	28984	GO:000551	42044635	chr13	42042937	+	hs|13q14.1	NM_01405	chr13:4204	NM_01405	Hs.507866
A_21_P001	-2.005661	-1.004078	2.005661	down	0.502387	0.431444	0.39786	-0.69064	-0.39786	-0.592043	DUXAP10	double hor	FALSE	double hor	503639		19662660	chr14	19662601	+	hs|14q11.2	BC013389	chr14:19662601-19662		Hs.744284
A_23_P743	-2.388073	-1.255847	2.388073	down	0.541084	0.756118	0.593973	-0.633754	-0.701528	-0.541084	OPN3	opsin 3	FALSE	Homo sapi	23596	GO:001829	2.42E+08	chr1	2.42E+08	-	hs|1q43	NM_01432	chr1:24175	NM_01432	Hs.409081
A_22_P000	-2.396404	-1.260871	2.396404	down	0.341486	0.492113	0.388736	-1.136029	-0.341486	-1.082765	LOC10192	uncharacte	FALSE	PREDICTED	1.02E+08		41840799	chr1	41840740	-	hs|1p34.2	XR_425140	chr1:41840	XR_425140	
A_23_P169	-2.134404	-1.093833	2.134404	down	0.635483	0.272312	0.660115	-0.272312	-1.039005	-0.402274	WISP1	WNT1 indu	FALSE	Homo sapi	8840	GO:000716	1.34E+08	chr8	1.34E+08	+	hs|8q24.22	NM_08083	chr8:13423	NM_08083	Hs.492974
A_23_P356	-2.226059	-1.154492	2.226059	down	0.538361	0.57957	0.499126	-0.622562	-0.499126	-0.724731	PLCE1	phospholip	FALSE	Homo sapi	51196	GO:004585	96087992	chr10	96087933	+	hs|10q23.3	NM_01634	chr10:9608	NM_01634	Hs.655033
A_23_P132	-2.282549	-1.190646	2.282549	down	0.484726	0.567956	0.67737	-0.587958	-0.769201	-0.484726	SPAG4	sperm assc	FALSE	Homo sapi	6676	GO:000573	34208649	chr20	34207653	+	hs|20q11.2	NM_00311	chr20:3420	NM_00311	Hs.123159
A_23_P370	-2.963779	-1.567438	2.963779	down	0.791068	0.69212	0.645627	-0.964631	-0.963243	-0.645627	BATF2	basic leucir	FALSE	Homo sapi	116071	GO:004356	64755631	chr11	64755572	-	hs|11q13.1	NM_13845	chr11:6475	NM_13845	Hs.124840
A_21_P000	-2.3401	-1.22657	2.3401	down	0.629206	0.539367	0.473197	-1.037767	-0.473197	-0.526976	SNORD114	small nucle	FALSE	Homo sapi	767592		1.01E+08	chr14	1.01E+08	+	hs|14q32.3	NR_00320	chr14:1014	NR_003207	
A_23_P768	-2.145443	-1.101275	2.145443	down	0.268482	0.621043	0.441329	-1.130458	-0.574032	-0.268482	ADSSL1	adenylosuc	FALSE	Homo sapi	122622	GO:004604	1.05E+08	chr14	1.05E+08	+	hs|14q32.3	NM_19916	chr14:1052	NM_19916	Hs.592327
A_23_P169	-3.415547	-1.772117	3.415547	down	0.772057	0.809078	0.814198	-1.086146	-0.772057	-1.062814	MMP1	matrix met	FALSE	Homo sapi	4312	GO:000550	1.03E+08	chr11	1.03E+08	-	hs|11q22.2	NM_00242	chr11:1026	NM_00242	Hs.83169
A_23_P154	-2.139341	-1.097167	2.139341	down	0.527087	0.493206	0.530889	-0.644205	-0.493206	-0.602906	TOX2	TOX high m	FALSE	Homo sapi	84969	GO:000635	42698155	chr20	42698096	+	hs|20q13.1	NM_03288	chr20:4269	NM_03288	Hs.26608
A_33_P329	-2.02483	-1.017801	2.02483	down	0.395705	0.349562	0.325357	-0.891253	-0.325357	-0.766168	PRKCI	protein kin	FALSE	Homo sapi	5584	GO:000551	1.7E+08	chr3	1.7E+08	+	hs|3q26.2	NM_00274	chr3:17002	NM_00274	Hs.478199
A_24_P133	-2.26007	-1.176368	2.26007	down	0.658928	0.729767	0.460004	-0.534825	-0.460004	-0.685575	KITLG	KIT ligand	FALSE	Homo sapi	4254	GO:000551	88886712	chr12	88886653	-	hs|12q21.3	NM_00089	chr12:8888	NM_00089	Hs.1048
A_22_P000	-6.874332	-2.78122	6.874332	down	1.18008	1.09942	1.233521	-1.910022	-1.09942	-1.821195	lnc-MFSD9	lnc-MFSD9	FALSE	1ITB_B Chain B, Type-1 Interleukin			1.03E+08	chr2	1.03E+08	-	hs|2q12.1		chr2:102789140-102789081		
A_33_P332	-2.337968	-1.225255	2.337968	down	0.647286	0.628145	0.52263	-0.702124	-0.52263	-0.65295	TSPY10	testis speci	FALSE	Homo sapi	1E+08	GO:000750	9366703	chrY	9366644	+	hs|Yp11.2	NM_00128	chrY:93666	NM_00128	Hs.646249
A_23_P185	-2.177869	-1.122917	2.177869	down	0.565612	0.508852	0.541338	-0.543783	-0.700315	-0.508852	PI4K2B	phosphatid	FALSE	Homo sapi	55300	GO:000443	25280046	chr4	25279987	+	hs|4p15.2	NM_01832	chr4:25279	NM_01832	Hs.191701
A_23_P109	-2.423003	-1.276796	2.423003	down	0.491578	0.667614	0.522633	-0.491578	-1.097997	-0.558988	SALL4	spalt-like tr	FALSE	Homo sapi	57167	GO:000079	50400915	chr20	50400856	-	hs|20q13.2	NM_02043	chr20:5040	NM_02043	Hs.517113
A_24_P384	-2.34842	-1.23169	2.34842	down	0.554488	0.577798	0.521803	-0.622407	-0.896773	-0.521803	EWSR1	EWS RNA-k	FALSE	Homo sapi	2130	GO:000551	29686480	chr22	29686421	+	hs|22q12.2	NM_00116	chr22:2968	NM_00116	Hs.374477
A_33_P320	-4.935321	-2.303144	4.935321	down	1.043076	1.051696	1.117848	-1.505285	-1.043076	-1.148451	RASGRP2	RAS guanyl	FALSE	Homo sapi	10235	GO:004300	64508476	chr11	64508420	-	hs|11q13.1	NM_15381	chr11:6450	NM_15381	Hs.99491
A_23_P254	-2.88456	-1.528351	2.88456	down	0.594695	0.73796	0.915988	-0.731212	-0.594695	-1.010504	RPA4	replication	FALSE	Homo sapi	29935	GO:000008	96140331	chrX	96140272	+	hs|Xq21.33	NM_01334	chrX:9614	NM_01334	Hs.659349
A_23_P204	-4.837425	-2.274239	4.837425	down	1.057811	0.939753	0.963644	-1.473404	-0.939753	-1.448355	MGP	matrix Gla	FALSE	Homo sapi	4256	GO:003101	15034999	chr12	15034940	-	hs|12p12.3	NM_00090	chr12:1503	NM_00090	Hs.365706
A_23_P255	-5.426204	-2.439943	5.426204	down	1.127454	1.097632	0.995264	-1.639119	-0.995264	-1.465098	ABLIM2	actin bindi	FALSE	Homo sapi	84448	GO:003003	7967135	chr4	7967076	-	hs|4p16.1	NM_03243	chr4:79671	NM_03243	Hs.233404
A_33_P325	-2.080945	-1.057239	2.080945	down	0.452066	0.54789	0.477635	-0.658358	-0.452066	-0.583703	WDR62	WD repeat	FALSE	Homo sapi	284403	GO:000573	36596011	chr19	36595952	+	hs|19q13.1	NM_00108	chr19:3659	NM_00108	Hs.116244
A_33_P330	-2.574348	-1.364207	2.574348	down	0.608496	0.608222	0.605067	-0.747874	-0.605067	-0.917894	TLE4	transducin-	FALSE	transducin-	7091	GO:000551	82242662	chr9	82242603	+	hs|9q21.31		chr9:82242603-82242662		
A_32_P162	-4.190958	-2.06728	4.190958	down	0.907893	0.986776	0.894556	-0.894556	-1.378983	-1.139076	C18orf54	chromoson	FALSE	Homo sapi	162681	GO:000557	51907544	chr18	51907485	+	hs|18q21.2	NM_17352	chr18:5190	NM_17352	Hs.208701
A_22_P000	-2.064933	-1.046095	2.064933	down	0.53618	0.468626	0.517458	-0.468626	-0.530432	-0.616962	lnc-BTBD1	lnc-BTBD1	FALSE	LNCipedia lincRNA (lnc-BTBD19-1),			45282689	chr1	45282630	+	hs|1p34.1		chr1:45282630-45282689		
A_33_P339	-2.374263	-1.24748	2.374263	down	0.458049	0.585483	0.554698	-1.078912	-0.458049	-0.607249	GRIN3B	glutamate	FALSE	Homo sapi	116444	GO:000526	1009707	chr19	1009648	+	hs|19p13.3	NM_13869	chr19:1009	NM_13869	Hs.660378
A_33_P325	-2.450431	-1.293036	2.450431	down	0.567383	0.57155	0.580359	-0.790141	-0.567383	-0.80229	MEG3	maternally	FALSE	Homo sapi	55384		1.01E+08	chr14	1.01E+08	+	hs|14q32.2	NR_00276	chr14:1013	NR_00276	Hs.654863
A_23_P111	-2.430236	-1.281097	2.430236	down	0.547822	0.61372	0.626656	-0.678255	-0.829016	-0.547822	PARP12	poly (ADP-r	FALSE	Homo sapi	64761	GO:000815	1.4E+08	chr7	1.4E+08	-	hs|7q34	NM_02275	chr7:13972	NM_02275	Hs.12646
A_24_P130	-2.201532	-1.138508	2.201532	down	0.569349	0.513584	0.380838	-0.862234	-0.380838	-0.708683	DDX3Y	DEAD (Asp-	FALSE	Homo sapi	8653	GO:000573	15027921	chrY	15027862	+	hs|Yq11.21	NM_00466	chrY:15027	NM_00466	Hs.99120
A_23_P681	-3.293092	-1.719443	3.293092	down	0.854961	0.796269	0.801168	-0.969937	-0.796269	-0.939724	IFIH1	interferon i	FALSE	Homo sapi	64135	GO:000372	1.63E+08	chr2	1.63E+08	-	hs|2q24.2	NM_02216	chr2:16312	NM_02216	Hs.163173
A_23_P640	-9.370277	-3.228092	9.370277	down	1.607084	1.57374	1.543368	-1.685091	-1.543367	-1.731626	RASGRP2	RAS guanyl	FALSE	Homo sapi	10235	GO:004300	64494473	chr11	64494414	-	hs|11q13.1	NM_15381	chr11:6449	NM_15381	Hs.99491
A_23_P326	-2.270998	-1.183326	2.270998	down	0.475829	0.547506	0.551322	-0.747386	-0.475828	-0.752107	LIMS2	LIM and se	FALSE	Homo sapi	55679	GO:200104	1.28E+08	chr2	1.28E+08	-	hs|2q14.3	NM_00116	chr2:12839	NM_00116	Hs.469881
A_21_P001	-2.08477	-1.059888	2.08477	down	0.467843	0.494783	0.556045	-0.553011	-0.467843	-0.640141	XLOC_l2_013594		FALSE	BROAD Institute lincRNA (XLOC_l2			1.28E+08	chr7	1.28E+08	+	hs|7q32.1		chr7:128173739-128231917		
A_21_P000	-2.062712	-1.044542	2.062712	down	0.377399	0.372227	0.316378	-1.142609	-0.608636	-0.316378	SNORA4	small nucle	FALSE	Homo sapi	619568		1.87E+08	chr3	1.87E+08	+	hs|3q27.3	NR_00258	chr3:1865	NR_00258	Hs.743897
A_21_P001	-2.104573	-1.073528	2.104573	down	0.402591	0.444772	0.621071	-0.863132	-0.402591	-0.486425	XLOC_l2_006399		FALSE	BROAD Institute lincRNA (XLOC_l2			10979310	chr18	10979251	-	hs|18p11.21		chr18:10979310-10979251		
A_33_P341	-3.910697	-1.967426	3.910697	down	0.893211	0.883994	0.961162	-1.232677	-1.04724	-0.883994	ANKRD36B	ankyrin rep	FALSE	Homo sapi	57730		98121431	chr2	98121372	-	hs|2q11.2	NM_02519	chr2:98121	NM_02519	Hs.532921
A_33_P331	-2.134487	-1.093889	2.134487	down	0.566595	0.44225	0.605946	-0.500989	-0.44225	-0.72364	EXD3	exonucleas	FALSE	Homo sapi	54932	GO:000551	1.4E+08	chr9	1.4E+08	-	hs|9q34.3	NM_00128	chr9:14026	NM_00128	Hs.495553
A_22_P000	-2.147948	-1.102959	2.147948	down	0.712925	0.225042	0.805925	-0.653236	-0.225042	-0.686708	lnc-SRBD1-	lnc-SRBD1-	FALSE	LNCipedia lincRNA (lnc-SRBD1-1), l			45875941	chr2	45875882	-	hs|2p21		chr2:45875941-45875882		
A_33_P337	-2.022484	-1.016128	2.022484	down	0.380807	0.262342	0.356224	-0.820756	-0.262342	-0.965914	MKI67	marker of p	FALSE	Homo sapi	4288	GO:000551	1.3E+08	chr10	1.3E+08	-	hs|10q26.2	NM_00241	chr10:1299	NM_00241	Hs.80976
A_23_P749	-2.193853	-1.133467	2.193853	down	0.444018	0.490561	0.494746	-0.769816	-0.444018	-0.757242	RCC2	regulator o	FALSE	Homo sapi	55920	GO:000551	17733553	chr1	17733494	-	hs|1p36.13	NM_01871	chr1:17733	NM_01871	Hs.380857
A_33_P321	-2.358259	-1.237722	2.358259	down	0.620041	0.619011	0.496844	-0.496844	-0.788482	-0.691943	RBFOX2	RNA bindin	FALSE	Homo sapi	23543	GO:000551	36140089	chr22	36140030	-	hs|22q12.3	NM_00103	chr22:3614	NM_00103	Hs.282998
A_23_P219	-4.408118	-2.140163	4.408118	down	1.053402	1.038105	1.026826	-1.147579	-1.127749	-1.026826	CSPG4	chondroitir	FALSE	Homo sapi	1464	GO:004877	75967271	chr15	75967212	-	hs|15q24.2	NM_00189	chr15:7596	NM_00189	Hs.513044
A_22_P000	-2.757862	-1.46355	2.757862	down	0.71419	0.658305	0.688509	-0.813497	-0.658305	-0.857844	LOC10192	uncharacte	FALSE	PREDICTED	1.02E+08		39480728	chr15	39480669	-	hs|15q14	XR_243144	chr15:3948	XR_243144	Hs.434739
A_33_P332	-2.7507	-1.459799	2.7507	down	0.628335	0.631914	0.741681	-0.728583	-1.020548	-0.628335	AGPAT4	1-acylglyce	FALSE	Homo sapi	56895	GO:001943	1.62E+08	chr6	1.62E+08	-	hs|6q26	NM_02013	chr6:16157	NM_02013	Hs.353175
A_23_P434	-2.008384	-1.006035	2.008384	down	0.533067	0.435302	0.555102	-0.435302	-0.571428	-0.487904	STT3B	STT3B, sub	FALSE	Homo sapi	201595	GO:000698	31674437	chr3	31670855	+	hs|3p23	NM_17886	chr3:3167	NM_17886	Hs.475812
A_23_P352	-2.023822	-1.017083	2.023822	down	0.461602	0.471297	0.419263	-0.419263	-0.677189	-0.602634	CD46	CD46 mole	FALSE	Homo sapi	4179	GO:000551	2.08E+08	chr1	2.08E+08	+	hs|1q32.2	NM_17235	chr1:20794	NM_17235	Hs.510402
A_21_P001	-2.435045	-1.283948	2.435045	down	0.564669	0.634898	0.622366	-0.564669	-0.748488	-0.716755			FALSE	testis specific protein, Y-linked 15,			9386866	chrY	9386807	+	hs|Yp11.2		chrY:9386807-9386866		
A_24_P208	-2.827893	-1.499728	2.827893	down	0.749744	0.565411	0.820062	-0.718963	-0.565411	-1.079592	SLC45A3	solute carri	FALSE	Homo sapi	85414	GO:001602	2.06E+08	chr1	2.06E+08	-	hs|1q32.1	NM_03310	chr1:20562	NM_03310	Hs.278695
A_23_P503	-2.578133	-1.366327	2.578133	down	0.499679	0.575702	0.545223	-0.906319	-0.499679	-1.072378	PHF10	PHD finger	FALSE	Homo sapi	55274	GO:000635	1.7E+08	chr6	1.7E+08	-	hs|6q27	NM_01828	chr6:1701	NM_01828	Hs.435933
A_33_P331	-2.343112	-1.228426	2.343112	down	0.557416	0.613762	0.640232	-0.69197	-0.557416	-0.624481	NOTCH3	notch 3	FALSE	Homo sapi	4854	GO:000551	15270548	chr19	15270489	-	hs|19p13.1	NM_00043	chr19:1527	NM_00043	Hs.8546

A_33_P334	-2.099782	-1.07024	2.099782	down	0.515121	0.512612	0.499429	-0.499429	-0.647246	-0.536884	RC3H2	ring finger	FALSE	Homo sapi	54542	GO:000020	1.26E+08	chr9	1.26E+08	-	hs|9q33.2	NM_01883	chr9:12563	NM_01883	Hs.533499
A_21_P001	-2.058329	-1.041474	2.058329	down	0.679851	0.592006	0.268683	-0.268683	-0.697841	-0.617358			FALSE	PREDICTED: Homo sapiens unchar			1.3E+08	chr3	1.3E+08	+	hs|3q22.1	XR_241558	chr3:12983	XR_241558	Hs.744344
A_32_P366	-2.216226	-1.148105	2.216226	down	0.564103	0.537514	0.507959	-0.657763	-0.507959	-0.669019	JAZF1	JAZF zinc fi	FALSE	Homo sapi	221895	GO:001705	27870436	chr7	27870377	-	hs|7p15.2	NM_17506	chr7:27870	NM_17506	Hs.368944
A_33_P341	-2.879933	-1.526035	2.879933	down	0.66726	0.767293	0.601352	-0.971512	-0.601352	-0.969336	DDX58	DEAD (Asp	FALSE	Homo sapi	23586	GO:000551	32455787	chr9	32455728	-	hs|9p21.1	NM_01431	chr9:32455	NM_01431	Hs.190622
A_22_P000	-2.419574	-1.274753	2.419574	down	0.717843	0.76873	0.533909	-0.593406	-0.676462	-0.533909	EEF1DP3	eukaryotic	FALSE	Homo sapi	196549		32520317	chr13	32520258	+	hs|13q13.1		chr13:3252	NR_027062	Hs.507667
A_24_P178	-2.128401	-1.08977	2.128401	down	0.443757	0.604073	0.533313	-0.47062	-0.77379	-0.443757	HCP5	HLA compl	FALSE	Homo sapi	10866	GO:000695	31432704	chr6	31432645	+	hs|6p21.33	NR_040662	chr6:31432	NR_040662	Hs.691948
A_24_P921	-2.204656	-1.140554	2.204656	down	0.540632	0.520688	0.523632	-0.520688	-0.537582	-0.778438	SRSF1	serine/argi	FALSE	Homo sapi	6426	GO:000551	56078449	chr17	56078390	-	hs|17q22	NM_00107	chr17:5607	NM_00107	Hs.68714
A_33_P329	-2.191397	-1.131851	2.191397	down	0.568279	0.475611	0.661478	-0.53748	-0.475611	-0.677094	HAS1	hyaluronar	FALSE	Homo sapi	3036	GO:000588	52220281	chr19	52220222	-	hs|19q13.4	NM_00152	chr19:5222	NM_00152	Hs.57697
A_33_P367	-2.157513	-1.109369	2.157513	down	0.549194	0.438922	0.431143	-0.771274	-0.70643	-0.431143			FALSE				35234990	chr15	35234931	-	hs|15q14		chr15:035234990-035234931		
A_33_P336	-2.635491	-1.398072	2.635491	down	0.651842	0.685253	0.632941	-0.632941	-0.813534	-0.777705	CFH	complemer	FALSE	Homo sapi	3075	GO:000695	1.97E+08	chr1	1.97E+08	+	hs|1q31.3	NM_00101	chr1:19664	NM_00101	Hs.363396
A_33_P335	-3.016163	-1.592715	3.016163	down	0.543788	0.673798	0.531667	-1.134168	-0.531667	-1.363056	DHRS4L1	dehydroge	FALSE	Homo sapi	728635	GO:001649	24520507	chr14	24520448	+	hs|14q11.2	NM_00127	chr14:2452	NM_00127	Hs.647569
A_23_P306	-2.754176	-1.461621	2.754176	down	0.675267	0.652633	0.693911	-0.812304	-0.652633	-0.898114	TNFRSF21	tumor necr	FALSE	Homo sapi	27242	GO:000551	47199542	chr6	47199483	-	hs|6p12.3	NM_01445	chr6:47199	NM_01445	Hs.443577
A_23_P701	-2.157456	-1.109331	2.157456	down	0.524427	0.497	0.585119	-0.584855	-0.639592	-0.497	TMED9	transmemt	FALSE	Homo sapi	54732	GO:000551	1.77E+08	chr5	1.77E+08	+	hs|5q35.3	NM_01751	chr5:17702	NM_01751	Hs.279929
A_23_P726	-2.205417	-1.141052	2.205417	down	0.258077	0.541809	0.970698	-0.696414	-0.258077	-0.698081	SDPR	serum depi	FALSE	Homo sapi	8436	GO:000551	1.93E+08	chr2	1.93E+08	-	hs|2q32.3	NM_00465	chr2:19270	NM_00465	Hs.26530
A_23_P609	-3.362149	-1.749384	3.362149	down	0.814923	0.877538	0.853556	-0.964734	-0.814923	-0.922477	PLCB1	phospholip	FALSE	Homo sapi	23236	GO:000551	8755271	chr20	8755212	+	hs|20p12.3	NM_01519	chr20:8755	NM_01519	Hs.431173
A_23_P386	-2.55762	-1.354802	2.55762	down	0.583808	0.605532	0.560769	-0.850719	-0.560769	-0.90281	FAM110A	family with	FALSE	Homo sapi	83541	GO:000551	826898	chr20	826839	+	hs|20p13	NM_00104	chr20:8268	NM_00104	Hs.574822
A_23_P360	-2.316192	-1.211755	2.316192	down	0.43675	0.444853	0.433015	-0.905078	-0.433015	-0.982553	ADAMTS4	ADAM met	FALSE	Homo sapi	9507	GO:000551	1.61E+08	chr1	1.61E+08	-	hs|1q23.3	NM_00509	chr1:16115	NM_00509	Hs.211604
A_33_P340	-2.000189	-1.000137	2.000189	down	0.35208	0.536534	0.351276	-0.746299	-0.351276	-0.662946	FOXO6	forkhead b	FALSE	Homo sapi	1E+08	GO:004356	41849257	chr1	41849198	+	hs|1p34.2	NM_00129	chr1:41849	NM_001291281	
A_23_P194	-2.438126	-1.285773	2.438126	down	0.564493	0.579453	0.48693	-0.770324	-0.48693	-0.969189	DDAH2	dimethylar	FALSE	Homo sapi	23564	GO:000551	31694977	chr6	31694918	-	hs|6p21.33	NM_01397	chr6:31694	NM_01397	Hs.247362
A_33_P331	-3.070004	-1.61824	3.070004	down	0.809294	0.649158	0.72389	-0.912221	-0.649158	-1.111			FALSE				1.32E+08	chr2	1.32E+08	-	hs|2q21.1		chr2:132056314-132056255		
A_23_P156	-2.231496	-1.158011	2.231496	down	0.455713	0.478451	0.599391	-0.840695	-0.455713	-0.644069	ENPP1	ectonucleo	FALSE	Homo sapi	5167	GO:000551	1.32E+08	chr6	1.32E+08	+	hs|6q23.2	NM_00620	chr6:13221	NM_00620	Hs.527295
A_24_P150	-2.174573	-1.120732	2.174573	down	0.407544	0.42909	0.453391	-1.020063	-0.644565	-0.407544	NXPH3	neurexoph	FALSE	Homo sapi	11248	GO:000367	47657055	chr17	47656996	+	hs|17q21.3	NM_00722	chr17:4765	NM_00722	Hs.55069
A_24_P267	-2.472328	-1.30587	2.472328	down	0.55115	0.537517	0.551828	-0.585713	-1.153886	-0.537517			FALSE				19005666	chr22	19005607	-	hs|22q11.21		chr22:19005666-19005607		
A_23_P331	-2.4263	-1.278758	2.4263	down	0.530789	0.597021	0.560119	-0.82003	-0.530788	-0.797525	COL5A2	collagen, ty	FALSE	Homo sapi	1290	GO:004859	1.9E+08	chr2	1.9E+08	-	hs|2q32.2	NM_00039	chr2:18989	NM_00039	Hs.445827
A_33_P330	-3.412959	-1.771023	3.412959	down	0.841477	0.816186	0.814584	-0.884482	-1.141755	-0.814584	POM121L1	POM121 tr	FALSE	Homo sapi	25812		22974270	chr22	22974211	-	hs|22q11.2	NR_024591	chr22:2297	NR_024591	Hs.367764
A_23_P137	-2.560371	-1.356353	2.560371	down	0.578322	0.580887	0.543783	-0.873851	-0.543783	-0.948434	S100A10	S100 calciu	FALSE	Homo sapi	6281	GO:000551	1.52E+08	chr1	1.52E+08	-	hs|1q21.3	NM_00296	chr1:15195	NM_00296	Hs.143873
A_33_P331	-2.258464	-1.175342	2.258464	down	0.492432	0.703377	0.757107	-0.581341	-0.499338	-0.492432	MICAL2	microtubul	FALSE	Homo sapi	9645	GO:001941	12281617	chr11	12281558	+	hs|11p15.3	NM_00128	chr11:1228	NM_00128	Hs.501928
A_24_P215	-2.388272	-1.255967	2.388272	down	0.452952	0.480861	0.451581	-0.982094	-0.45158	-0.948833	ATP10A	ATPase, cla	FALSE	Homo sapi	57194	GO:000551	25923934	chr15	25923875	-	hs|15q12	NM_02449	chr15:2592	NM_02449	Hs.659258
A_21_P001	-2.00167	-1.001204	2.00167	down	0.331018	0.500112	0.585394	-0.611647	-0.331018	-0.644425			FALSE	Homo sapiens cDNA clone IMAGE:			97637553	chr9	97637494	+	hs|9q22.32	BC042892	chr9:97637494-976375		Hs.655466
A_33_P340	-2.133048	-1.092917	2.133048	down	0.394984	0.366434	0.332771	-0.681863	-1.169927	-0.332771	TLE6	transducin-	FALSE	Homo sapi	79816	GO:004323	2994089	chr19	2994030	+	hs|19p13.3	NM_00114	chr19:2994	NM_00114	Hs.334507
A_24_P374	-2.784357	-1.477344	2.784357	down	0.770845	0.704042	0.718015	-0.77624	-0.704042	-0.758847	PDK1	pyruvate d	FALSE	Homo sapi	5163	GO:000474	1.73E+08	chr2	1.73E+08	+	hs|2q31.1	NM_00261	chr2:17346	NM_00261	Hs.470633
A_22_P000	-2.017673	-1.012692	2.017673	down	0.317005	0.67188	0.414418	-0.987702	-0.317005	-0.330067	lnc-LENG9-	lnc-LENG9-	FALSE	LNCipedia lincRNA (lnc-LENG9-1),			54959409	chr19	54959350	-	hs|19q13.42		chr19:54959409-54959350		
A_23_P390	-2.379483	-1.250648	2.379483	down	0.616337	0.586649	0.553903	-0.77138	-0.553903	-0.669773	TTC39B	tetratricop	FALSE	Homo sapi	158219		15172034	chr9	15171975	-	hs|9p22.3	NM_15257	chr9:15172	NM_15257	Hs.563630
A_22_P000	-2.027671	-1.019824	2.027671	down	0.531777	0.542604	0.414925	-0.711848	-0.414925	-0.443391	LINC00087	long interg	FALSE	Homo sapi	644596		1.34E+08	chrX	1.34E+08	-	hs|Xq26.3	NR_024493	chrX:13422	NR_024493	Hs.656427
A_23_P870	-2.104009	-1.073141	2.104009	down	0.486651	0.448174	0.455291	-0.448916	-0.932218	-0.448174	TAGLN	transgelin	FALSE	Homo sapi	6876	GO:000551	1.17E+08	chr11	1.17E+08	+	hs|11q23.3	NM_00100	chr11:1170	NM_00100	Hs.410977
A_33_P324	-2.910605	-1.541319	2.910605	down	0.905599	0.62525	0.807007	-0.779049	-0.881803	-0.62525			FALSE				9477884	chrY	9477825	-	hs|Yp11.2		chrY:009477884-009477825		
A_21_P001	-2.610798	-1.384491	2.610798	down	0.793125	0.638556	0.605288	-0.65726	-0.605288	-0.853956			FALSE												
A_23_P158	-2.162629	-1.112786	2.162629	down	0.477669	0.473906	0.470974	-0.725845	-0.470975	-0.71899	SLC16A3	solute carri	FALSE	Homo sapi	9123	GO:003196	80197347	chr17	80197288	+	hs|17q25.3	NM_00104	chr17:8019	NM_00104	Hs.500761
A_33_P322	-2.03986	-1.02847	2.03986	down	0.55429	0.414854	0.347137	-0.609601	-0.347137	-0.812392	SLC17A9	solute carri	FALSE	Homo sapi	63910	GO:000521	61599941	chr20	61599882	+	hs|20q13.3	NM_00130	chr20:6159	NM_00130	Hs.512686
A_33_P339	-2.046783	-1.033358	2.046783	down	0.288935	0.577918	0.488717	-0.59642	-0.288935	-0.85915	BLZF1	basic leucir	FALSE	Homo sapi	8548	GO:000370	1.69E+08	chr1	1.69E+08	+	hs|1q24.2	NM_00366	chr1:16936	NM_00366	Hs.130746
A_22_P000	-3.086945	-1.62618	3.086945	down	0.744171	0.761889	0.745238	-0.948171	-0.74417	-0.9349	AGRN	agrin	FALSE	Homo sapi	375790	GO:000551	991491	chr1	991432	+	hs|1p36.33	NM_19857	chr1:99143	NM_19857	Hs.273330
A_23_P359	-2.23753	-1.161907	2.23753	down	0.474823	0.484962	0.413678	-0.790418	-0.413678	-0.908161	MET	MET proto-	FALSE	Homo sapi	4233	GO:000551	1.16E+08	chr7	1.16E+08	+	hs|7q31.2	NM_00024	chr7:11643	NM_00024	Hs.132966
A_24_P261	-2.0394	-1.028145	2.0394	down	0.630256	0.412092	0.583094	-0.540499	-0.412092	-0.506403	DKK3	dickkopf W	FALSE	Homo sapi	27122	GO:003032	11984974	chr11	11984915	-	hs|11p15.3	NM_01588	chr11:1198	NM_01588	Hs.292156
A_24_P237	-2.234067	-1.159672	2.234067	down	0.437194	0.504835	0.696555	-0.567112	-0.437194	-0.836126	ANKRD37	ankyrin rep	FALSE	Homo sapi	353322	GO:000573	1.86E+08	chr4	1.86E+08	+	hs|4q35.1	NM_18172	chr4:18632	NM_18172	Hs.508154
A_21_P001	-2.012113	-1.008711	2.012113	down	0.490578	0.429765	0.57218	-0.429765	-0.45705	-0.646795	lnc-PPIAL4	lnc-PPIAL4	FALSE	Q3SWV3_HUMAN (Q3SWV3) LOC			1.43E+08	chr1	1.43E+08	-	hs|1q21.1		chr1:143464707-143464648		
A_23_P252	-3.399087	-1.765147	3.399087	down	0.880191	0.782061	0.890926	-0.95473	-1.005472	-0.782061	FILIP1L	filamin A ir	FALSE	Homo sapi	11259	GO:000815	99567410	chr3	99567351	-	hs|3q12.1	NM_18290	chr3:99567	NM_18290	Hs.104672
A_33_P332	-2.131076	-1.091582	2.131076	down	0.531551	0.426663	0.490444	-0.651631	-0.426663	-0.747793	IGSF9B	immunoglc	FALSE	Homo sapi	22997	GO:009715	1.34E+08	chr11	1.34E+08	-	hs|11q25	AB028953	chr11:133785472-1337		Hs.204121
A_23_P150	-2.646109	-1.403872	2.646109	down	0.707483	0.978464	0.550351	-0.775152	-0.649817	-0.550351	MMP12	matrix met	FALSE	Homo sapi	4321	GO:006005	1.03E+08	chr11	1.03E+08	-	hs|11q22.2	NM_00242	chr11:1027	NM_00242	Hs.1695
A_23_P933	-2.0628	-1.044604	2.0628	down	0.468071	0.433479	0.461509	-0.433479	-0.860227	-0.477047	RGL2	ral guanine	FALSE	Homo sapi	5863	GO:000562	33260865	chr6	33260806	-	hs|6p21.32	NM_00476	chr6:33260	NM_00476	Hs.509622
A_33_P384	-3.388678	-1.760723	3.388678	down	0.85573	0.862611	0.835255	-0.835255	-0.855819	-1.037498	IKZF2	IKAROS fan	FALSE	Homo sapi	22807	GO:000551	2.14E+08	chr2	2.14E+08	-	hs|2q34	NM_00107	chr2:21387	NM_00107	Hs.604950
A_23_P368	-2.546803	-1.348687	2.546803	down	0.56148	0.693754	0.561722	-0.959964	-0.56148	-0.707663	CHSY3	chondroitir	FALSE	Homo sapi	337876	GO:003020	1.3E+08	chr5	1.3E+08	+	hs|5q23.3	NM_17585	chr5:12952	NM_17585	Hs.213137
A_23_P206	-2.033023	-1.023626	2.033023	down	0.442204	0.429724	0.438387	-0.682505	-0.429724	-0.648335	ITGA11	integrin, al	FALSE	Homo sapi	22801	GO:000830	68594130	chr15	68594071	-	hs|15q23	NM_00100	chr15:6859	NM_00100	Hs.436416
A_21_P000	-3.110924	-1.637343	3.110924	down	0.839804	0.539001	0.63281	-1.399739	-0.539001	-0.961674	lnc-ATAD5-	lnc-ATAD5-	FALSE	LNCipedia lincRNA (lnc-ATAD5-3),			29070198	chr17	29070139	+	hs|17q11.2		chr17:29070139-29070198		
A_22_P000	-3.716111	-1.893794	3.716111	down	0.705254	0.865445	0.730469	-1.327684	-0.705254	-1.347274			FALSE				50683115	chr16	50683056	+	hs|16q12.1		chr16:50683056-50683115		
A_22_P000	-2.095182	-1.067075	2.095182	down	0.701175	0.376872	0.37251	-0.37251	-0.74696	-0.631199	HNRNPU-A	HNRNPU ai	FALSE	Homo sapi	284702		2.45E+08	chr1	2.45E+08	-	hs|1q44	NR_026778	chr1:24500	NR_026778	Hs.743421
A_21_P001	-2.046285	-1.033007	2.046285	down	0.378557	0.403476	0.396786	-0.69491	-0.378557	-0.846736	LOC101928	serine/thre	FALSE	PREDICTED	1.02E+08		1.28E+08	chr7	1.28E+08	-	hs|7q32.1		chr7:12817	XR_428252	Hs.445078
A_22_P000	-2.505386	-1.325033	2.505386	down	0.595717	0.57893	0.561846	-0.894944	-0.561846	-0.781816			FALSE	AU253022 human favorable neuro			1.01E+08	chr14	1.01E+08	+	hs|14q32.3	AU253022	chr14:101430703-1014		Hs.118101
A_33_P329	-2.08513	-1.060137	2.08513	down	0.420196	0.394228	0.418158	-0.721447	-0.394228	-0.832154	HAS1	hyaluronar	FALSE	Homo sapi	3036	GO:000588	52216452	chr19	52216393	-	hs|19q13.4	NM_00152	chr19:5221	NM_00152	Hs.57697

A_24_P335	-3.108875	-1.636393	3.108875	down	0.812012	0.744298	0.74803	-0.794024	-1.066514	-0.744298	OAS3	2'-5'-oligoa	FALSE	Homo sapi	4940	GO:000372	1.13E+08	chr12	1.13E+08	+	hs|12q24.1	NM_00618	chr12:1134	NM_00618	Hs.744211
A_33_P325	-3.951055	-1.982238	3.951055	down	0.985494	1.012643	0.977274	-0.987329	-1.0067	-0.977274	GGT5	gamma-glu	FALSE	Homo sapi	2687	GO:001936	24615682	chr22	24615623	-	hs|22q11.2	NM_00109	chr22:2461	NM_00109	Hs.437156
A_21_P001	-3.206501	-1.681	3.206501	down	0.74004	0.792022	0.768986	-0.919878	-1.082033	-0.74004	LINC01088	long interg	FALSE	Homo sapi	1.01E+08		80007416	chr4	80007360	+	hs|4q21.21	NR_038342	chr4:80007	NR_038342	Hs.480055
A_33_P325	-2.120925	-1.084694	2.120925	down	0.472263	0.513955	0.431459	-0.675484	-0.431459	-0.729461	COMTD1	catechol-O	FALSE	Homo sapi	118881	GO:000573	76993805	chr10	76993746	-	hs|10q22.2	NM_14458	chr10:7699	NM_14458	Hs.355333
A_33_P322	-2.402329	-1.264434	2.402329	down	0.661889	0.636977	0.585232	-0.614659	-0.709313	-0.585232	EIF2AK2	eukaryotic	FALSE	Homo sapi	5610	GO:000551	37368837	chr2	37368778	-	hs|2p22.2	NM_00113	chr2:37368	NM_00113	Hs.131431
A_23_P648	-2.957547	-1.564401	2.957547	down	0.564374	0.803868	0.682674	-1.050174	-0.564374	-1.027739	KCNJ8	potassium	FALSE	Homo sapi	3764	GO:000524	21918724	chr12	21918665	-	hs|12p12.1	NM_00498	chr12:2191	NM_00498	Hs.102308
A_22_P000	-2.434068	-1.283369	2.434068	down	0.559677	0.633886	0.663772	-0.78725	-0.559677	-0.645847	RPS6KA2-IT	RPS6KA2 ir	FALSE	Homo sapi	1.01E+08		1.67E+08	chr6	1.67E+08	-	hs|6q27	NR_046793	chr6:16687	NR_046793	Hs.675698
A_21_P001	-2.897645	-1.534881	2.897645	down	0.723684	0.800271	0.68932	-0.68932	-0.988262	-0.713786			FALSE	nuclear paraspeckle assembly tran			65194182	chr11	65194123	+	hs|11q13.1		chr11:65194123-65194		Hs.523789
A_19_P003	-2.076768	-1.05434	2.076768	down	0.38882	0.455462	0.455365	-0.754516	-0.38882	-0.720037	TUG1	taurine up-	FALSE	Homo sapi	55000		31374064	chr22	31374005	+	hs|22q12.2	NR_110492	chr22:3137	NR_110492	Hs.554829
A_21_P000	-2.016281	-1.011697	2.016281	down	0.626054	0.584976	0.413288	-0.413288	-0.447391	-0.550093			FALSE				64440505	chr3	64440446	+	hs|3p14.1		chr3:64440446-64440505		
A_23_P870	-2.493299	-1.318056	2.493299	down	0.56798	0.588092	0.586628	-0.571772	-1.071715	-0.56798	TAGLN	transgelin	FALSE	Homo sapi	6876	GO:000551	1.17E+08	chr11	1.17E+08	+	hs|11q23.3	NM_00100	chr11:1170	NM_00100	Hs.410977
A_24_P488	-2.40554	-1.266361	2.40554	down	0.546402	0.523916	0.574269	-0.819444	-0.523916	-0.811135	APOL2	apolipopro	FALSE	Homo sapi	23780	GO:000662	36622708	chr22	36622649	-	hs|22q12.3	NM_14563	chr22:3662	NM_14563	Hs.474740
A_24_P318	-3.546179	-1.826266	3.546179	down	0.942122	0.952126	0.833006	-1.017541	-0.833006	-0.900996	ITGB3	integrin, be	FALSE	Homo sapi	3690	GO:000551	45389086	chr17	45389027	+	hs|17q21.3	NM_00021	chr17:4538	NM_00021	Hs.218040
A_33_P328	-2.095062	-1.066993	2.095062	down	0.486236	0.519057	0.567215	-0.569952	-0.572282	-0.486236			FALSE				15201431	chr16	15201372	-	hs|16p13.11		chr16:015201431-015201372		
A_33_P329	-2.877751	-1.524942	2.877751	down	0.762181	0.63815	0.706353	-1.137516	-0.692475	-0.63815			FALSE				3105573	chr11	3105514	-	hs|11p15.4		chr11:003105573-003105514		
A_33_P321	-2.322466	-1.215657	2.322466	down	0.543446	0.529355	0.506187	-0.748262	-0.506187	-0.813535	DNAJC6	DnaJ (Hsp4	FALSE	Homo sapi	9829	GO:007231	65880860	chr1	65880801	+	hs|1p31.3	NM_00125	chr1:65880	NM_00125	Hs.647643
A_23_P363	-5.404071	-2.434047	5.404071	down	1.171785	1.202571	1.204436	-1.171785	-1.208722	-1.342842	FRZB	frizzled-rela	FALSE	Homo sapi	2487	GO:004281	1.84E+08	chr2	1.84E+08	-	hs|2q32.1	NM_00146	chr2:18369	NM_00146	Hs.128453
A_19_P008	-5.393584	-2.431244	5.393584	down	0.824458	0.936858	0.946295	-1.834264	-0.824457	-1.927401	CASC15	cancer susc	FALSE	Homo sapi	401237		22111058	chr6	22110999	+	hs|6p22.3	NR_015410	chr6:22110	NR_015410	Hs.712707
A_33_P335	-2.771454	-1.470643	2.771454	down	0.810918	0.721753	0.703323	-0.703323	-0.730863	-0.741751	SSC5D	scavenger r	FALSE	Homo sapi	284297	GO:200048	56030410	chr19	56030351	+	hs|19q13.4	NM_00114	chr19:5603	NM_00114	Hs.554182
A_23_P391	-12.81319	-3.679558	12.81319	down	1.844648	1.71038	1.672811	-2.142335	-1.672811	-1.995689	PLXDC1	plexin dom	FALSE	Homo sapi	57125	GO:000551	37223475	chr17	37223416	-	hs|17q12	NM_02040	chr17:3722	NM_02040	Hs.125036
A_23_P666	-2.416378	-1.272846	2.416378	down	0.667491	0.600575	0.636468	-0.600575	-0.638208	-0.675222	KAT2A	K(lysine) ac	FALSE	Homo sapi	2648	GO:000551	40265294	chr17	40265235	-	hs|17q21.2	NM_02107	chr17:4026	NM_02107	Hs.463045
A_24_P270	-4.104347	-2.037153	4.104347	down	0.98979	0.934695	0.917045	-1.139587	-0.917045	-1.213295	IFI27	interferon,	FALSE	Homo sapi	3429	GO:000110	94583026	chr14	94582967	+	hs|14q32.1	NM_00553	chr14:9458	NM_00553	Hs.532634
A_23_P104	-3.32111	-1.731665	3.32111	down	0.573786	0.78333	0.942088	-1.074704	-0.573786	-1.247303	KIRREL3	kin of IRRE	FALSE	Homo sapi	84623	GO:003009	1.26E+08	chr11	1.26E+08	-	hs|11q24.2	NM_03253	chr11:1262	NM_03253	Hs.376015
A_23_P794	-2.343287	-1.228534	2.343287	down	0.550975	0.552379	0.582998	-0.641674	-0.550975	-0.8066	STON1-GTF	STON1-GTF	FALSE	Homo sapi	286749		48906615	chr2	48906556	+	hs|2p16.3	NM_17231	chr2:48906	NM_17231	Hs.732016
A_24_P137	-2.354604	-1.235484	2.354604	down	0.607018	0.594027	0.624086	-0.679792	-0.594027	-0.607503	USP53	ubiquitin sp	FALSE	Homo sapi	54532	GO:000815	1.2E+08	chr4	1.2E+08	+	hs|4q26	NM_01905	chr4:12021	NM_01905	Hs.431081
A_23_P102	-2.03289	-1.023532	2.03289	down	0.451759	0.45528	0.44977	-0.57983	-0.44977	-0.684187	SLC40A1	solute carri	FALSE	Homo sapi	30061	GO:000551	1.9E+08	chr2	1.9E+08	-	hs|2q32.2	NM_01458	chr2:19042	NM_01458	Hs.643005
A_23_P358	-2.380425	-1.251219	2.380425	down	0.654191	0.598828	0.550276	-0.550276	-0.646316	-0.75377	GOLGA6L4	golgin A6 fa	FALSE	Homo sapi	643707		84908786	chr15	84908727	+	hs|15q25.2	NM_00126	chr15:8490	NM_00126	Hs.534573
A_23_P424	-2.003825	-1.002756	2.003825	down	0.366676	0.475221	0.524767	-0.653877	-0.366676	-0.621052	TCF20	transcriptic	FALSE	Homo sapi	6942	GO:000635	42556763	chr22	42556704	-	hs|22q13.2	NM_00565	chr22:4255	NM_00565	Hs.475018
A_23_P119	-2.464189	-1.301113	2.464189	down	0.510126	0.5559	0.5513	-0.930787	-0.510126	-0.845101	KLF2	Kruppel-lik	FALSE	Homo sapi	10365	GO:005124	16438305	chr19	16438246	+	hs|19p13.1	NM_01627	chr19:1643	NM_01627	Hs.744182
A_21_P000	-3.213106	-1.683969	3.213106	down	0.770185	0.763979	0.76775	-1.057501	-0.928512	-0.763979	SNORD114	small nucle	FALSE	Homo sapi	767590		1.01E+08	chr14	1.01E+08	+	hs|14q32.3	NR_003205	chr14:1014	NR_003205	
A_23_P130	-2.347559	-1.231162	2.347559	down	0.577322	0.536858	0.642073	-0.727144	-0.536858	-0.67323	ROCK1	Rho-associa	FALSE	Homo sapi	6093	GO:000551	18535191	chr18	18535132	-	hs|18q11.1	NM_00540	chr18:1853	NM_00540	Hs.306307
A_33_P321	-2.492955	-1.317857	2.492955	down	0.499438	0.490288	0.478592	-1.150647	-0.478592	-0.856013	ATP7A	ATPase, Cu	FALSE	Homo sapi	538	GO:000580	77302373	chrX	77302314	+	hs|Xq21.1	NM_00005	chrX:77302	NM_00005	Hs.496414
A_21_P001	-2.672764	-1.418332	2.672764	down	0.562059	0.55592	0.483948	-1.217765	-0.483948	-0.951359	XLOC_l2_009136		FALSE	BROAD Institute lincRNA (XLOC_l2			15304669	chr21	15304586	-	hs|21q11.2		chr21:15304669-15304586		
A_21_P000	-2.82804	-1.499802	2.82804	down	0.649717	0.884092	0.656048	-0.649717	-0.75455	-0.905284	lnc-IRX3-4	lnc-IRX3-4:	FALSE	Q5CSV7_CRYPV (Q5CSV7) Predicte			54953949	chr16	54953890	-	hs|16q12.2		chr16:54953949-54953890		
A_22_P000	-2.078715	-1.055692	2.078715	down	0.428688	0.542305	0.549719	-0.647851	-0.569826	-0.428688	SNHG23	small nucle	FALSE	small nucle	1.01E+08		1.01E+08	chr14	1.01E+08	+	hs|14q32.3	XR_424548	chr14:1014	XR_424548	Hs.709410
A_22_P000	-2.024084	-1.017269	2.024084	down	0.63378	0.409371	0.667982	-0.475755	-0.409371	-0.455547	lnc-CLCN6-	lnc-CLCN6-	FALSE	Q5JZE1_HUMAN (Q5JZE1) Natriure			11908136	chr1	11908077	+	hs|1p36.22		chr1:11908077-11908136		
A_23_P161	-4.598386	-2.201128	4.598386	down	0.975009	1.025772	1.095543	-1.210692	-1.321357	-0.975009	LEPR	leptin recep	FALSE	Homo sapi	3953	GO:004572	66102188	chr1	66102129	+	hs|1p31.3	NM_00230	chr1:66102	NM_00230	Hs.723178
A_24_P937	-2.637399	-1.399116	2.637399	down	0.555601	0.664454	0.621488	-0.934185	-0.5556	-0.86602	PRSS23	protease, s	FALSE	Homo sapi	11098	GO:000425	86522121	chr11	86522062	+	hs|11q14.2	NM_00717	chr11:8652	NM_00717	Hs.25338
A_23_P743	-2.390517	-1.257323	2.390517	down	0.427728	0.639642	0.420478	-0.716004	-0.420478	-1.147638	NUF2	NUF2, NDC	FALSE	Homo sapi	83540	GO:000551	1.63E+08	chr1	1.63E+08	+	hs|1q23.3	NM_14569	chr1:16332	NM_14569	Hs.651950
A_23_P435	-2.673825	-1.418905	2.673825	down	0.58497	0.627309	0.599752	-0.896425	-0.584971	-0.963288	WSB1	WD repeat	FALSE	Homo sapi	26118	GO:003555	25639727	chr17	25639668	+	hs|17q11.1	NM_01562	chr17:2563	NM_01562	Hs.446017
A_23_P149	-3.468732	-1.794408	3.468732	down	0.978402	0.892416	0.849152	-0.888161	-0.849152	-0.925942	FMO1	flavin conta	FALSE	Homo sapi	2326	GO:005066	1.71E+08	chr1	1.71E+08	+	hs|1q24.3	NM_00202	chr1:17125	NM_00202	Hs.1424
A_33_P321	-2.43251	-1.282446	2.43251	down	0.602693	0.591361	0.484277	-0.778232	-0.484277	-0.906499	P4HA1	prolyl 4-hy	FALSE	Homo sapi	5033	GO:000551	74767142	chr10	74767083	-	hs|10q22.1	NM_00114	chr10:7476	NM_00114	Hs.500047
A_21_P000	-6.21117	-2.634865	6.21117	down	1.081139	1.057568	1.040685	-1.764111	-1.040685	-1.920407			FALSE				1.48E+08	chr2	1.48E+08	+	hs|2q22.3		chr2:147606877-147607878		
A_21_P000	-2.225647	-1.154225	2.225647	down	0.549696	0.546713	0.354413	-0.987207	-0.354413	-0.670232	lnc-TNFRSF	lnc-TNFRSF	FALSE	LNCipedia lincRNA (lnc-TNFRSF9-1			8066636	chr1	8066577	-	hs|1p36.23		chr1:8066636-8066577		
A_22_P000	-2.070896	-1.050255	2.070896	down	0.431068	0.386491	0.566172	-0.663266	-0.386491	-0.717277	lnc-ENPP1-	lnc-ENPP1-	FALSE	DB576317 RIKEN full-length enrich			1.32E+08	chr6	1.32E+08	+	hs|6q23.2	DB576317	chr6:132398474-13239		Hs.730533
A_23_P770	-3.357513	-1.747393	3.357513	down	0.838481	0.797357	0.814105	-0.973983	-0.797357	-1.020898	SLC25A29	solute carri	FALSE	Homo sapi	123096	GO:000574	1.01E+08	chr14	1.01E+08	-	hs|14q32.2	NM_00103	chr14:1007	NM_00103	Hs.578109
A_33_P328	-2.571008	-1.362334	2.571008	down	0.585472	0.653543	0.674022	-0.902047	-0.585472	-0.686444	ALDH1L2	aldehyde d	FALSE	Homo sapi	160428	GO:000573	1.05E+08	chr12	1.05E+08	-	hs|12q23.3	NM_00103	chr12:1054	NM_00103	Hs.42572
A_33_P340	-2.653671	-1.40799	2.653671	down	0.692578	0.656023	0.686379	-0.77515	-0.656023	-0.757815	HELZ2	helicase wi	FALSE	Homo sapi	85441	GO:000551	62189535	chr20	62189476	-	hs|20q13.3	NM_00103	chr20:6218	NM_00103	Hs.517180
A_24_P350	-2.028501	-1.020414	2.028501	down	0.560749	0.396942	0.559988	-0.441656	-0.396942	-0.704965	RNF150	ring finger	FALSE	Homo sapi	57484	GO:000827	1.42E+08	chr4	1.42E+08	-	hs|4q31.21	NM_02072	chr4:14178	NM_02072	Hs.659104
A_33_P334	-2.324349	-1.216827	2.324349	down	0.484412	0.738943	0.637196	-0.796657	-0.484412	-0.508859	PRSS53	protease, s	FALSE	Homo sapi	339105	GO:000425	31094871	chr16	31094812	-	hs|16p11.2	NM_00103	chr16:3109	NM_00103	Hs.569575
A_21_P000	-2.582278	-1.368645	2.582278	down	0.755299	0.669194	0.603454	-0.673887	-0.603454	-0.800646	SNORA16B	small nucle	FALSE	Homo sapi	692157		2.13E+08	chr1	2.13E+08	+	hs|1q32.3	NR_004389	chr1:21252	NR_004389	Hs.707958
A_23_P522	-2.841841	-1.506826	2.841841	down	0.657594	0.731881	0.708639	-0.881803	-0.657594	-0.882967	IFIT1	interferon-	FALSE	Homo sapi	3434	GO:000551	91163497	chr10	91163438	+	hs|10q23.3	NM_00154	chr10:9116	NM_00154	Hs.20315
A_23_P168	-2.607282	-1.382546	2.607282	down	0.735703	0.939559	0.584561	-0.584561	-0.680543	-0.622711	C7orf69	chromoson	FALSE	Homo sapi	80099	GO:000557	47859384	chr7	47859325	+	hs|7p12.3	NM_02503	chr7:47859	NM_02503	Hs.287647
A_23_P128	-2.648448	-1.405147	2.648448	down	0.446621	0.509602	0.547961	-1.046542	-0.446621	-1.218095	SOCS2	suppressor	FALSE	Homo sapi	8835	GO:000551	93969858	chr12	93969799	+	hs|12q22	NM_00387	chr12:9396	NM_00387	Hs.737206
A_23_P106	-2.424728	-1.277823	2.424728	down	0.673474	0.571989	0.702345	-0.642818	-0.670855	-0.571989	SEMA7A	semaphorin	FALSE	Homo sapi	8482	GO:000551	74702424	chr15	74702365	-	hs|15q24.1	NM_00361	chr15:7470	NM_00361	Hs.24640
A_24_P295	-2.528602	-1.33834	2.528602	down	0.668457	0.646247	0.603636	-0.746072	-0.746973	-0.603636	RASSF4	Ras associa	FALSE	Homo sapi	83937	GO:000551	45489827	chr10	45489768	+	hs|10q11.2	NM_03202	chr10:4548	NM_03202	Hs.522895
A_33_P333	-2.147863	-1.102902	2.147863	down	0.352873	0.560687	0.433873	-0.864294	-0.352873	-0.744106	SHROOM3	shroom far	FALSE	Homo sapi	57619	GO:000738	77704323	chr4	77704264	+	hs|4q21.1	NM_02085	chr4:77704	NM_02085	Hs.702168
A_24_P243	-6.79482	-2.764435	6.79482	down	1.298973	1.23062	1.314017	-1.68908	-1.23062	-1.529995	PDK4	pyruvate d	FALSE	Homo sapi	5166	GO:000828	95213366	chr7	95213307	-	hs|7q21.3	NM_00261	chr7:95213	NM_00261	Hs.8364

A_33_P339	-2.041954	-1.029951	2.041954	down	0.538187	0.694859	0.416718	-0.467837	-0.555534	-0.416718	FAM178A	family with	FALSE	Homo sapi	55719	GO:000563	1.03E+08	chr10	1.03E+08	+	hs|10q24.3	NM_00124	chr10:1026	NM_00124	Hs.447458
A_24_P932	-9.833298	-3.297675	9.833298	down	1.339407	1.42568	1.507056	-2.09684	-1.339407	-2.184637	HMBOX1	homeobox	FALSE	homeobox	79618	GO:000551	28910521	chr8	28910462	+	hs|8p12	XM_00527	chr8:2891C	XM_005273639	
A_24_P277	-2.051124	-1.036415	2.051124	down	0.627151	0.350267	0.501972	-0.634666	-0.350267	-0.644922	CXCL5	chemokine	FALSE	Homo sapi	6374	GO:000800	74861957	chr4	74861898	-	hs|4q13.3	NM_00299	chr4:74861	NM_00299	Hs.89714
A_23_P968	-2.054233	-1.0386	2.054233	down	0.434732	0.456691	0.657757	-0.590671	-0.541216	-0.434732	SLAMF9	SLAM fami	FALSE	Homo sapi	89886	GO:000487	1.6E+08	chr1	1.6E+08	-	hs|1q23.2	NM_03343	chr1:15992	NM_03343	Hs.661712
A_33_P334	-4.071724	-2.02564	4.071724	down	0.882649	0.790226	0.698386	-1.429308	-0.698386	-1.577965	SPEN	spen family	FALSE	Homo sapi	23013	GO:000119	16255167	chr1	16255108	+	hs|1p36.13	NM_01500	chr1:16255	NM_01500	Hs.744843
A_23_P214	-2.395162	-1.260123	2.395162	down	0.613267	0.594928	0.632494	-0.688741	-0.594928	-0.656011	FBN2	fibrillin 2	FALSE	Homo sapi	2201	GO:000551	1.28E+08	chr5	1.28E+08	-	hs|5q23.3	NM_00199	chr5:12759	NM_00199	Hs.519294
A_23_P112	-2.572871	-1.363379	2.572871	down	0.570676	0.592769	0.636311	-0.834123	-0.570676	-0.885583	DNAJB5	DnaJ (Hsp4	FALSE	Homo sapi	25822	GO:000698	34998340	chr9	34998281	+	hs|9p13.3	NM_01226	chr9:34998	NM_01226	Hs.237506
A_32_P767	-2.601141	-1.379144	2.601141	down	0.739555	0.648241	0.577413	-0.698977	-0.895835	-0.577413	NT5DC3	5'-nucleoti	FALSE	Homo sapi	51559	GO:004323	1.04E+08	chr12	1.04E+08	-	hs|12q23.3	NM_00103	chr12:1041	NM_00103	Hs.48428
A_33_P332	-2.383125	-1.252854	2.383125	down	0.464413	0.478998	0.520374	-0.934925	-0.464413	-0.895439	HR	hair growth	FALSE	Homo sapi	55806	GO:001660	21972023	chr8	21971964	-	hs|8p21.3	NM_00514	chr8:21972	NM_00514	Hs.272367
A_21_P001	-2.077777	-1.055041	2.077777	down	0.505037	0.582913	0.369647	-0.690293	-0.369647	-0.647585	LINC01296	long interg	FALSE	Homo sapi	642477		19925300	chr14	19925241	-	hs|14q11.2	NR_12211	chr14:1992	NR_12211	Hs.744284
A_23_P11C	-2.171084	-1.118415	2.171084	down	0.580155	0.589635	0.5316	-0.540459	-0.581798	-0.5316	FST	follistatin	FALSE	Homo sapi	10468	GO:000551	52780896	chr5	52780837	+	hs|5q11.2	NM_01340	chr5:5278C	NM_01340	Hs.9914
A_23_P118	-2.102929	-1.0724	2.102929	down	0.379904	0.408667	0.439306	-0.828341	-0.379905	-0.781077	CAMK2N1	calcium/ca	FALSE	Homo sapi	55450	GO:003005	20809460	chr1	20809401	-	hs|1p36.12	NM_01858	chr1:20809	NM_01858	Hs.731383
A_21_P001	-2.164073	-1.113749	2.164073	down	0.617726	0.540543	0.431262	-0.715188	-0.431262	-0.605268	BMS1P6	BMS1 pseu	FALSE	Homo sapi	642826		48200409	chr10	48200350	+	hs|10q11.2	NR_02449	chr10:4820	NR_02449	Hs.711898
A_23_P162	-2.227497	-1.155424	2.227497	down	0.3676	0.459615	0.483394	-0.885129	-0.3676	-0.902933	ACTR6	ARP6 actin-	FALSE	Homo sapi	64431	GO:000551	1.01E+08	chr12	1.01E+08	+	hs|12q23.1	NM_02249	chr12:1006	NM_02249	Hs.115088
A_22_P00C	-2.095452	-1.067261	2.095452	down	0.503027	0.681859	0.343844	-0.634844	-0.343844	-0.694365			FALSE	Q3AUE7_CHLCH (Q3AUE7) Glucos			65220537	chr5	65220478	-	hs|5q12.3		chr5:65220537-65220478		
A_23_P11C	-2.282111	-1.190369	2.282111	down	0.527268	0.590201	0.811057	-0.527268	-0.541204	-0.574108	HERC5	HECT and F	FALSE	Homo sapi	51191	GO:005068	89426948	chr4	89426889	+	hs|4q22.1	NM_01632	chr4:89426	NM_01632	Hs.26663
A_24_P267	-2.092317	-1.065101	2.092317	down	0.300257	0.46363	0.348727	-0.695597	-0.300257	-1.086836	SAMHD1	SAM doma	FALSE	Homo sapi	25939	GO:000551	35533863	chr20	35533804	-	hs|20q11.2	NM_01547	chr20:3553	NM_01547	Hs.580681
A_23_P119	-2.241119	-1.164219	2.241119	down	0.471798	0.501835	0.46963	-0.7107	-0.46963	-0.869063	SLC39A10	solute carri	FALSE	Homo sapi	57181	GO:004687	1.97E+08	chr2	1.97E+08	+	hs|2q32.3	NM_02034	chr2:1966C	NM_02034	Hs.650158
A_24_P131	-6.650048	-2.733365	6.650048	down	1.052082	1.052836	1.085473	-1.921111	-1.052082	-2.036509	CD86	CD86 mole	FALSE	Homo sapi	942	GO:000551	1.22E+08	chr3	1.22E+08	+	hs|3q13.33	NM_00688	chr3:12183	NM_00688	Hs.171182
A_33_P322	-2.303274	-1.203686	2.303274	down	0.450765	0.513855	0.528719	-0.856933	-0.450765	-0.810023	COL4A4	collagen, ty	FALSE	Homo sapi	1286	GO:002261	2.28E+08	chr2	2.28E+08	-	hs|2q36.3	NM_00009	chr2:22786	NM_00009	Hs.591645
A_24_P857	-2.435974	-1.284499	2.435974	down	0.546613	0.79469	0.46391	-0.564515	-1.019859	-0.46391	THEMIS2	thymocyte	FALSE	Homo sapi	9473	GO:005085	28212522	chr1	28212463	+	hs|1p35.3	NM_00103	chr1:28212	NM_00103	Hs.10649
A_23_P155	-2.103796	-1.072995	2.103796	down	0.480449	0.51438	0.570964	-0.604068	-0.480449	-0.568675	FMO3	flavin conta	FALSE	Homo sapi	2328	GO:005066	1.71E+08	chr1	1.71E+08	+	hs|1q24.3	NM_00100	chr1:17108	NM_00100	Hs.445350
A_33_P326	-2.050133	-1.035718	2.050133	down	0.339842	0.701212	0.686625	-0.363673	-0.67596	-0.339842	SLC17A5	solute carri	FALSE	Homo sapi	26503	GO:000682	74345187	chr6	74345128	-	hs|6q13	NM_01243	chr6:74345	NM_01243	Hs.597422
A_33_P33C	-2.472605	-1.306032	2.472605	down	0.627212	0.678888	0.604535	-0.604535	-0.701148	-0.70178	SSC5D	scavenger r	FALSE	Homo sapi	284297	GO:200048	56030362	chr19	56030303	+	hs|19q13.4	NM_00114	chr19:5603	NM_00114	Hs.554182
A_24_P385	-2.228438	-1.156033	2.228438	down	0.527419	0.58779	0.48896	-0.624052	-0.48896	-0.750917	PTPRF	protein tyr	FALSE	Homo sapi	5792	GO:001647	44089242	chr1	44089183	+	hs|1p34.2	NM_00284	chr1:44089	NM_00284	Hs.272062
A_23_P13C	-2.301498	-1.202573	2.301498	down	0.613097	0.607237	0.497459	-0.698033	-0.497459	-0.694435	PIEZO2	piezo-type	FALSE	Homo sapi	63895	GO:000838	10671274	chr18	10671215	-	hs|18p11.2	NM_02206	chr18:1067	NM_02206	Hs.436902
A_33_P322	-3.223741	-1.688736	3.223741	down	0.759761	0.79033	0.871907	-0.802719	-1.081729	-0.759761	PCDH18	protocadhe	FALSE	Homo sapi	54510	GO:000588	1.38E+08	chr4	1.38E+08	-	hs|4q28.3	NM_00130	chr4:13845	NM_00130	Hs.591691
A_21_P00C	-2.403798	-1.265315	2.403798	down	0.562126	0.665317	0.649602	-0.707764	-0.562125	-0.649013	SNORA16A	small nucle	FALSE	Homo sapi	692073		28907491	chr1	28907432	-	hs|1p35.3	NR_00303	chr1:28907	NR_00303	Hs.632377
A_23_P348	-2.386496	-1.254894	2.386496	down	0.517486	0.530433	0.523705	-0.587818	-1.087752	-0.517486	MKL1	megakaryo	FALSE	Homo sapi	57591	GO:000551	40806886	chr22	40806827	-	hs|22q13.1	NM_02083	chr22:408C	NM_02083	Hs.654688
A_23_P161	-2.126462	-1.088455	2.126462	down	0.615804	0.482283	0.443425	-0.443425	-0.819595	-0.460832	MOV10	Mov10 RIS	FALSE	Homo sapi	4343	GO:000551	1.13E+08	chr1	1.13E+08	+	hs|1p13.2	NM_02096	chr1:11324	NM_02096	Hs.514941
A_23_P349	-3.170637	-1.664773	3.170637	down	0.786272	0.84161	0.868061	-0.883872	-0.786272	-0.828232	CCDC85A	coiled-coil	FALSE	Homo sapi	114800		56612864	chr2	56612805	+	hs|2p16.1	NM_00108	chr2:56612	NM_00108	Hs.117136
A_33_P337	-2.565338	-1.359149	2.565338	down	0.537611	0.743369	0.640901	-0.694176	-0.537611	-0.92378	MKI67	marker of p	FALSE	Homo sapi	4288	GO:000551	1.3E+08	chr10	1.3E+08	-	hs|10q26.2	NM_00241	chr10:1299	NM_00241	Hs.80976
A_21_P00C	-2.10943	-1.076853	2.10943	down	0.444616	0.402645	0.565837	-0.840376	-0.402645	-0.574441	FLJ42351	uncharacte	FALSE	Homo sapi	400999		1.13E+08	chr2	1.13E+08	-	hs|2q13	NR_03387	chr2:11339	NR_03387	Hs.635980
A_21_P00C	-3.985842	-1.994885	3.985842	down	0.894434	0.968341	1.002457	-0.894434	-1.0888	-1.136188	SNORD114	small nucle	FALSE	Homo sapi	767600		1.01E+08	chr14	1.01E+08	+	hs|14q32.3	NR_00321	chr14:1014	NR_003215	
A_21_P00C	-2.605825	-1.38174	2.605825	down	0.506537	0.603664	0.473082	-0.690677	-0.473082	-1.398178			FALSE	PREDICTED: Homo sapiens unchara			69187201	chr11	69187142	+	hs|11q13.3	XR_252919	chr11:6918	XR_252919	
A_33_P325	-2.005362	-1.003862	2.005362	down	0.455668	0.392924	0.469986	-0.392924	-0.899313	-0.400773	PHLDA3	pleckstrin h	FALSE	Homo sapi	23612	GO:005189	2.01E+08	chr1	2.01E+08	-	hs|1q32.1	NM_01239	chr1:20143	NM_01239	Hs.268557
A_23_P356	-8.76104	-3.131102	8.76104	down	1.386137	1.588813	1.491231	-1.386137	-1.893703	-1.647286	KCNIP3	Kv channel	FALSE	Homo sapi	30818	GO:000588	96051825	chr2	96051766	+	hs|2q11.1	NM_01343	chr2:96051	NM_01343	Hs.437376
A_33_P344	-2.688851	-1.42699	2.688851	down	0.634328	0.662395	0.702581	-0.768936	-0.634328	-0.878401	lnc-WDR1-	lnc-WDR1-	FALSE	Homo sapiens mRNA; cDNA DKFZp			10069830	chr4	10069771	-	hs|4p16.1	AL713660	chr4:10069830-100697		Hs.618463
A_24_P348	-2.763121	-1.466299	2.763121	down	0.720205	0.66163	0.726046	-0.728478	-0.66163	-0.900908	CCNK	cyclin K	FALSE	Homo sapi	8812	GO:000551	99959921	chr14	99959862	+	hs|14q32.2	NM_00109	chr14:9995	NM_00109	Hs.510409
A_32_P108	-3.084158	-1.624877	3.084158	down	0.568071	0.614547	0.534364	-1.315603	-0.534365	-1.30768	AK4	adenylate k	FALSE	Homo sapi	205	GO:004603	65692691	chr1	65692632	+	hs|1p31.3	NM_00100	chr1:65692	NM_00100	Hs.10862
A_22_P00C	-5.367556	-2.424265	5.367556	down	0.785647	0.979697	0.967763	-1.812218	-0.785647	-1.941825	FAM155A-I	FAM155A i	FALSE	Homo sapi	1.01E+08		1.08E+08	chr13	1.08E+08	-	hs|13q33.3	NR_04684	chr13:1084	NR_04684	Hs.653972
A_24_P136	-2.988926	-1.579627	2.988926	down	0.646225	0.842677	0.75999	-0.9843	-0.646225	-0.859467	SLC8A1	solute carri	FALSE	Homo sapi	6546	GO:006040	40657389	chr2	40657330	-	hs|2p22.1	NM_02109	chr2:40657	NM_02109	Hs.468274
A_22_P00C	-2.098	-1.069015	2.098	down	0.471688	0.573945	0.476033	-0.613297	-0.600394	-0.471688	SLC25A29	solute carri	FALSE	Homo sapi	123096	GO:000574	1.01E+08	chr14	1.01E+08	-	hs|14q32.2	NM_00129	chr14:1007	NM_00129	Hs.578109
A_24_P381	-2.039764	-1.028402	2.039764	down	0.522045	0.370561	0.557321	-0.642736	-0.370561	-0.621982	TRIM6	tripartite m	FALSE	Homo sapi	117854	GO:000367	5633926	chr11	5633867	+	hs|11p15.4	NM_00100	chr11:5633	NM_00100	Hs.729048
A_23_P656	-2.157879	-1.109614	2.157879	down	0.575929	0.472878	0.558898	-0.472878	-0.667746	-0.580511	TMOD3	tropomodu	FALSE	Homo sapi	29766	GO:000693	52192444	chr15	52192385	+	hs|15q21.2	NM_01454	chr15:5219	NM_01454	Hs.4998
A_33_P324	-2.292843	-1.197137	2.292843	down	0.384286	0.516084	0.64062	-0.522713	-1.143422	-0.384286	ESRRA	estrogen-re	FALSE	Homo sapi	2101	GO:000551	64083420	chr11	64083361	+	hs|11q13.1	NM_00128	chr11:6408	NM_00128	Hs.110849
A_21_P001	-2.009636	-1.006934	2.009636	down	0.531137	0.307092	0.486091	-0.655118	-0.307092	-0.734273	LOC102725	uncharacte	FALSE	PREDICTED	1.03E+08		68746026	chr9	68745967	+	hs|9q21.11		chr9:68745	XR_432427	
A_21_P001	-2.126051	-1.088176	2.126051	down	0.486735	0.476338	0.477403	-0.688497	-0.476338	-0.659218	MEG9	maternally	FALSE	Homo sapi	1.01E+08		1.02E+08	chr14	1.02E+08	+	hs|14q32.3	NR_04766	chr14:1015	NR_04766	Hs.301755
A_23_P309	-2.086725	-1.061241	2.086725	down	0.518916	0.558512	0.408772	-0.408772	-0.793304	-0.495447	HECTD4	HECT doma	FALSE	Homo sapi	283450	GO:000573	1.13E+08	chr12	1.13E+08	-	hs|12q24.1	NM_00110	chr12:1126	NM_00110	Hs.530943
A_21_P00C	-2.380493	-1.25126	2.380493	down	0.614408	0.761058	0.183478	-0.183478	-0.751514	-1.259845	SPATA3-AS	SPATA3 an	FALSE	SPATA3 an	348761		2.32E+08	chr2	2.32E+08	-	hs|2q37.1		chr2:231857053-231856697		
A_23_P324	-5.800157	-2.536092	5.800157	down	1.126929	1.137264	1.163179	-1.596873	-1.126928	-1.457102	ISG20	interferon	FALSE	Homo sapi	3669	GO:003061	89198843	chr15	89198784	+	hs|15q26.1	NM_00220	chr15:8919	NM_00220	Hs.459265
A_21_P001	-4.323799	-2.112299	4.323799	down	0.828639	0.911905	0.951274	-0.828639	-1.107808	-1.708634	AK4	adenylate k	FALSE	Homo sapi	205	GO:004603	65691857	chr1	65691798	+	hs|1p31.3	NM_00100	chr1:65691	NM_00100	Hs.10862
A_33_P329	-2.478279	-1.309339	2.478279	down	0.500942	0.798807	0.645211	-0.90643	-0.500942	-0.575683	OPN3	opsin 3	FALSE	Homo sapi	23596	GO:001829	2.42E+08	chr1	2.42E+08	-	hs|1q43	NM_01432	chr1:24175	NM_01432	Hs.409081
A_32_P198	-2.854958	-1.51347	2.854958	down	0.712064	0.85035	0.647871	-0.722926	-0.647871	-0.959327	YWHAZ	tyrosine 3-	FALSE	Homo sapi	7534	GO:000551	1.02E+08	chr8	1.02E+08	-	hs|8q22.3	NM_14569	chr8:10196	NM_14569	Hs.492407
A_22_P00C	-2.538415	-1.343928	2.538415	down	0.440424	0.59079	0.815238	-0.440424	-0.589451	-1.155458	lnc-DPCD-1	lnc-DPCD-1	FALSE	LNCipedia lincRNA (lnc-DPCD-1), li			1.04E+08	chr10	1.04E+08	+	hs|10q24.32		chr10:103570763-103570822		
A_24_P402	-3.029829	-1.599236	3.029829	down	0.733266	0.764308	0.724782	-0.854323	-0.724782	-0.996248	TGFB2	transformi	FALSE	Homo sapi	7042	GO:000551	2.19E+08	chr1	2.19E+08	+	hs|1q41	NM_00323	chr1:21861	NM_00323	Hs.133379
A_23_P21C	-2.48739	-1.314633	2.48739	down	0.6701	0.606242	0.567499	-0.731038	-0.567499	-0.801519	JAG1	jagged 1	FALSE	Homo sapi	182	GO:000551	10619120	chr20	10619061	-	hs|20p12.2	NM_00021	chr20:1061	NM_00021	Hs.626544
A_33_P331	-2.289263	-1.194883	2.289263	down	0.514821	0.452469	0.667043	-0.638715	-0.452469	-0.859134	NF1P2	neurofibro	FALSE	Homo sapi	440225		22136977	chr15	22133286	-	hs|15q11.2	NR_02850	chr15:2213	NR_02850	Hs.722451

A_23_P394	-5.827391	-2.54285	5.827391	down		1.296702	1.286732	1.237279	-1.237279	-1.29665	-1.273907	BST2	bone marr	FALSE	Homo sapi	684	GO:000551	17514113	chr19	17514054	-	hs|19p13.1	NM_00433	chr19:1751	NM_00433	Hs.118110
A_23_P303	-2.029855	-1.021377	2.029855	down		0.419851	0.4005	0.386529	-0.724659	-0.38653	-0.74606	P4HA2	prolyl 4-hy	FALSE	Homo sapi	8974	GO:001622	1.32E+08	chr5	1.32E+08	-	hs|5q31.1	NM_00419	chr5:13152	NM_00419	Hs.519568
A_33_P322	-2.242746	-1.165266	2.242746	down		0.434222	0.44909	0.382873	-0.866824	-0.382874	-0.979916	CD276	CD276 mol	FALSE	Homo sapi	80381	GO:000551	74006850	chr15	74006791	+	hs|15q24.1	NM_00102	chr15:7400	NM_00102	Hs.744915
A_24_P287	-2.783674	-1.47699	2.783674	down		0.786511	0.810503	0.660579	-0.729528	-0.660579	-0.783271	RSAD2	radical S-a	FALSE	Homo sapi	91543	GO:200055	7037259	chr2	7037200	+	hs|2p25.2	NM_08065	chr2:70372	NM_08065	Hs.17518
A_19_P008	-5.047222	-2.33549	5.047222	down		0.850025	0.943735	0.951019	-1.653747	-0.850025	-1.757918	LOC40095	uncharacte	FALSE	Homo sapi	400958		65129780	chr2	65129721	-	hs|2p14		chr2:65129	NR_03658	Hs.591565
A_23_P916	-3.805889	-1.928233	3.805889	down		0.993504	0.96169	0.928722	-0.994289	-0.928722	-0.977775	POM121L9	POM121 tr	FALSE	Homo sapi	29774		24661154	chr22	24661095	+	hs|22q11.2	NR_00371	chr22:2466	NR_00371	Hs.534980
A_22_P000	-2.37155	-1.245831	2.37155	down		0.480251	0.468139	0.449618	-0.449618	-1.411861	-0.478006	NEURL4	neuralized	FALSE	Homo sapi	84461	GO:000551	7220858	chr17	7220799	-	hs|17p13.1	NM_03244	chr17:7220	NM_03244	Hs.654794
A_24_P137	-2.149998	-1.104336	2.149998	down		0.3698	0.276386	0.205318	-0.481421	-0.205318	-1.774765	IFRD1	interferon-	FALSE	Homo sapi	3475	GO:000751	1.12E+08	chr7	1.12E+08	+	hs|7q31.1	NM_00100	chr7:11211	NM_00100	Hs.7879
A_23_P581	-3.107111	-1.635574	3.107111	down		0.861328	0.852777	0.664876	-0.865121	-0.664876	-0.997744	NPY2R	neuropepti	FALSE	Homo sapi	4887	GO:000551	1.56E+08	chr4	1.56E+08	+	hs|4q32.1	NM_00091	chr4:15613	NM_00091	Hs.37125
A_32_P105	-10.12067	-3.339233	10.12067	down		1.631859	1.800231	1.641585	-1.634304	-1.631859	-1.677862	ANXA8L1	annexin A8	FALSE	Homo sapi	728113	GO:000554	47763018	chr10	47762959	+	hs|10q11.2	NM_00109	chr10:4776	NM_00109	Hs.744068
A_24_P304	-3.716236	-1.893842	3.716236	down		1.035294	0.962619	0.669987	-1.295981	-0.669987	-1.047659	IFIT2	interferon-	FALSE	Homo sapi	3433	GO:003209	91066672	chr10	91066613	+	hs|10q23.3	NM_00154	chr10:9106	NM_00154	Hs.437609
A_33_P339	-2.4352	-1.28404	2.4352	down		0.636056	0.610284	0.470671	-0.823684	-0.470671	-0.840753	C17orf96	chromoson	FALSE	Homo sapi	1E+08	GO:004866	36828068	chr17	36828009	-	hs|17q12	NM_00113	chr17:3682	NM_00113	Hs.445574
A_23_P399	-3.726776	-1.897928	3.726776	down		0.801297	0.821951	0.835256	-1.269468	-0.801296	-1.164516	ACTG2	actin, gamr	FALSE	Homo sapi	72	GO:000693	74146759	chr2	74146700	+	hs|2p13.1	NM_00161	chr2:74146	NM_00161	Hs.516105
A_33_P324	-5.149599	-2.36446	5.149599	down		1.527276	0.98266	1.13133	-1.417007	-1.052447	-0.98266	REP15	RAB15 effe	FALSE	Homo sapi	387849	GO:000551	27850325	chr12	27850266	+	hs|12p11.2	NM_00102	chr12:2785	NM_00102	Hs.269836
A_33_P327	-2.095144	-1.067049	2.095144	down		0.363511	0.288602	0.693294	-0.288602	-0.550469	-1.01667	ATXN3	ataxin 3	FALSE	Homo sapi	4287	GO:000551	92529192	chr14	92529133	-	hs|14q32.1	NM_00499	chr14:9252	NM_00499	Hs.532632
A_33_P327	-2.3567	-1.236768	2.3567	down		0.523111	0.598905	0.489736	-0.752379	-0.856437	-0.489736	PGF	placental g	FALSE	Homo sapi	5228	GO:000551	75408599	chr14	75408540	-	hs|14q24.3	NM_00263	chr14:7540	NM_00263	Hs.252820
A_24_P296	-2.071508	-1.050682	2.071508	down		0.371941	0.415904	0.447821	-0.784781	-0.371942	-0.759656	MAP2K3	mitogen-ac	FALSE	Homo sapi	5606	GO:000551	21218523	chr17	21218464	+	hs|17p11.2	NM_14510	chr17:2121	NM_14510	Hs.514012
A_33_P323	-2.138824	-1.096818	2.138824	down		0.434703	0.425201	0.449537	-0.750159	-0.425201	-0.805652	DGKZ	diacylglyce	FALSE	Homo sapi	8525	GO:000485	46402097	chr11	46402038	+	hs|11p11.2	NM_00110	chr11:4640	NM_00110	Hs.502461
A_24_P415	-2.125496	-1.087799	2.125496	down		0.476241	0.47118	0.608523	-0.72602	-0.47118	-0.510255	SGK494	uncharacte	FALSE	Homo sapi	124923	GO:000467	26935213	chr17	26935154	-	hs|17q11.2	NM_00117	chr17:2693	NM_00117	Hs.729077
A_24_P333	-2.155246	-1.107853	2.155246	down		0.442498	0.488887	0.447991	-0.794552	-0.442498	-0.707132	ATP6V0A1	ATPase, H+	FALSE	Homo sapi	535	GO:000828	40674535	chr17	40674476	+	hs|17q21.2	NM_00517	chr17:4067	NM_00517	Hs.463074
A_33_P335	-2.826932	-1.499237	2.826932	down		0.759686	0.776871	0.636386	-0.779642	-0.90874	-0.636386	RASAL2	RAS protei	FALSE	Homo sapi	9462	GO:000551	1.78E+08	chr1	1.78E+08	+	hs|1q25.2	NM_17069	chr1:17844	NM_17069	Hs.496139
A_33_P366	-2.025617	-1.018361	2.025617	down		0.381266	0.456294	0.513317	-0.476898	-0.381266	-0.846041	GPR180	G protein-c	FALSE	Homo sapi	160897	GO:000718	95285319	chr13	95285260	+	hs|13q32.1	NM_18098	chr13:9528	NM_18098	Hs.439363
A_33_P331	-2.302553	-1.203234	2.302553	down		0.484114	0.572309	0.643739	-0.538137	-0.887289	-0.484114	HERC6	HECT and F	FALSE	Homo sapi	55008	GO:000573	89317293	chr4	89317234	+	hs|4q22.1	NM_00116	chr4:89317	NM_00116	Hs.529317
A_33_P331	-2.584244	-1.369742	2.584244	down		0.678679	0.684619	0.636804	-0.636804	-0.744464	-0.727855	AHR	aryl hydroc	FALSE	Homo sapi	196	GO:000551	17382822	chr7	17382763	+	hs|7p21.1	NM_00162	chr7:17382	NM_00162	Hs.171189
A_22_P000	-2.09022	-1.063655	2.09022	down		0.597859	0.465736	0.435245	-0.634263	-0.435245	-0.622618	PSMD5-AS	PSMD5 ant	FALSE	Homo sapi	253039		1.24E+08	chr9	1.24E+08	+	hs|9q33.2	NR_02440	chr9:12360	NR_02440	Hs.594170
A_33_P326	-2.397583	-1.261581	2.397583	down		0.52422	0.645314	0.600959	-0.679116	-0.52422	-0.810914	RHEBL1	Ras homolc	FALSE	Homo sapi	121268	GO:003192	49458528	chr12	49458469	-	hs|12q13.1	NM_00130	chr12:4945	NM_00130	Hs.159013
A_23_P161	-2.737579	-1.452901	2.737579	down		0.565478	0.714229	0.595012	-0.941545	-0.565478	-0.97696	ANKRD1	ankyrin rep	FALSE	Homo sapi	27063	GO:000551	92672621	chr10	92672562	-	hs|10q23.3	NM_01439	chr10:9267	NM_01439	Hs.448589
A_32_P593	-2.688976	-1.427057	2.688976	down		0.475572	0.49688	0.524333	-1.144472	-0.475572	-1.164343	HIVEP3	human imr	FALSE	Homo sapi	59269	GO:000573	41972662	chr1	41972603	-	hs|1p34.2	NM_02450	chr1:41972	NM_02450	Hs.403972
A_23_P571	-2.790644	-1.480598	2.790644	down		0.590224	0.540357	0.552929	-1.131296	-0.540357	-1.086632	SLC2A1	solute carri	FALSE	Homo sapi	6513	GO:000551	43391933	chr1	43391874	-	hs|1p34.2	NM_00651	chr1:43391	NM_00651	Hs.473721
A_33_P331	-2.115291	-1.080856	2.115291	down		0.522924	0.515737	0.463021	-0.463021	-0.776328	-0.501538	OLAH	oleoyl-ACP	FALSE	oleoyl-ACP	55301	GO:001629	15104036	chr10	15103977	+	hs|10p13		chr10:15103977-15104036		
A_21_P001	-2.106715	-1.074995	2.106715	down		0.353571	0.707775	0.556675	-0.353571	-0.48544	-0.767954			FALSE	double homeobox A pseudogene 8			16186208	chr22	16186149	-	hs|22q11.1		chr22:16186208-16186149		
A_23_P380	-2.779376	-1.474761	2.779376	down		0.875343	0.748962	0.62062	-0.62062	-0.782655	-0.776082	APOL4	apolipopro	FALSE	Homo sapi	80832	GO:000662	36585942	chr22	36585883	-	hs|22q12.3	NM_03064	chr22:3658	NM_03064	Hs.115099
A_33_P339	-2.476525	-1.308317	2.476525	down		0.551846	0.649322	0.932253	-0.551846	-0.591299	-0.648385			FALSE				1.29E+08	chr5	1.29E+08	+	hs|5q23.3		chr5:129477947-129478006		
A_33_P329	-2.753344	-1.461185	2.753344	down		0.740522	0.638401	0.563113	-0.563113	-1.060648	-0.817756	IMPA2	inositol(my	FALSE	Homo sapi	3613	GO:005283	11999185	chr18	11999126	+	hs|18p11.2	NM_01421	chr18:1199	NM_01421	Hs.743311
A_23_P176	-3.521048	-1.816005	3.521048	down		0.796794	0.790689	0.813579	-1.165652	-0.790689	-1.090612	MX1	MX dynam	FALSE	Homo sapi	4599	GO:000551	42831073	chr21	42831014	+	hs|21q22.3	NM_00246	chr21:4283	NM_00246	Hs.517307
A_33_P340	-3.124332	-1.643548	3.124332	down		0.842232	0.70313	0.755329	-1.030447	-0.896375	-0.70313	SLC6A9	solute carri	FALSE	Homo sapi	6536	GO:001537	44462221	chr1	44462162	-	hs|1p34.1	NM_20164	chr1:44462	NM_20164	Hs.442590
A_23_P310	-2.311911	-1.209086	2.311911	down		0.646328	0.523386	0.535111	-0.695848	-0.523386	-0.703199	KIAA1217	KIAA1217	FALSE	Homo sapi	56243	GO:000367	24836518	chr10	24836459	+	hs|10p12.1	NM_01959	chr10:2483	NM_01959	Hs.445885
A_23_P384	-2.319176	-1.213612	2.319176	down		0.678721	0.726312	0.462929	-0.722345	-0.587601	-0.462929	DZIP1L	DAZ intera	FALSE	Homo sapi	199221	GO:004238	1.38E+08	chr3	1.38E+08	-	hs|3q22.3	NM_17354	chr3:13778	NM_17354	Hs.351403
A_23_P383	-2.158718	-1.110175	2.158718	down		0.581703	0.608492	0.508877	-0.560457	-0.508877	-0.562119	CHST15	carbohydra	FALSE	Homo sapi	51363	GO:003020	1.26E+08	chr10	1.26E+08	-	hs|10q26.1	NM_01589	chr10:1257	NM_01589	Hs.287537
A_23_P776	-2.107555	-1.07557	2.107555	down		0.439032	0.464494	0.386236	-0.834704	-0.714008	-0.386236	ZNF821	zinc finger	FALSE	Homo sapi	55565	GO:000635	71893808	chr16	71893749	-	hs|16q22.2	NM_01753	chr16:7189	NM_01753	Hs.744055
A_33_P326	-2.291357	-1.196202	2.291357	down		0.451462	0.622377	0.476733	-0.822363	-0.451462	-0.764211	HAUS5	HAUS augn	FALSE	Homo sapi	23354	GO:005129	36114511	chr19	36114452	+	hs|19q13.1	NM_01530	chr19:3611	NM_01530	Hs.7426
A_33_P334	-7.336971	-2.875185	7.336971	down		1.396485	1.327513	1.359294	-1.604005	-1.327513	-1.610743	HES4	hes family	FALSE	Homo sapi	57801	GO:000635	934404	chr1	934345	-	hs|1p36.33	NM_02117	chr1:93440	NM_02117	Hs.154029
A_23_P652	-4.086388	-2.030826	4.086388	down		0.939333	0.977547	0.961256	-0.939333	-1.334255	-0.940755	COL4A1	collagen, ty	FALSE	Homo sapi	1282	GO:000551	1.11E+08	chr13	1.11E+08	-	hs|13q34	NM_00184	chr13:1108	NM_00184	Hs.17441
A_23_P151	-2.343989	-1.228966	2.343989	down		0.542713	0.404931	0.603155	-0.404931	-0.886319	-0.844848	PTGER2	prostaglanc	FALSE	Homo sapi	5732	GO:000718	52794489	chr14	52794430	+	hs|14q22.1	NM_00095	chr14:5279	NM_00095	Hs.2090
A_22_P000	-2.35496	-1.235703	2.35496	down		0.509035	0.747709	0.794423	-0.509035	-0.515168	-0.631737	lnc-GOLGA	lnc-GOLGA	FALSE	Homo sapiens, clone IMAGE:5221			29969614	chr15	29969555	+	hs|15q13.1		chr15:29969555-29969		Hs.525720
A_23_P884	-2.598871	-1.377885	2.598871	down		0.521395	0.843567	0.596669	-0.521395	-0.784311	-0.866317	TGFB3	transformi	FALSE	Homo sapi	7043	GO:000551	76424963	chr14	76424904	-	hs|14q24.3	NM_00323	chr14:7642	NM_00323	Hs.592317
A_33_P321	-2.4579	-1.297426	2.4579	down		0.564826	0.571165	0.702518	-0.774949	-0.564826	-0.713995			FALSE	ankyrin repeat domain 20 family, r			14227698	chr18	14227639	+	hs|18p11.21		chr18:14227639-14227		Hs.721707
A_33_P334	-2.214019	-1.146668	2.214019	down		0.40726	0.474156	0.455405	-0.942234	-0.40726	-0.753688	CECR2	cat eye syn	FALSE	PREDICTED	27443	GO:000551	18019203	chr22	18019144	+	hs|22q11.2	XM_00672	chr22:1801	XM_00672	Hs.658723
A_33_P323	-2.058323	-1.04147	2.058323	down		0.55739	0.482438	0.539217	-0.508429	-0.554498	-0.482438	CTPS1	CTP syntha	FALSE	Homo sapi	1503	GO:000624	41475925	chr1	41475866	+	hs|1p34.2	NM_00190	chr1:41475	NM_00190	Hs.473087
A_24_P418	-2.445113	-1.289901	2.445113	down		0.518193	0.603322	0.656474	-0.730771	-0.518193	-0.84275	MACF1	microtubul	FALSE	Homo sapi	23499	GO:000551	39750014	chr1	39749808	+	hs|1p34.3	NM_01209	chr1:39749	NM_01209	Hs.472475
A_24_P728	-2.30786	-1.206556	2.30786	down		0.507048	0.47074	0.625326	-0.47074	-0.79728	-0.748533	XLOC_l2_011874		FALSE	BROAD Institute lincRNA (XLOC_l2			1.77E+08	chr5	1.77E+08	+	hs|5q35.3		chr5:177482509-177482568		
A_32_P192	-2.137783	-1.096115	2.137783	down		0.478452	0.443757	0.396479	-0.396479	-0.399833	-1.173347	ENPP1	ectonucleo	FALSE	Homo sapi	5167	GO:000551	1.32E+08	chr6	1.32E+08	+	hs|6q23.2	NM_00620	chr6:13221	NM_00620	Hs.527295
A_33_P325	-2.253108	-1.171916	2.253108	down		0.6192	0.575271	0.502739	-0.544764	-0.502739	-0.771037	PLEKHH3	pleckstrin h	FALSE	Homo sapi	79990	GO:000716	40820030	chr17	40819971	-	hs|17q21.2	NM_02492	chr17:4082	NM_02492	Hs.632251
A_23_P307	-4.751508	-2.248385	4.751508	down		1.041463	1.030641	1.130184	-1.254989	-1.030641	-1.257239	ACAN	aggrecan	FALSE	Homo sapi	176	GO:000551	89417830	chr15	89417771	+	hs|15q26.1	NM_01322	chr15:8941	NM_01322	Hs.2159
A_32_P656	-2.225997	-1.154451	2.225997	down		0.372222	0.408671	0.356827	-0.968534	-0.356828	-1.000272	PRL	prolactin	FALSE	Homo sapi	5617	GO:000551	22287617	chr6	22287558	-	hs|6p22.3	NM_00094	chr6:22287	NM_00094	Hs.1905
A_33_P361	-2.607773	-1.382818	2.607773	down		0.540423	0.642522	0.503901	-0.891729	-0.503901	-1.065979	PMAIP1	phorbol-12	FALSE	Homo sapi	5366	GO:000551	57570750	chr18	57570691	+	hs|18q21.3	NM_02112	chr18:5757	NM_02112	Hs.96
A_33_P337	-2.698037	-1.43191	2.698037	down		0.676862	0.660828	0.788288	-0.660828	-0.74482	-0.764104	DRD4	dopamine r	FALSE	Homo sapi	1815	GO:000551	640703	chr11	640644	+	hs|11p15.5	NM_00079	chr11:6406	NM_00079	Hs.99922
A_24_P331	-2.208525	-1.143083	2.208525	down		0.467448	0.483922	0.466242	-0.601308	-0.466242	-0.944088	KRT80	keratin 80,	FALSE	Homo sapi	144501	GO:003005	52563544	chr12	52563485	-	hs|12q13.1	NM_18250	chr12:5256	NM_18250	Hs.140978

A_22_P000	-2.081965	-1.057946	2.081965	down	0.375201	0.413704	0.61018	-0.803279	-0.596272	-0.375201	lnc-PITPNC	lnc-PITPNC	FALSE	LNCipedia lincRNA (lnc-PITPNC1-1)			65241902	chr17	65241843	+	hs|17q24.2		chr17:65241843-65241902		
A_24_P879	-2.301931	-1.202844	2.301931	down	0.601751	0.550934	0.614605	-0.656972	-0.550934	-0.633338	MAP1B	microtubul	FALSE	Homo sapi	4131	GO:000551	71504815	chr5	71504756	+	hs|5q13.2	NM_00590	chr5:71504	NM_00590	Hs.335079
A_32_P128	-2.35564	-1.236119	2.35564	down	0.457469	0.603449	0.564568	-0.824675	-0.457469	-0.800728	USP53	ubiquitin sp	FALSE	Homo sapi	54532	GO:000815	1.2E+08	chr4	1.2E+08	+	hs|4q26	NM_01905	chr4:12021	NM_01905	Hs.431081
A_23_P105	-2.178471	-1.123316	2.178471	down	0.754788	0.435275	0.563657	-0.435275	-0.655376	-0.525577	EPSTI1	epithelial s	FALSE	Homo sapi	94240		43528115	chr13	43500538	-	hs|13q14.1	NM_03325	chr13:4352	NM_03325	Hs.546467
A_33_P339	-2.311113	-1.208588	2.311113	down	0.618441	0.447947	0.446017	-0.446017	-1.001363	-0.665978	HLA-B	major histc	FALSE	major histc	3106	GO:0002474|GO:0005886|GO:0005783|GO:0019221|GO:0051087|G						XR_246962		XR_246962	Hs.77961
A_23_P962	-2.238809	-1.162732	2.238809	down	0.433213	0.39406	0.486994	-0.768944	-1.010924	-0.39406	REEP4	receptor ac	FALSE	Homo sapi	80346	GO:000801	21995837	chr8	21995778	-	hs|8p21.3	NM_02523	chr8:21995	NM_02523	Hs.289063
A_21_P000	-2.998326	-1.584157	2.998326	down	0.79643	0.66412	0.72873	-0.914239	-0.66412	-0.984833	PARP9	poly (ADP-r	FALSE	Homo sapi	83666	GO:000551	1.22E+08	chr3	1.22E+08	-	hs|3q21.1	NM_00114	chr3:12225	NM_00114	Hs.518200
A_23_P205	-2.790105	-1.480319	2.790105	down	0.59686	0.695068	0.645734	-0.59686	-1.087926	-0.81851	SLC46A3	solute carri	FALSE	Homo sapi	283537	GO:000815	29278193	chr13	29278134	-	hs|13q12.3	NM_18178	chr13:2927	NM_18178	Hs.117167
A_23_P205	-2.510087	-1.327738	2.510087	down	0.56435	0.626955	0.610493	-0.833371	-0.564349	-0.783694	COL4A2	collagen, ty	FALSE	Homo sapi	1284	GO:000551	1.11E+08	chr13	1.11E+08	+	hs|13q34	NM_00184	chr13:1111	NM_00184	Hs.508716
A_32_P147	-4.316349	-2.109812	4.316349	down	0.967549	1.077011	0.943218	-1.101009	-0.943218	-1.297428	SLC8A1	solute carri	FALSE	Homo sapi	6546	GO:006040	40339484	chr2	40339425	-	hs|2p22.1	NM_02109	chr2:40339	NM_02109	Hs.468274
A_23_P144	-3.038421	-1.603322	3.038421	down	0.711058	0.7204	0.738263	-0.785933	-1.143254	-0.711058	COL7A1	collagen, ty	FALSE	Homo sapi	1294	GO:000551	48602588	chr3	48602401	-	hs|3p21.31	NM_00009	chr3:48602	NM_00009	Hs.476218
A_23_P369	-2.111093	-1.07799	2.111093	down	0.401548	0.648039	0.628646	-0.401548	-0.58938	-0.56481	ZMYND8	zinc finger,	FALSE	zinc finger,	23613	GO:000551	45866354	chr20	45866295	-	hs|20q13.1	AL137703	chr20:45866354-45866		Hs.446240
A_33_P378	-2.032683	-1.023386	2.032683	down	0.669383	0.752917	0.334226	-0.582224	-0.397181	-0.334226	C8orf60	chromoson	FALSE	Homo sapi	619426		1.42E+08	chr8	1.42E+08	+	hs|8q24.3	AK022255	chr8:142182820-142182879		
A_23_P445	-2.090545	-1.063879	2.090545	down	0.750238	0.396044	0.521444	-0.462883	-0.664983	-0.396044	KLF11	Kruppel-lik	FALSE	Homo sapi	8462	GO:004306	10194705	chr2	10194646	+	hs|2p25.1	NM_00359	chr2:10194	NM_00359	Hs.12229
A_23_P118	-2.055543	-1.03952	2.055543	down	0.474552	0.545136	0.477744	-0.528088	-0.618487	-0.474552	RGS11	regulator o	FALSE	Homo sapi	8786	GO:003168	318383	chr16	318324	-	hs|16p13.3	NM_00383	chr16:3183	NM_00383	Hs.65756
A_23_P120	-2.252278	-1.171385	2.252278	down	0.446774	0.440034	0.398294	-0.900163	-0.398294	-0.930595	ATF4	activating t	FALSE	Homo sapi	468	GO:000551	39918636	chr22	39918577	+	hs|22q13.1	NM_00167	chr22:3991	NM_00167	Hs.496487
A_21_P001	-2.426075	-1.278624	2.426075	down	0.59652	0.602546	0.617992	-0.59652	-0.799654	-0.622639	SYNGR2	synaptogyr	FALSE	Homo sapi	9144	GO:003159	76168272	chr17	76168213	+	hs|17q25.3	NM_00471	chr17:7616	NM_00471	Hs.464210
A_23_P408	-2.834328	-1.503007	2.834328	down	0.704034	0.695941	0.675675	-0.871938	-0.675675	-0.885756	CHST2	carbohydra	FALSE	Homo sapi	9435	GO:000580	1.43E+08	chr3	1.43E+08	+	hs|3q24	NM_00426	chr3:14284	NM_00426	Hs.8786
A_21_P000	-2.845196	-1.508528	2.845196	down	0.940004	0.867187	0.574862	-0.770503	-0.798165	-0.574862	SNORD114	small nucle	FALSE	Homo sapi	767589		1.01E+08	chr14	1.01E+08	+	hs|14q32.3	NR_00320	chr14:1014	NR_003204	
A_23_P401	-5.248901	-2.392016	5.248901	down	1.114499	1.167572	1.05878	-1.273134	-1.05878	-1.503283	EDIL3	EGF-like re	FALSE	Homo sapi	10085	GO:000517	83238522	chr5	83238463	-	hs|5q14.3	NM_00571	chr5:83238	NM_00571	Hs.482730
A_23_P819	-3.094754	-1.629825	3.094754	down	0.782633	0.801847	0.803679	-0.86693	-0.782634	-0.851751	ISG15	ISG15 ubiq	FALSE	Homo sapi	9636	GO:000551	949626	chr1	949567	+	hs|1p36.33	NM_00510	chr1:94956	NM_00510	Hs.458485
A_24_P416	-2.191937	-1.132206	2.191937	down	0.545648	0.518318	0.551027	-0.541138	-0.722169	-0.518318	APOL3	apolipopro	FALSE	Homo sapi	80833	GO:000716	36536995	chr22	36536936	-	hs|22q12.3	NM_14564	chr22:3653	NM_14564	Hs.474737
A_24_P333	-3.671178	-1.876243	3.671178	down	0.812144	1.124669	1.117738	-0.894434	-0.812144	-0.867601	SGIP1	SH3-domai	FALSE	Homo sapi	84251	GO:000551	67209764	chr1	67209705	+	hs|1p31.3	NM_03229	chr1:67209	NM_03229	Hs.132121
A_33_P336	-2.57496	-1.36455	2.57496	down	0.634028	0.935287	0.604307	-0.608549	-0.604307	-0.707173	LBH	limb bud ar	FALSE	Homo sapi	81606	GO:001046	30480625	chr2	30480566	+	hs|2p23.1	NM_03091	chr2:30480	NM_03091	Hs.567598
A_21_P000	-6.708038	-2.745891	6.708038	down	1.180311	1.097143	1.251357	-1.725187	-1.097143	-1.886533	lnc-RPP30-	lnc-RPP30-	FALSE	LNCipedia lincRNA (lnc-RPP30-2), l			92750040	chr10	92749981	+	hs|10q23.31		chr10:92749981-92750040		
A_33_P321	-2.0144	-1.01035	2.0144	down	0.300271	0.338639	0.313938	-0.939055	-0.300271	-0.838877	TAP2	transporter	FALSE	Homo sapi	6891	GO:000551	32793281	chr6	32793222	-	hs|6p21.32	NM_00129	chr6:32793	NM_00129	Hs.502
A_33_P331	-2.376982	-1.249131	2.376982	down	0.514494	0.576905	0.489082	-0.936485	-0.489082	-0.741344			FALSE				6830547	chr17	6830488	-	hs|17p13.1		chr17:006830547-006830488		
A_33_P331	-2.003651	-1.002631	2.003651	down	0.447969	0.36672	0.429805	-0.36672	-1.007775	-0.388904	PRKAA1	protein kin	FALSE	Homo sapi	5562	GO:000551	40769578	chr5	40769519	-	hs|5p13.1	NM_20690	chr5:40769	NM_20690	Hs.43322
A_33_P330	-2.133454	-1.093191	2.133454	down	0.45929	0.541978	0.351971	-0.351971	-1.059239	-0.515124	GGA1	golgi-assoc	FALSE	Homo sapi	26088	GO:000551	38028688	chr22	38028629	+	hs|22q13.1	NM_00117	chr22:3802	NM_00117	Hs.499158
A_24_P393	-2.118341	-1.082935	2.118341	down	0.667385	0.478583	0.527168	-0.578285	-0.478583	-0.518801	DNAJB4	DnaJ (Hsp4	FALSE	Homo sapi	11080	GO:000698	78482092	chr1	78482033	+	hs|1p31.1	NM_00703	chr1:78482	NM_00703	Hs.13852
A_33_P332	-2.62442	-1.391999	2.62442	down	0.664381	0.590481	0.550674	-0.915131	-0.550674	-0.904654			FALSE	ankyrin repeat domain 20 family, r			14185368	chr18	14185309	+	hs|18p11.2	BC022023	chr18:14185309-14185		Hs.740188
A_32_P766	-3.938683	-1.977713	3.938683	down	1.013482	1.121054	0.897993	-0.926248	-0.897993	-1.07637			FALSE	keratin 16 pseudogene 6 [Source:H			16722625	chr17	16722566	-	hs|17p11.2		chr17:16722625-16722566		
A_22_P000	-2.416218	-1.272751	2.416218	down	0.456054	0.398542	0.734597	-0.398542	-0.554765	-1.27575	lnc-FAM49	lnc-FAM49	FALSE	LNCipedia lincRNA (lnc-FAM49B-1)			1.31E+08	chr8	1.31E+08	-	hs|8q24.21		chr8:131155765-131155706		
A_22_P000	-2.222447	-1.152149	2.222447	down	0.378591	0.562879	0.350513	-0.740687	-0.350513	-1.073266	LOC10192	uncharacte	FALSE	Homo sapi	1.02E+08		42158855	chr2	42158796	-	hs|2p21	NR_11026	chr2:42158	NR_110262	
A_23_P184	-2.094152	-1.066366	2.094152	down	0.403872	0.386998	0.399745	-0.768463	-0.386998	-0.853023	PPARGC1A	peroxisome	FALSE	Homo sapi	10891	GO:000551	23793996	chr4	23793937	-	hs|4p15.2	NM_01326	chr4:23793	NM_01326	Hs.527078
A_32_P116	-2.386428	-1.254853	2.386428	down	0.532443	0.566273	0.523985	-0.857539	-0.523985	-0.760332	ZNF469	zinc finger	FALSE	Homo sapi	84627	GO:000635	88507146	chr16	88507087	+	hs|16q24.2	NM_00112	chr16:8850	NM_00112	Hs.54925
A_22_P000	-4.495793	-2.168576	4.495793	down	1.017736	1.080118	1.082818	-1.149974	-1.017736	-1.157346	POM121L1	POM121 tr	FALSE	Homo sapi	25812		22974050	chr22	22974028	-	hs|22q11.2	NR_02459	chr22:2297	NR_02459	Hs.367764
A_33_P331	-2.118488	-1.083035	2.118488	down	0.560093	0.538639	0.407345	-0.407345	-0.489393	-0.84629	CCDC14	coiled-coil	FALSE	Homo sapi	64770	GO:000581	1.24E+08	chr3	1.24E+08	-	hs|3q21.1	NM_02275	chr3:12363	NM_02275	Hs.645028
A_23_P387	-3.866291	-1.95095	3.866291	down	0.945395	1.051705	0.944912	-1.01806	-0.947866	-0.944912	CDH2	cadherin 2,	FALSE	Homo sapi	1000	GO:000551	25532140	chr18	25532081	-	hs|18q12.1	NM_00179	chr18:2553	NM_00179	Hs.464829
A_33_P321	-3.378369	-1.756327	3.378369	down	0.787708	0.746607	0.787963	-1.115706	-1.08439	-0.746607	IL18R1	interleukin	FALSE	Homo sapi	8809	GO:000551	1.03E+08	chr2	1.03E+08	+	hs|2q12.1	NM_00385	chr2:10298	NM_00385	Hs.469521
A_33_P334	-2.654243	-1.4083	2.654243	down	0.588065	0.746221	0.64032	-0.76698	-0.588065	-0.895251	CORO7	coronin 7	FALSE	Homo sapi	79585	GO:000551	4404604	chr16	4404545	-	hs|16p13.3	NM_02453	chr16:4404	NM_02453	Hs.437957
A_21_P000	-2.020206	-1.014502	2.020206	down	0.718589	0.491821	0.340538	-0.401074	-0.340538	-0.750947	HERC2P7	hect domai	FALSE	Homo sapi	1E+08		23393879	chr15	23393820	+	hs|15q11.2		chr15:2339	NR_03647	Hs.146211
A_21_P000	-3.519972	-1.815564	3.519972	down	0.77156	0.930698	0.831067	-1.120418	-0.77156	-1.021388	lnc-RP11-5	lnc-RP11-5	FALSE	BC011509 Cox15 protein {Mus mu			82393814	chr15	82393755	+	hs|15q25.2		chr15:82393755-82393814		
A_33_P329	-2.516964	-1.331685	2.516964	down	0.643987	0.62628	0.646066	-0.743246	-0.62628	-0.709195	ANGPTL4	angiopoieti	FALSE	Homo sapi	51129	GO:000485	8439256	chr19	8439197	+	hs|19p13.2	NM_13931	chr19:8439	NM_13931	Hs.9613
A_21_P000	-2.530981	-1.339696	2.530981	down	0.499308	0.763311	0.515161	-1.142548	-0.499308	-0.599453			FALSE				76698858	chr12	76698799	+	hs|12q21.2		chr12:76698799-76698858		
A_33_P334	-2.296051	-1.199155	2.296051	down	0.560012	0.486748	0.431726	-0.776564	-0.431726	-0.910689			FALSE	activating transcription factor 4 ps			1.54E+08	chrX	1.54E+08	+	hs|Xq28		chrX:153810202-153810261		
A_22_P000	-2.160803	-1.111568	2.160803	down	0.710986	0.435618	0.737198	-0.454331	-0.435618	-0.560953	LOC10192	uncharacte	FALSE	Homo sapi	1.02E+08		1.34E+08	chr4	1.34E+08	-	hs|4q28.3	NR_12588	chr4:13401	NR_12588	Hs.649960
A_21_P000	-2.230366	-1.157281	2.230366	down	0.683069	0.719356	0.374121	-0.68213	-0.639045	-0.374121	SNORD114	small nucle	FALSE	Homo sapi	767591		1.01E+08	chr14	1.01E+08	+	hs|14q32.3	NR_00320	chr14:1014	NR_003206	
A_23_P920	-2.301722	-1.202714	2.301722	down	0.421452	0.782857	0.562884	-0.421452	-0.978618	-0.440878	CELSR3	cadherin, E	FALSE	Homo sapi	1951	GO:003288	48674303	chr3	48674244	-	hs|3p21.31	NM_00140	chr3:48674	NM_00140	Hs.631926
A_23_P230	-2.6476	-1.404685	2.6476	down	0.630001	0.623365	0.641309	-0.866131	-0.623365	-0.829884	IFI44	interferon-	FALSE	Homo sapi	10561	GO:000573	79128538	chr1	79128479	+	hs|1p31.1	NM_00641	chr1:79128	NM_00641	Hs.82316
A_23_P530	-2.103164	-1.072561	2.103164	down	0.476997	0.507082	0.532301	-0.617169	-0.476996	-0.607139	OSBPL5	oxysterol b	FALSE	Homo sapi	114879	GO:001602	3108697	chr11	3108638	-	hs|11p15.4	NM_02089	chr11:3108	NM_02089	Hs.436166
A_22_P000	-2.027276	-1.019543	2.027276	down	0.519907	0.472088	0.556224	-0.53775	-0.472088	-0.50057	PDGFRB	platelet-de	FALSE	Homo sapi	5159	GO:000551	1.5E+08	chr5	1.5E+08	-	hs|5q32	NM_00260	chr5:14953	NM_00260	Hs.509067
A_33_P321	-4.332532	-2.115211	4.332532	down	1.051578	1.003557	0.949342	-1.156038	-0.949342	-1.235775	MAP2	microtubul	FALSE	Homo sapi	4133	GO:000551	2.11E+08	chr2	2.11E+08	+	hs|2q34	NM_00237	chr2:21059	NM_00237	Hs.368281
A_23_P119	-6.500229	-2.700491	6.500229	down	1.470339	1.247765	0.971643	-1.710584	-0.971643	-1.729498	PPP6R1	protein pho	FALSE	Homo sapi	22870	GO:000551	55741657	chr19	55741598	-	hs|19q13.4	NM_01493	chr19:5574	NM_01493	Hs.515610
A_24_P135	-2.223548	-1.152864	2.223548	down	0.601509	0.570093	0.544336	-0.544336	-0.62112	-0.577197	PTPLB	protein tyr	FALSE	protein tyr	201562	GO:000551	1.23E+08	chr3	1.23E+08	-	hs|3q21.1	AL832532	chr3:123210797-12321		Hs.705480
A_33_P325	-3.324266	-1.733036	3.324266	down	0.74643	0.790972	0.829998	-1.081839	-0.74643	-1.003438	TFPI	tissue facto	FALSE	tissue facto	7035	GO:001095	1.88E+08	chr2	1.88E+08	-	hs|2q32.1	AB209866	chr2:188349577-188349518		
A_33_P326	-3.037796	-1.603025	3.037796	down	0.835257	0.654695	0.732155	-0.654695	-1.167266	-0.765008	POM121L1	POM121 tr	FALSE	Homo sapi	25812		22975037	chr22	22974978	-	hs|22q11.22		chr22:2297	NR_02459	Hs.367764
A_33_P337	-3.097186	-1.630958	3.097186	down	0.74331	0.841594	0.807404	-0.906029	-0.74331	-0.851227	LMNTD2	lamin tail d	FALSE	Homo sapi	256329	GO:004802	554917	chr11	554858	-	hs|11p15.5	NM_17357	chr11:5549	NM_17357	Hs.669395

A_23_P691	-2.16912	-1.11711	2.16912	down	0.467089	0.474612	0.447824	-0.802914	-0.447824	-0.711067	PLSCR1	phospholip	FALSE	Homo sapi	5359	GO:200037	1.46E+08	chr3	1.46E+08	-	hs|3q24	NM_02110	chr3:14623	NM_02110	Hs.130759
A_33_P381	-2.546274	-1.348388	2.546274	down	0.619945	0.725163	0.569899	-0.680259	-0.88	-0.569899			FALSE	Homo sapiens BPA-1 mRNA for bra			65765425	chr1	65765366	+	hs|1p31.3	AB088847	chr1:65765366-65765425		
A_21_P000	-3.551482	-1.828421	3.551482	down	0.896922	0.868758	0.819935	-0.975668	-0.819935	-1.104046	SNORD114	small nucle	FALSE	Homo sapi	767593		1.01E+08	chr14	1.01E+08	+	hs|14q32.3	NR_003208	chr14:1014	NR_003208	
A_24_P175	-2.111811	-1.078481	2.111811	down	0.439283	0.446435	0.453776	-0.675448	-0.439283	-0.781216	MARS	methionyl-	FALSE	Homo sapi	4141	GO:000573	57910432	chr12	57910373	+	hs|12q13.3	NM_00499	chr12:5791	NM_00499	Hs.632707
A_24_P231	-4.900963	-2.293065	4.900963	down	1.022726	1.052239	1.007286	-1.393803	-1.007286	-1.395857	LEPR	leptin recep	FALSE	Homo sapi	3953	GO:004572	66100708	chr1	66100649	+	hs|1p31.3	NM_00100	chr1:66100	NM_00100	Hs.723178
A_24_P381	-2.544656	-1.347471	2.544656	down	0.868801	0.934812	0.466768	-0.729588	-0.466768	-0.575675	PACSIN3	protein kin	FALSE	Homo sapi	29763	GO:000551	47200985	chr11	47200795	-	hs|11p11.2	NM_01622	chr11:4720	NM_01622	Hs.334639
A_23_P208	-2.050142	-1.035724	2.050142	down	0.391507	0.639792	0.492393	-0.391507	-0.78242	-0.409553	TLE6	transducin-	FALSE	Homo sapi	79816	GO:004323	2994022	chr19	2993527	+	hs|19p13.3	NM_02476	chr19:2993	NM_02476	Hs.334507
A_23_P148	-2.098289	-1.069213	2.098289	down	0.426479	0.40868	0.432801	-0.779871	-0.40868	-0.751127	PTGER4	prostaglan	FALSE	Homo sapi	5734	GO:000551	40693622	chr5	40693563	+	hs|5p13.1	NM_00095	chr5:40693	NM_00095	Hs.199248
A_23_P334	-2.10937	-1.076812	2.10937	down	0.469489	0.51537	0.476299	-0.697398	-0.469489	-0.602391	IGF2R	insulin-like	FALSE	Homo sapi	3482	GO:005121	1.61E+08	chr6	1.61E+08	+	hs|6q25.3	NM_00087	chr6:16052	NM_00087	Hs.487062
A_23_P347	-2.031658	-1.022658	2.031658	down	0.461178	0.47855	0.560607	-0.542012	-0.461178	-0.564446	KIF2C	kinesin fam	FALSE	Homo sapi	11004	GO:000551	45233125	chr1	45233066	+	hs|1p34.1	NM_00684	chr1:45233	NM_00684	Hs.720061
A_33_P327	-4.580609	-2.19554	4.580609	down	0.992322	1.000517	1.00206	-1.295838	-0.992321	-1.303561	FMO2	flavin conta	FALSE	Homo sapi	2327	GO:005066	1.71E+08	chr1	1.71E+08	+	hs|1q24.3	NM_00146	chr1:17117	NM_00146	Hs.144912
A_32_P960	-2.207749	-1.142576	2.207749	down	0.406109	0.542616	0.428993	-0.677966	-0.406109	-0.965935	MEX3A	mex-3 RNA	FALSE	Homo sapi	92312	GO:000093	1.56E+08	chr1	1.56E+08	-	hs|1q22	NM_00109	chr1:15604	NM_00109	Hs.591496
A_23_P428	-2.125927	-1.088092	2.125927	down	0.478052	0.557681	0.477277	-0.621066	-0.477277	-0.652923	TTC21A	tetratricop	FALSE	Homo sapi	199223		39180368	chr3	39180309	+	hs|3p22.2	NM_14575	chr3:39180	NM_14575	Hs.443935
A_23_P310	-2.546227	-1.348361	2.546227	down	0.489854	0.553942	0.499925	-0.766258	-0.489854	-1.245249	MDGA1	MAM doma	FALSE	Homo sapi	266727	GO:000367	37600354	chr6	37600295	-	hs|6p21.2	NM_15348	chr6:37600	NM_15348	Hs.437993
A_33_P328	-2.126645	-1.088579	2.126645	down	0.505871	0.422919	0.61837	-0.422919	-0.641406	-0.654252	TLN1	talin 1	FALSE	talin 1 [Sou	7094	GO:000551	35723457	chr9	35723398	-	hs|9p13.3	BC020881	chr9:35723457-357233		Hs.471014
A_23_P435	-2.062223	-1.0442	2.062223	down	0.333625	0.428007	0.411586	-0.829934	-0.333625	-0.795823	GPC4	glypican 4	FALSE	Homo sapi	2239	GO:000588	1.32E+08	chrX	1.32E+08	-	hs|Xq26.2	NM_00144	chrX:13243	NM_00144	Hs.58367
A_22_P000	-2.424389	-1.277621	2.424389	down	0.746545	0.715258	0.385477	-0.385477	-0.851079	-0.749028			FALSE	Homo sapiens mRNA; cDNA DKFZp			3905460	chr6	3905401	-	hs|6p25.2	BX647262	chr6:3905460-3905401		Hs.407992
A_33_P325	-2.086503	-1.061087	2.086503	down	0.529422	0.50213	0.527941	-0.50213	-0.580146	-0.541493	ROCK2	Rho-associ	FALSE	Homo sapi	9475	GO:000551	11323535	chr2	11323476	-	hs|2p25.1	NM_00485	chr2:11323	NM_00485	Hs.681743
A_33_P335	-2.09391	-1.0662	2.09391	down	0.517916	0.497256	0.444452	-0.530704	-0.444452	-0.76382	EMB	embigin	FALSE	Homo sapi	133418	GO:003005	49694981	chr5	49694922	-	hs|5q11.1	NM_19844	chr5:49694	NM_19844	Hs.561411
A_23_P800	-6.017063	-2.589059	6.017063	down	1.243591	1.05042	1.180995	-1.430832	-1.05042	-1.810921	FER1L4	fer-1-like fa	FALSE	Homo sapi	80307	GO:001602	34146579	chr20	34146520	-	hs|20q11.2	NR_119376	chr20:3414	NR_119376	Hs.72222
A_33_P331	-2.845431	-1.508647	2.845431	down	0.781259	0.607044	0.639823	-0.750567	-1.140205	-0.607044	MIR17HG	miR-17-92	FALSE	Homo sapi	407975		92002655	chr13	92002596	+	hs|13q31.3	NR_027350	chr13:9200	NR_027350	Hs.24115
A_24_P362	-3.530226	-1.81976	3.530226	down	0.798943	0.767449	0.812597	-1.013392	-1.299449	-0.767449	PFKFB4	6-phosphot	FALSE	Homo sapi	5210	GO:000597	48555513	chr3	48555454	-	hs|3p21.31	NM_00456	chr3:48555	NM_00456	Hs.476217
A_21_P001	-2.777405	-1.473738	2.777405	down	0.641309	0.778931	0.73971	-0.779646	-0.641309	-0.840308	XLOC_l2_008976		FALSE	BROAD Institute lincRNA (XLOC_l2			14724906	chr21	14724847	+	hs|21q11.2		chr21:14724847-14724906		
A_33_P341	-2.203909	-1.140065	2.203909	down	0.46046	0.292724	0.670567	-0.292724	-0.443725	-1.259995	DOCK9	dedicator c	FALSE	dedicator c	23348	GO:000551	99461622	chr13	99461563	-	hs|13q32.3	AK090793	chr13:99461622-99461563		
A_22_P000	-2.899866	-1.535986	2.899866	down	0.682168	0.787447	0.85046	-0.842514	-0.763203	-0.682168	SNHG23	small nucle	FALSE	small nucle	1.01E+08		1.01E+08	chr14	1.01E+08	+	hs|14q32.3	XR_424548	chr14:1014	XR_424548	Hs.709410
A_24_P378	-2.718394	-1.442755	2.718394	down	0.691636	0.74183	0.735592	-0.718149	-0.74942	-0.691636	IRF7	interferon	FALSE	Homo sapi	3665	GO:000551	612702	chr11	612643	-	hs|11p15.5	NM_00403	chr11:6127	NM_00403	Hs.166120
A_24_P315	-2.78719	-1.478811	2.78719	down	0.712752	0.666972	0.569116	-0.695425	-1.223052	-0.569116	POM121L8	POM121 tr	FALSE	Homo sapi	29797		21637901	chr22	21637842	+	hs|22q11.2	NR_024583	chr22:2163	NR_024583	Hs.396963
A_32_P194	-2.81051	-1.490832	2.81051	down	0.752685	0.695041	0.652533	-0.652533	-0.984093	-0.73561	SUN2	Sad1 and U	FALSE	Homo sapi	25777	GO:000551	39132019	chr22	39131960	-	hs|22q13.1	NM_01537	chr22:3913	NM_01537	Hs.517622
A_24_P109	-3.010278	-1.589897	3.010278	down	0.75134	0.797905	0.651177	-0.930277	-0.651177	-0.987814	NBEAL1	neurobeacl	FALSE	Homo sapi	65065		2.04E+08	chr2	2.04E+08	+	hs|2q33.2	NM_00111	chr2:20391	NM_00111	Hs.648846
A_33_P342	-2.62731	-1.393586	2.62731	down	1.089545	0.793003	0.549374	-0.549374	-0.574404	-0.625058	TMEM40	transmemb	FALSE	Homo sapi	55287	GO:001602	12775584	chr3	12775525	-	hs|3p25.2	NM_00128	chr3:12775	NM_00128	Hs.475502
A_21_P000	-2.021706	-1.015573	2.021706	down	0.425256	0.442077	0.445431	-0.728203	-0.425256	-0.580496	lnc-PEAK1.	lnc-PEAK1.	FALSE	LNCipedia lincRNA (lnc-PEAK1.1-1)			77657555	chr15	77649433	-	hs|15q24.3		chr15:77657555-77649433		
A_33_P381	-2.668587	-1.416076	2.668587	down	0.652691	0.707198	0.647815	-0.647815	-0.908835	-0.683874	SEMA3F	sema doma	FALSE	Homo sapi	6405	GO:002178	50226015	chr3	50225956	+	hs|3p21.31	NM_00418	chr3:50225	NM_00418	Hs.32981
A_33_P325	-2.681646	-1.423119	2.681646	down	0.630035	0.681347	0.625655	-0.857494	-0.625654	-0.849171	HAPLN3	hyaluronar	FALSE	Homo sapi	145864	GO:000150	89420640	chr15	89420581	-	hs|15q26.1	NM_17823	chr15:8942	NM_17823	Hs.447530
A_32_P465	-2.05966	-1.042406	2.05966	down	0.551583	0.470901	0.351496	-0.691726	-0.351496	-0.710018	RHBDL2	rhomboid,	FALSE	Homo sapi	54933	GO:000588	39351574	chr1	39351515	-	hs|1p34.3	NM_01782	chr1:39351	NM_01782	Hs.524626
A_33_P331	-2.31038	-1.20813	2.31038	down	0.540625	0.567202	0.550521	-0.540625	-0.869519	-0.555899	TAGLN2	transgelin 2	FALSE	Homo sapi	8407	GO:000551	1.6E+08	chr1	1.6E+08	-	hs|1q23.2	NM_00127	chr1:15988	NM_00127	Hs.517168
A_23_P314	-2.94164	-1.556621	2.94164	down	0.617107	0.576519	0.682995	-0.752481	-1.464241	-0.576519	UST	uronyl-2-su	FALSE	Homo sapi	10090	GO:003020	1.49E+08	chr6	1.49E+08	+	hs|6q25.1	NM_00571	chr6:14939	NM_00571	Hs.657370
A_23_P156	-2.489782	-1.31602	2.489782	down	0.504323	0.500586	0.493277	-0.930098	-0.493278	-1.026499	METTL21A	methyltran	FALSE	Homo sapi	151194	GO:000551	2.08E+08	chr2	2.08E+08	-	hs|2q33.3	NM_00112	chr2:20847	NM_00112	Hs.664764
A_33_P336	-3.949502	-1.981671	3.949502	down	0.997992	0.952337	0.864808	-1.082047	-0.864808	-1.18302	MGP	matrix Gla	FALSE	Homo sapi	4256	GO:003101	15034886	chr12	15034827	-	hs|12p12.3	NM_00119	chr12:1503	NM_00119	Hs.365706
A_33_P339	-2.712155	-1.43944	2.712155	down	0.74435	0.757026	0.631019	-0.836859	-0.631019	-0.718047	BRWD1	bromodom	FALSE	Homo sapi	54014	GO:000367	40574389	chr21	40574330	-	hs|21q22.2	NM_01896	chr21:4057	NM_01896	Hs.654740
A_24_P316	-2.112899	-1.079224	2.112899	down	0.452101	0.525169	0.469059	-0.452101	-0.709776	-0.629466	NT5E	5'-nucleoti	FALSE	Homo sapi	4907	GO:000588	86181005	chr6	86176992	+	hs|6q14.3	NM_00252	chr6:86176	NM_00252	Hs.153952
A_23_P216	-2.161946	-1.112331	2.161946	down	0.445258	0.389356	0.549217	-0.597159	-0.389356	-0.966646	PHF20L1	PHD finger	FALSE	PHD finger	51105	GO:000551	1.34E+08	chr8	1.34E+08	+	hs|8q24.22	AB101203	chr8:133837383-133837442		
A_23_P677	-2.199588	-1.137233	2.199588	down	0.612825	0.523881	0.565582	-0.523881	-0.596412	-0.589118	BARD1	BRCA1 asso	FALSE	Homo sapi	580	GO:000551	2.16E+08	chr2	2.16E+08	-	hs|2q35	NM_00046	chr2:21559	NM_00046	Hs.591642
A_33_P331	-2.113414	-1.079575	2.113414	down	0.463456	0.543444	0.474285	-0.650502	-0.463455	-0.643584	PLOD2	procollager	FALSE	Homo sapi	5352	GO:000166	1.46E+08	chr3	1.46E+08	-	hs|3q24	NM_18294	chr3:14578	NM_18294	Hs.477866
A_22_P000	-2.699844	-1.432876	2.699844	down	0.682774	0.559299	0.683664	-0.559299	-0.750529	-1.063064	FAM13A-AS	FAM13A ar	FALSE	Homo sapi	285512		89649834	chr4	89649775	+	hs|4q22.1	NR_002806	chr4:89649	NR_002806	Hs.744957
A_23_P259	-3.766356	-1.913169	3.766356	down	0.878453	0.941841	0.820776	-1.121109	-0.820776	-1.156553	CLIC4	chloride int	FALSE	Homo sapi	25932	GO:000551	25167308	chr1	25166518	+	hs|1p36.11	NM_01394	chr1:25166	NM_01394	Hs.440544
A_23_P333	-3.388722	-1.760742	3.388722	down	0.959034	0.806768	0.868335	-0.819219	-1.022101	-0.806768	IGSF10	immunoglo	FALSE	Homo sapi	285313	GO:000150	1.51E+08	chr3	1.51E+08	-	hs|3q25.1	NM_17882	chr3:15115	NM_17882	Hs.58561
A_32_P222	-2.339429	-1.226156	2.339429	down	0.569959	0.52391	0.602761	-0.52391	-0.602536	-0.855394	ARHGEF37	Rho guanin	FALSE	Homo sapi	389337	GO:000573	1.49E+08	chr5	1.49E+08	+	hs|5q32	NM_00100	chr5:14901	NM_00100	Hs.256206
A_23_P105	-2.317498	-1.212568	2.317498	down	0.479555	0.527344	0.460261	-0.888206	-0.460261	-0.822078	FRY	furry homo	FALSE	Homo sapi	10129	GO:000573	32870617	chr13	32870558	+	hs|13q13.1	NM_02303	chr13:3287	NM_02303	Hs.507669
A_33_P332	-2.048354	-1.034465	2.048354	down	0.369535	0.442611	0.42434	-0.825951	-0.369535	-0.671424			FALSE	AY247741 TRB2 protein {Bos tauru			12864918	chr2	12864859	+	hs|2p24.3		chr2:012864859-012864918		
A_33_P340	-3.345913	-1.7424	3.345913	down	0.854407	0.833149	0.805509	-0.922631	-0.805509	-1.005995	NFASC	neurofascir	FALSE	Homo sapi	23114	GO:000551	2.05E+08	chr1	2.05E+08	+	hs|1q32.1	NM_00100	chr1:20498	NM_00100	Hs.13349
A_23_P348	-2.359295	-1.238356	2.359295	down	0.529963	0.539216	0.449132	-0.840856	-0.449133	-0.906768	NUAK1	NUAK fami	FALSE	Homo sapi	9891	GO:000551	1.06E+08	chr12	1.06E+08	-	hs|12q23.3	NM_01484	chr12:1064	NM_01484	Hs.524692
A_22_P000	-2.026608	-1.019067	2.026608	down	0.428805	0.477399	0.609201	-0.432907	-0.680084	-0.428805	MIR612	microRNA	FALSE	Homo sapi	693197		65212028	chr11	65211969	+	hs|11q13.1	NR_030343	chr11:6521	NR_030343	Hs.523789
A_23_P213	-2.054555	-1.038826	2.054555	down	0.437865	0.441979	0.405002	-0.694214	-0.405002	-0.732416	AHNAK	AHNAK nuc	FALSE	Homo sapi	79026	GO:000551	62201142	chr11	62201083	-	hs|11q12.3	NM_02406	chr11:6220	NM_02406	Hs.502756
A_23_P303	-2.856134	-1.514064	2.856134	down	0.667311	0.869938	0.916735	-0.667311	-0.722635	-0.698261	TRIM7	tripartite m	FALSE	Homo sapi	81786	GO:000573	1.81E+08	chr5	1.81E+08	-	hs|5q35.3	NM_03334	chr5:18063	NM_03334	Hs.487412
A_21_P000	-3.089168	-1.627219	3.089168	down	0.838828	0.791393	0.79606	-0.824921	-0.791393	-0.839061	SNORD114	small nucle	FALSE	Homo sapi	767606		1.01E+08	chr14	1.01E+08	+	hs|14q32.3	NR_003219	chr14:1014	NR_003219	
A_24_P126	-2.047696	-1.034001	2.047696	down	0.450073	0.4463	0.449927	-0.697778	-0.4463	-0.611625	CAV1	caveolin 1,	FALSE	Homo sapi	857	GO:000551	1.16E+08	chr7	1.16E+08	+	hs|7q31.2	NM_00175	chr7:11619	NM_00175	Hs.74034
A_22_P000	-3.134779	-1.648364	3.134779	down	0.737891	0.77928	0.876091	-0.804342	-1.009598	-0.737891			FALSE	PREDICTED: Homo sapiens unchara			1.64E+08	chr4	1.64E+08	+	hs|4q32.2	XR_425799	chr4:16366	XR_425799	
A_33_P339	-2.106369	-1.074758	2.106369	down	0.503445	0.521882	0.492874	-0.628545	-0.492873	-0.584656	PARP10	poly (ADP-r	FALSE	Homo sapi	84875	GO:000551	1.45E+08	chr8	1.45E+08	-	hs|8q24.3	NM_03278	chr8:14505	NM_03278	Hs.348609

A_33_P384	-2.011365	-1.008175	2.011365	down	0.375873	0.239459	0.445631	-0.970754	-0.239459	-0.753349	B4GALNT1	beta-1,4-N	FALSE	Homo sapi	2583	GO:003017	58020014	chr12	58019955	-	hs|12q13.3	NM_00147	chr12:5802	NM_00147	Hs.159481
A_23_P924	-2.207777	-1.142595	2.207777	down	0.584928	0.558004	0.495939	-0.638255	-0.495939	-0.654719	TLR2	toll-like rec	FALSE	Homo sapi	7097	GO:000551	1.55E+08	chr4	1.55E+08	+	hs|4q31.3	NM_00326	chr4:15462	NM_00326	Hs.519033
A_23_P211	-2.057778	-1.041088	2.057778	down	0.535855	0.466614	0.303432	-0.556299	-0.303432	-0.95763	PAXBP1	PAX3 and P	FALSE	Homo sapi	94104	GO:000107	34131469	chr21	34131410	-	hs|21q22.1	NM_01332	chr21:3413	NM_01332	Hs.644004
A_23_P146	-2.64869	-1.405279	2.64869	down	0.611743	0.531315	0.651996	-0.96501	-0.531315	-0.924459	ASAP1-IT1	ASAP1 intr	FALSE	Homo sapi	29065		1.31E+08	chr8	1.31E+08	-	hs|8q24.21	NR_00276	chr8:13130	NR_00276	Hs.639318
A_23_P691	-3.056921	-1.612079	3.056921	down	0.670176	0.732768	0.68456	-1.034634	-0.670176	-1.043924	P3H2	prolyl 3-hy	FALSE	Homo sapi	55214	GO:003141	1.9E+08	chr3	1.9E+08	-	hs|3q28	NM_01819	chr3:18967	NM_01819	Hs.374191
A_23_P213	-2.043762	-1.031227	2.043762	down	0.255411	0.526598	0.260494	-0.846728	-0.255411	-0.949039	ZGRF1	zinc finger,	FALSE	Homo sapi	55345	GO:000827	1.13E+08	chr4	1.13E+08	-	hs|4q25	NM_01839	chr4:11346	NM_01839	Hs.380346
A_23_P363	-4.639966	-2.214114	4.639966	down	1.035446	1.018358	1.03956	-1.28443	-1.018358	-1.246191	KRT86	keratin 86,	FALSE	Homo sapi	3892	GO:000519	52702897	chr12	52702840	+	hs|12q13.1	NM_00228	chr12:5270	NM_00228	Hs.278658
A_24_P189	-2.235305	-1.160472	2.235305	down	0.602438	0.566569	0.552299	-0.552299	-0.594816	-0.612994	ENDOD1	endonuclea	FALSE	Homo sapi	23052	GO:000451	94865371	chr11	94865312	+	hs|11q21	NM_01503	chr11:9486	NM_01503	Hs.167115
A_33_P325	-2.002753	-1.001984	2.002753	down	0.422109	0.304401	0.351663	-0.839442	-0.304401	-0.783937	PCDH18	protocadhe	FALSE	Homo sapi	54510	GO:000588	1.38E+08	chr4	1.38E+08	-	hs|4q28.3	NM_01903	chr4:13844	NM_01903	Hs.591691
A_23_P667	-2.061876	-1.043958	2.061876	down	0.301755	0.617328	0.45995	-0.630633	-0.301755	-0.820452	KRT19	keratin 19,	FALSE	Homo sapi	3880	GO:000551	39679977	chr17	39679918	-	hs|17q21.2	NM_00227	chr17:3967	NM_00227	Hs.654568
A_33_P332	-2.360045	-1.238814	2.360045	down	0.750686	0.414589	0.822022	-0.481446	-0.83311	-0.414589	SMG5	SMG5 nons	FALSE	Homo sapi	23381	GO:000551	1.56E+08	chr1	1.56E+08	-	hs|1q22	NM_01532	chr1:15623	NM_01532	Hs.516837
A_22_P000	-3.047384	-1.607571	3.047384	down	0.787519	0.791256	0.724445	-0.952767	-0.724445	-0.842281	MEG8	maternally	FALSE	maternally	79104		1.01E+08	chr14	1.01E+08	+	hs|14q32.2	XR_424550	chr14:1013	XR_424550	
A_33_P331	-3.88181	-1.95673	3.88181	down	0.962562	0.931154	0.726808	-1.740321	-0.782536	-0.726808	HMHA1	histocompa	FALSE	Homo sapi	23526	GO:004354	1086626	chr19	1086567	+	hs|19p13.3	NM_01229	chr19:1086	NM_01229	Hs.465521
A_23_P201	-2.918428	-1.545192	2.918428	down	0.739519	0.761652	0.781673	-0.781996	-0.831216	-0.739519	IFI6	interferon,	FALSE	Homo sapi	2537	GO:000573	27992896	chr1	27992837	-	hs|1p36.11	NM_02287	chr1:27992	NM_02287	Hs.523847
A_22_P000	-2.266721	-1.180607	2.266721	down	0.672433	0.436709	0.709276	-0.436709	-0.783166	-0.503528	MEG3	maternally	FALSE	Homo sapi	55384		1.01E+08	chr14	1.01E+08	+	hs|14q32.2	NR_03335	chr14:1012	NR_03335	Hs.654863
A_32_P950	-2.391467	-1.257896	2.391467	down	0.828934	0.762093	0.441771	-0.691687	-0.607431	-0.441771	AK4	adenylate k	FALSE	Homo sapi	205	GO:004603	65694158	chr1	65694099	+	hs|1p31.3	NM_00100	chr1:65694	NM_00100	Hs.10862
A_23_P626	-2.557864	-1.354939	2.557864	down	0.58086	0.685812	0.669019	-0.78764	-0.58086	-0.760627	MX2	MX dynam	FALSE	Homo sapi	4600	GO:001922	42780560	chr21	42780501	+	hs|21q22.3	NM_00246	chr21:4278	NM_00246	Hs.926
A_23_P139	-3.985134	-1.994628	3.985134	down	0.961979	1.008832	0.980021	-0.961979	-1.049024	-1.022051	OASL	2'-5'-oligoa	FALSE	Homo sapi	8638	GO:000372	1.21E+08	chr12	1.21E+08	-	hs|12q24.3	NM_00373	chr12:1214	NM_00373	Hs.118633
A_33_P340	-2.983363	-1.576939	2.983363	down	0.705978	0.728736	0.596332	-1.059225	-0.596332	-1.044215	NR2F1	nuclear rec	FALSE	Homo sapi	7025	GO:000551	92929776	chr5	92929717	+	hs|5q15	NM_00565	chr5:92929	NM_00565	Hs.519445
A_23_P261	-2.325993	-1.217847	2.325993	down	0.710307	0.608529	0.495005	-0.598633	-0.495005	-0.746062	RORA	RAR-relate	FALSE	Homo sapi	6095	GO:000551	60789716	chr15	60789657	-	hs|15q22.2	NM_13426	chr15:6078	NM_13426	Hs.560343
A_21_P000	-3.574314	-1.837666	3.574314	down	0.997314	0.923581	0.883452	-0.9201	-0.905099	-0.883452	SNORD114	small nucle	FALSE	Homo sapi	767609		1.01E+08	chr14	1.01E+08	+	hs|14q32.3	NR_00322	chr14:1014	NR_00322	Hs.709862
A_33_P338	-2.054773	-1.038979	2.054773	down	0.601222	0.500502	0.502475	-0.503747	-0.500502	-0.508489	TREX1	three prim	FALSE	Homo sapi	11277	GO:003240	48509034	chr3	48508975	+	hs|3p21.31	NM_01638	chr3:48508	NM_01638	Hs.707026
A_33_P340	-2.441206	-1.287594	2.441206	down	0.821903	0.517053	0.760711	-0.719602	-0.517053	-0.52646	PRR34-AS1	PRR34 anti	FALSE	Homo sapi	150381		46454321	chr22	46454262	+	hs|22q13.3	NR_02703	chr22:4645	NR_02703	Hs.659113
A_33_P321	-2.376993	-1.249138	2.376993	down	0.47408	0.677017	0.555462	-0.935547	-0.47408	-0.631228	DBNL	drebrin-like	FALSE	Homo sapi	28988	GO:000551	44111108	chr7	44111049	+	hs|7p13	AB209486	chr7:44111049-44111108		
A_24_P690	-3.692751	-1.884696	3.692751	down	0.907522	0.866368	0.922283	-1.104953	-0.866368	-0.986594	ENC1	ectoderma	FALSE	Homo sapi	8507	GO:001714	73924314	chr5	73924255	-	hs|5q13.3	NM_00363	chr5:73924	NM_00363	Hs.744844
A_33_P329	-2.106333	-1.074734	2.106333	down	0.554695	0.528708	0.500162	-0.51486	-0.500162	-0.625615	TMEM175	transmemb	FALSE	Homo sapi	84286	GO:001602	952441	chr4	952382	+	hs|4p16.3	NM_00129	chr4:95238	NM_00129	Hs.478936
A_23_P151	-2.252786	-1.17171	2.252786	down	0.444391	0.475002	0.426973	-0.863386	-0.426972	-0.878407	LIMA1	LIM domai	FALSE	Homo sapi	51474	GO:000551	50569850	chr12	50569791	-	hs|12q13.1	NM_01635	chr12:5056	NM_01635	Hs.525419
A_23_P211	-2.213655	-1.14643	2.213655	down	0.378214	0.449257	0.495632	-0.851028	-0.378214	-0.886946	COL18A1	collagen, ty	FALSE	Homo sapi	80781	GO:000551	46933617	chr21	46933558	+	hs|21q22.3	NM_03058	chr21:4693	NM_03058	Hs.517356
A_33_P324	-2.63184	-1.396072	2.63184	down	0.644121	0.626663	0.725398	-0.626663	-0.869638	-0.695733	ZEB2	zinc finger	FALSE	Homo sapi	9839	GO:002184	1.45E+08	chr2	1.45E+08	-	hs|2q22.3	NM_01479	chr2:14518	NM_01479	Hs.34871
A_19_P003	-3.042016	-1.605028	3.042016	down	0.637268	0.707575	0.787078	-0.637268	-1.333012	-0.712884	ZSWIM6	zinc finger,	FALSE	Homo sapi	57688	GO:004881	60768799	chr5	60768740	+	hs|5q12.1	NM_02092	chr5:60768	NM_02092	Hs.744939
A_24_P307	-2.016972	-1.012191	2.016972	down	0.724224	0.345169	0.742967	-0.345169	-0.483221	-0.395823			FALSE	glyceraldehyde 3 phosphate dehyc			1.2E+08	chr1	1.2E+08	-	hs|1p12		chr1:120139395-120139336		
A_23_P314	-26.63742	-4.735383	26.63742	down	2.395254	2.394649	2.348026	-2.348025	-2.370881	-2.349313	SUSD2	sushi doma	FALSE	Homo sapi	56241	GO:000551	24585037	chr22	24584978	+	hs|22q11.2	NM_01960	chr22:2458	NM_01960	Hs.131819
A_21_P000	-6.856596	-2.777493	6.856596	down	1.130357	1.099072	1.151758	-1.933758	-1.099072	-1.918462	lnc-SUPT3H	lnc-SUPT3H	FALSE	Homo sapiens cDNA clone IMAGE:			45539917	chr6	45539858	-	hs|6p21.1		chr6:45539917-455398		Hs.714205
A_23_P992	-2.075272	-1.0533	2.075272	down	0.655334	0.726855	0.251285	-0.251285	-0.36885	-0.906292	LIN7A	lin-7 homo	FALSE	Homo sapi	8825	GO:000551	81191501	chr12	81191442	-	hs|12q21.3	NM_00466	chr12:8119	NM_00466	Hs.144333
A_22_P000	-2.21011	-1.144118	2.21011	down	0.420504	0.681005	0.456196	-0.420504	-0.792453	-0.661694	FAM110A	family with	FALSE	Homo sapi	83541	GO:000551	826379	chr20	826320	+	hs|20p13	NM_00128	chr20:8263	NM_00128	Hs.574822
A_33_P321	-8.049391	-3.00888	8.049391	down	1.451937	1.441496	1.468622	-1.441496	-1.563994	-1.659095	RIMS1	regulating s	FALSE	Homo sapi	22999	GO:000551	73112480	chr6	73112421	+	hs|6q13	NM_01498	chr6:73112	NM_01498	Hs.485729
A_33_P338	-2.122572	-1.085813	2.122572	down	0.554412	0.36891	0.406768	-0.625103	-0.933337	-0.36891	ABLIM1	actin bindir	FALSE	Homo sapi	3983	GO:000551	1.16E+08	chr10	1.16E+08	-	hs|10q25.3	NM_00100	chr10:1161	NM_00100	Hs.438236
A_23_P133	-2.18915	-1.130371	2.18915	down	0.446428	0.697722	0.413431	-0.637483	-0.413431	-0.782618	MCTP1	multiple C2	FALSE	Homo sapi	79772	GO:000554	94042412	chr5	94042353	-	hs|5q15	NM_02471	chr5:94042	NM_02471	Hs.655087
A_23_P772	-4.404722	-2.139051	4.404722	down	1.141033	1.01498	1.144557	-1.017646	-1.01498	-1.083957	HAPLN1	hyaluronar	FALSE	Homo sapi	1404	GO:003101	82937382	chr5	82937323	-	hs|5q14.3	NM_00188	chr5:82937	NM_00188	Hs.2799
A_23_P476	-2.038707	-1.027655	2.038707	down	0.379909	0.42974	0.314931	-0.856718	-0.314931	-0.786735	PHLDA2	pleckstrin h	FALSE	Homo sapi	7262	GO:004599	2949755	chr11	2949696	-	hs|11p15.4	NM_00331	chr11:2949	NM_00331	Hs.154036
A_33_P337	-4.595448	-2.200205	4.595448	down	1.016594	1.031591	1.020511	-1.194606	-1.016593	-1.320722	FBXO32	F-box prote	FALSE	Homo sapi	114907	GO:000551	1.25E+08	chr8	1.25E+08	-	hs|8q24.13	NM_05822	chr8:12451	NM_05822	Hs.403933
A_33_P333	-2.765852	-1.467724	2.765852	down	0.769923	0.823627	0.528877	-0.962002	-0.789866	-0.528877	MGA	MGA, MAX	FALSE	Homo sapi	23269	GO:000566	42021447	chr15	42021388	+	hs|15q15.1	NM_00108	chr15:4202	NM_00108	Hs.187569
A_33_P341	-2.529526	-1.338867	2.529526	down	0.658817	0.670024	0.641783	-0.705093	-0.641783	-0.699101	ODF3B	outer dens	FALSE	Homo sapi	440836		50968929	chr22	50968870	-	hs|22q13.3	NM_00101	chr22:5096	NM_00101	Hs.531314
A_23_P253	-2.255561	-1.173486	2.255561	down	0.709283	0.258341	0.525196	-1.004476	-0.258341	-0.764823	MTFR2	mitochond	FALSE	Homo sapi	113115	GO:000906	1.37E+08	chr6	1.37E+08	-	hs|6q23.3	NM_13841	chr6:13655	NM_13841	Hs.121536
A_33_P335	-2.005431	-1.003912	2.005431	down	0.552542	0.299498	0.83134	-0.672304	-0.299498	-0.356555	PIEZO2	piezo-type	FALSE	Homo sapi	63895	GO:000838	10742608	chr18	10742549	-	hs|18p11.2	NM_02206	chr18:1074	NM_02206	Hs.436902
A_33_P341	-2.039852	-1.028465	2.039852	down	0.384097	0.336682	0.426231	-0.46455	-1.137151	-0.336682	SCARF2	scavenger r	FALSE	Homo sapi	91179	GO:000551	20779338	chr22	20779279	-	hs|22q11.2	NM_15333	chr22:2077	NM_15333	Hs.474251
A_24_P181	-2.004564	-1.003289	2.004564	down	0.539024	0.430989	0.389301	-0.623755	-0.389301	-0.637497	ASPH	aspartate b	FALSE	Homo sapi	444	GO:000551	62537754	chr8	62537695	-	hs|8q12.3	NM_03246	chr8:62537	NM_03246	Hs.332422
A_23_P883	-2.483904	-1.312609	2.483904	down	0.679955	0.606691	0.648903	-0.675586	-0.720001	-0.606691	HSPA2	heat shock	FALSE	Homo sapi	3306	GO:000551	65009597	chr14	65009538	+	hs|14q23.3	NM_02197	chr14:6500	NM_02197	Hs.432648
A_23_P135	-2.084653	-1.059807	2.084653	down	0.300427	0.374255	0.421513	-0.820709	-0.300427	-0.962091	RALGDS	ral guanine	FALSE	Homo sapi	5900	GO:004354	1.36E+08	chr9	1.36E+08	-	hs|9q34.2	NM_00104	chr9:13597	NM_00104	Hs.106185
A_24_P926	-2.086412	-1.061024	2.086412	down	0.421468	0.507433	0.506979	-0.68534	-0.421468	-0.640384	NKTR	natural kille	FALSE	Homo sapi	4820	GO:000041	42672701	chr3	42672047	+	hs|3p22.1	NM_00538	chr3:42672	NM_00538	Hs.529509
A_23_P101	-2.07625	-1.05398	2.07625	down	0.4376	0.448276	0.530606	-0.750869	-0.4376	-0.55699	TMEM106A	transmemb	FALSE	Homo sapi	113277	GO:000815	41372045	chr17	41371986	+	hs|17q21.3	NM_00129	chr17:4137	NM_00129	Hs.536474
A_21_P001	-3.106512	-1.635296	3.106512	down	0.726872	0.848768	0.690808	-0.951219	-0.690808	-0.997411			FALSE	Homo sapiens clone DNA57695 G\			60233038	chr18	60232979	+	hs|18q21.3	AY358662	chr18:60232979-6023		Hs.656351
A_33_P321	-4.350466	-2.12117	4.350466	down	1.060022	1.107926	0.893241	-1.082685	-0.893241	-1.326395	LEPROT	leptin rece	FALSE	Homo sapi	54741	GO:006040	65889034	chr1	65888975	+	hs|1p31.3	AK094801	chr1:65888975-65889034		
A_22_P000	-5.886973	-2.557526	5.886973	down	0.993585	1.080459	0.954617	-1.6669	-0.954617	-2.0224	CCNT2-AS1	CCNT2 anti	FALSE	Homo sapi	1E+08		1.36E+08	chr2	1.36E+08	-	hs|2q21.3	NR_03654	chr2:13562	NR_03654	Hs.729028
A_33_P329	-2.387174	-1.255304	2.387174	down	0.476404	0.606931	0.454219	-1.032487	-0.454219	-0.741651	FNDC1	fibronectin	FALSE	Homo sapi	84624	GO:003196	1.6E+08	chr6	1.6E+08	+	hs|6q25.3	NM_03253	chr6:15964	NM_03253	Hs.520525
A_33_P333	-2.202667	-1.139252	2.202667	down	0.473095	0.618939	0.503901	-0.582317	-0.473095	-0.766407	BTN2A1	butyrophili	FALSE	Homo sapi	11120	GO:000662	26465609	chr6	26465550	+	hs|6p22.2	NM_00119	chr6:26465	NM_00119	Hs.159028
A_23_P694	-3.397695	-1.764557	3.397695	down	0.836741	0.855649	0.801682	-0.955195	-0.801682	-1.042722	CLEC3B	C-type lect	FALSE	Homo sapi	7123	GO:000165	45077561	chr3	45077502	+	hs|3p21.31	NM_00327	chr3:45077	NM_00327	Hs.476092
A_32_P114	-2.44331	-1.288837	2.44331	down	0.505464	0.547866	0.49837	-0.920918	-0.49837	-0.895522	IKZF2	IKAROS fan	FALSE	Homo sapi	22807	GO:000551	2.14E+08	chr2	2.14E+08	-	hs|2q34	NM_00107	chr2:21386	NM_00107	Hs.604950

A_33_P328	-5.380405	-2.427715	5.380405	down	1.066016	1.095375	1.044191	-1.192438	-1.044191	-1.840933	B3GALT2	UDP-Gal:be	FALSE	Homo sapi	8707	GO:000931	1.93E+08	chr1	1.93E+08	-	hs|1q31.2	NM_00378	chr1:19314	NM_00378	Hs.518834
A_23_P132	-2.554455	-1.353015	2.554455	down	0.639275	0.645934	0.638622	-0.754622	-0.741972	-0.638622	USP18	ubiquitin sp	FALSE	Homo sapi	11274	GO:000551	18659677	chr22	18659618	+	hs|22q11.2	NM_01741	chr22:1865	NM_01741	Hs.38260
A_23_P977	-2.171849	-1.118924	2.171849	down	0.453487	0.451234	0.435694	-0.781721	-0.435695	-0.798941	TXNIP	thioredoxir	FALSE	Homo sapi	10628	GO:000551	1.45E+08	chr1	1.45E+08	+	hs|1q21.1	NM_00647	chr1:14544	NM_00647	Hs.709057
A_23_P927	-4.036809	-2.013215	4.036809	down	0.71256	0.90393	0.847615	-1.2015	-0.71256	-1.66148	HSPB3	heat shock	FALSE	Homo sapi	8988	GO:000698	53752143	chr5	53752084	+	hs|5q11.2	NM_00630	chr5:53752	NM_00630	Hs.41707
A_23_P132	-2.528947	-1.338537	2.528947	down	0.601147	0.669788	0.645986	-0.755667	-0.601148	-0.741874	GGA1	golgi-assoc	FALSE	Homo sapi	26088	GO:000551	38029539	chr22	38029480	+	hs|22q13.1	NM_00100	chr22:3802	NM_00100	Hs.499158
A_24_P366	-2.895091	-1.533608	2.895091	down	0.587352	0.663555	0.662735	-1.065094	-0.587353	-1.034737	SYNGR2	synaptogyr	FALSE	Homo sapi	9144	GO:003159	76168805	chr17	76168747	+	hs|17q25.3	BC105992	chr17:76168747-7616		Hs.464210
A_23_P321	-2.213931	-1.14661	2.213931	down	0.467912	0.471256	0.468607	-0.688242	-0.467912	-0.875901	PDZD8	PDZ domai	FALSE	Homo sapi	118987	GO:003555	1.19E+08	chr10	1.19E+08	-	hs|10q25.3	NM_17379	chr10:1190	NM_17379	Hs.501149
A_23_P705	-2.009767	-1.007028	2.009767	down	0.458313	0.555942	0.47418	-0.526955	-0.547383	-0.458313	SLC39A7	solute carri	FALSE	Homo sapi	7922	GO:000551	33171859	chr6	33171800	+	hs|6p21.32	NM_00697	chr6:33171	NM_00697	Hs.631995
A_33_P331	-2.064665	-1.045908	2.064665	down	0.447598	0.463798	0.420298	-0.695919	-0.420298	-0.689813	CFH	complemer	FALSE	Homo sapi	3075	GO:000695	1.97E+08	chr1	1.97E+08	+	hs|1q31.3	NM_00101	chr1:19665	NM_00101	Hs.363396
A_33_P328	-3.806819	-1.928586	3.806819	down	0.807792	0.903016	0.931539	-1.161442	-0.807792	-1.174178	FMO6P	flavin conta	FALSE	Homo sapi	388714		1.71E+08	chr1	1.71E+08	+	hs|1q24.3	NR_00260	chr1:17113	NR_00260	Hs.448988
A_33_P330	-2.252086	-1.171262	2.252086	down	0.600992	0.222169	0.397014	-0.818228	-0.222169	-1.253213	ATF6B	activating t	FALSE	Homo sapi	1388	GO:000551	32083106	chr6	32083047	-	hs|6p21.33	NM_00438	chr6:32083	NM_00438	Hs.42853
A_24_P254	-2.555253	-1.353466	2.555253	down	0.575714	0.607984	0.666854	-0.785284	-0.575714	-0.848849	ARHGEF9	Cdc42 guar	FALSE	Homo sapi	23229	GO:005105	62855397	chrX	62855338	-	hs|Xq11.1	NM_01518	chrX:62855	NM_01518	Hs.54697
A_21_P000	-8.195024	-3.034748	8.195024	down	1.176517	1.162332	1.408042	-2.007236	-1.162332	-2.187785	lnc-HMCN1	lnc-HMCN1	FALSE	LNCipedia lincRNA (lnc-HMCN1-2),			1.86E+08	chr1	1.86E+08	+	hs|1q25.3		chr1:185532449-185590104		
A_33_P328	-3.260146	-1.704937	3.260146	down	0.832346	0.800893	0.799504	-0.799503	-1.060864	-0.821701	IFIT3	interferon-	FALSE	Homo sapi	3437	GO:000551	91099821	chr10	91099762	+	hs|10q23.3	NM_00128	chr10:9109	NM_00128	Hs.744072
A_24_P261	-2.159043	-1.110392	2.159043	down	0.504562	0.463524	0.396404	-0.759019	-0.396404	-0.811263	PFKFB3	6-phosphot	FALSE	Homo sapi	5209	GO:000597	6277121	chr10	6277062	+	hs|10p15.1	NM_00456	chr10:6277	NM_00456	Hs.195471
A_24_P298	-2.287126	-1.193536	2.287126	down	0.510056	0.509004	0.555499	-0.647819	-0.509004	-0.849225	AXIN2	axin 2	FALSE	Homo sapi	8313	GO:000551	63554447	chr17	63554388	-	hs|17q24.1	NM_00465	chr17:6355	NM_00465	Hs.156527
A_23_P996	-2.058267	-1.041431	2.058267	down	0.536414	0.499627	0.481505	-0.51898	-0.481505	-0.606261	G2E3	G2/M-phas	FALSE	Homo sapi	55632	GO:000020	31084745	chr14	31084686	+	hs|14q12	NM_01776	chr14:3108	NM_01776	Hs.509008
A_33_P329	-2.816038	-1.493667	2.816038	down	0.527506	0.8997	0.806794	-0.527506	-0.600439	-1.119056	CEP55	centrosoma	FALSE	Homo sapi	55165	GO:000551	95279537	chr10	95279478	+	hs|10q23.3	NM_01813	chr10:9527	NM_01813	Hs.14559
A_33_P340	-4.114944	-2.040873	4.114944	down	0.901011	0.939746	0.872148	-1.222047	-0.872148	-1.315518	CMPK2	cytidine mo	FALSE	Homo sapi	129607	GO:000412	6988526	chr2	6988467	-	hs|2p25.2	NM_20731	chr2:69885	NM_20731	Hs.7155
A_23_P527	-3.167011	-1.663122	3.167011	down	0.805432	0.812923	0.878842	-0.857339	-0.829397	-0.805432	MMP7	matrix met	FALSE	Homo sapi	4316	GO:000278	1.02E+08	chr11	1.02E+08	-	hs|11q22.2	NM_00242	chr11:1023	NM_00242	Hs.2256
A_21_P000	-2.090261	-1.063683	2.090261	down	0.44725	0.552596	0.481068	-0.602414	-0.44725	-0.660471	PEAK1	pseudopod	FALSE	pseudopod	79834	GO:000551	77657560	chr15	77544868	-	hs|15q24.3		chr15:77657560-77544868		
A_33_P321	-2.437749	-1.28555	2.437749	down	0.476476	0.624477	0.538082	-0.858321	-0.476476	-0.882817	CDH2	cadherin 2,	FALSE	Homo sapi	1000	GO:000551	25530995	chr18	25530936	-	hs|18q12.1	NM_00179	chr18:2553	NM_00179	Hs.464829
A_33_P339	-2.057185	-1.040672	2.057185	down	0.443459	0.44329	0.386239	-0.641188	-0.386239	-0.821601	NOG	noggin	FALSE	Homo sapi	9241	GO:000551	54672515	chr17	54672456	+	hs|17q22	NM_00545	chr17:5467	NM_00545	Hs.248201
A_22_P000	-2.1296	-1.090582	2.1296	down	0.495761	0.49927	0.454367	-0.604814	-0.454367	-0.763168			FALSE				20878851	chr2	20878792	+	hs|2p24.1		chr2:20878792-20878851		
A_23_P103	-2.121906	-1.085361	2.121906	down	0.499266	0.514646	0.466881	-0.608829	-0.466881	-0.69958	CFHR3	complemer	FALSE	Homo sapi	10878	GO:007256	1.97E+08	chr1	1.97E+08	+	hs|1q31.3	NM_02102	chr1:19674	NM_02102	Hs.709217
A_23_P845	-2.368804	-1.244159	2.368804	down	0.317467	0.518241	0.527875	-0.95148	-0.317467	-1.099946	LFNG	LFNG O-fuc	FALSE	Homo sapi	3955	GO:004574	2568030	chr7	2567971	+	hs|7p22.3	NM_00104	chr7:25679	NM_00104	Hs.159142
A_23_P374	-3.221953	-1.687935	3.221953	down	0.779651	0.958962	0.793059	-0.875532	-0.779651	-0.876953	ADAM19	ADAM met	FALSE	Homo sapi	8728	GO:000750	1.57E+08	chr5	1.57E+08	-	hs|5q33.3	NM_03327	chr5:15690	NM_03327	Hs.483944
A_24_P212	-2.300036	-1.201656	2.300036	down	0.560844	0.545053	0.402186	-0.944817	-0.749883	-0.402186	MCTP1	multiple C2	FALSE	Homo sapi	79772	GO:000554	94208929	chr5	94208870	-	hs|5q15	NM_02471	chr5:94208	NM_02471	Hs.655087
A_23_P385	-2.548909	-1.34988	2.548909	down	0.895124	0.603875	0.611133	-0.603875	-0.631946	-0.703687	DEPDC7	DEP domai	FALSE	Homo sapi	91614	GO:005105	33054883	chr11	33054824	+	hs|11p13	NM_13916	chr11:3305	NM_13916	Hs.280990
A_33_P324	-2.090266	-1.063687	2.090266	down	0.428518	0.482712	0.411263	-0.663038	-0.411263	-0.794265	AFF1	AF4/FMR2	FALSE	Homo sapi	4299	GO:000551	88036450	chr4	88036391	+	hs|4q22.1	NM_00593	chr4:88036	NM_00593	Hs.480190
A_24_P206	-2.056581	-1.040248	2.056581	down	0.446972	0.534682	0.561725	-0.537658	-0.592735	-0.446972	CRYAB	crystallin, a	FALSE	Homo sapi	1410	GO:000551	1.12E+08	chr11	1.12E+08	-	hs|11q23.1	NM_00188	chr11:1117	NM_00188	Hs.53454
A_23_P394	-2.40203	-1.264254	2.40203	down	0.517966	0.737307	0.451182	-0.967728	-0.451182	-0.667397	DPY19L1	dpy-19-like	FALSE	Homo sapi	23333	GO:000003	34971230	chr7	34971171	-	hs|7p14.3	NM_01528	chr7:34971	NM_01528	Hs.408623
A_23_P502	-2.679194	-1.421799	2.679194	down	0.661458	0.701668	0.648688	-0.680239	-0.924658	-0.648688	RASAL2	RAS proteir	FALSE	Homo sapi	9462	GO:000551	1.78E+08	chr1	1.78E+08	+	hs|1q25.2	NM_17069	chr1:17844	NM_17069	Hs.496139
A_33_P323	-2.417659	-1.273611	2.417659	down	0.541407	0.616538	0.4083	-1.043207	-0.4083	-0.80308	KIF14	kinesin fam	FALSE	Homo sapi	9928	GO:000801	2.01E+08	chr1	2.01E+08	-	hs|1q32.1	NM_01487	chr1:20052	NM_01487	Hs.3104
A_33_P330	-4.481306	-2.163919	4.481306	down	1.083493	1.072535	1.056488	-1.065795	-1.156961	-1.056487	LEPR	leptin rece	FALSE	Homo sapi	3953	GO:004572	66088663	chr1	66088604	+	hs|1p31.3	NM_00119	chr1:66088	NM_00119	Hs.723178
A_23_P431	-2.494543	-1.318775	2.494543	down	0.506086	0.532809	0.530548	-1.020803	-0.506086	-0.859995	ZHX1	zinc fingers	FALSE	Homo sapi	11244	GO:000551	1.24E+08	chr8	1.24E+08	-	hs|8q24.13	NM_00101	chr8:12426	NM_00101	Hs.521800
A_33_P321	-2.409188	-1.268547	2.409188	down	0.512146	0.566717	0.498932	-0.817233	-0.498932	-0.911682	GALNT1	polypeptid	FALSE	Homo sapi	2589	GO:004847	33290012	chr18	33289953	+	hs|18q12.2	NM_02047	chr18:3328	NM_02047	Hs.514806
A_21_P000	-7.139368	-2.835796	7.139368	down	1.078069	1.10838	1.102142	-2.044347	-1.078069	-2.096381	lnc-SOX6-1	lnc-SOX6-1	FALSE	LNCipedia lincRNA (lnc-SOX6-1), lir			15932586	chr11	15932527	-	hs|11p15.2		chr11:15932586-15932527		
A_23_P150	-3.615955	-1.854377	3.615955	down	0.883764	0.961873	0.903262	-0.984572	-0.883763	-0.945896	IGF2	insulin-like	FALSE	Homo sapi	3481	GO:000551	2150453	chr11	2150394	-	hs|11p15.5	NM_00061	chr11:2150	NM_00061	Hs.272259
A_23_P336	-2.608058	-1.382976	2.608058	down	0.594263	0.712886	0.597606	-0.594263	-0.861291	-0.788618	SLCO1B3	solute carri	FALSE	Homo sapi	28234	GO:001632	21036535	chr12	21036476	+	hs|12p12.2	NM_01984	chr12:2103	NM_01984	Hs.504966
A_22_P000	-7.337519	-2.875292	7.337519	down	1.199724	1.090615	1.093234	-1.979084	-1.090615	-2.172604	lnc-TCL1B-	lnc-TCL1B-	FALSE	LNCipedia lincRNA (lnc-TCL1B-2), l			96178179	chr14	96178120	+	hs|14q32.13		chr14:96178120-96178179		
A_24_P365	-2.163139	-1.113126	2.163139	down	0.379705	0.250007	0.653069	-0.974895	-0.250007	-0.831696	COL8A2	collagen, ty	FALSE	Homo sapi	1296	GO:000558	36560922	chr1	36560863	-	hs|1p34.3	NM_00520	chr1:36560	NM_00520	Hs.353001
A_23_P228	-2.644454	-1.40297	2.644454	down	0.66883	0.706483	0.601756	-0.871504	-0.601756	-0.758579	TSPY3	testis speci	FALSE	Homo sapi	728137	GO:000750	9237850	chrY	9237685	+	hs|Yp11.2	NM_00107	chrY:92376	NM_00107	Hs.556121
A_33_P328	-2.191868	-1.132161	2.191868	down	0.431131	0.946174	0.559665	-0.519495	-0.508887	-0.431131	DNM1L	dynamin 1-	FALSE	dynamin 1-	10059	GO:000551	32893017	chr12	32892958	+	hs|12p11.2	AK123906	chr12:32892958-32893017		
A_33_P327	-2.608715	-1.383339	2.608715	down	0.617218	0.655019	0.700426	-0.687979	-0.872159	-0.617218	KRT83	keratin 83,	FALSE	Homo sapi	3889	GO:004263	52709761	chr12	52709702	-	hs|12q13.1	NM_00228	chr12:5270	NM_00228	Hs.720768
A_33_P326	-2.168586	-1.116755	2.168586	down	0.547508	0.54753	0.571366	-0.561712	-0.574641	-0.547508	GP1BB	glycoprotei	FALSE	Homo sapi	2812	GO:000551	19712297	chr22	19712238	+	hs|22q11.2	NM_00040	chr22:1971	NM_00040	Hs.283743
A_24_P143	-2.68106	-1.422804	2.68106	down	0.660677	0.780363	0.701072	-0.660677	-0.687712	-0.777911	TMEM47	transmemb	FALSE	Homo sapi	83604	GO:000815	34646240	chrX	34646181	-	hs|Xp21.1	NM_03144	chrX:34646	NM_03144	Hs.8769
A_22_P000	-2.897261	-1.53469	2.897261	down	0.632147	0.586646	0.710288	-1.053629	-0.586646	-1.034712	lnc-CERS5-	lnc-CERS5-	FALSE	Homo sapiens mRNA; cDNA DKFZp			50579422	chr12	50579363	-	hs|12q13.1	BX647194	chr12:50579422-5057		Hs.731286
A_23_P119	-2.252342	-1.171426	2.252342	down	0.354807	0.417635	0.356423	-1.000749	-1.029856	-0.354807	ICAM5	intercellula	FALSE	Homo sapi	7087	GO:000690	10407381	chr19	10407322	+	hs|19p13.2	NM_00325	chr19:1040	NM_00325	Hs.465862
A_33_P325	-2.34886	-1.231961	2.34886	down	0.48084	0.66606	0.526953	-0.83935	-0.48084	-0.70184	C14orf80	chromoson	FALSE	Homo sapi	283643		1.06E+08	chr14	1.06E+08	+	hs|14q32.3	NM_00113	chr14:1059	NM_00113	Hs.720306
A_33_P352	-2.207614	-1.142488	2.207614	down	0.566941	0.335693	0.374991	-1.041181	-0.335693	-0.772965	LOC284219	uncharacte	FALSE	Homo sapi	284219		8767754	chr18	8767695	+	hs|18p11.2	AK094436	chr18:8767695-876775		Hs.657510
A_23_P394	-2.050778	-1.036172	2.050778	down	0.439911	0.486407	0.488819	-0.641823	-0.439911	-0.611644	PTRF	polymerase	FALSE	Homo sapi	284119	GO:000551	40554536	chr17	40554477	-	hs|17q21.2	NM_01223	chr17:4055	NM_01223	Hs.437191
A_22_P000	-2.21125	-1.144862	2.21125	down	0.537331	0.539932	0.530674	-0.675361	-0.530674	-0.620616	PLCE1-AS1	PLCE1 antis	FALSE	Homo sapi	1E+08		96043203	chr10	96043144	-	hs|10q23.3	NR_03396	chr10:9604	NR_03396	Hs.677562
A_33_P324	-2.002066	-1.00149	2.002066	down	0.402941	0.381569	0.394663	-0.687744	-0.381569	-0.755982	OBSL1	obscurin-lil	FALSE	Homo sapi	23363	GO:000551	2.2E+08	chr2	2.2E+08	-	hs|2q35	NM_01531	chr2:22041	NM_01531	Hs.526594
A_23_P985	-2.47155	-1.305416	2.47155	down	0.592946	0.673295	0.637572	-0.705101	-0.592945	-0.714389	FADS2	fatty acid d	FALSE	Homo sapi	9415	GO:000663	61634813	chr11	61634754	+	hs|11q12.2	NM_00426	chr11:6163	NM_00426	Hs.502745
A_23_P166	-3.770289	-1.914675	3.770289	down	0.912069	0.959126	0.924978	-1.030595	-0.912069	-1.00519	LINC00312	long interg	FALSE	Homo sapi	29931		8615381	chr3	8615322	+	hs|3p26.1	NR_02406	chr3:86153	NR_02406	Hs.433151
A_33_P320	-3.374351	-1.75461	3.374351	down	0.940033	0.92653	0.82477	-0.909086	-0.838641	-0.82477	RASGRP2	RAS guanyl	FALSE	Homo sapi	10235	GO:004300	64508971	chr11	64508912	-	hs|11q13.1	NM_15381	chr11:6450	NM_15381	Hs.99491
A_23_P628	-2.124903	-1.087397	2.124903	down	0.467668	0.549608	0.4341	-0.681835	-0.4341	-0.694882	GBP1	guanylate b	FALSE	Homo sapi	2633	GO:005160	89518848	chr1	89518789	-	hs|1p22.2	NM_00205	chr1:89518	NM_00205	Hs.62661

A_33_P321	-2.239054	-1.162889	2.239054	down		0.661773	0.508172	0.491553	-0.659768	-0.491553	-0.675849	SIRPB1	signal-regu	FALSE	PREDICTED	10326	GO:000551	1558942	chr20	1558883	-		hs|20p13	XM_00671	chr20:1558	XM_006710250	
A_23_P58C	-2.293891	-1.197797	2.293891	down		0.582374	0.560781	0.582273	-0.571059	-0.736124	-0.560781	CCDC80	coiled-coil	FALSE	Homo sapi	151887	GO:003019	1.12E+08	chr3	1.12E+08	-		hs|3q13.2	NM_19951	chr3:11232	NM_19951	Hs.477128
A_21_P00C	-7.375783	-2.882796	7.375783	down		1.625128	1.12424	1.676808	-1.61026	-1.12424	-1.487712	lnc-HNF1A-	lnc-HNF1A-	FALSE	LNCipedia lincRNA (lnc-HNF1A-1),			1.21E+08	chr12	1.21E+08	+		hs|12q24.31		chr12:121343059-121343118		
A_23_P151	-2.087519	-1.061789	2.087519	down		0.399617	0.548516	0.442685	-0.422124	-0.972808	-0.399617	PITPNM1	phosphatid	FALSE	Homo sapi	9600	GO:000581	67261724	chr11	67261492	-		hs|11q13.2	NM_00491	chr11:6726	NM_00491	Hs.372295
A_33_P332	-2.008939	-1.006434	2.008939	down		0.453926	0.411367	0.379485	-0.70268	-0.379485	-0.692358	PLCE1-AS1	PLCE1 antis	FALSE	Homo sapi	1E+08		96039115	chr10	96039056	-		hs|10q23.3	NR_03396	chr10:9603	NR_03396	Hs.677562
A_33_P326	-2.107076	-1.075243	2.107076	down		0.419165	0.433851	0.435232	-0.738153	-0.419165	-0.780163	TMUB1	transmemb	FALSE	Homo sapi	83590	GO:000573	1.51E+08	chr7	1.51E+08	-		hs|7q36.1	NM_03143	chr7:15077	NM_03143	Hs.726215
A_23_P306	-3.376453	-1.755509	3.376453	down		0.845676	0.880066	0.807206	-0.995224	-0.807206	-0.931148	GALNT1	polypeptid	FALSE	Homo sapi	2589	GO:004847	33291379	chr18	33291320	+		hs|18q12.2	NM_02047	chr18:3329	NM_02047	Hs.514806
A_23_P355	-2.42214	-1.276282	2.42214	down		0.517052	0.59696	0.554071	-0.80646	-0.517052	-0.837253	USP54	ubiquitin sp	FALSE	Homo sapi	159195	GO:000551	75257445	chr10	75257386	-		hs|10q22.2	NM_15258	chr10:7525	NM_15258	Hs.657355
A_23_P108	-2.471805	-1.305565	2.471805	down		0.588427	0.682422	0.641892	-0.588427	-0.769568	-0.645958	RPL23AP32	ribosomal p	FALSE	Homo sapi	56969		54756427	chr2	54756368	+		hs|2p16.2	NR_00222	chr2:54756	NR_00222	Hs.657366
A_33_P331	-2.110123	-1.077327	2.110123	down		0.508762	0.592186	0.564316	-0.519929	-0.508762	-0.538026	ITIH3	inter-alpha	FALSE	Homo sapi	3699	GO:001095	52842697	chr3	52842638	+		hs|3p21.1	NM_00221	chr3:52842	NM_00221	Hs.76716
A_32_P206	-2.143031	-1.099653	2.143031	down		0.406648	0.433227	0.475757	-0.760837	-0.406648	-0.815841	DNAH2	dynein, axo	FALSE	Homo sapi	146754	GO:000153	7736989	chr17	7736930	+		hs|17p13.1	NM_02087	chr17:7736	NM_02087	Hs.367649
A_23_P997	-2.161664	-1.112142	2.161664	down		0.589813	0.591754	0.472854	-0.55441	-0.654741	-0.472854	CDKL1	cyclin-depe	FALSE	Homo sapi	8814	GO:000750	50805745	chr14	50805686	-		hs|14q21.3	NM_00419	chr14:5080	NM_00419	Hs.679430
A_23_P218	-2.539453	-1.344518	2.539453	down		0.661305	0.668557	0.65971	-0.65971	-0.685299	-0.698971	SLC38A2	solute carri	FALSE	Homo sapi	54407	GO:001517	46754684	chr12	46754625	-		hs|12q13.1	NM_01897	chr12:4675	NM_01897	Hs.221847
A_23_P487	-12.61247	-3.656779	12.61247	down		1.803609	1.841322	1.676031	-2.061627	-1.676031	-1.911718	DIO2	deiodinase	FALSE	Homo sapi	1734	GO:000588	80664399	chr14	80664340	-		hs|14q31.1	NM_01398	chr14:8066	NM_01398	Hs.202354
A_22_P00C	-2.504801	-1.324696	2.504801	down		0.56813	0.654378	0.586425	-0.837321	-0.759705	-0.56813	lnc-ZC3H12	lnc-ZC3H12	FALSE	RST31404 Athersys RAGE Library H			1.49E+08	chr6	1.49E+08	-		hs|6q25.1	BG211832	chr6:149348895-14934		Hs.659484
